# Integrative Taxonomic Study of Chinese *Anastatus* (Hymenoptera: Eupelmidae) Based on Morphology and *COI* Barcoding, with Description of Eleven New Species

**DOI:** 10.3390/insects17090918

**Published:** 2026-09-01

**Authors:** Ramzan Khan, Zixuan Li, Haoran Liao, Huayan Chen, Alhadi Adam, Fukiha Kanwal, Lingfei Peng

**Affiliations:** 1 Biological Control Research Institute, Fujian Agriculture and Forestry University, Fuzhou 350002, China; ramzanpanwar158p@gmail.com (R.K.); liaohaoran76@gmail.com (H.L.); alhadialdow@gmail.com (A.A.); 2 China Fruit Fly Research and Control Center of Food and Agriculture Organization/International Atomic Energy Agency, Fuzhou 350002, China; 3 State Key Laboratory of Agricultural and Forestry Biosecurity, Fuzhou 350002, China; fakihak852@gmail.com; 4 State Key Laboratory of Plant Diversity and Specialty Crops, South China National Botanical Garden, Chinese Academy of Sciences, Guangzhou 510650, China; lizixuan1813@163.com (Z.L.); huayanc@scbg.ac.cn (H.C.); 5 Guangdong Provincial Key Laboratory of Applied Botany, Guangzhou 510650, China; 6 University of Chinese Academy of Sciences, Beijing 100049, China; 7 Key Lab of Biopesticide and Chemical Biology, Ministry of Education, College of Plant Protection, Fujian Agriculture and Forestry University, Fuzhou 350002, China

**Keywords:** species delimitation, DNA barcoding, integrative taxonomy, eupelmids, new synonymy, biological control, parasitoid wasps

## Abstract

Thirty-two Chinese *Anastatus* species (Hymenoptera: Chalcidoidea: Eupelmidae) are studied, comprising 20 previously described species and 11 new species. Ten of the new species belong to the subgenus *A*. (*Anastatus*): *A*. (*Anastatus*) *nozdormu* **sp. nov.**, *A.* (*Anastatus*) *kryptos* **sp. nov.**, *A.* (*Anastatus*) *napoleonis* **sp. nov.**, *A.* (*Anastatus*) *yalini* **sp. nov.**, *A.* (*Anastatus*) *battutai* **sp. nov.**, *A.* (*Anastatus*) *beaglei* **sp. nov.**, *A.* (*Anastatus*) *neltharion* **sp. nov.**, *A.* (*Anastatus*) *tigrinus* **sp. nov.**, *A.* (*Anastatus*) *huijuanae* **sp. nov.**, *A.* (*Anastatus*) *ashrafi* **sp. nov.** One belongs to the subgenus *A*. (*Cladanastatus*): *A.* (*Cladanastatus*) *alexstrasza* **sp. nov.** One additional species is newly recorded from China: *A.* (*Anastatus*) *mantoidae* Motschulsky **rec. nov.** A new synonymy is also proposed: *A.* (*Anastatus*) *flavaeratus* Peng & Tang, 2020 **syn. nov.** under *A.* (*Anastatus*) *gastropachae* Ashmead, 1904. Morphologically similar species were assigned to three informal species groups (*formosanus*, *fulloi*, and *japonicus* groups) based on morphology. Of the already-described species of the Chinese *Anastatus*, 15 were newly recorded from different provinces of China; this was true for all species except *A. colemani*, *A. bifasciatus*, *A. polikiarkoudus*, *A. taibaiensis*, and *A. zdenekbouceki*. Molecular analyses based on the mitochondrial cytochrome *c* oxidase subunit I (*COI*) gene were conducted on a total of 34 species (26 species derived from the specimens collected from China and eight species’ sequences downloaded from the BOLD and NCBI). Multivariate morphometric analyses (PCA, LDA, MANOVA) were employed to distinguish morphologically similar species. A comprehensive diagnostic key to all Chinese *Anastatus* species is also provided, accompanied by photographs of diagnostic characters.

## Simple Summary

Parasitoid wasps of the genus *Anastatus* are tiny insects that oviposit into the eggs of agricultural and forest pests, killing them before they can hatch and cause damage to agricultural crops and forest trees. These wasps are already used successfully as natural pest control against different agricultural insect pests in China, Europe, the United States, and other countries. However, identifying these wasps has long been difficult because most of them are morphologically similar. Therefore, in this study, we combined detailed morphology with DNA analysis to revise all known Chinese *Anastatus* species. We examined specimens from 23 provinces and described eleven species new to science, recorded one species in China for the first time, and corrected a previous misidentification by merging two species into one. Species identification is essential when selecting the most effective wasp against a specific pest in an integrated pest management (IPM) program. This work therefore directly supports the development of safer, more targeted biological control strategies that reduce reliance on chemical pesticides.

## 1. Introduction

*Anastatus* Motschulsky, 1859 (Hymenoptera: Eupelmidae) is one of the most widely distributed genera in the family Eupelmidae, with approximately 160 recognized species worldwide [1,2,3,4]. As the fourth genus established in the subfamily Eupelminae by date of description, and the second-most speciose genus after *Eupelmus* Dalman, *Anastatus* exhibits remarkable morphological diversity and a cosmopolitan distribution [5]. This genus has a complex taxonomic history, with 16 accepted synonyms, of which *Cacotropia* Motschulsky, 1863, is the oldest [5]. Currently, the genus is divided into two subgenera, the nominotypical *A*. (*Anastatus*) Motschulsky, which comprises almost all described species, and *A.* (*Cladanastatus*) Bouček, characterized by the type species of *A*. (*Cladanastatus*) *madagascariensis* [6,7] alongside the newly described *A*. (*Cladanastatus*) *alexstrasza* **sp. nov.**

Most *Anastatus* species are primary egg parasitoids of other insects. However, a few have been recorded as hyperparasitoids, attacking primary parasitoids (Hymenoptera: Scelionidae and Ichneumonidae) and developing mainly within Lepidoptera eggs, while some have been reared from Coleoptera larvae and Diptera puparia [8,9]. This adaptability has facilitated the development of several *Anastatus* species as pivotal biological control agents against a variety of agricultural and forest pests. For instance, *A. bifasciatus* (Geoffroy) has been extensively used in Europe against *Halyomorpha halys* (Stål) (Hemiptera: Pentatomidae) [4,10,11]. *A. orientalis* Yang and Choi is currently utilized in the northeastern USA as an effective egg parasitoid against *Lycorma delicatula* (Hemiptera: Fulgoridae) [4,12,13]. Moreover, *A. fulloi* Sheng & Wang (previously identified as *A. japonicus* Ashmead or *Anastatus* sp.) has been successfully used as an egg parasitoid against *Tessaratoma papillosa* (Drury) (Hemiptera: Pentatomidae) in southern China since the 1960s [4], and *A. gansuensis* is deployed against *Caligula japonica* Moore (Lepidoptera: Saturniidae) as an effective biological control agent in northern China [14,15]. The effectiveness of these biocontrol programs depends on accurate species-level identification. However, taxonomic resolution within *Anastatus*, as in many groups within Chalcidoidea, has historically relied mainly on morphology, limiting the understanding of species-level diversity, particularly among cryptic species. Molecular data can reveal such hidden diversity: integrative barcoding, for example, has revealed 16–24 previously undetected cryptic species in the eulophid genera *Oomyzus* and *Quadrastichus* [16], and combined molecular and cytogenetic analysis has resolved species long conflated within *Eupelmus* (*Macroneura*) under a single name [17].

The taxonomic history of Chinese *Anastatus* reflects this same pattern. Peng et al. [4] comprehensively reviewed the group based on morphology alone. Wang et al. [1] subsequently incorporated *COI* barcoding, but molecular data were available for only 13 of the 21 recognized species. They also provided descriptions of seven new species and established a partial phylogenetic framework. Moreover, Li et al. [3] documented morphological variations in the fore wing hyaline crossband with few dark setae medially in *A. gansuensis* and, by providing *COI* sequences of both *A. gansuensis* and *A. gastropachae*, suggested that *A. gastropachae* also exhibits variations in the metallic lustre of the mesoscutum—a point further highlighted in the present study. While these studies supported the validity of many *Anastatus* species, several described and new species remained unsupported by molecular data [1]. Consequently, species boundaries within Chinese *Anastatus* remain incompletely resolved, and patterns of intraspecific versus interspecific genetic divergence are still unclear. Furthermore, the currently available molecular data do not adequately capture the geographic diversity of those species, as evidenced by recent new provincial records of already-described Chinese *Anastatus* species.

Applying this integrative framework, the present study revises the 21 previously recognized Chinese *Anastatus* species, bringing the total to 32. This includes the addition of 12 species: eleven newly described species (ten in *A*. (*Anastatus*) and one in *A*. (*Cladanastatus*)) and the first record of *A.* (*Anastatus*) *mantoidae* from China. Additionally, *A. flaviratus* is proposed as a new synonym of *A. gastropachae*, and 15 species within *A*. (*Anastatus*) are reported as new provincial records. Based on a total of 325 *COI* sequences and supported by ASAP (Assemble Species by Automatic Partitioning), a phylogenetic analysis was conducted for 34 species worldwide (26 from China and 8 from other countries). We were unable to provide molecular analyses for five species (*A. colemani*, *A. polikiarkoudus*, *A. zdenekbouceki*, *A*. *ashrafi* **sp. nov.**, and *A. mantoidae* **rec. nov.**). A detailed key is also provided to all 32 Chinese *Anastatus* based on morphology along with photographs. For clarity, morphologically similar species are informally grouped into three clusters: the *formosanus* groups, the *fulloi* groups, and *japonicus* groups. These are further illustrated in the Results section. All new species are described primarily from females, with diagnostic photomicrographs distinguishing them from described species. The exceptions are *A. dexingensis* and *A. formosanus*, which are described from males based on *COI* gene and morphology.

## 2. Materials and Methods

### 2.1. Sampling of Specimens

This study was based on 462 specimens collected from 23 provinces in China, with collection carried out between 2002 and 2025. Specimens were obtained using Malaise trapping, sweeping or rearing. Specimens were preserved in 95% ethanol at −20 °C until DNA extraction, and subsequently stored at −80 °C after DNA extraction.

Following complete desiccation, voucher specimens and their associated DNA samples were deposited in the Biological Control Research Institute, Fujian Agriculture and Forestry University, Fuzhou, Fujian, China (FAFU). New distribution records presented in this paper are indicated by an asterisk (*).

### 2.2. Imaging

Specimens were examined using a Leica M165C stereomicroscope (Leica Microsystems AG, Heerbrugg, Switzerland) equipped with a Leica LED 5000 HDI dome light source (Leica Microsystems AG, Heerbrugg, Switzerland) and imaged with a Leica MC170 HD digital camera (Leica Microsystems AG, Heerbrugg, Switzerland) attached to the microscope. The resulting image stacks were focus-stacked using Zerene Stacker version 1.04 (Zerene Systems, LLC, Richland, WA, USA). Final images were processed in Adobe Photoshop CC2019 (Adobe Systems Incorporated, Los Angeles, CA, USA) to adjust brightness, contrast, and sharpness.

### 2.3. Morphological Terminology and Abbreviations

Morphological terminology for structure, sculpture, and coloration follows Gibson [18] and the external morphology of the adult Chalcidoidea [19]. The abbreviations used in the description are as follows:

cc = costal cell of the fore wing;

fln = flagellomeres 1 to 8 (e.g., fl1, fl2);

fln_W = flagellomere width;

Gtn = gastral tergite number;

LOL = minimal distance between the anterior and a posterior ocelli;

MPOD = maximum diameter of a posterior ocellus;

mv = marginal vein of the fore wing;

OOL = ocellocular line (minimal distance between a posterior ocellus and the inner orbit);

pedicel_L = pedicel length;

pmv = postmarginal vein of the fore wing;

POL = minimal distance between the posterior ocelli;

scape_L = scape length;

smv = submarginal vein of the fore wing;

stv = stigmal vein of the fore wing.

### 2.4. DNA Extraction, PCR Amplification, Sequencing and Alignment

DNA extraction was performed using the Tiangen DNA Extraction Kit (Tiangen Biotech, Beijing, China), following the manufacturer’s instructions with the following modifications: (i) the gaster of some specimens was pierced with an insect pin to create a hole rather than crushed, whereas most specimens were used directly without any piercing, thereby preserving the specimens while maximizing DNA yield; (ii) lysis incubation was prolonged to at least 12 h at 57 °C in a thermoshaker; and (iii) the adsorption column was equilibrated at room temperature for 3 min before the eluent was added. The *COI* barcode fragment was amplified using the primers LCO1490 (5′-GGTCAACAAATCATAAAGATATTGG-3′) and HCO2198 (5′-TAAACTTCAGGGTGACCAAAAAATCA-3′) [20].

PCR amplification was performed in a 25 μL reaction volume containing 12.5 μL PCR master MIX (containing enzyme, buffer, and dNTPs), 1 μL of each primer (100 μM), 8.5 μL ddH_2_O, and 2 μL of DNA template. The thermal cycling conditions were as follows: initial denaturation at 95 °C for 2 min, followed by 35 cycles of denaturation at 95 °C for 15 s, annealing at 50 °C for 15 s, and extension at 72 °C for 15 s; followed by a final extension at 72 °C for 5 min and a hold at 4 °C. PCR products were visualized by electrophoresis on a 2% agarose gel and subsequently sent to Tingke Biotechnology (Beijing, China) for nanopore and Sanger sequencing. Nanopore sequencing was used for routine *COI* barcoding due to cost efficiency, whereas Sanger sequencing was reserved for verification when multiple templates were suspected. Geneious R11 (Auckland, New Zealand) was used to inspect chromatogram quality, and confirmed sequences were exported in FASTA format.

A total of 325 *COI* sequences were analysed: 267 were newly generated in this study, representing 26 species from 23 Chinese provinces, and 58 were retrieved from public databases. The latter comprised 35 sequences downloaded from the BOLD (Barcode of Life Data, https://www.boldsystems.org/, accessed on 15 January 2026) system, including both unidentified and already identified species, and 23 sequences of previously described species from outside their type localities (provided in Supplementary Table S1). These included *A. acherontiae* Narayanan, Subba Rao & Ramachandra Rao, *A. bangalorensis* Mani & Kurian, *A. semiflavidus* Gahan, *A. mirabilis* Walsh & Riley, *A. bifasciatus* Geoffroy, *A. sidereus* Erdös, *A. orientalis* Yang & Choi, *A. echidna* Motschulsky, *A. meilingensis* Sheng, *A. tenuipes* Bolívar y Pieltain, *A. temporalis* Askew, and *A. japonicus* Ashmead.

Sequences were aligned using ClustalW in Geneious R11 and checked by translation to amino acid sequences to confirm the absence of stop codons or frameshifts. All newly generated sequences were submitted to GenBank (NCBI); accession numbers are provided in Supplementary Table S1 (entries 1–267 are new to this study, and entries 268–325 were retrieved from the BOLD and GenBank, https://www.ncbi.nlm.nih.gov/genbank/, accessed on 15 January 2026).

### 2.5. ASAP Species Delimitation and Phylogenetic Analysis

To objectively delimit species boundaries, we employed an integrative taxonomic framework combining morphological examination with molecular species delimitation. Initial morphospecies sorting was performed based on discrete morphological features. Subsequently, ASAP was conducted on a K2P (Kimura 2-Parameter) distance matrix to identify potential molecular operational taxonomic units (MOTUs). Among the ranked partitioning schemes generated by ASAP, we selected the optimal partition based on the lowest ASAP score (i.e., the highest slope break in the distance distribution), which represents the most statistically supported grouping. Species boundaries were then confirmed when morphological clusters and molecular MOTUs were congruent. In cases of conflict between morphological and molecular data (e.g., *A. makrysourus* splitting into two MOTUs, *A. shichengensis* into three, and *A. huijuanae* **sp. nov.** into two), we conservatively retained a single species designation if no diagnostic morphological differences were detected, treating the molecular divergence as an intraspecific variation. Conversely, if molecular partitions correlated with consistent morphological differences, they were recognized as distinct species.

A maximum likelihood (ML) tree was inferred from the 325 *COI* sequences, with *Zaischnopsis* sp. (GenBank ID: MZ868006) designated as an outgroup. The ML tree was constructed using IQ-TREE **version 3.0.1** within PhyloSuite under the TIM + R2 + F model, with 5000 ultrafast bootstrap replicates and the Shimodaira-Hasegawa-like approximate likelihood ratio test. The tree was visualized using iTOL v.5. Bootstrap values ranged from 70% to 100% [21,22,23,24]. The resulting phylogenetic tree was interpreted with respect to species monophyly and bootstrap support. Finally, Adobe Illustrator (Adobe Inc., San Jose, CA, USA) was used to refine the aesthetic value of the phylogenetic tree.

### 2.6. Morphometric Analyses

Morphometric analyses were performed in Python 3.11. Data management and preliminary calculations were cross-checked in Microsoft Excel (Microsoft 365). Principal component analysis (PCA) was performed on 21 antennal characters (lengths and widths of the scape, pedicel, flagellomeres, and clava). Linear discriminant analysis (LDA) with leave-one-out cross-validation was used to test species classification accuracy. Additionally, multivariate analysis of variance (MANOVA) was conducted to test for significant differences in antennal morphometrics among species [25,26].

Kernel density estimation (KDE) plots and boxplot visualizations were generated to visualize distributions [27]. Pairwise comparisons between morphologically similar species were made using Welch’s two-sample *t*-tests (unequal variances assumed) to accommodate unequal sample sizes and variances [28]. Effect sizes were quantified using Cohen’s d (calculated as the mean difference divided by the pooled standard deviation) [29], and diagnostic utility was assessed via the area under the receiver operating characteristic curve (AUC). All analyses and data management were conducted using the following libraries in Python 3.11: NumPy [30], SciPy [28], scikit-learn [26], statsmodels [25], matplotlib [31], and seaborn [27].

## 3. Results

### 3.1. Phylogenetic Analysis Based on COI Sequences

Some BOLD sequences lacking species-level identification clustered with previously described species from China, while others grouped with the newly described species. For example, two specimens from Thailand (THAMJ1185-23, THAMH5106-23) were identified as *A. shichengensis*, and two additional Thailand specimens (THAMG333-23, THAMG12611-23) as *A. makrysourus*. One specimen from Thailand (THAMJ15436-23) was identified as *A. kryptos* **sp. nov.**, and one specimen from Ivory Coast (CIBFB9416-25) was identified as *A. nozdormu* **sp. nov**. In addition, six specimens, selected as representatives from a larger pool of conspecific sequences sharing the same BOLD identification, were identified as *A. gansuensi*, including three from Bulgaria (GMBUA1120-14, GMBSV699-18, GMBSR1276-18), two from Portugal (LPSYA1994-23, LPSYA1969-23), and one from Germany (AMTPA267-15).

**Figure 1:**
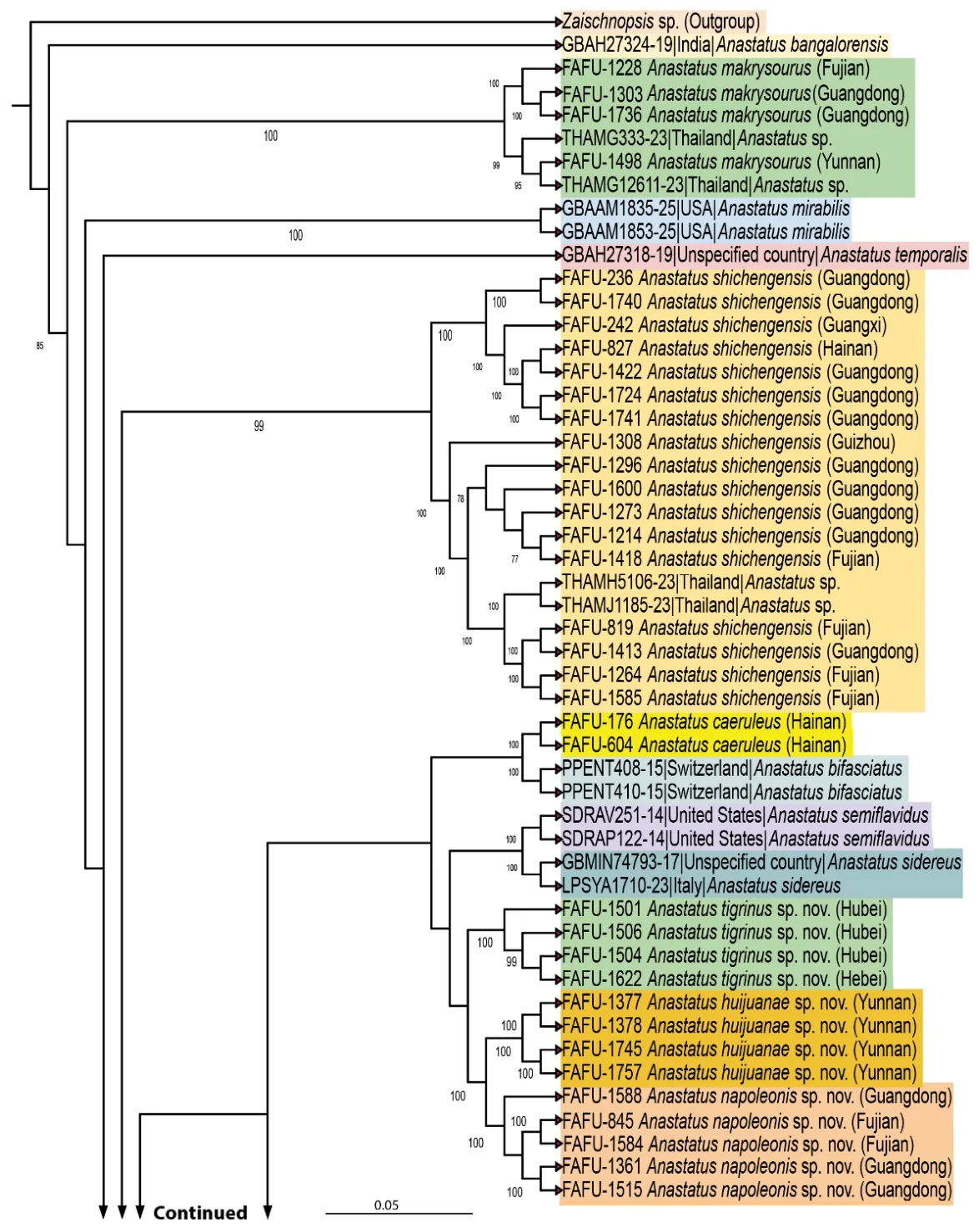

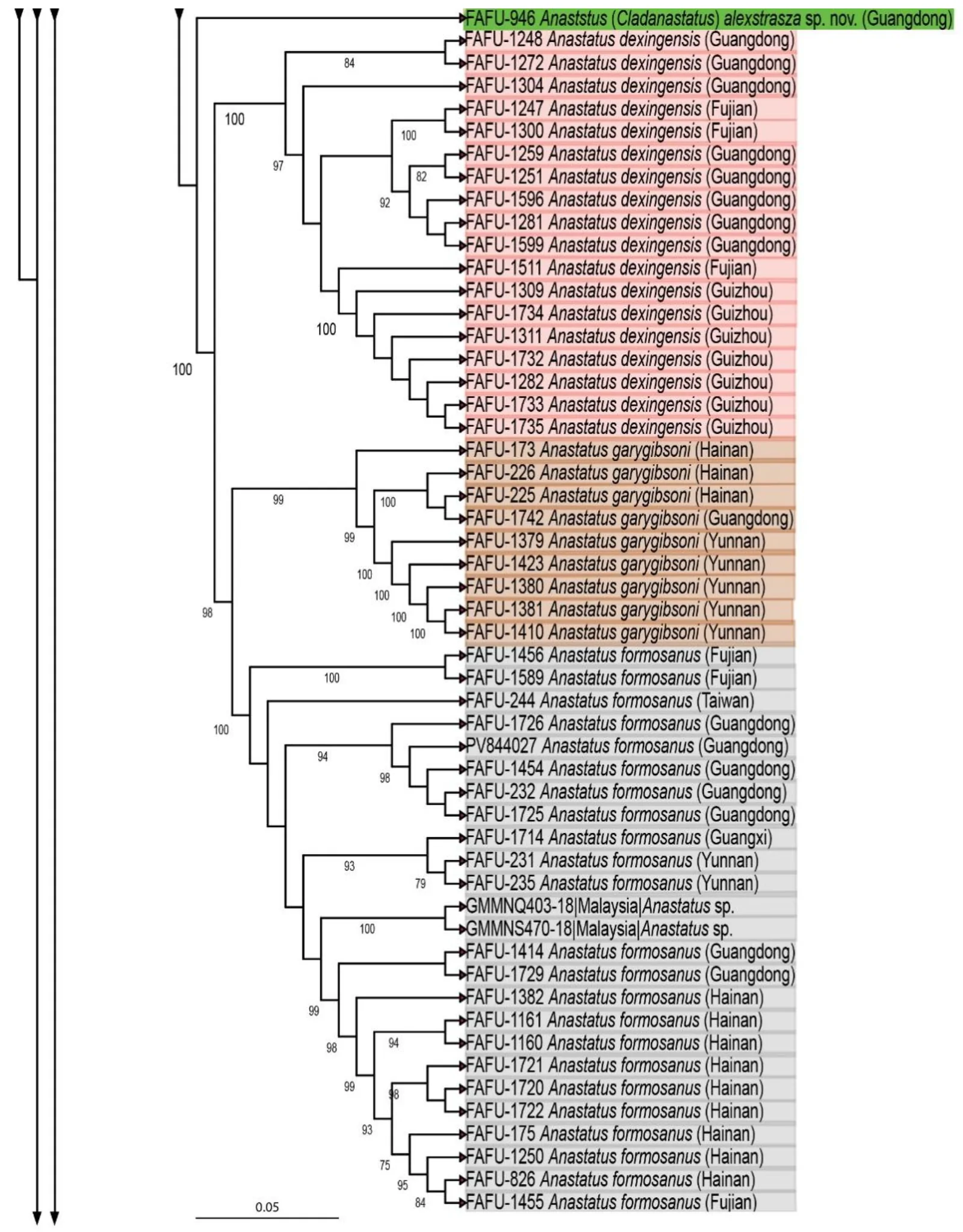

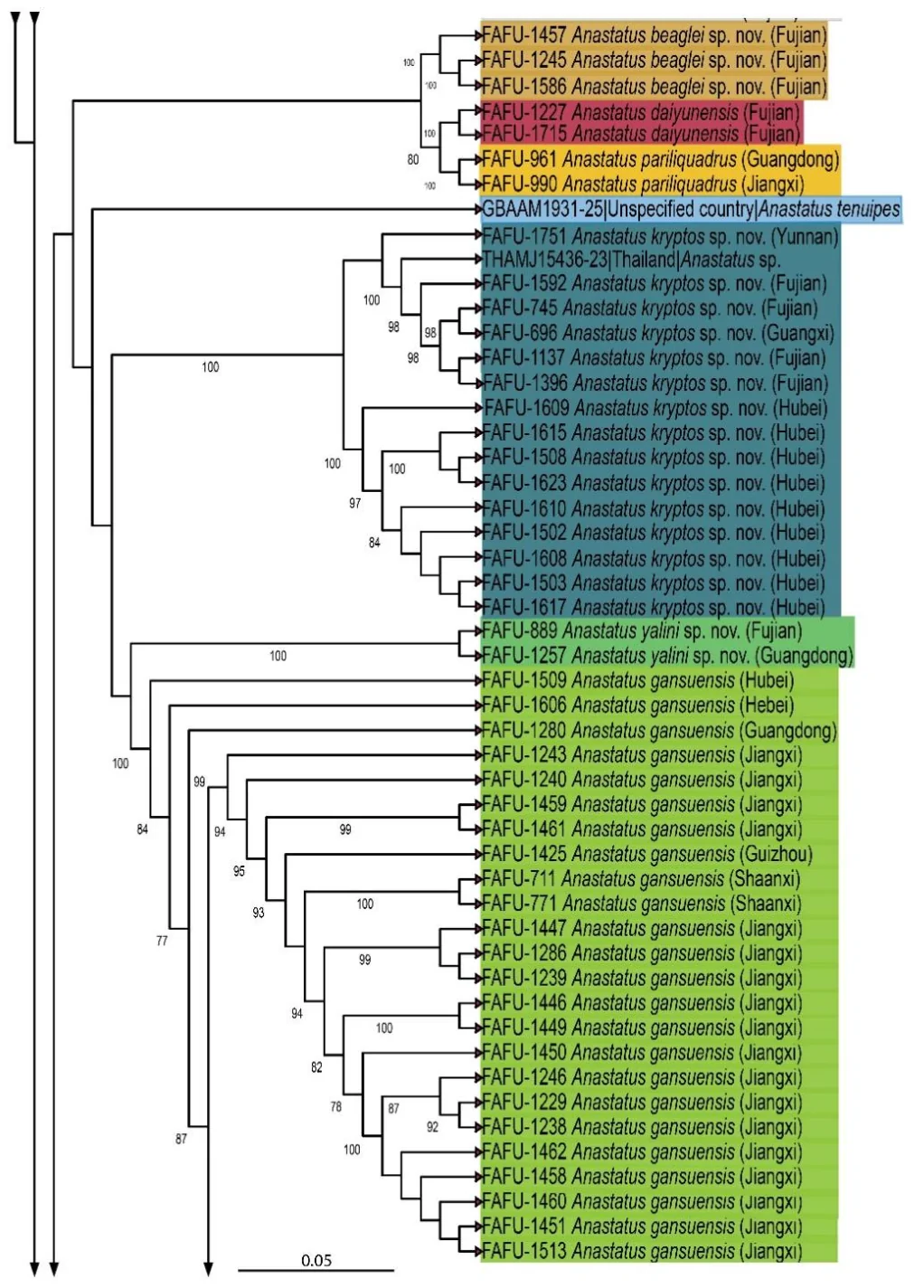

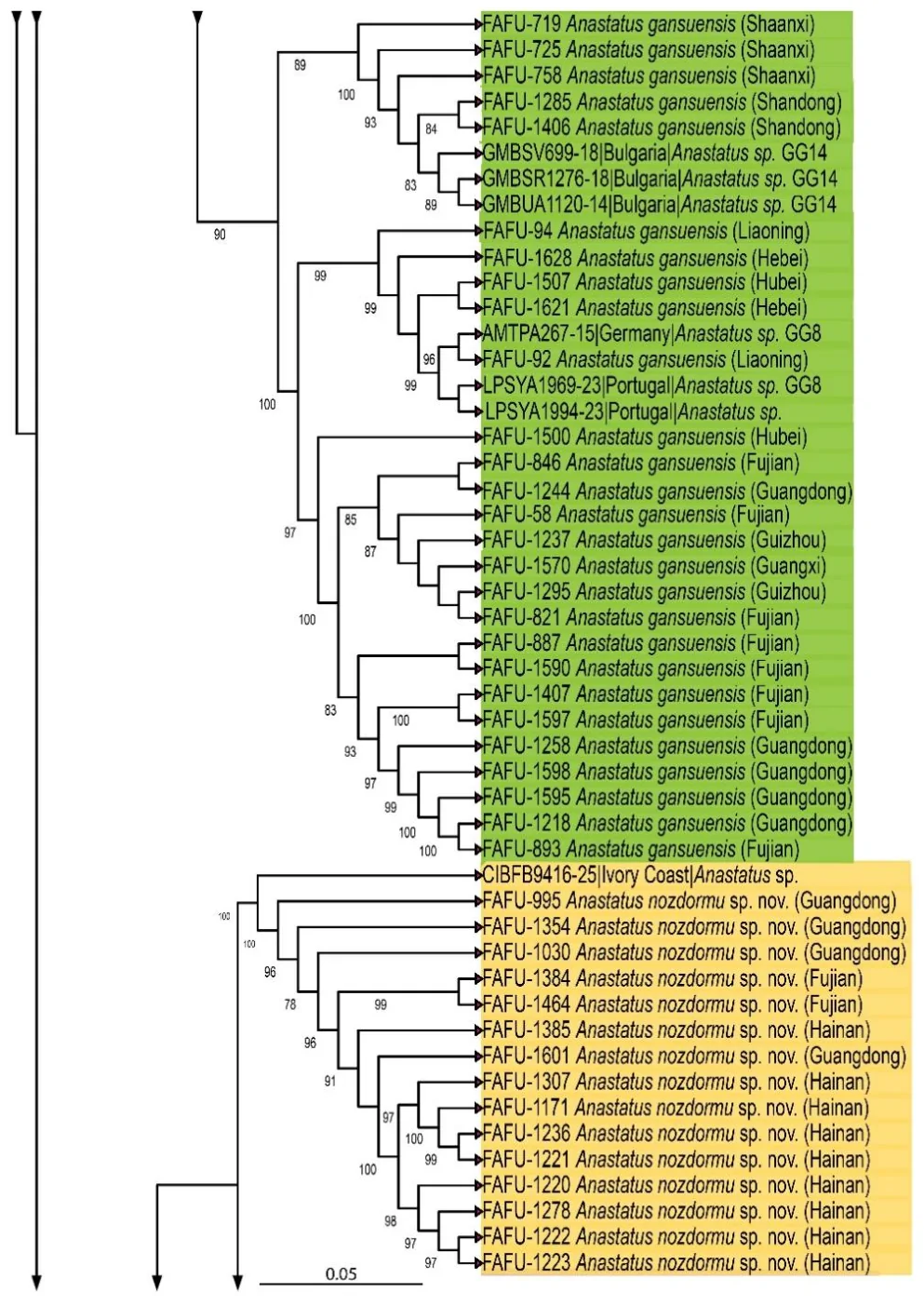

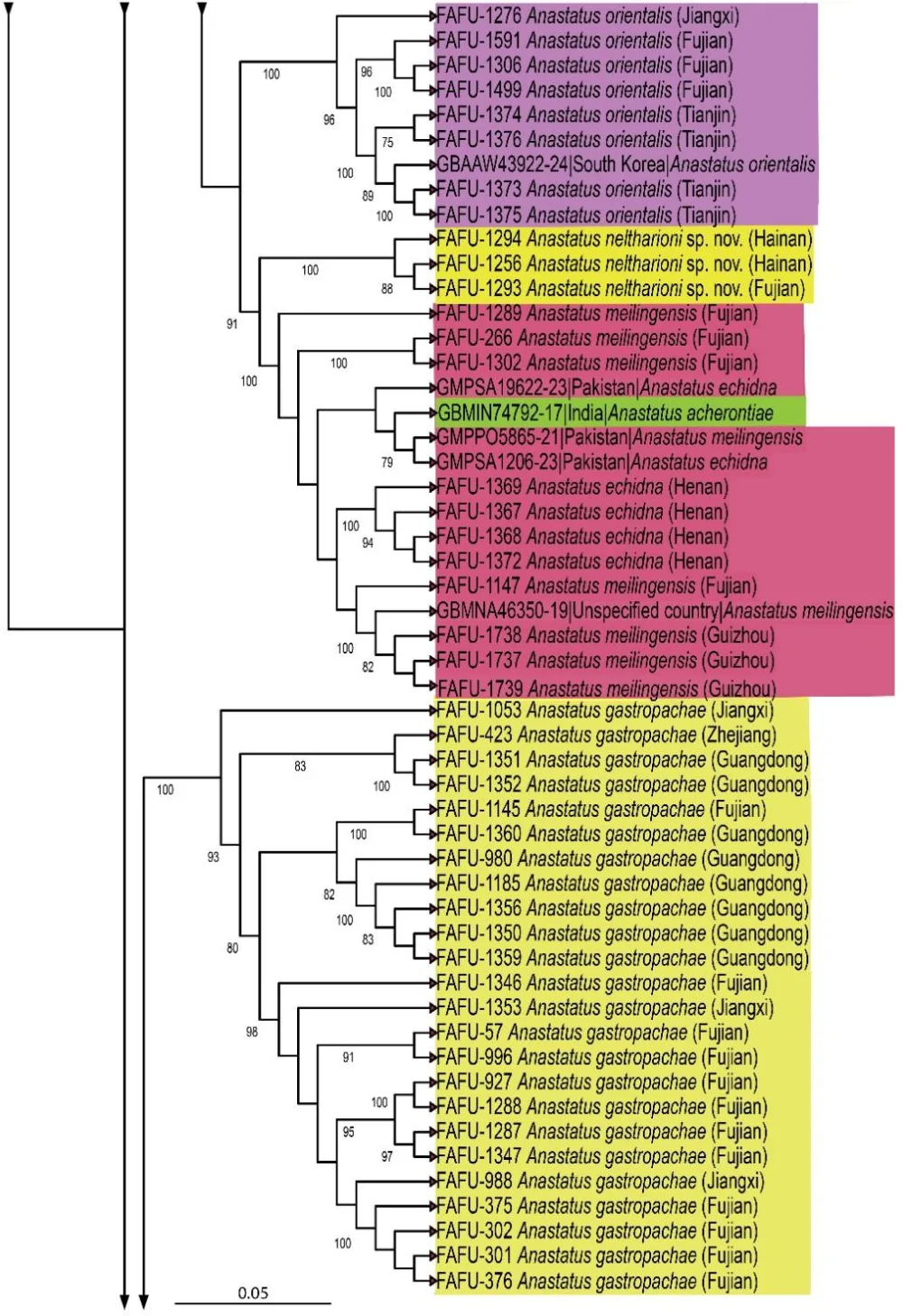

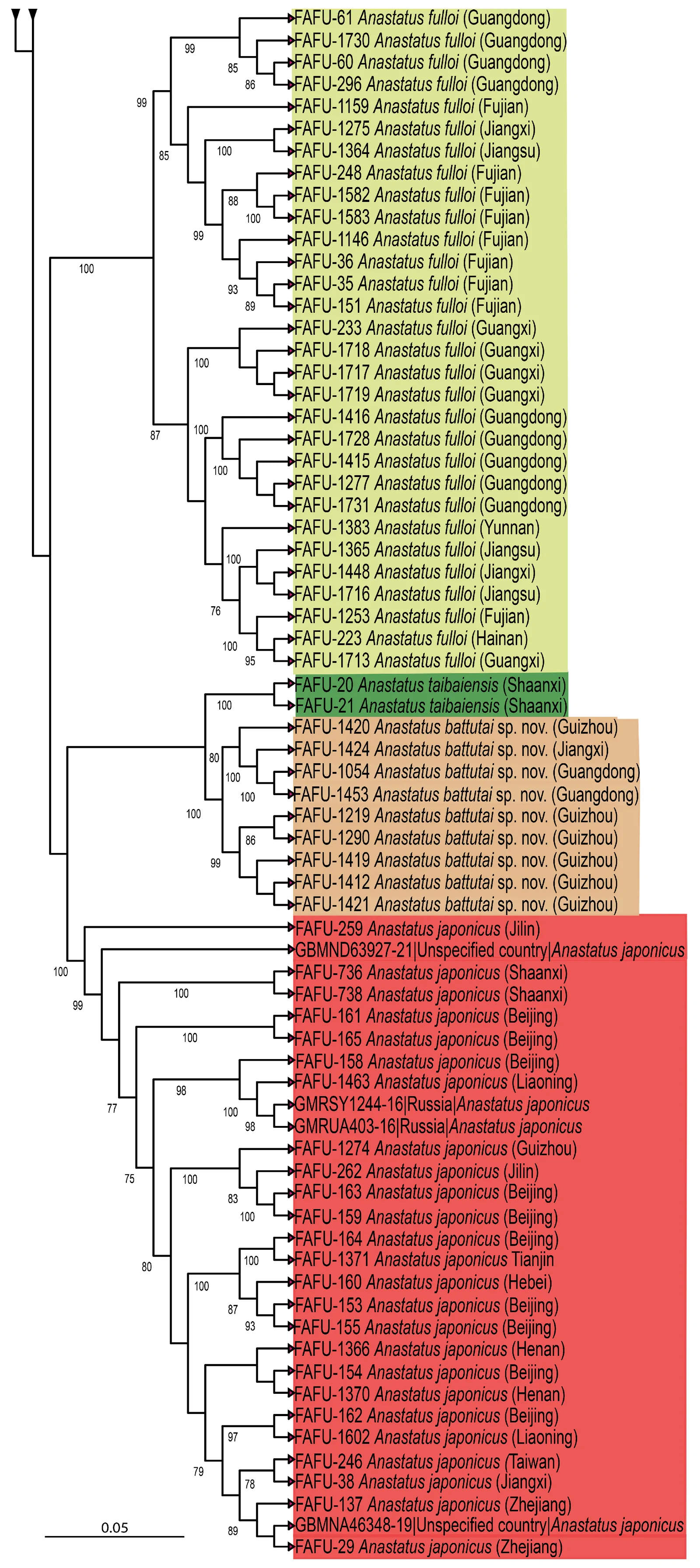
Maximum likelihood (ML) phylogenetic tree of *Anastatus* species based on the mitochondrial cytochrome c oxidase subunit I (*COI*) gene. The tree was constructed using IQ-TREE with the best-fit substitution model. Bootstrap support values (≥70%) are shown at the nodes. *Zaischnopsis* sp. was used as the outgroup. Different colours represent different species (except for *A. echidna* and *A. meilingensis*, which share the same colour).

The overall *COI* analysis illustrated the relationships of 34 *Anastatus* species from different countries. The ASAP analysis largely supported the *COI*-based delimitations, although *A. makrysourus*, *A. huijuanae* **sp. nov.**, and *A. shichengensis* presented incongruences that are detailed in the remarks on each species in this section and in the Discussion section. All *Anastatus* species formed monophyletic clades in the maximum likelihood (ML) tree, except for *A. echidna* and *A. meilingensis*, which are further explained in the Discussion section (Figure 1).

Despite the broad geographic scale of sampling, encompassing 23 provinces, no consistent morphological differentiation was observed among geographically distant populations within any species. Specimens assigned to the same species based on morphology clustered together in the ML tree regardless of provincial origin, and no clade structure within nominal species correlated with geographic provenance. Although ASAP analysis revealed multiple putative MOTUs within *A. makrysourus*, *A. shichengensis*, and *A. huijuanae* **sp. nov.**, these partitions did not correspond to discrete geographic ranges or diagnosable morphological differences. Consequently, the phylogenetic results provided no evidence of cryptic species among the Chinese material; instead, the observed molecular substructure was interpreted as intraspecific geographic variation pending support from additional nuclear markers.

Accurate identification of *Anastatus* species is challenging due to their conserved general habitus and subtle interspecific differences. To facilitate practical identification and comparative diagnosis, 16 of the 32 Chinese species were arranged into three informal species groups based on readily observable external characters. The *formosanus* group, characterized by the profemoral tooth structure, comprises *A. garygibsoni*, *A. formosanus*, and *A. dexingensis*. The *fulloi* group, characterized by the dark procoxae, comprises *A. taibaiensis*, *A. gansuensis*, *A. kryptos* **sp. nov.**, *A. orientalis*, *A. battutai* **sp. nov.**, and *A. fulloi*. Similarly, the *japonicus* group, characterized by the acropleuron colouration (distinctly paler than dark mesoscutum) comprises *A. tigrinus* **sp. nov.**, *A. napoleonis* **sp. nov.**, *A. huijuanae* **sp. nov.**, *A. nozdormu* **sp. nov.**, *A. yalini* **sp. nov.**, *A. beaglei* **sp. nov.**, and *A. japonicus*. These groupings were used below solely to facilitate comparison among similar species; they were not interpreted as phylogenetic clades except the *formosanus* group. The remaining 16 species did not share sufficient diagnostic similarity to be placed in any of these groups and were treated individually.

### 3.2. Description of New Species

#### 3.2.1. *Anastatus* (*Anastatus*) *ashrafi* Khan, Li & Peng, sp. nov. (Figure 2)

Zoobank:urn:lsid:zoobank.org:act:D2E1C19D-63C2-422F-9059-7D446734ECFD

**Figure 2:**
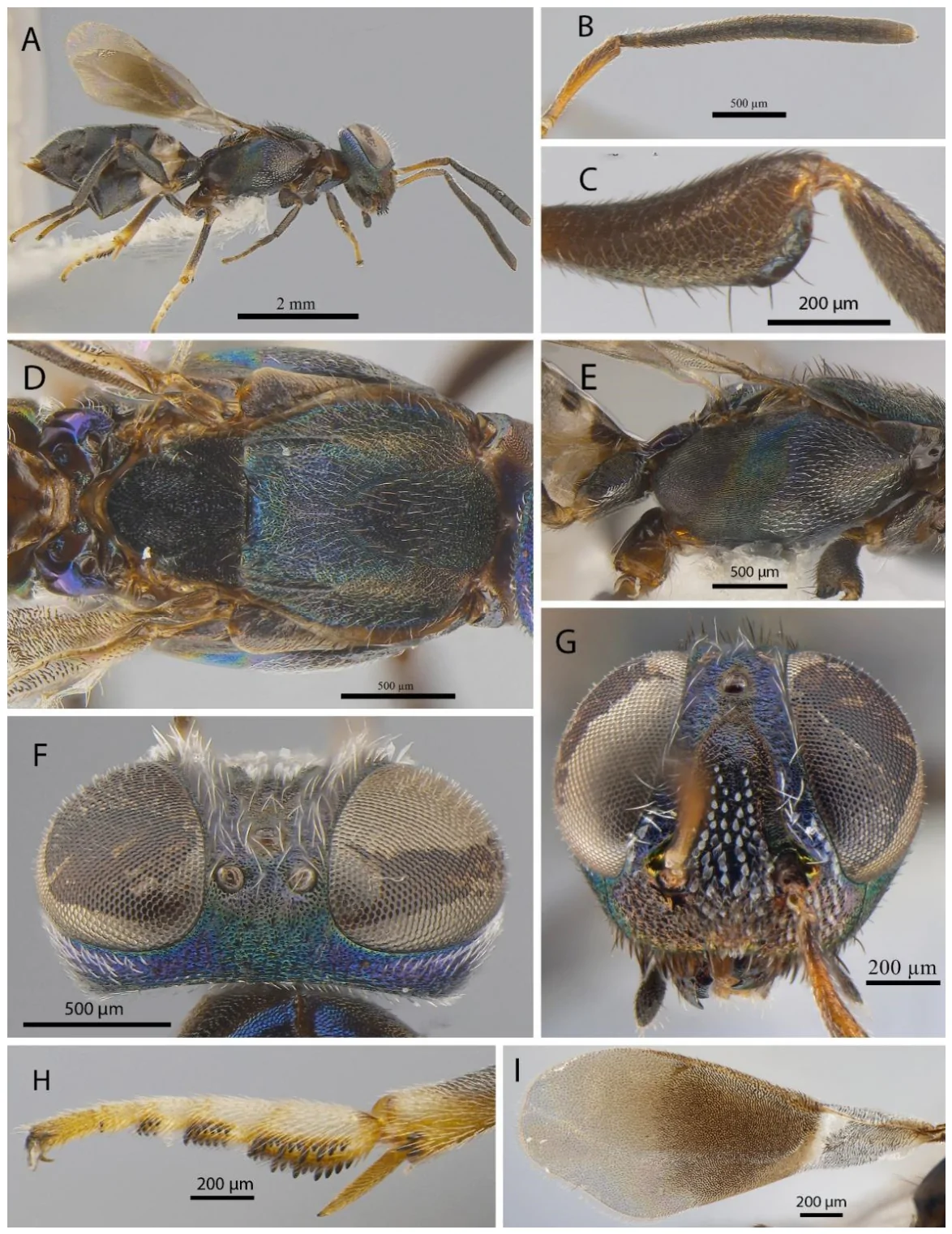
*Anastatus* (*Anastatus*) *ashrafi* **sp. nov.** holotype ♀ (FAFU-1577): (**A**) body lateral; (**B**) antenna; (**C**) profemur; (**D**) mesosoma; (**E**) acropleuron; (**F**) head dorsal; (**G**) head front; (**H**) mesotibial spur and mesotarsus; (**I**) fore wing.

**Type material. Holotype.** ♀(FAFU), Shangsi, Shiwandashan, Guangxi Province, China, 16.VIII.2020, Li Tao leg., FAFU-1577.

**Etymology.** The species epithet *ashrafi* is a genitive noun honouring Mr. Muhammad Ashraf, father of the first author, in recognition of his support for the author’s scientific research.

**Description of holotype. FEMALE.** Body length excluding ovipositor sheaths 5.8 mm (Figure 2A).

**Head.** Temple and vertex mostly blue with slight green lustre; frons slightly blue to dull grey; ocellus dark brown (Figure 2F,G); upper face violet blue with slightly yellowish lustre, lower face and base of torulus yellowish green to reddish purple; torulus dark brown; gena bluish green to violaceous; clypeus yellowish blue; labrum and mandible dark brown. Temple and frontovertex deeply reticulate; upper face and lower face strongly reticulate with scrobal depression slightly mesh-like alutaceous. Vertex and temple with long white setae, while interantennal prominence and lower face with dense scale-like white setae. Antenna (Figure 2B) dark violet with slight green lustre, densely setose with dark brown setae except scape pale yellow to white; pedicel violet bluish green with pale brown to white setae. Relative length (width) of scape 85.9(14.3), pedicel 17.0(10.2), fl1–fl8: 7.9(10.0), 23.9(10.9), 25.9(12.2), 28.8(14.6), 23.6(16.6), 20.4(18.0), 19.0(18.9), 17.1(19.0), clava 41.7(19.4); OOL: POL: LOL: MPOD = 1.0: 4.3: 3.0: 3.0.

**Mesosoma.** Overall dark bluish to pale brown (Figure 2D); pronotum with dark violet bluish spots laterally; mesonotum, scutellum, and axillae violet bluish with mesoscutal lateral lobes slightly pale brownish lustre; metanotum and propodeum dark brown medially, while bluish laterally. Mesoscutum strongly punctate-reticulate; scutellar–axillar complex reticulate with few or no setae. Acropleuron (Figure 2E) dark violet blue, except medially strongly bluish; mesopectus densely setose with white setae; overall alutaceous. Legs (Figure 2A,C,E,H) overall dark violet blue with slight green lustre; mesotarsus pale brown to white except mesotarsal pegs dark, spur light brown. Fore wing macropterous (Figure 2I), without hyaline crossband, densely setose with dark setae, except apical margin with light brown setae. Relative measurements of smv:mv:pmv:stv = 5.9:6.5:2.3:1.0.

**Gaster.** Overall dark violet-blue, except Gt1 and Gt2 white to pale brown, with dark spots dorsally and green metallic lustre ventrally (Figure 2A); coriaceous to slightly alutaceous; ovipositor sheaths dark to pale brown; longer than half of mesotibial apical spur.

**Distribution.** ORIENTAL: China (Guangxi).

**Host.** Unknown.

**Diagnosis.** *A. ashrafi* is distinguished from other Chinese species of the subgenus *A*. (*Anastatus*) by the following combination of characters: fore wing without hyaline crossband behind marginal vein (Figure 2I); mesoscutum mostly punctate-reticulate (Figure 2D); antennal fl2 no longer than clava (Figure 2B); head in frontal view with scale-like white setae (Figure 2G).

#### 3.2.2. *Anastatus* (*Anastatus*) *battutai* Khan, Li & Peng, sp. nov. (Figure 3)

Zoobank:urn:lsid:zoobank.org:act:1B2D5D60-1730-4DAD-A81F-DCA02AB81FC2

**Figure 3:**
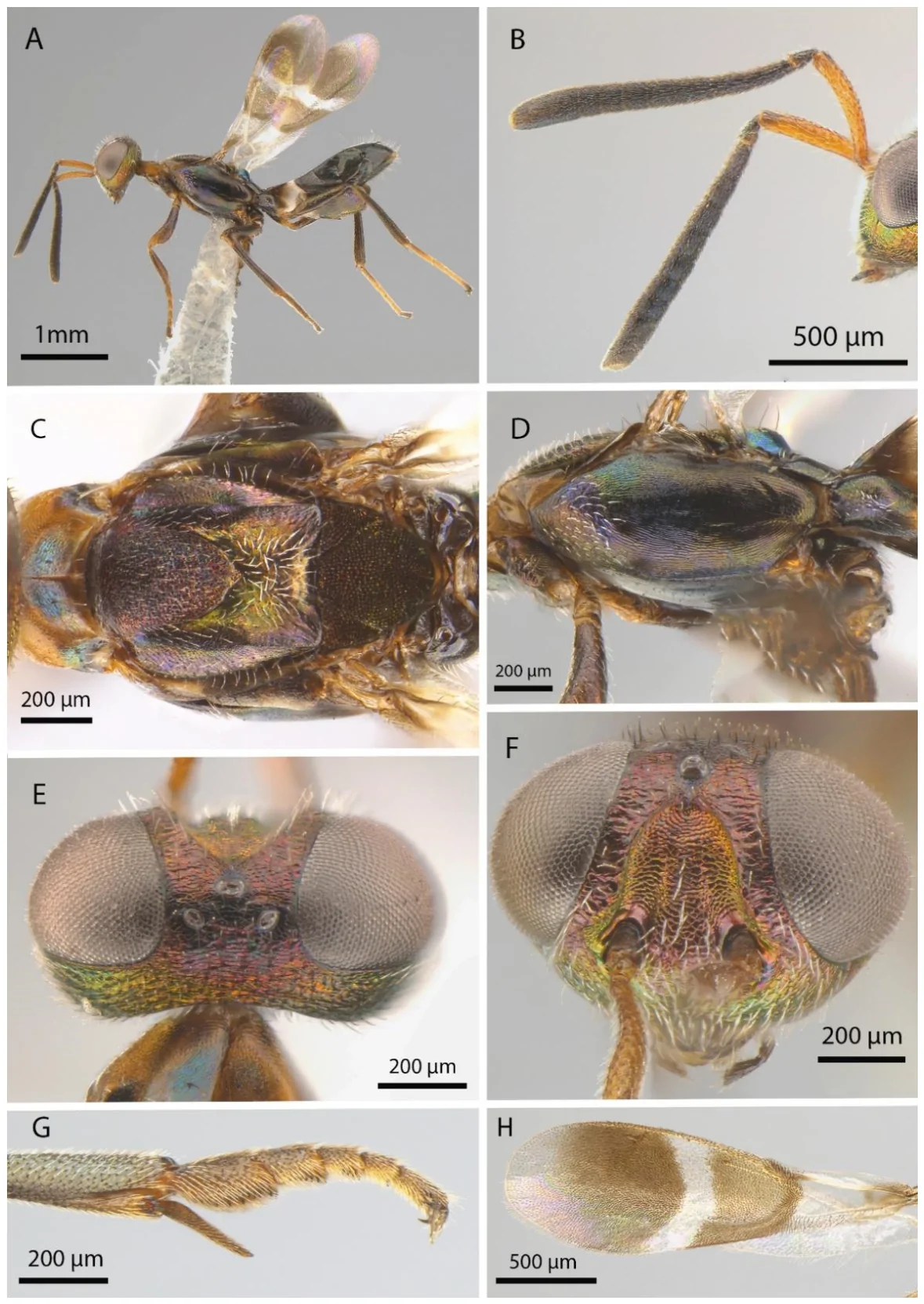
*Anastatus* (*Anastatus*) *battutai* **sp. nov.** holotype ♀ (FAFU-1424, except for **G**): (**A**) body lateral; (**B**) antennae; (**C**) mesosoma; (**D**) acropleuron; (**E**) head dorsal; (**F**) head front; (**G**) mesotibial spur and mesotarsus (FAFU-1453); (**H**) fore wing.

**Type material. Holotype.** ♀(FAFU), Xingangshan, Jiangxi Province, China, 15.V.2022, FAFU-1424. **Paratypes.** 1♀(FAFU), Daiyun Mountain, Dehua, Quanzhou, Fujian Province, China, 15.VI.2015, FAFU-270; 1♀(FAFU), Haizhu Wetland, Guangzhou, Guangdong Province, China, 22.III–22.IV.2022, FAFU-978; 1♀(FAFU), Haizhu Wetland, Guangzhou, Guangdong Province, China, 29.III.2022, FAFU-1023; 1♀(FAFU), Guizhou Province, China, 10–21.XI.2020, FAFU-1219; 1♀(FAFU), Huaping National Nature Reserve, Longsheng, Guilin, Guangxi Zhuang Autonomous Region, China, 23.VIII.2020, FAFU-1263.

**Non-type material.** 1♀(FAFU), Guizhou Province, China, 18–27.VII.2020, FAFU-1290; 1♀(FAFU), Guizhou Province, China, 10–21.XI.2020, FAFU-1412; 2♀(FAFU), Hesha Ba, Guizhou Province, China, 19.IX–7.X.2021, FAFU-1419,1420; 1♀(FAFU), Guizhou Province, China, 20.VIII–4.IX.2020, FAFU-1421; 1♀(FAFU), Chebaling, Guangdong Province, China, 10.VI.2023, FAFU-1453.

**Etymology.** The species epithet *battutai* is a Latinized genitive masculine patronym honouring Ibn Battuta (1304–1369), the renowned Moroccan scholar and greatest medieval traveller, who journeyed approximately 75,000 miles across Africa, Asia, and Europe over 29 years, documenting the geography and cultures of the 14th-century world in his *Rihla*. The name is given in recognition of the species’ wide distribution across five provinces of southern China, reminiscent of his extraordinary journeys across the known world.

**Description of holotype. FEMALE.** Body length excluding ovipositor sheaths 3.4 mm (Figure 3A).

**Head.** Temple greenish blue to yellow (Figure 3E,F); frontovertex violet blue to volcanic red with slight green lustre; ocellus pale to dark grey in dorsal view; parascrobal region, scrobal depression, and interantennal prominence mostly volcanic red with violet blue to green metallic lustre in frontal view; gena and clypeus volcanic red with bluish green to yellowish metallic lustre; labrum and mandible dark brown; torulus dark brown. Temple mesh-like coriaceous to alutaceous; frontovertex slightly mesh-like coriaceous to alutaceous; upper and lower face coriaceous to reticulate; lower face, lower parascrobal region, lower part of interantennal prominence, frons, and temple densely setose with dark brown to white setae. Antenna (Figure 3B) with scape yellowish brown, slightly dark brown posteriorly; pedicel and flagellum bluish purple with green lustre, and densely setose with pale to dark brown setae. Relative length (width) of scape 74.4(12.0), pedicel 19.5(10.3), fl1–fl8: 7.6(10.0), 17.8(12.7), 20.2(15.3), 20.7(17.3), 18.5(19.1), 15.6(19.6), 16.2(19.5), 15.5(20.4), clava 40.9(19.6); OOL:POL:LOL:MPOD = 1.0:2.8:1.5:1.3.

**Mesosoma.** Overall dark violet blue to volcanic red; pronotum slightly yellowish brown anteriorly, with dark bluish purple to black spots with green metallic lustre laterally and medially (Figure 3C); mesoscutal medial lobe with anterior convex region dark violet blue to volcanic red, lateral lobes violet blue with slight yellowish to green metallic lustre, posterior depressed region dark violet blue marginally, but yellowish green to violet bluish spots medially; scutellar–axillar complex dark bluish purple to green; propodeum dark brown. Pronotum weakly coriaceous to alutaceous; mesoscutal medial lobe with anterior convex region punctate-reticulate; lateral lobes mesh-like reticulate to considerably transversely alutaceous; posterior depressed region smooth, with margin slightly coriaceous to alutaceous and densely setose with white setae; scutellar–axillar complex strongly reticulate. Acropleuron (Figure 3D) dark with greenish blue metallic lustre, and completely alutaceous. Legs (Figure 3A,D,G) overall dark, except mesotarsus pale; pro- and mesocoxa dark brown with green metallic lustre; procoxa with dense white setae; metacoxa violet blue with white setae laterally; mesotarsal pegs and claws considerably darker; spur dark brown. Fore wing macropterous (Figure 3H), densely setose with dark setae except hyaline crossband behind mv; basal cell without dark setae; costal cell with few lanceolate setae; parastigma of smv densely setose with dark setae. Relative measurements of smv:mv:pmv:stv = 7.8:6.4:3.5:1.0.

**Gaster.** Overall dark except Gt1 and Gt2 pale brown to white (Figure 3A). Gaster longer than mesosoma; overall coriaceous to alutaceous, and densely setose with pale brown to white lanceolate setae posteriorly; ovipositor sheaths pale brown and slightly exserted in lateral view.

**Distribution.** ORIENTAL: China (Jiangxi, Guangdong, Guizhou, Guangxi, Fujian).

**Host.** Unknown.

**Figure 4:**
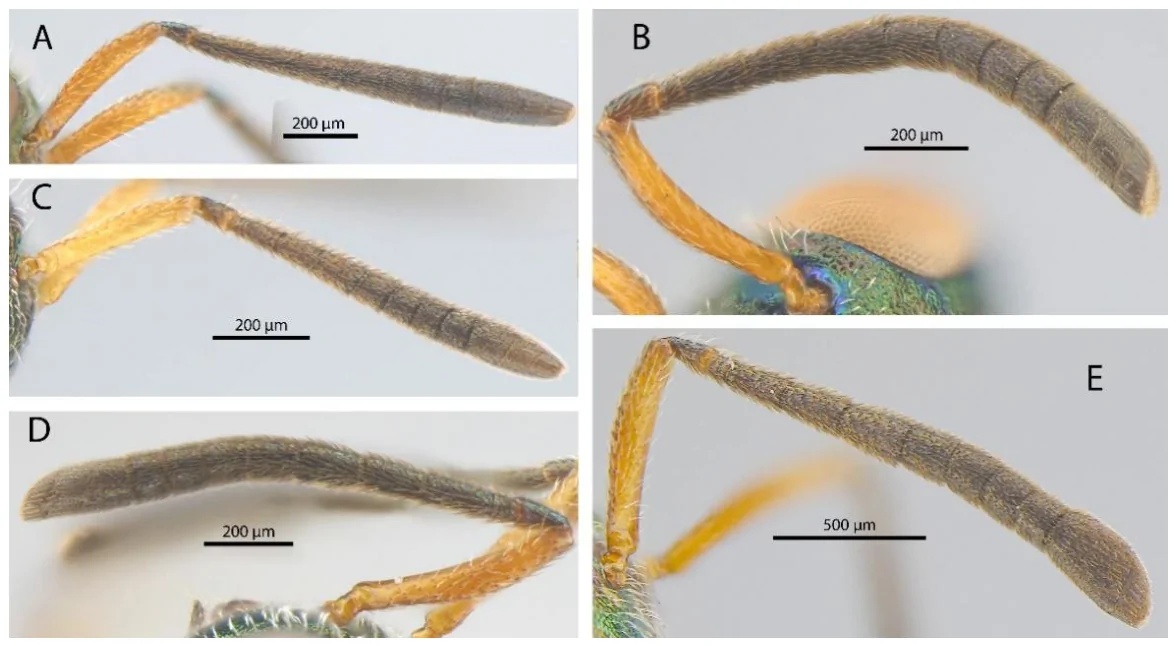
Antennae of *A. fulloi* ♀ and *Anastatus* (*Anastatus*) *battutai* **sp. nov.** ♀, *A*. *fulloi*: (**A**) (FAFU-1383), (**C**) (FAFU-1277), (**D**) (FAFU-1046); *A*. *battutai*: (**B**) (FAFU-270), (**E**) (FAFU-1421).

**Diagnosis.** *A. battutai* is morphologically similar to *A. fulloi*, but differs in antennal fl2 and fl4 markedly wider than in *A. fulloi* (Figure 4B,E); female gaster consistently longer than mesosoma (Figure 3A). Detailed comparisons are provided in the remarks section below.

**Remarks.** *A. battutai* is morphologically close to *A. fulloi*, but differs in antennal proportions: the fl2_W and fl4_W are wider, and the pedicel_L and scape_L are shorter (Figure 4).

**Figure 5:**
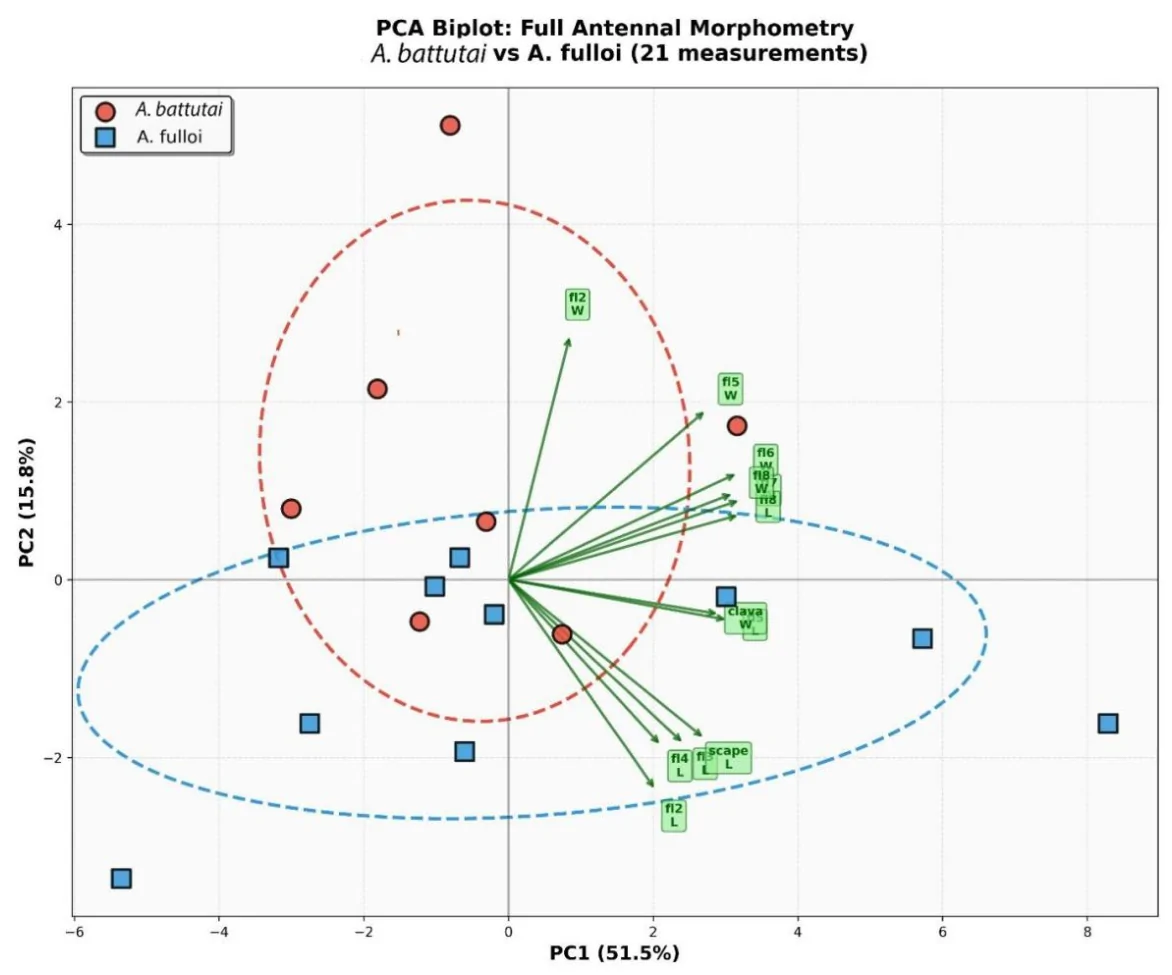
Principal component analysis of 21 antennal characters (excluding fl1_W due to zero variance) separating *A. battutai* (red circles, n = 7) from *A. fulloi* (blue squares, n = 10) along PC1 (51.5% variance) and PC2 (15.8% variance), with cumulative variance of 67.3%. Dashed ellipses = 95% confidence regions. Green arrows: variable loadings. fl2_W showed the strongest discriminatory power (Welch’s *t*-test: *p*-value < 0.001).

A principal component analysis (PCA) of 21 antennal characters (lengths and widths of the scape, pedicel, flagellomeres and clava; excluding fl1_W due to zero variance) separated *A. battutai* from *A. fulloi* along PC1 (51.5% variance explained) and PC2 (15.8% variance explained), with cumulative explained variance of 67.3% (Figure 5).

**Figure 6:**
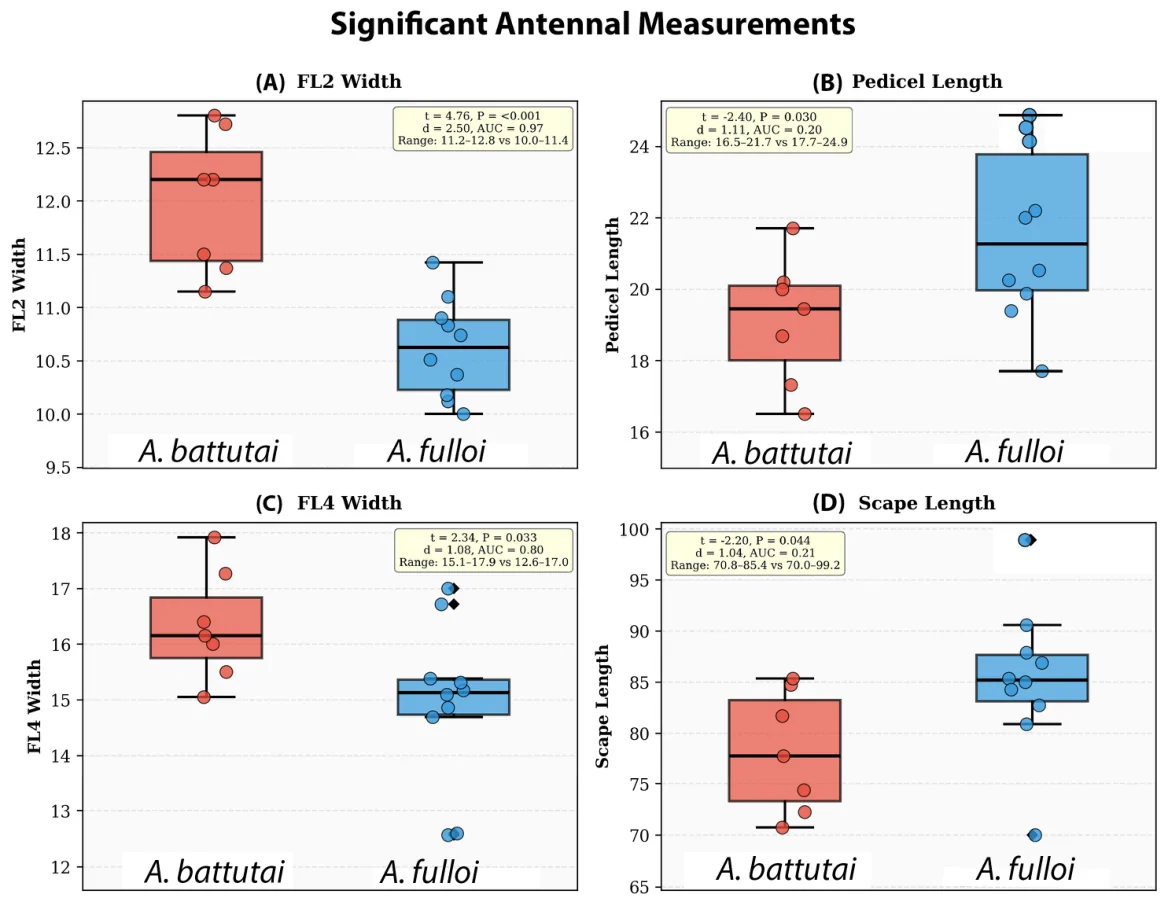
Morphometric differentiation of *A. battutai* (blue) and *A. fulloi* (red) based on antennal characters. (**A**) boxplot of fl2 width; (**B**) pedicel length; (**C**) fl4 width; (**D**) scape length. (Boxplots show median and individual data points. *A. battutai* displays wider fl2_W. Statistics: Welch’s *t*-test: t_10_ = 4.76, *p* < 0.001, with Cohen’s d = 2.50 and AUC = 0.97).

*A. battutai* exhibited significantly wider fl2 and fl4 than *A. fulloi* (Figure 6A,C), whereas *A. fulloi* had a longer scape and pedicel (Figure 6B,D). Among these characters, fl2_W showed the strongest discriminatory power (Welch’s *t*-test: t_10_ = 4.76, *p* < 0.001), with a very large effect size (Cohen’s d = 2.50) and the highest univariate discriminatory ability (AUC = 0.97). The width of fl4, pedicel length, and scape length were also significant but had comparatively lower diagnostic utility (Figure 6).

When analysed together, the four characters significantly distinguished the two species (MANOVA: Wilks’ λ = 0.300, F_3,13_ = 10.12, *p* = 0.001), corroborating the morphological patterns depicted in the PCA biplot and boxplots (Table 1). In addition to the multivariate analysis of antennal measurements (PCA: 100% LDA cross-validation accuracy), the *COI*-based ML tree recovered the two species as reciprocally monophyletic lineages with strong bootstrap support (99% and 100%), consistent with substantial genetic divergence.

#### 3.2.3. *Anastatus* (*Anastatus*) *beaglei* Khan, Li & Peng, sp. nov. (Figure 7)

Zoobank:urn:lsid:zoobank.org:act:0DB8B328-72AB-4A48-9003-3A737BFC859C

**Figure 7:**
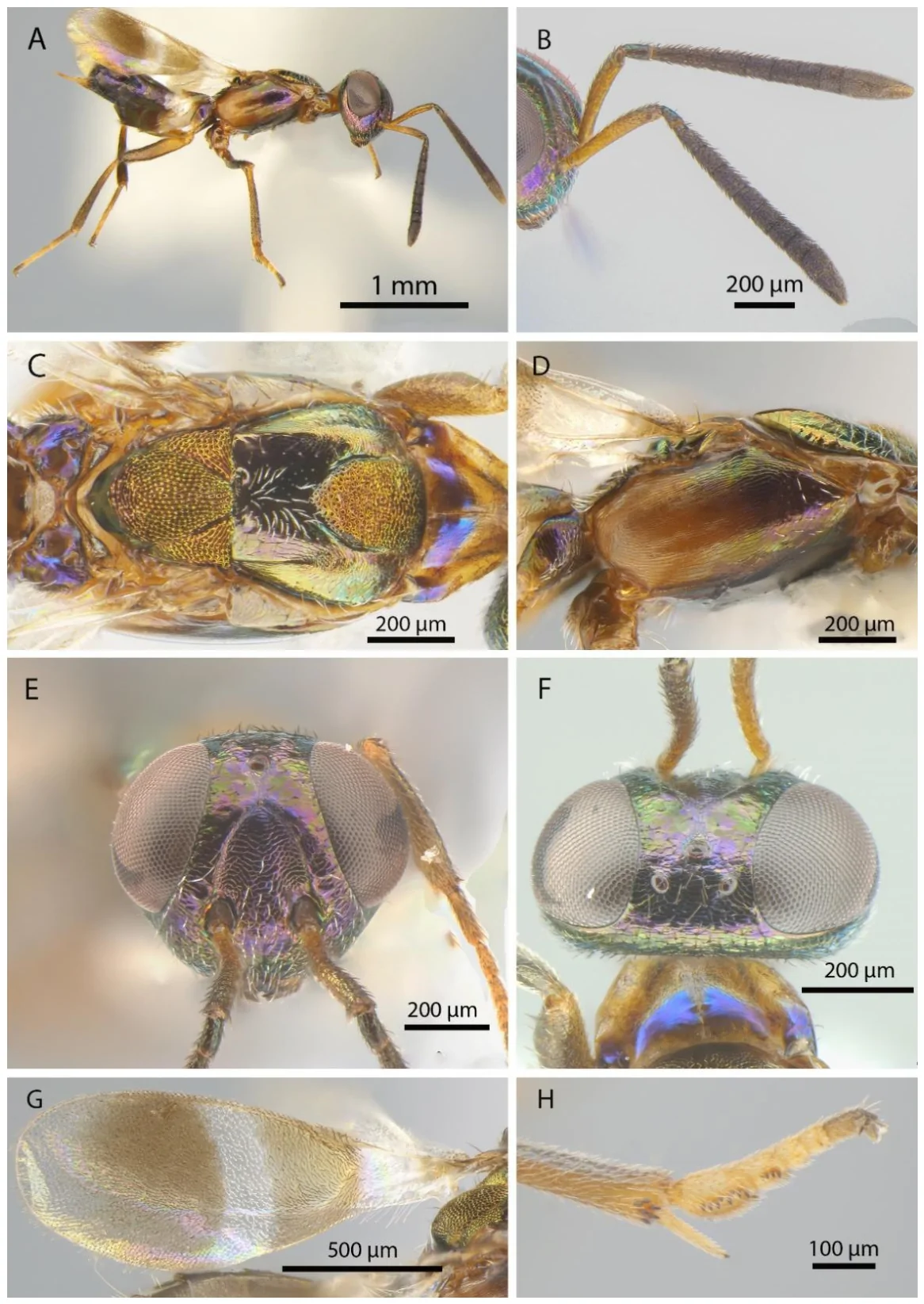
*Anastatus* (*Anastatus*) *beaglei* **sp. nov.** holotype ♀ (FAFU-1457, except for H): (**A**), body lateral; (**B**) antennae; (**C**) mesosoma; (**D**) acropleuron; (**E**) head front; (**F**) head dorsal; (**G**) fore wing; (**H**) mesotibial spur and mesotarsus (FAFU-1245).

**Type material. Holotype.** ♀(FAFU), Wuyishan, Nanping, Fujian Province, China, Malaise trap, 2.XII.2022, FAFU-1457. **Paratypes.** 1♀(FAFU), Wuyishan, Nanping, Fujian Province, China, Malaise trap, 2.XII.2022, FAFU-1245; 1♀(FAFU), Yulinting, Wuyishan, Fujian Province, China, 24.VIII.2022, FAFU-1586.

**Etymology.** The species name *beaglei* refers to HMS Beagle, the Royal Navy ship that carried Charles Darwin on his transformative voyage in the 1830s. The observations made during this journey later contributed to Darwin’s development of the theory of evolution by natural selection.

**Description of holotype. FEMALE.** Body length excluding ovipositor sheaths 2.6 mm (Figure 7A).

**Head.** Mostly bluish purple to green; temple bluish laterally and bluish green medially (Figure 7E,F); vertex slightly bluish to purple with green metallic lustre; frons bluish with green and purple spots; ocellus dark brown in dorsal view; parascrobal region, interantennal prominence, and scrobal depression mostly purple with slightly bluish to purple metallic lustre in frontal view; torulus dark brown; gena bluish purple to green; clypeus greenish blue; labrum and mandible dark brown. Temple coriaceous to alutaceous; frontovertex mesh-like coriaceous to slightly reticulate; parascrobal region coriaceous; interantennal prominence and scrobal depression mesh-like coriaceous to reticulate; scrobal depression shallow and inverted V-shaped. Lower face with lower parascrobal region and interantennal prominence densely setose with white setae; frontovertex and temple with dark brown setae. Antenna (Figure 7B) with scape yellowish brown; pedicel bluish green to dark brown; flagellum violet blue with slight green metallic lustre and densely setose with dark brown setae. Relative length (width) of scape 78.1(13.4), pedicel 16.7(11.6), fl1–fl8: 6.9(10.0), 19.0(11.3), 19.7(12.8), 23.4(14.4), 19.0(14.6), 16.9(17.4), 16.7(17.4), 16.5(18.3), clava 46.7(19.8); OOL:POL:LOL:MPOD = 1.0:3.1:1.9:1.3.

**Mesosoma.** Pronotum mostly yellowish brown to brown posteriorly with blue to brown metallic lustre (Figure 7C); mesoscutal medial lobe with anterior convex region yellowish to reddish brown with slight green metallic lustre; lateral lobes bluish green to purple; posterior depressed region violet blue to purple with green lustre; scutellar–axillar complex yellowish to dark brown with slight bluish green metallic lustre; propodeum dark brown with bluish purple lustre. Pronotum coriaceous to smooth; mesoscutal medial lobe with anterior convex region reticulate to slightly punctate-reticulate; lateral lobes transversely alutaceous to slightly mesh-like reticulate; posterior depressed region coriaceous to reticulate with few scattered white setae; scutellar–axillar complex strongly reticulate. Acropleuron (Figure 7D) darker anteriorly with violet bluish to green metallic lustre, considerably alutaceous throughout to slightly coriaceous right upper anteriorly. Legs (Figure 7A,D,H) dark with mesotarsus yellowish brown; mesocoxa slightly dark; metacoxa dark with violet bluish to green metallic lustre; spur yellowish brown; mesotarsal pegs and apical tarsomere with claws, dark brown. Fore wing (Figure 7G) macropterous, with complete hyaline crossband behind mv; crossband straight with uniform dark setae marginally; costal cell with five lanceolate setae; basal cell with two rows of brown setae along posterior margin. Relative measurements of smv:mv:pmv:stv = 6.2:6.1:3.3:1.0.

**Gaster.** Overall dark brown except Gt1 and Gt2 pale brown to white, sculpture coriaceous to alutaceous (Figure 7A); densely setose with pale brown to white lanceolate setae; ovipositor sheaths yellowish brown and exserted; slightly longer than mesotarsus.

**Distribution.** ORIENTAL: China (Fujian).

**Host.** Unknown.

**Diagnosis.** *A. beaglei* is morphologically close to *A. japonicus*, *A. nozdormu*, and *A. yalini*, but is distinguished by: frons mostly smooth to coriaceous (Figure 7E,F) vs. mostly deeply coriaceous or occasionally reticulate in other three species; hyaline crossband of fore wing broader and straight (Figure 7G) vs. narrower and curved in *A. japonicus* (for figures of *A. japonicus*, see below in the *A. japonicus* section).

#### 3.2.4. *Anastatus* (*Anastatus*) *huijuanae* Khan, Li & Peng, sp. nov. (Figure 8)

Zoobank:urn:lsid:zoobank.org:act:514DED1F-BD78-451A-B1B2-8AA37B4D5572

**Figure 8:**
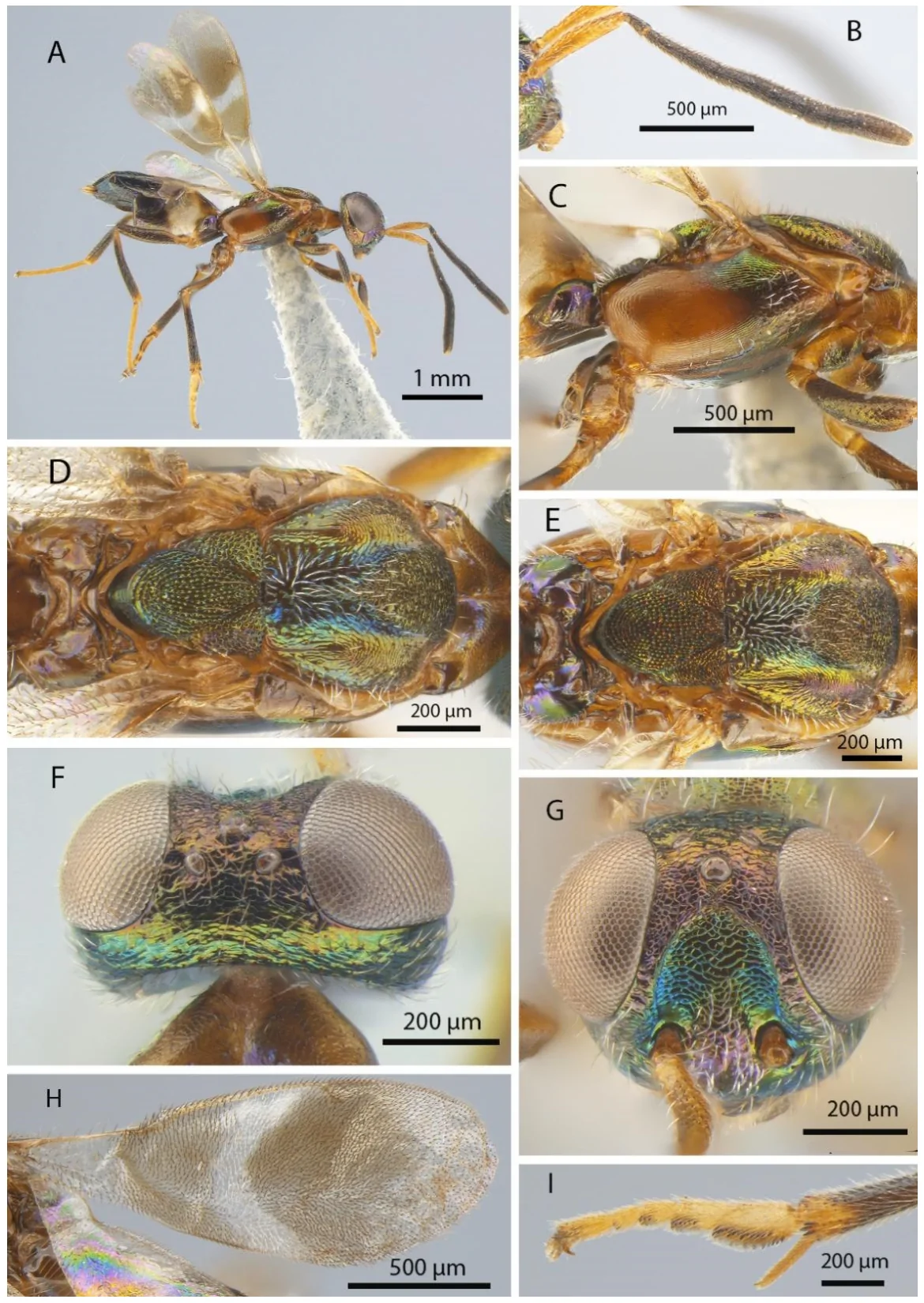
*Anastatus* (*Anastatus*) *huijuanae* **sp. nov.** holotype ♀ (FAFU-1377, except for **A**–**C**,**E**,**I**): (**A**) body lateral (FAFU-1378); (**B**) antenna (FAFU-1378); (**C**) acropleuron (FAFU-1378); (**D**) mesosoma; (**E**) mesosoma (FAFU-1378); (**F**) head dorsal; (**G**) head front; (**H**) fore wing; (**I**) mesotibial spur and mesotarsus (FAFU-1378).

**Type material. Holotype.** ♀(FAFU), Shiti Village, Baoshan, Yunnan Province, China, II.2025, Liu Chuan leg., FAFU-1377. **Paratypes.** 1♀(FAFU), FAFU-1378, same data as holotype; 2♀(FAFU), Lushui, Gulang River (Ganhe Luo), Yunnan Province, China, 1889 m, 6.I.2024, FAFU-1745, FAFU-1757; 1♀(FAFU), Lushui, Gulang River (Ganhe Luo), Yunnan Province, China, 20.IV.2024, FAFU-1755.

**Etymology.** The species is named *huijuanae* in honour of Mrs. Ding Huijuan, the mother of the collector, who accompanied the field collection during a family trip to Yunnan and provided continuous encouragement and support throughout the collection of the type material.

**Description of holotype. FEMALE.** Body length excluding ovipositor sheaths 3.6 mm (Figure 8A).

**Head.** Temple and vertex bluish green with yellowish orange lustre (Figure 8F,G); frons dark, slightly with green to orange lustre; ocellus dark brown; upper parascrobal region violet blue to slightly orange, lower parascrobal region violet blue with green lustre; scrobal depression bluish with slight yellowish green metallic lustre; torulus dark brown; gena bluish green; clypeus bluish; labrum and mandible dark brown. Temple and vertex coriaceous to alutaceous; frons mesh-like reticulate; upper face and lower face strongly reticulate except scrobal depression slightly mesh-like alutaceous, shallow and wide. Vertex and temple with brown setae, interantennal prominence and lower face with dense white setae. Antenna (Figure 8B) dark violet with slight green lustre and densely setose with dark brown setae except scape pale yellowish brown; pedicel violet bluish green with pale brown to white setae. Relative length (width) of scape 97.2(18.0), pedicel 26.4(10.8), fl1–fl8: 10.6(10.0), 28.2(11.2), 30.8(13.2), 34.2(14.6), 24.0(14.8), 25.6(17.6), 21.6(18.6), 20.8(20.0), clava 54.0(20.4); OOL:POL:LOL:MPOD = 1.0:2.6:1.7:1.3.

**Mesosoma.** Mostly dark brown (Figure 8D,E), except pronotum bluish medially with dark spots laterally; mesoscutal medial lobe with anterior convex region, posterior depressed region, and scutellar–axillar complex bright green to bluish metallic lustre; propodeum dark brown. Mesoscutal medial lobe with anterior convex region, and scutellar–axillar complex punctate-reticulate; lateral lobes, and posterior depressed region transversely alutaceous; posterior depressed region densely setose with white setae, whereas, lateral lobes bare or with very few setae. Acropleuron (Figure 8C) dark brown; overall alutaceous, except mesopectus dark green to slightly orange with few white setae and mesh-like reticulate. Legs (Figure 8A,C,I) dark brown; procoxa with green metallic lustre; metacoxa with violet blue to green lustre; tarsus pale brown except mesotarsal pegs dark; spur light brown. Fore wing (Figure 8H) macropterous, costal cell and basal cell with light brown setae; hyaline crossband behind mv densely setose with white setae. Relative measurements of smv:mv:pmv:stv = 6.3:5.5:1.6:1.0.

**Gaster.** Dark brown medially (Figure 8A), except Gt1 and Gt2 white to pale brown with green metallic lustre posteriorly; coriaceous to alutaceous; ovipositor sheaths pale brown; exserted to length of mesotibial apical spur.

**Distribution.** ORIENTAL: China (Yunnan).

**Host.** Unknown.

**Diagnosis.** *A. huijuanae* is morphologically close to *A. napoleonis* but differs in: mesoscutum mostly dark brown with orangish to bluish metallic lustre (Figure 8D,E) vs. mostly greenish in *A. napoleonis* (for figures of *A. napoleonis*, see below in the *A. napoleonis* section); posterior concave lobe of mesoscutum densely setose with white setae (Figure 8D,E) vs. few white setae in *A. napoleonis*; acropleuron and profemur mostly dark brown with green lustre (Figure 8C) vs. acropleuron and profemur pale, except mesopectus with slightly dark greenish to violet spot anterodorsally in *A. napoleonis*. *A. huijuanae* also morphologically similar to *A. tigrinus*; diagnostic characters separating the two species are discussed under diagnosis of *A. tigrinus*.

#### 3.2.5. *Anastatus* (*Anastatus*) *kryptos* Khan, Li & Peng, sp. nov. (Figure 9)

Zoobank:urn:lsid:zoobank.org:act:17DF906A-EC71-43F3-A495-2E4A711B03B9

**Figure 9:**
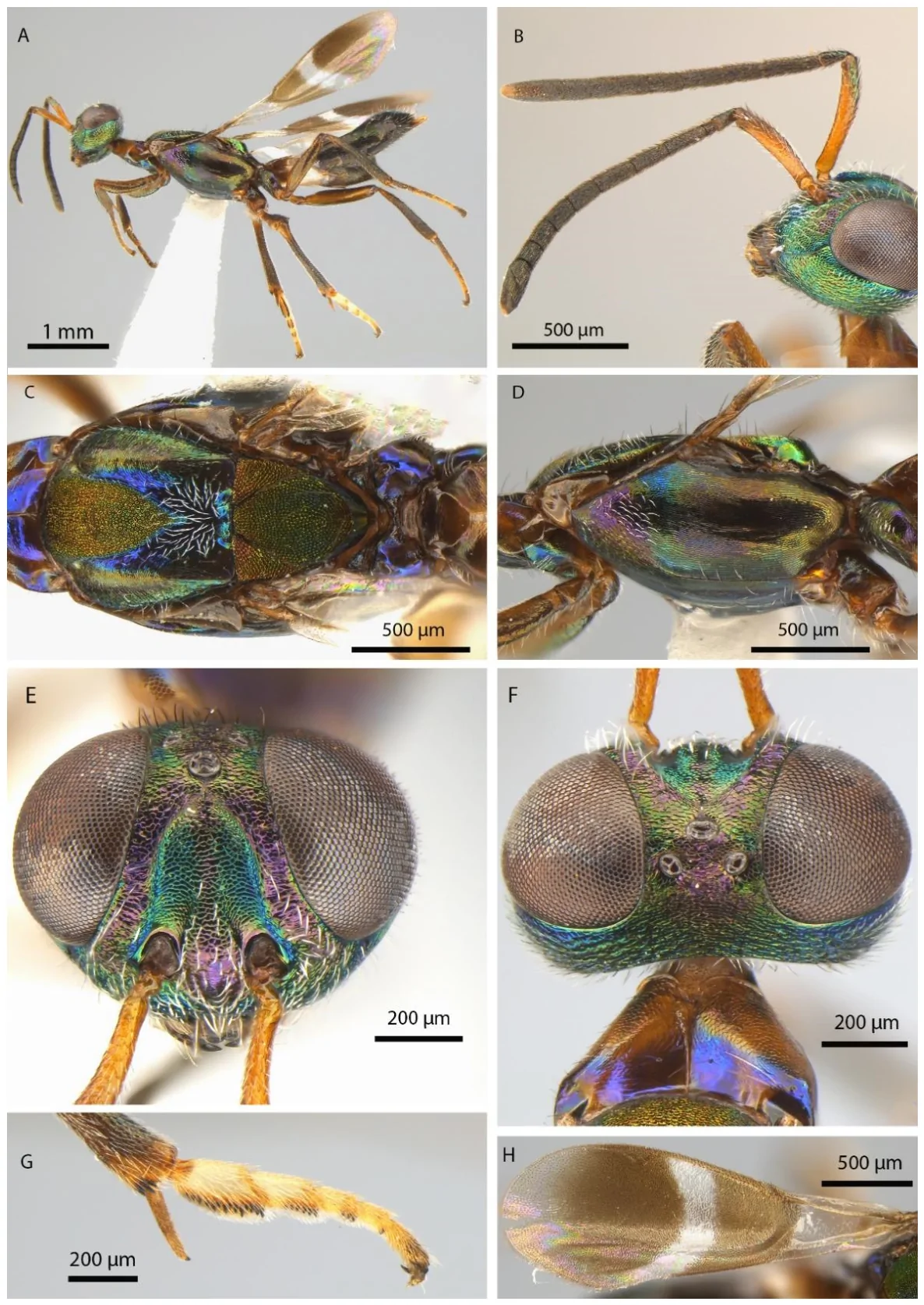
*Anastatus* (*Anastatus*) *kryptos* **sp. nov.** holotype ♀ (FAFU-745): (**A**) body lateral; (**B**) antennae; (**C**) mesosoma; (**D**) acropleuron; (**E**) head front; (**F**) head dorsal; (**G**) mesotibial spur and mesotarsus; (**H**) fore wing.

**Type material. Holotype.** ♀(FAFU), Longqishan Nature Reserve, Sanming, Fujian Province, China, 2018, FAFU-745. **Paratypes.** 1♀(FAFU), Pinggangshan, Minjiangyuan Nature Reserve, Sanming, Fujian Province, China, 30.VI–30.VII.2024, FAFU-1592; 1♀(FAFU), Jiuniutang, Mao’er Mountain, Xing’an County, Guangxi Zhuang Autonomous Region, China, 22.XI.2019, FAFU-696; 1♀(FAFU), Wuyishan, Nanping, Fujian Province, China, 1.V.2024, Jin Yancai leg., FAFU-1137; 1♀(FAFU), Nanling National Reserve, Shaoguan, Guangdong Province, China, 21.VI–6.VII.2020, FAFU-1411; 1♀(FAFU), Dishuihe, Lushui, Yunnan Province, China, 1761 m, 12.I.2024, FAFU-1747; 1♀(FAFU), Gulang River, Lushui, Yunnan Province, China, 1884 m, 13.XII.2023, FAFU-1751; 1♀(FAFU), Pinggangshang, Minjiangyuan Nature Reserve, Sanming City, Fujian Province, China, 31.V.2024, FAFU-1396.

**Non-type material.** All the non-type materials were collected from the same locality on various dates: Shennongjia Forest District, Hubei Province, China, Malaise trap, 1♀(FAFU), 14–31.VII.2022, Liu Meike leg., FAFU-1508; 1♀(FAFU), 31.VII–20.VIII.2022, Liu Meike leg., FAFU-1502; 1♀(FAFU), 20.VII.2022, Liu Meike leg., FAFU-1503; 1♀(FAFU), 21.VII.2023, Che Jinting leg., FAFU-1610; 1♀(FAFU), 30.VII.2023, Che Jinting leg., FAFU-1615; 1♀(FAFU), 27.VII.2023, Che Jinting leg., FAFU-1632; 2♀(FAFU), 13.VII.2023, Che Jinting leg., FAFU-1616, 1617; 1♀(FAFU), 12.VIII.2023, Liu Meike leg., FAFU-1609; 1♀, 14.VII.2022, Liu Meike leg., FAFU-1614; 1♀(FAFU), 11.IX.2023, Liu Meike leg., FAFU-1618; 3♀(FAFU), 14.IX.2023, Liu Meike leg., FAFU-1608, 1623, 1624; 4♀(FAFU), 15.X.2023, Liu Meike leg., FAFU-1626, 1629, 1630, 1631.

**Etymology.** *kryptos* is derived from the Greek word *kryptós*, meaning “hidden” or “concealed,” referring to its cryptic appearance, which makes it difficult to distinguish from *A. gansuensis*.

**Description of holotype. FEMALE.** Body length excluding ovipositor sheaths 4.1 mm (Figure 9A).

**Head.** Temple (Figure 9E,F) with bluish green metallic lustre; vertex violet blue with green lustre; frons mostly green to violet blue, ocellus dark brown; in frontal view, parascrobal region violet blue; lower parascrobal region with slight green metallic lustre; scrobal depression blue with green lustre; torulus dark brown, but base purple to green; interantennal prominence bluish purple with green lustre; gena greenish blue with slight yellowish lustre; clypeus blue; labrum and mandible dark brownish to blue. Temple and vertex mesh-like reticulate to slightly alutaceous; frons slightly mesh-like reticulate; scrobal depression shallow, inverted V-shaped; interantennal prominence and parascrobal region reticulate with slightly imbricate alutations; lower face coriaceous to reticulate, densely setose with white setae. Antenna (Figure 9B) with scape pale brown, but dark greenish blue posteriorly; scape dark bluish to green; flagellum violet blue to slightly green, with pale to dark brown setae; posterior end of clava pale brown. Relative length (width) of scape 85.3(14.8), pedicel 23.6(9.5), fl1–fl8: 10.3(10.0), 31.4(11.2), 27.2(12.6), 30.3(11.9), 22.8(13.6), 22.2(14.8), 20.7(15.9), 18.6(15.7), clava 46.4(15.5); OOL: POL: LOL: MPOD = 1.0: 2.7: 1.4: 1.7.

**Mesosoma.** Overall dark, except pronotum sometimes slightly light brown with strong bluish metallic lustre (Figure 9C); mesoscutal medial lobe with anterior convex region, and scutellar–axillar complex with violet bluish to green metallic lustre; lateral lobes, and posterior depressed region dark blue to green with purple lustre; propodeum dark brown with bluish lustre. Pronotum coriaceous to alutaceous; mesoscutal medial lobe with anterior convex region, and scutellar–axillar complex slightly punctate to strongly reticulate; lateral lobes transversely alutaceous with few setae marginally; posterior depressed region mesh-like reticulate to alutaceous with dense white setae. Acropleuron (Figure 9D) dark with purple to green metallic lustre with uniform white setae anteriorly; slightly reticulate to alutaceous. Legs (Figure 9A,D,G) dark brown, except pro- and metacoxa with greenish blue to purple metallic lustre; mesotarsus pale brown to white except tibial spur, mesotarsal pegs and tibial claws dark. Fore wing (Figure 9H) macropterous, covered with dark setae except hyaline crossband behind mv densely setose with white setae; basal cell with few white setae; costal cell with six to seven lanceolate setae. Relative measurements of cc:smv:mv:pmv:stv = 6.9:4.3:6.1:2.8:1.0.

**Gaster.** Overall dark brown except apex of syntergum pale. Gt1 and Gt2 pale brown to white, with dense white setae posteriorly with coriaceous to alutaceous (Figure 9A); gaster longer than mesosoma; ovipositor sheaths yellowish-brown, exserted, with few white setae, and as long as mesotibial apical spur.

**Distribution.** ORIENTAL: China (Guangxi, Fujian, Guangdong, Yunnan).

**Host.** Unknown.

**Diagnosis.** *A. kryptos* morphologically similar to *A. gansuensis*, but differs in: posterior concave lobe of mesoscutum densely setose with white setae (Figure 9C) vs. sparsely setose, white setae restricted medially in *A. gansuensis* (for figures of *A. gansuensis*, see below in the *A. gansuensis* section); fore wing with hyaline crossband broad and complete (Figure 9H) vs. crossband narrower, sometimes constricted or appearing as two spots in *A. gansuensis*.

*A. kryptos* is also morphologically close to *A. catalonicus* Bolívar y Pieltain, 1935 [32], but differs in: fore wing hyaline crossband broad (Figure 9H) vs. constricted medially in *A. catalonicus*; gaster slender, longer than mesosoma (Figure 9A) vs. shorter than mesosoma in *A. catalonicus* [32,33].

**Figure 10:**
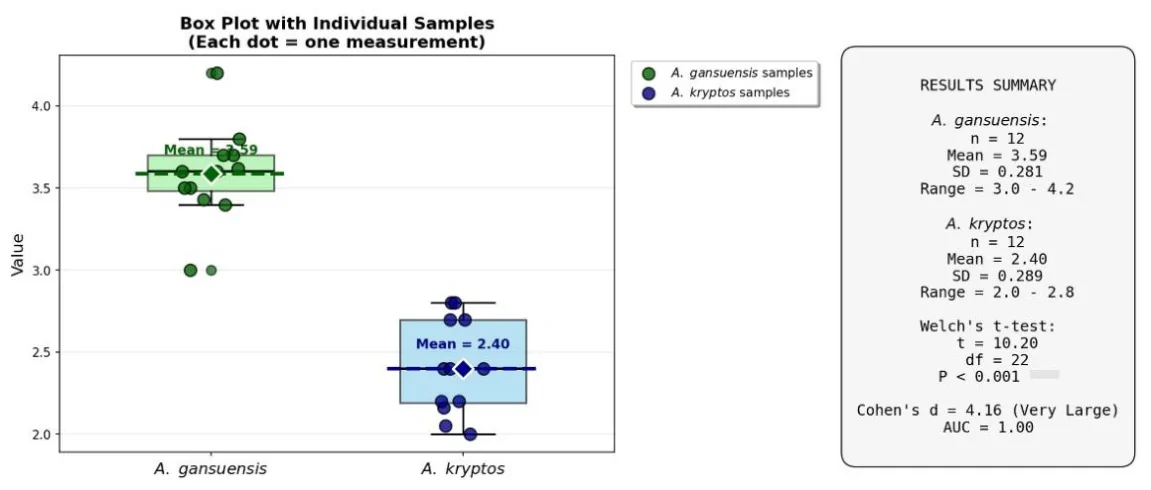
Morphometric variations of the fore wing hyaline crossband between *A. gansuensis* and *A. kryptos*. Data represent the ratio of the infuscate region width (posterior to parastigma) relative to the hyaline crossband width. Box plot shows the median, interquartile range (IQR), and full range of ratios, highlighting the diagnostic gap between species; horizontal dashed lines indicate quartiles and medians. (n = 12 for *A. gansuensis*; n = 12 for *A. kryptos*).

**Remarks.** The fore wing hyaline crossband ratio (infuscate region width/hyaline crossband width) is significantly higher in *A. gansuensis* (n = 12, $\bar{x}$ = 3.6; range 3.0–4.2×) than in *A. kryptos* (n = 12, $\bar{x}$ = 2.4; range 2.0–2.8×) (Welch’s *t*-test, *t* = 10.2, df = 22, *p* < 0.001; Cohen’s d = 4.2, AUC = 1.0). This higher ratio reflects a constricted or incomplete hyaline crossband in *A. gansuensis*, which is often interrupted medially by dense dark setae or appearing as two spots. In contrast, *A. kryptos* exhibited a complete hyaline crossband of uniform width across the fore wing disc (Figure 9H). The two species showed no overlap in this ratio, which serves as a reliable diagnostic morphometric character to distinguish these two species (Figure 10).

#### 3.2.6. *Anastatus* (*Anastatus*) *napoleonis* Khan, Li & Peng, sp. nov. (Figure 11)

Zoobank:urn:lsid:zoobank.org:act:3E8994FB-C695-462C-865E-EC16245A54A3

**Figure 11:**
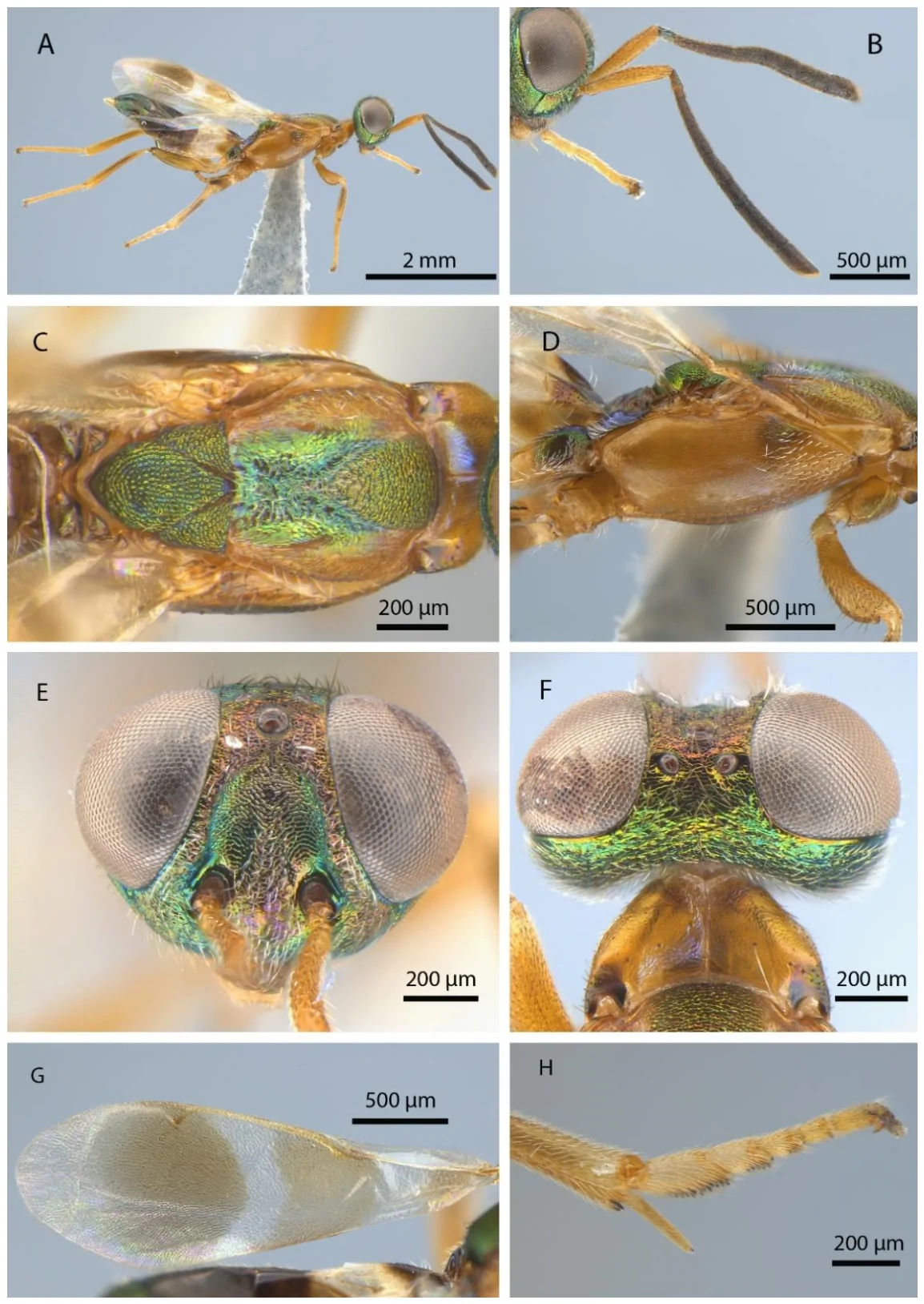
*Anastatus* (*Anastatus*) *napoleonis* **sp. nov.** holotype ♀ (FAFU-1361, except for **C**): (**A**) body lateral; (**B**) antennae; (**C**) mesosoma (FAFU-845); (**D**) acropleuron; (**E**) head front; (**F**) head dorsal; (**G**) fore wing; (**H**) mesotibial spur and mesotarsus.

**Type material. Holotype.** ♀(FAFU), Nanling National Nature Reserve, Shaoguan, Guangdong Province, China, Malaise trap, 9–21.VI.2020, FAFU-1361. **Paratypes.** 1♀(FAFU), Nanling National Nature Reserve, Shaoguan, Guangdong Province, China, Malaise trap, 28.X.2021–6.I.2022, FAFU-1587; 1♀(FAFU), Nanling National Nature Reserve, Shaoguan, Guangdong Province, China, Malaise trap, 19.X.2021–11.I.2022, FAFU-1588; 1♀(FAFU), Nanling Nature Reserve, Ruyuan Yao Autonomous County, Shaoguan, Guangdong Province, China, 23.V.2020, FAFU-1184; 1♀(FAFU), Nanling National Nature Reserve, Ruyuan Yao Autonomous County, Shaoguan, Guangdong Province, China, Malaise trap, 9.IV–28.V.2021, FAFU-1215; 1♀(FAFU), Nanling National Nature Reserve, Ruyuan Yao Autonomous County, Shaoguan, Guangdong Province, China, 28.X.2020, FAFU-1224.

**Non-type material.** 2♀(FAFU), Minglong Cave, Emei Mount, Leshan, Sichuan Province, China, 4.VI.2019, FAFU-681, 682; 1♀(FAFU), Goudunping, Tianbaoyan Nature Reserve, Yong’an City, Sanming, Fujian Province, China, 14.X.2020, FAFU-845; 1♀(FAFU), Shengtangshan, Dayao Mountain, Guangxi Zhuang Autonomous Region, China, 10.VII.2020, Li Tao leg., FAFU-1569; 1♀(FAFU), Tongmuguan, Wuyishan, Nanping, Fujian Province, China, 6.XI.2021, FAFU-1572; 1♀(FAFU), Da’anyuan, Wuyishan, Nanping, Fujian Province, China, Malaise trap, 08.X.2022, FAFU-1584; 1♀(FAFU), Nanling National Nature Reserve, Ruyuan Yao Autonomous County, Shaoguan, Guangdong Province, China, Malaise trap, 28.X–25.XI.2020, FAFU-1234; 2♀(FAFU), Nanling National Nature Reserve, Shaoguan, Guangdong Province, China, Malaise trap, 28.X–25.XI.2020, FAFU-1514, 1515.

**Etymology.** The specific name *napoleonis* is a patronym formed in the Latin genitive case from the Latinized name Napoleon, referring to Napoleon Bonaparte, who was crowned Emperor of France on 2 December 1804.

**Description of holotype. FEMALE.** Body length excluding ovipositor sheaths 4.1 mm (Figure 11A).

**Head.** Temple and vertex bluish green with yellow lustre (Figure 11E,F); frons dark violet to yellow; ocellus dark brown; upper parascrobal region yellowish purple; lower parascrobal region bluish purple; torulus margin blue with slight green metallic lustre while torulus base dark brown; gena bluish purple with green lustre; clypeus bluish green; labrum and mandible dark brown. Temple and vertex reticulate to alutaceous; frons deeply reticulate; upper face and lower face strongly reticulate except scrobal depression slightly alutaceous at base, scrobal depression shallow and wide; vertex, temple, parascrobal region, interantennal prominence and lower face with dense white setae. Antenna (Figure 11B) dark violet with dark brown setae, except scape pale yellowish brown; pedicel dark blue with dense white setae. Relative length (width) of scape 93.4(18.0), pedicel 22.0(11.4), fl1–fl8: 10.1(10.0), 35.0(10.9), 33.8(14.6), 36.0(16.3), 29.1(17.0), 25.6(19.2), 21.6(19.6), 18.9(19.6), clava 50.7(20.9); OOL:POL:LOL:MPOD = 1.0:2.9:1.8:1.4.

**Mesosoma.** Overall pale brown, except pronotum medially with bluish lustre and dark spots laterally (Figure 11C); mesoscutal medial lobe with posterior depressed region, scutellar–axillar complex bright green to blue metallic lustre; propodeum dark brown. Mesoscutal medial lobe with anterior convex region, and scutellar–axillar complex punctate-reticulate; lateral lobes transversely alutaceous with few setae; posterior depressed region mesh-like reticulate. Acropleuron (Figure 11D) yellowish to pale brown, except mesopectus with slightly green lustre; overall alutaceous, anteriorly with uniform white setae. Legs (Figure 11A,D,H) pale brown, except procoxa with light green lustre; mesofemur and mesotibia dark posteriorly; metacoxa dark green with few white setae; metafemur and metatibia with dark lustre; spur light brown. Fore wing (Figure 11G) macropterous; costal cell and basal cell hyaline with light brown setae; hyaline crossband behind mv. Relative measurements of smv:mv:pmv:stv = 7.2:6.6:2.6:1.0.

**Gaster.** Dark brown medially, Gt1 and Gt2 white to pale brown, posterior terga with green metallic lustre (Figure 11A); sculpture coriaceous to reticulate, alutaceous dorsally; ovipositor sheaths pale brown; exserted, and as long as last segment of mesotarsus.

**Distribution.** ORIENTAL: China (Guangdong, Fujian, Guangxi, Sichuan).

**Host.** Unknown.

**Diagnosis.** *A. napoleonis* is morphologically similar to *A. tigrinus*, but differs in: gaster lacks transverse bands (Figure 11A) vs. transverse bands present in *A. tigrinus* (for figures of *A. tigrinus*, see below in the *A. tigrinus* section); fore wing with complete hyaline crossband (Figure 11G) vs. crossband constricted medially in *A. tigrinus*. *A. napoleonis* is also morphologically close to *A. huijuanae*; see the diagnosis under *A. huijuanae* for detailed diagnostic characters.

#### 3.2.7. Anastatus (Anastatus) neltharion Khan, Li & Peng, sp. nov. (Figure 12)

Zoobank:urn:lsid:zoobank.org:act:F4380497-982A-499B-851D-139B4DA8E0AB

**Figure 12:**
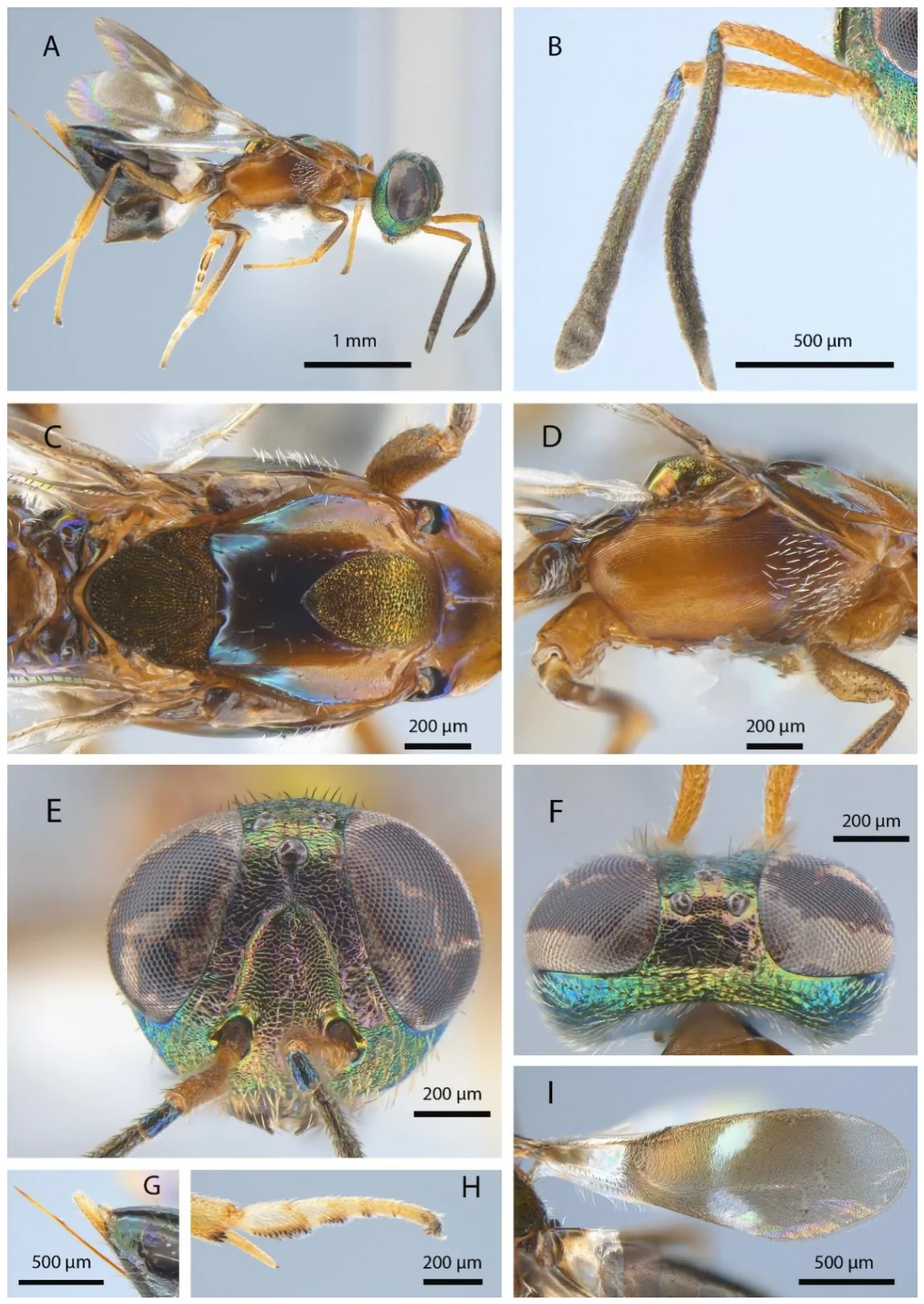
*Anastatus* (*Anastatus*) *neltharion* **sp. nov.** holotype ♀ (FAFU-1294): (**A**) body lateral; (**B**) antennae; (**C**) mesosoma; (**D**) acropleuron; (**E**) head front; (**F**) head dorsal; (**G**) ovipositor; (**H**) mesotibial spur and mesotarsus; (**I**) fore wing.

**Type material. Holotype.** ♀(FAFU), Dong Island, Sansha, Hainan Province, China, 10.I.2024, Shi Zhiyuan leg., FAFU-1294. **Paratypes.** 1♀(FAFU), Dong Island, Sansha, Hainan Province, China, 13.I.2024, Chen Huayan leg., FAFU-1255; 1♀(FAFU), Dong Island, Sansha, Hainan Province, China, 13.I.2024, Chen Huayan leg., FAFU-1256; 1♀(FAFU), Fuzhou, Fujian Province, China, 9.X.2024, Ni Yuanzhou leg., FAFU-1293.

**Etymology.** The species epithet *neltharion* is a noun in apposition derived from Neltharion (Deathwing), the lord of the black dragonflight in World of Warcraft. The name refers to the dark posterior lobe of the mesoscutum, which resembles the heavy dark plating of the dragon’s back, and the fiery metallic lustre of the lateral lobes, which resembles molten magma glowing through dark volcanic scales.

**Description of holotype. FEMALE.** Body length excluding ovipositor sheaths 3.6 mm (Figure 12A).

**Head.** Mostly bluish violet, except temple bluish green laterally; vertex greenish purple; frons bluish violet with green lustre (Figure 12E,F); ocellus dark brown in dorsal view; parascrobal region and interantennal prominence bluish purple ventrally with slight green metallic lustre; scrobal depression bluish violet with slight yellowish lustre; torulus dark brown; gena bluish green; clypeus bluish; labrum and mandible dark brown. Temple and vertex coriaceous to alutaceous; frons coriaceous to reticulate; upper face and lower face mostly coriaceous to reticulate with dense light brown setae; scrobal depression slightly alutaceous. Antenna (Figure 12B) dark bluish violet with dense brown setae, except scape pale brown; pedicel dark blue with slight greenish lustre. Relative length (width) of scape 95.0(13.8), pedicel 17.0(10.6), fl1–fl8: 7.5(10.0), 26.3(10.4), 28.3(12.0), 31.8(13.8), 24.6(15.2), 22.1(15.4), 20.6(16.0), 18.1(15.8), clava 41.7(25.4); OOL:POL:LOL:MPOD = 1.0:4.4:2.6:3.2.

**Mesosoma.** Mostly dark brown, except pronotum laterally with dark bluish spots and medially with shaded blue region (Figure 12C); mesoscutal medial lobe with anterior convex region, and scutellar–axillar complex light brown with green lustre; lateral lobes light brown with slight blue metallic lustre; posterior depressed region uniformly dark brown with slight blue metallic lustre marginally. Mesoscutal medial lobe with anterior convex region, and scutellar–axillar complex strongly reticulate; lateral lobes coriaceous to slightly alutaceous; posterior depressed region smooth with only few scattered setae. Acropleuron (Figure 12D) pale yellow to brown, except mesopectus bluish green metallic lustre with uniform white setae anteriorly. Legs (Figure 12A,D,H) pale brown, except pro- and mesofemur, protibia, anterior side of mesotibia, metacoxa, parts of metafemur and metatibia dark brown; spur light brown; two rows of mesotarsal pegs dark brown; entire tarsus pale brown to white. Fore wing (Figure 12I) macropterous, covered with dark to light brown setae, with two hyaline spots behind mv; costal cell and basal cell entirely bare; parastigma of smv with dense dark setae; smv with seven lanceolate setae. Relative measurements of smv:mv:pmv:stv = 6.1:5.0:2.9:1.0.

**Gaster.** Dark brown, except Gt1, Gt2, and partly Gt3 pale brown to white with dense pale brown setae dorsally and laterally (Figure 12A); ovipositor sheaths (Figure 12G) exserted; pale with dense light brown setae.

**Distribution.** ORIENTAL: China (Hainan, Fujian).

**Host.** Unknown.

**Diagnosis.** *A. neltharion* morphologically similar to *A. echidna*, but differs in: posterior concave lobe of mesoscutum with few dark brown setae (Figure 12C) vs. dense white setae in *A. echidna* (for figures of *A. echidna*, see below in the *A. echidna* section); anterior convex lobe of mesoscutum with apex rounded (Figure 12C) vs. apex sharply V-shaped in *A. echidna*; dark setae between fore wing hyaline spots more dispersed (Figure 12I) vs. less dispersed in *A. echidna*; ovipositor sheaths comparatively longer (Figure 12G) vs. shorter in *A. echidna*.

#### 3.2.8. Anastatus (Anastatus) nozdormu Khan, Li & Peng, sp. nov. (Figure 13)

Zoobank:urn:lsid:zoobank.org:act:27B3AFB2-928D-4A38-91BB-8F9E5766ADE6

**Figure 13:**
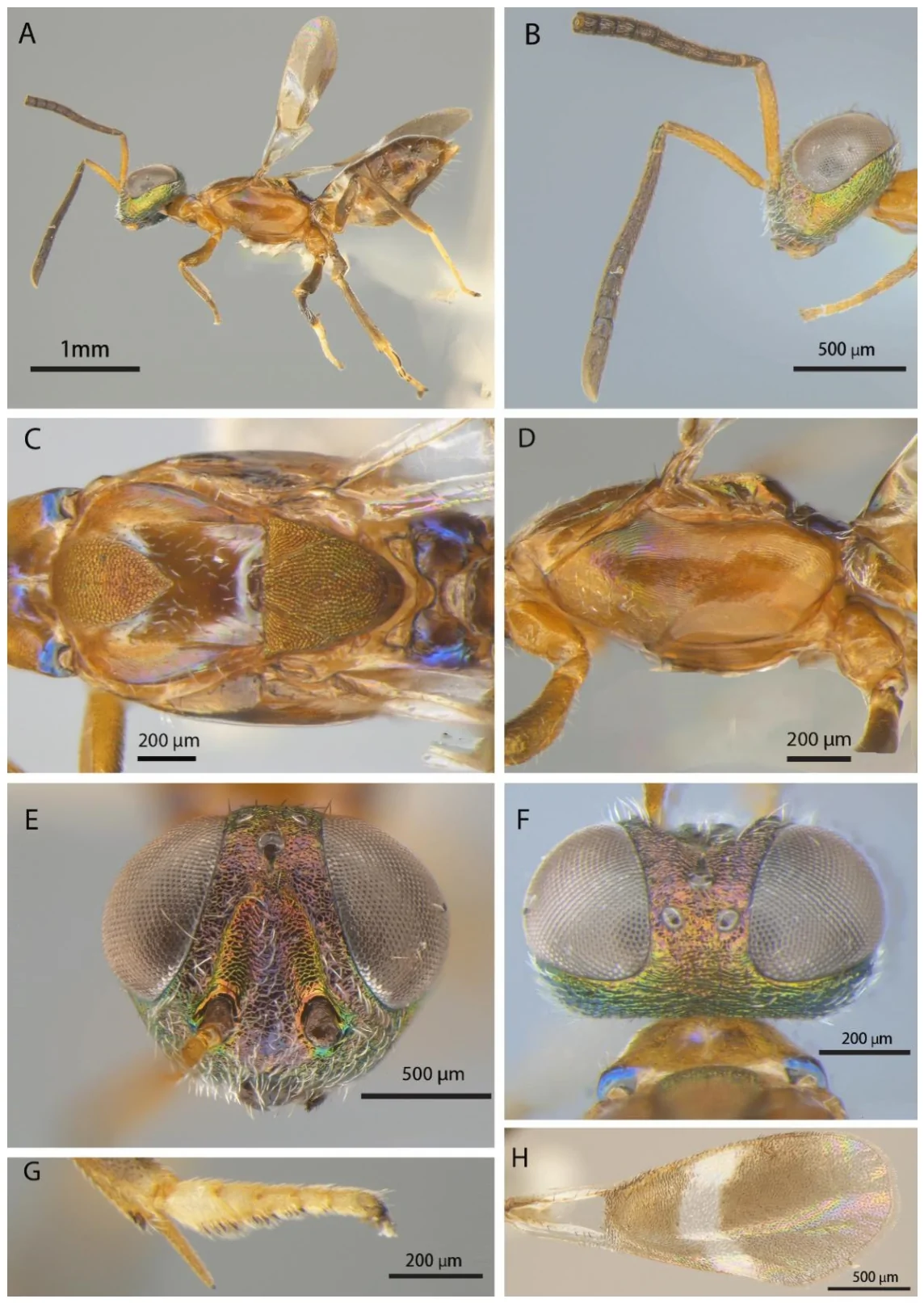
*Anastatus* (*Anastatus*) *nozdormu* **sp. nov.** holotype ♀ (FAFU-1223, except for **H**): (**A**) body lateral; (**B**) antennae; (**C**) mesosoma; (**D**) acropleuron; (**E**) head front; (**F**) head dorsal; (**G**) mesotibial spur and mesotarsus; (**H**) fore wing (FAFU-1385).

**Type material. Holotype.** ♀(FAFU), Pattle Island, Sansha, Hainan Province, China, 9.I.2024, Chen Huayan leg., FAFU-1223. **Paratypes.** 2♀(FAFU), same data as holotype, FAFU-1222, 1278; 2♀(FAFU), Robert Island, Sansha, Hainan Province, China, 9.I.2024, FAFU-1266, 1220; 1♀(FAFU), Haizhu Wetland, Guangzhou, Guangdong Province, China, 14.X–14.XI.2021, FAFU-1030.

**Non-type material.** 3♀(FAFU), Haizhu Wetland, Guangzhou, Guangdong Province, China, 10.IX–10.X.2021, FAFU-1021, 1022, 1024; 1♀(FAFU), Haizhu Wetland, Guangzhou, Guangdong Province, China, 29.V–10.VI.2021, FAFU-995; 1♀(FAFU), Futian Mangrove Ecological Park, Shenzhen, Guangdong Province, China, IX–X.2020, Li Kunyuan leg., FAFU-1354; 1♀(FAFU), Dadong Mountain, Qingyuan, Guangdong Province, China, 24.V–6.VI.2021, FAFU-1601; 1♀(FAFU), Xili Village, Guanqiao Town, Nan’an County, Quanzhou, Fujian Province, China, 29.VII.2025, Wang Qiyan leg., FAFU-1464; 1♀(FAFU), Xili Village, Guanqiao Town, Nan’an County, Quanzhou, Fujian Province, China, 29.IX.2025, Wang Qiyan leg., FAFU-1384; 1♀(FAFU), Woody Island, Sansha, Hainan Province, China, 6.I.2024, FAFU-1171; 1♀(FAFU), Woody Island, Sansha, Hainan Province, China, 18.VIII.2019, FAFU-1221; 2♀(FAFU), Money Island, Sansha, Hainan Province, China, 8.I.2024, FAFU-1305, 1307; 1♀(FAFU), Chenhang Island, Sansha, Hainan Province, China, 10.I.2024, FAFU-1385; 1♀(FAFU), Sansha East Island, Hainan Province, China, 10.I.2024, Shi Zhiyuan leg., FAFU-1236; 2♀(FAFU), Experimental Station of Research Institute of Tropical Forestry, Chinese Academy of Forestry, Jianfeng, Ledong Li Autonomous County, Hainan Province, China, 30.X–30.XI.2019, Xu Chunyang leg., FAFU-1578, 1301.

**Etymology.** The species epithet *nozdormu* is a noun in apposition derived from Nozdormu, the lord of the bronze dragonflight who commands time and the timeways in World of Warcraft, in reference to the bronze-amber body colour of this species.

**Description of holotype. FEMALE.** Body length excluding ovipositor sheaths 3 mm (Figure 13A).

**Head.** Temple and vertex green, while temple slightly bluish laterally; frons bluish purple to green metallic lustre (Figure 13E,F); ocellus pale grey in dorsal view; parascrobal region and interantennal prominence dark blue to purple in frontal view; scrobal depression blue to dark violet with slightly yellowish margin; torulus bluish purple at upper margin, while lower margin greenish blue; gena bluish purple with green lustre; clypeus dark bluish purple; labrum dark brown. Temple and vertex reticulate to alutaceous; frons reticulate; upper face and lower face strongly reticulate; lower parascrobal region, interantennal prominence, and lower face densely setose with white setae. Antenna (Figure 13B) dark blue to purple with dense light brown to white setae, except pale yellowish brown scape with white setae. Relative length (width) of scape 90.3(11.9), pedicel 20.9(8.6), fl1–fl8: 8.4(10.0), 23.0(10.3), 25.5(11.7), 26.3(13.8), 19.4(14.8), 19.0(17.3), 19.2(18.0), 16.7(18.0), clava 52.1(18.0); OOL:POL:LOL:MPOD = 1.0:3.2:2.4:2.3.

**Mesosoma.** Overall pale brown except pronotum with slight blue metallic lustre medially, and bluish spots laterally (Figure 13C); mesoscutal medial lobe with anterior convex region, and scutellar–axillar complex brown with green lustre; posterior depressed region dark brown; propodeum purple to dark brown. Mesoscutal medial lobe with anterior convex region, and scutellar–axillar complex strongly reticulate; lateral lobes, and posterior depressed region smooth with green lustre and few scattered white setae; propodeal plical depression broad. Acropleuron (Figure 13D) overall pale yellow except mesopectus violet bluish to green and mostly alutaceous. Legs (Figure 13A,D) pale brown, except mesofemur, metafemur, mesotibia, metatibia, and metacoxa dark brown; spur light brown (Figure 13G). Fore wing (Figure 13H) macropterous, covered with dark brown setae except basal cell bare; broad hyaline crossband with white setae; costal cell bare; smv with six to seven lanceolate setae. Relative measurements of smv:mv:pmv:stv = 6.9:5.8:3.0:1.0.

**Gaster.** Overall dark brown except Gt1 and Gt2 pale brown to white, completely coriaceous to reticulate with densely setose and lanceolate posterior to gaster (Figure 13A); ovipositor sheaths light brow, exserted, and slightly shorter than mesotibial apical spur.

**Distribution.** ORIENTAL: China (Guangdong, Fujian, Hainan).

**Host.** Unknown.

**Diagnosis.** *A. nozdormu* is morphologically close to *A. japonicus*, but differs in: basal cell of fore wing bare (Figure 13H) vs. setose in *A. japonicus* (for figures of *A. japonicus*, see below in the *A. japonicus* section); posterior lobe of mesoscutum with few scattered white setae (Figure 13C) vs. densely setose in *A. japonicus*; mesoscutal sculpture weaker (Figure 13C) vs. stronger in *A. japonicus*. *A. nozdormu* also similar to *A. yalini*, but differs in: posterior lobe of mesoscutum pale brown, setae evenly scattered throughout (Figure 13C) vs. dark greenish purple to violet, few setae medially in *A. yalini* (for figures of *A. yalini*, see below in the *A. yalini* section); propodeal callus broader (Figure 13C) vs. narrower in *A. yalini*.

#### 3.2.9. Anastatus (Anastatus) tigrinus Khan, Li & Peng, sp. nov. (Figure 14)

Zoobank:urn:lsid:zoobank.org:act:93375E86-9A4D-4B82-B6C7-C83101FA0ECB

**Figure 14:**
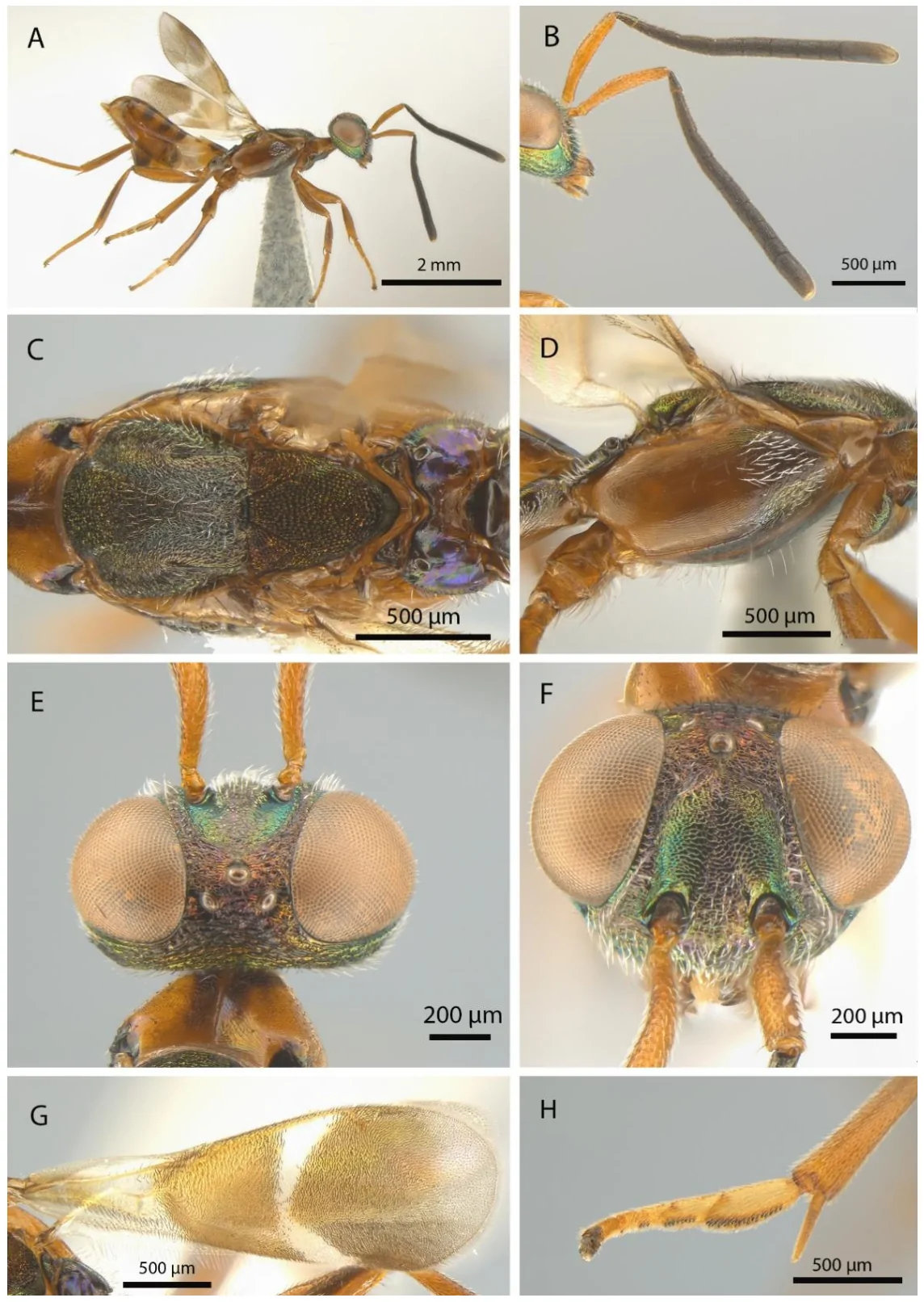
*Anastatus* (*Anastatus*) *tigrinus* **sp. nov.** holotype ♀ (FAFU-1501, except for **G**,**H**): (**A**) body lateral; (**B**) antennae; (**C**) mesosoma; (**D**) acropleuron; (**E**) head dorsal; (**F**) head front; (**G**) fore wing (FAFU-1504); (**H**) mesotibial spur and mesotarsus (FAFU-1504).

**Type material. Holotype.** ♀(FAFU), Shennongjia Forest District, Hubei Province, China, 14.IX.2023, Liu Meike leg., FAFU-1501. **Paratypes.** same locality with holotype, 1♀(FAFU), 10.IX.2022, FAFU-1504; 1♀(FAFU), 23.X.2022, FAFU-1506; 2♀(FAFU), 24.VIII.2023, FAFU-1603, 1622; 1♀(FAFU), 19–20.VIII.2022, FAFU-1627.

**Etymology.** The species name *tigrinus* is derived from Latin, meaning “tiger-like,” and refers to the conspicuous transverse banding pattern on the gaster, which resembles the characteristic stripes of a tiger.

**Description of holotype. FEMALE.** Body length excluding ovipositor sheaths 4.5 mm (Figure 14A).

**Head.** Temple dark brown to green with bluish metallic lustre in dorsal view (Figure 14E,F); vertex mostly dark brown with slight green lustre; frons dark brown with yellowish brown to green lustre; ocellus dark brown; parascrobal region and interantennal prominence dark brown to violet blue, except lower parascrobal region with green lustre in frontal view; scrobal depression bluish green to purple metallic lustre; torulus dark brown; gena yellowish to bluish green; clypeus violet blue to green; labrum and mandible dark brown. Temple and vertex reticulate to alutaceous; frons mesh-like reticulate; parascrobal region reticulate; scrobal depression shallow with interantennal prominence mesh-like reticulate to slightly alutaceous; lower face including lower parascrobal region and interantennal prominence densely setose with white setae. Antenna (Figure 14B) with scape orangish brown; pedicel bluish green to purple; flagellomeres dark violet blue with dense dark brown setae. Relative length (width) of scape 94.8(14.7), pedicel 20.3(9.3), fl1–fl8: 10.6(10.0), 31.3(12.4), 29.4(16.3), 31.0(18.0), 25.1(18.8), 23.2(19.0), 20.5(19.4), 16.8(19.7), clava 51.9(21.0); OOL:POL:LOL:MPOD = 1.0:2.6:1.4:1.6.

**Mesosoma.** Overall dark, except pronotum yellowish brown with dark violet bluish to green spots laterally and slightly brown lustre medially (Figure 14C); mesonotum dark with violet bluish to green, whereas scutellar–axillar complex bright bluish green to orangish purple metallic lustre; propodeum dark brown with violet blue to green metallic lustre. Pronotum coriaceous to alutaceous; mesoscutal medial lobe with anterior convex region reticulate anteriorly and punctate-reticulate posteriorly but indistinct; posterior depressed region coriaceous to reticulate with few white setae, whereas considerably distinct; lateral lobes of mesoscutum reticulate to slightly longitudinally alutaceous with few white setae; scutellar–axillar complex strongly reticulate. Acropleuron (Figure 14D) orangish to pale brown with slight bluish green lustre and dense uniform white setae anteriorly, whereas alutaceous to slightly coriaceous. Legs (Figure 14A,D,H) overall pale brown except procoxa and metacoxa purple to green with white setae, except mesocoxa with slight green lustre; tibia with dark brown infuscations; mesotarsus pale brown, except mesotarsal pegs and apical tarsomere bearing dark brown claws. Fore wing (Figure 14G) macropterous with complete hyaline crossband, interrupted medially with dark setae; costal cell with nine to ten lanceolate setae; parastigma of smv densely setose with dark setae. Relative measurements of smv:mv:pmv:stv = 8.9:6.3:3.0:1.0.

**Gaster.** Overall longer than mesosoma with Gt1 and Gt2 orangish brown to white, while alternating dark brown and orange-brown bands transversely in dorsal and lateral view (Figure 14A); posterior margin evenly rounded; apex narrow; syntergum pale brown with pointed apex; gastral tergites smooth to weakly coriaceous with pale brown setae; ovipositor sheaths half of length of mesotibial spur; pale brown; exserted with few setae.

**Distribution.** ORIENTAL: China (Hebei, Hubei).

**Host.** Unknown.

**Diagnosis.** *A. tigrinus* is morphologically close to *A. napoleonis* and *A. huijuanae*, but differs in: gaster with dark brown to orangish transverse bands (Figure 14A) vs. bands absent in both species; fore wing hyaline crossband constricted medially with few dark setae (Figure 14G) vs. crossband complete, without dark setae in both species.

#### 3.2.10. Anastatus (Anastatus) yalini Khan, Li & Peng, sp. nov. (Figure 15)

Zoobank:urn:lsid:zoobank.org:act:6BB070DC-7EF8-4A84-849B-D33E35756887

**Figure 15:**
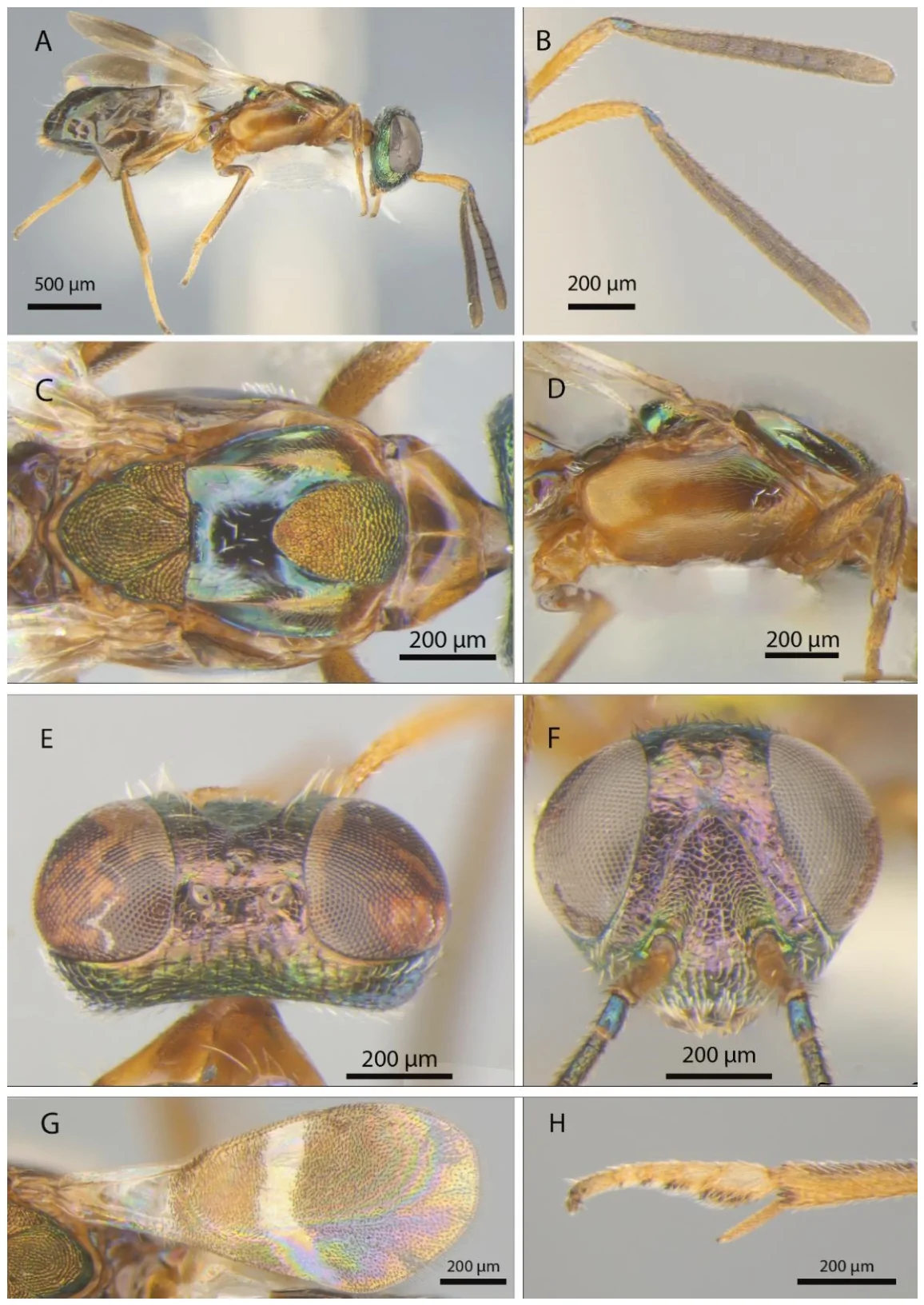
*Anastatus* (*Anastatus*) *yalini* **sp. nov.** holotype ♀ (FAFU-1257): (**A**) body lateral; (**B**) antennae; (**C**) mesosoma; (**D**) acropleuron; (**E**) head dorsal; (**F**) head front; (**G**) fore wing; (**H**) mesotibial spur and mesotarsus.

**Type material. Holotype.** ♀(FAFU), oil tea plantation, Xiaokeng Town, Shaoguan, Guangdong Province, China, 12.XII.2024–6.I.2025, Chen Sijie leg., FAFU-1257. **Paratypes.** 1♀(FAFU), Anhou, bamboo forest, Tianbaoyan Nature Reserve, Yong’an, Sanming, Fujian Province, China, 6.VII.2021, FAFU-889.

**Etymology.** The species is named *yalini* in honour of Dr. Yalin Zhang, supervisor of the corresponding author, who is a professor at Northwest A&F University.

**Description of holotype. FEMALE.** Body length excluding ovipositor sheaths 2.6 mm (Figure 15A).

**Head.** Overall violet blue with temple greenish blue to purple; vertex greenish to purple; frons bluish purple with green lustre; ocellus dark brown in dorsal view (Figure 15E,F); parascrobal region violet blue with margin having green metallic lustre; scrobal depression, including interantennal prominence bluish purple; torulus base and scrobal depression lower margin green; gena bluish green metallic lustre; clypeus bluish; labrum and mandible dark brown in frontal view. Temple reticulate to alutaceous, whereas frontovertex slightly mesh-like reticulate; upper face including parascrobal region and scrobal depression mesh-like coriaceous to reticulate, while upper parascrobal region with few pale setae; scrobal depression shallow and inverted V-shaped; lower face coriaceous to slightly alutaceous, and densely setose with white setae. Antenna (Figure 15B) with scape pale brown; pedicel dark bluish; flagellomeres dark violet blue except fl1 and fl2 green metallic lustre and densely setose with pale brown setae. Relative length (width) of scape 89.4(13.6), pedicel 19.7(11.8), fl1–fl8: 8.5(10.0), 23.4(12.1), 26.0(15.0), 33.1(17.6), 22.3(20.0), 22.6(20.2), 18.9(21.3), 20.0(20.5), clava 60.2(21.8); OOL:POL:LOL:MPOD = 1.0:3.3:1.8:1.5.

**Mesosoma.** Pronotum slightly pale brown to dark, except dark brown spots having slight blue lustre laterally (Figure 15C); mesoscutal medial lobe with anterior convex region, and scutellar–axillar complex bluish green to orangish lustre; lateral lobes bluish green to yellowish orange metallic lustre; posterior depressed region dark violet blue with slight green lustre; propodeum dark brown with bluish lustre. Pronotum smooth; mesoscutal medial lobe with anterior convex region strongly reticulate; lateral lobes alutaceous; posterior depressed region almost smooth with few setae medially; scutellar–axillar complex punctate-reticulate. Acropleuron (Figure 15D) overall pale yellow; mesopectus dark greenish with orangish metallic lustre, and with few non-uniform white setae anteriorly. Legs (Figure 15A,D,H) pale to dark brown, except pro- and mesocoxa pale, whereas metacoxa dark bluish green to purple with few white setae; meso- and metatarsus pale brown to white except dark mesotarsal pegs and claws; spur light brown with small dark spot posteriorly. Fore wing (Figure 15G) macropterous; covered with dark setae except hyaline crossband behind mv with few short white setae; basal cell and costal cell almost bare with one to two lanceolate setae at costal margin; parastigma of smv covered by dense dark setae; dark setal pattern of hyaline crossband margin non-uniform; stv short and unclear. Relative measurements of smv:mv:pmv:stv = 7.3: 6.0: 2.5: 1.0.

**Gaster.** Overall dark brown with greenish to purple metallic lustre posteriorly except Gt1 and Gt2 pale brown to white (Figure 15A); finely coriaceous to alutaceous posteriorly with few pale brown setae; ovipositor sheaths short; exserted; pale brown; densely setose with light brown setae, and as long as first segment of mesotarsus.

**Distribution.** ORIENTAL: China (Guangdong, Fujian).

**Host.** Unknown.

**Diagnosis.** *A. yalini* is morphologically close to *A*. *japonicus*, but differs in: posterior lobe of mesoscutum and mesopectus with few white setae (Figure 15C) vs. densely setose with white setae in *A. japonicus* (Figure 30A,B); basal cell of fore wing bare (Figure 15G) vs. not bare in *A. japonicus* (Figure 30C); hyaline crossband with white setae not evident (Figure 15G) vs. densely setose with white setae in *A. japonicus* (Figure 30C); fore wing apex rounded (Figure 15G) vs. sharper in *A. japonicus* (Figure 30C). *A. yalini* also similar to *A. nozdormu*, but differs in: posterior lobe of mesoscutum dark greenish purple to violet, few setae medially (Figure 15C) vs. pale brown, setae scattered evenly throughout in *A. nozdormu* (Figure 13C); callus of propodeum narrower (Figure 15C) vs. broader in *A. nozdormu* (Figure 13C).

#### 3.2.11. Anastatus (Cladanastatus) alexstrasza Khan, Li & Peng sp. nov. (Figure 16)

Zoobank:urn:lsid:zoobank.org:act:DF28BD8E-6F1C-4D70-A90A-8A35115BCB1C

**Figure 16:**
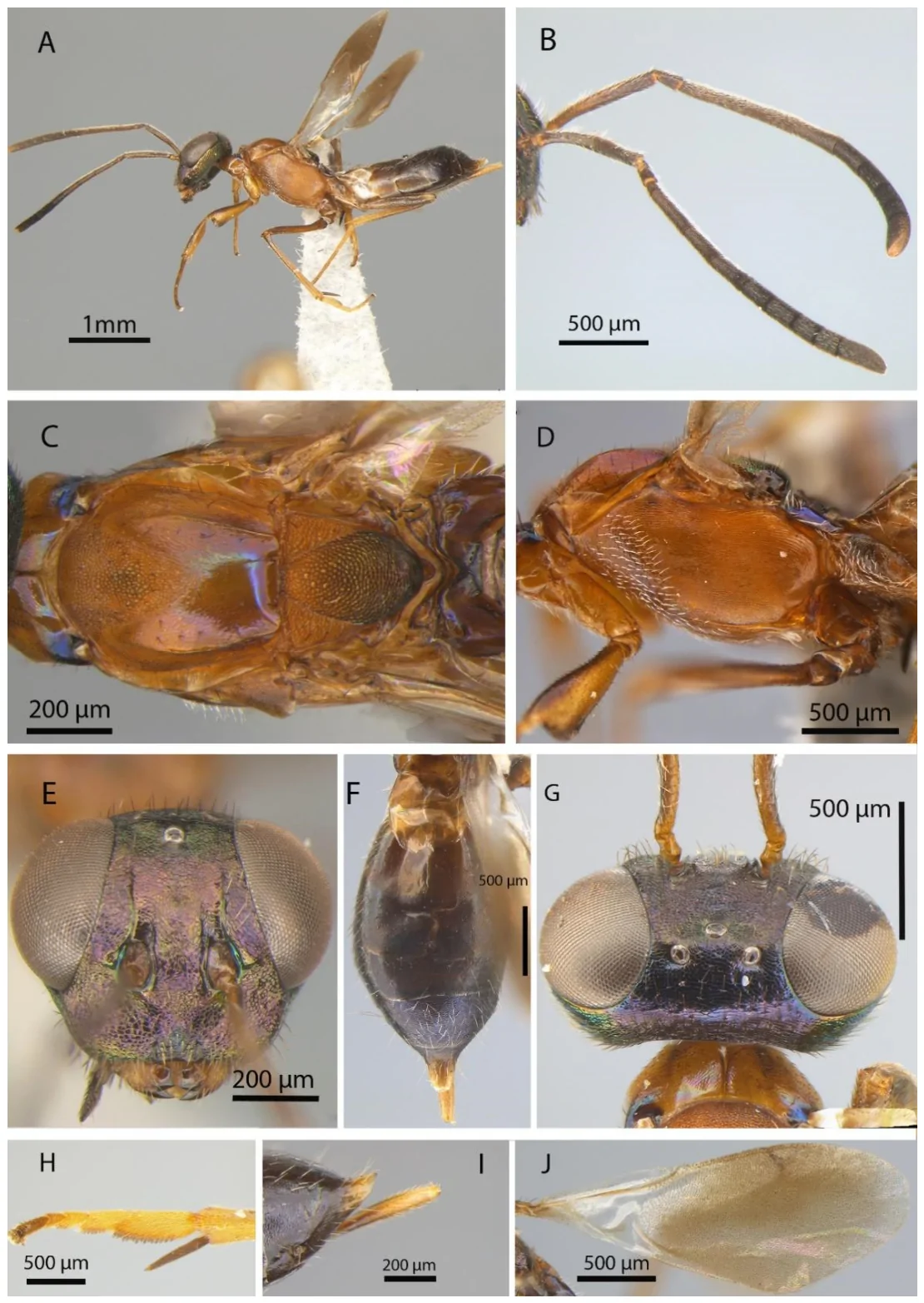
*Anastatus* (*Cladanastatus*) *alexstrasza* **sp. nov.** holotype ♀ (FAFU-946, except for **A**–**C**,**E**,**I**,**J**): (**A**) body lateral (FAFU-1128); (**B**) antennae (FAFU-1128); (**C**) mesosoma (FAFU-1128); (**D**) acropleuron; (**E**) head front (FAFU-1128); (**F**) gaster dorsal and ovipositor sheaths; (**G**) head dorsal; (**H**) mesotibial apical spur and mesotarsus; (**I**) ovipositor sheaths (FAFU-1128); (**J**) fore wing (FAFU-1230).

**Type material. Holotype.** ♀(FAFU), Haizhu Wetland, Guangzhou, Guangdong Province, China, Malaise trap, 20.V–20.VI.2022, FAFU-946. **Paratypes.** 1♀(FAFU), Wuguishan Nature Reserve, Guangdong Province, China, Malaise trap, 28.V–11.VI.2020, FAFU-1128; 1♀(FAFU), Dapeng Peninsula, Shenzhen, Guangdong Province, China, Malaise trap, 31.V–30.VI.2021, Chen Longlong leg., FAFU-1230.

**Etymology.** The species epithet *alexstrasza* is a noun in apposition derived from Alexstrasza the Life-Binder, queen of the red dragonflight in World of Warcraft, in recognition of the species’ reddish-orange body colouration, reminiscent of the fiery red scales of the dragon-queen. The name is treated as a noun in apposition.

**Description of holotype. FEMALE.** Body length excluding ovipositor sheaths about 3.7 mm (Figure 16A).

**Head.** Overall purple with temple green laterally in dorsal view (Figure 16E,G); vertex with slight bluish metallic lustre; ocellus pale; ocellar triangle and frons with bluish green metallic lustre; parascrobal region slightly greenish to blue by upper region, while lower region bluish to purple; scrobal depression shallow, whereas lateral margin sharp and upper margin indistinct with broad ∩-shaped margin convex at ends; torulus margin bluish; interantennal prominence bluish purple with green lustre; gena bluish green to purple metallic lustre; clypeus bluish; labrum brown with mandible and maxillary palps dark brown with dense setae in frontal view. Temple reticulate to alutaceous; frontovertex deeply reticulate; upper face and lower face reticulate with dense setae on lower face. Antenna (Figure 16B) dark brown except fl1 to fl4 with white setae. Relative length (width) of scape 96.3(11.4), pedicel 22.0(10.4), fl1–fl8: 11.1(10.0), 45.0(11.1), 33.0(12.5), 36.0(13.0), 26.1(15.3), 28.0(16.6), 23.8(17.4), 22.2(18.3), clava 44.4(28.0); OOL:POL:LOL:MPOD = 1.0:2.5:1.2:1.0.

**Mesosoma.** Overall reddish to orange with sparse dark setae except bluish spots on pronotum laterally in dorsal view (Figure 16C); mesoscutal medial lobe with posterior depressed region weakly bluish purple; scutellar–axillar complex with green metallic lustre posteriorly; propodeum dark brown with long plical region and V-shaped plical depression, propodeal spiracles pale brown. Mesoscutal medial lobe with anterior convex region reticulate anteriorly and weakly coriaceous to smooth posteriorly; lateral lobes smooth to longitudinally alutaceous; scutellar–axillar complex strongly reticulate. Acropleuron (Figure 16D) reddish to orangish; overall alutaceous. Legs (Figure 16A,D) with front leg dark except coxa, lateral of femur, and posterior of tibiae; middle and hind legs dark except posterior of tibiae and tarsus; spur dark brown (Figure 16H). Fore wing (Figure 16J) macropterous; costal cell not completely bare dorsally except few setae medially; basal cell almost bare except few setae; discal and apical region completely infuscate with brown setae; basal part of smv with six to seven lanceolate setae; cubital area and vannal area completely bare, except mediocubital fold with few setae. Relative measurements of smv:mv:pmv:stv = 8.2:5.2:3.6:1.0.

**Gaster.** Overall dark brown except Gt1 and Gt2 pale, completely coriaceous to reticulate with dense white setae laterally (Figure 16A,F); syntergal flange smooth and pale; ovipositor sheaths darker at base and pale yellow at tip with white setae, whereas length of ovipositor sheaths about 0.13× length of gaster and 0.29× length of hind tibia.

**Distribution.** ORIENTAL: China (Guangdong).

**Host.** Unknown.

**Diagnosis.** *A*. (*Cladanastatus*) *alexstrasza* is morphologically close to *A*. (*Cladanastatus*) *madagascariensis* Bouček, 1979, but differs in: ovipositor sheaths much shorter 0.13× gaster length and 0.29× hind tibial length (Figure 16A,F), vs. 0.38× and 0.42×, respectively, in *A. madagascariensis* [7]; syntergum sharply pointed (Figure 16F) vs. more rounded in *A*. (*Cladanastatus*) *madagascariensis*.

### 3.3. New Record from China

#### *Anastatus* (*Anastatus*) *mantoidae* Motschulsky rec. nov. (Figure 17)

*Anastatus mantoidae* Motschulsky, 1859: 116–117. Lectotype ♀ [ZMMU] designated by Bouček, 1988: 550.

*Anastatus mantoidae*; Motschulsky, 1863: fig. 11.

*Podagrion mantoidae*; Dalla Torre, 1898: 369; Schmiedeknecht, 1909: 117; Pruthi & Mani, 1940: 3, fig. 2.

*Anastatus mantoidae*; Gahan & Fagan, 1923: 12 [generic status]; Mani, 1938: 42 [catalogue]; Islam & Hayat, 1986: 57 [catalogue]; Mani, 1989: 691–692, fig. 160 [figure reproduced from Pruthi & Mani, 1940, fig. 2 and labelled as *A. mantoidae*; misidentification]; Grissell, 1995: 242 [nomenclatural history].

*Anastatus* (*Anastatus*) *mantoidae*; Narendran, 2009: 85–86, 94, fig. 39 [key].

*Anastatus* (*Anastatus*) *mantoidae*; Gibson, 2020: 488–497, figs. 1–6. Described both sexes.

**Material examined.** 1♀(FAFU), Shangsi, Shiwandashan, Guangxi Province, China, 16.VIII.2020, FAFU-1576.

**Description.** See Gibson, [5].

**Distribution.** ORIENTAL: *China (Guangxi), Indonesia (Java, Sumatra), Sri Lanka, Thailand.

**Host.** Mantodea: Eggs of Mantidae [5].

**Remarks.** *A. mantoidae* is close to *Anastatus motschulskyi* while variations in these species are explained in Gibson, [5].

**Figure 17:**
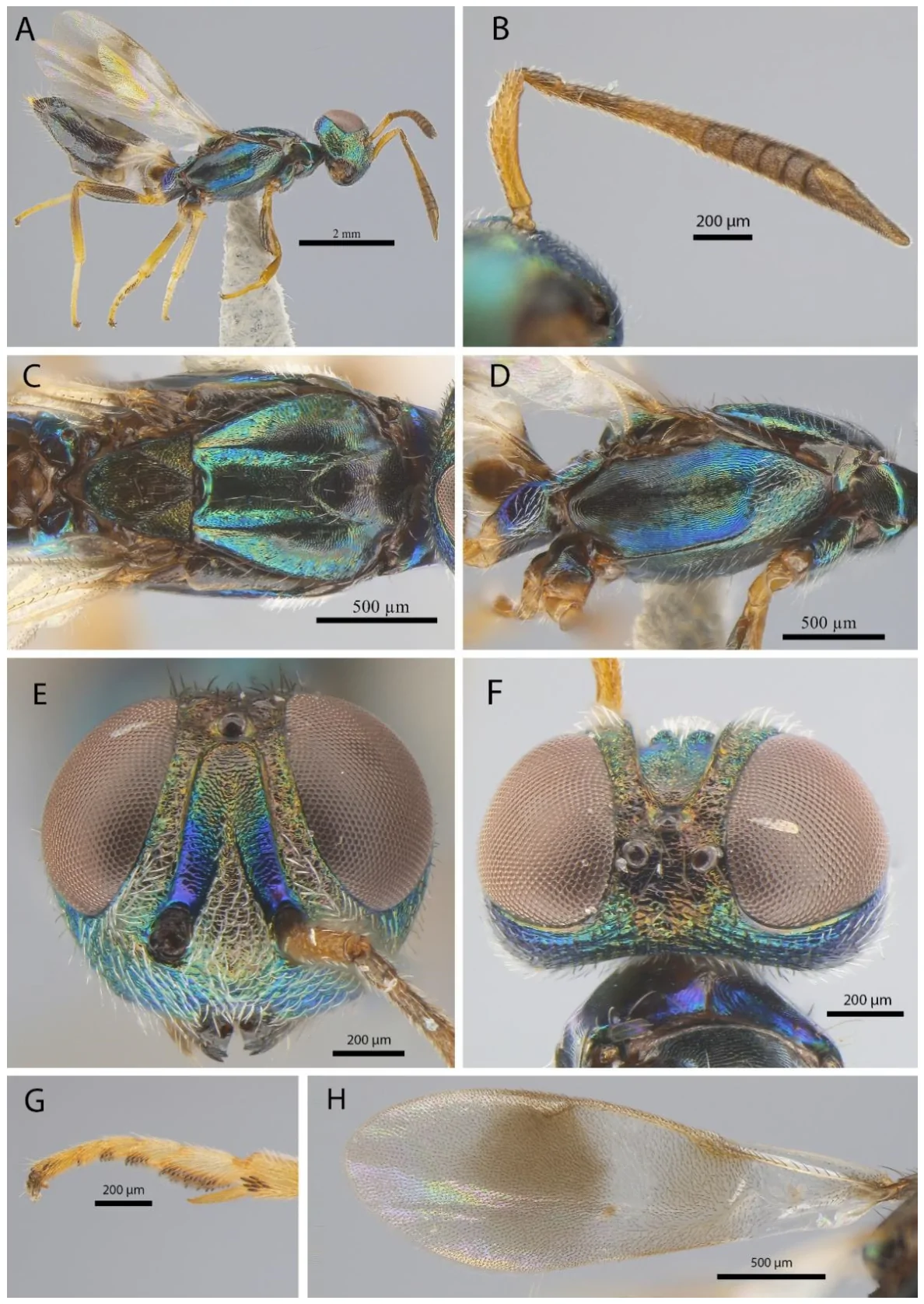
*Anastatus* (*Anastatus*) *mantoidae* (FAFU-1576) ♀: (**A**) body lateral; (**B**) antenna; (**C**) mesosoma; (**D**) acropleuron; (**E**) head front; (**F**) head dorsal; (**G**) mesotibial spur and mesotarsus; (**H**) fore wing.

### 3.4. Already Described Chinese Anastatus Species

#### 3.4.1. Anastatus (Anastatus) bifasciatus Geoffroy, 1785

*Cynips bifasciatus* Geoffroy in Fourcroy 1785: 388. Described: female.

*Diplolepis bifasciata* (Geoffroy); Dalla Torre, 1898: 418.

*Anastatus bifasciatus* (Geoffroy); Nikol’skaya, 1952: 490, fig. 477.

*Anastatus* (*Anastatus*) *bifasciatus* (Geoffroy); Gibson, 1995: 105 (by implication); Narendran, 2009: 78, fig. 12.

*Cinips bifasciata* Fonscolombe, 1832: 294. Described: female. Synonymy by Bouček, 1970: 80.

*Pteromalus bifasciatus* (Fonscolombe); Nees ab Esenbeck, 1834: 426.

*Eupelmus bifasciatus* (Fonscolombe); Förster, 1860: 122.

*Anastatus bifasciatus* (Fonscolombe); Ruschka, 1921: 264.

*Anastatus eurycephalus* Masi, 1919: 321–324, figs 26(1–5), 27. Described: female. Synonym of *A. bifasciatus* (Fonscolombe) by Bolívar y Pieltain, 1935: 280; synonymy by Bouček, 1970: 80.

*Cerycium pretense* Erdős, 1946: 138–139, figs 4a–b. Described: male. Synonymy by Bouček, 1970: 80.

*Cinips bombycum* Fonscolombe, 1832: 295. Described: male. Synonymy by Graham, 1992: 1101.

*Diplolepis bombycum*; Dalla Torre, 1898: 418.

*Eupelmus subaeneus* De Stefani, 1898: 251. Described: female. Synonymy by Bouček, 1970: 80.

*Pteromalus oomyzus* Rondani, 1872: 202–203. Described: male (see Bouček 1974: 261). Synonymy by Bouček, 1974: 261, 277.

*Misocoris oomyzus*; Rondani, 1877: 187, plate II, figs 55–58.

*Misocoris oophagus* Rondani, 1877: 187, plate II, figs 61–63. Described: male. Synonymy by Bouček, 1974: 262, 277.

*Pteromalus ovivorus* Rondani, 1872: 203. Described: female. Synonymy by Bouček, 1974: 262, 277.

*Misocoris ovivorus*; Rondani, 1877: 187, plate II, figs 59–60.

*Cynips gemmarum* Fonscolombe, 1832: 296. Described: male. Synonymy by Bouček, 1970: 80.

*Pteromalus gemmarum*; Nees ab Esenbeck, 1834: 425.

*Anastatus (Anastatus) bifasciatus*, Peng et al., 2020: 357–361, figs. 1,2. Described: both sexes.

Material examined. None.

**Description.** See Peng et al. [4].

**Distribution.** PALAEARCTIC: Europe, Middle East, northern Africa, and East Asia (Japan and Korea); AFROTROPICAL: Kenya; ORIENTAL: India; and NEARCTIC: United States [4].

**Hosts.** *A. bifasciatus* has approximately 30 host species belonging to Hemiptera, Lepidoptera, and Orthoptera, and has also been recorded as a hyperparasitoid on two braconid species (Hymenoptera). Nevertheless, some of these host associations are likely questionable, based on some misidentified parasitoid specimens mentioned by Peng et al. [4].

#### 3.4.2. *Anastatus* (*Anastatus*) *caeruleus* Wang & Peng, 2024

*Anastatus caeruleus*, Wang, et al., 2024: 6–8, fig. 4.

**Type material examined. Holotype.** ♀(FAFU), Lichee Orchard, Longmen Road, Danzhou, Hainan Province, China, 140 m, 23.III.2017, Liu Jiasheng leg., FAFU-176.

**Non-type material examined.** 3♀(FAFU), Longmen Road Orchard, Danzhou, Hainan Province, China, 23.III.2017, with no DNA information; 1♀, Nanmao Lake Park, Dehong, Yunnan Province, China, 766 m, 6.IV.2017, FAFU-657; 1♀, Hainan University, Danzhou, Hainan Province, China, 24.III.2017, 153 m, FAFU-604.

**Description.** See Wang et al. [1].

**Distribution.** ORIENTAL: China (Hainan, *Yunnan).

**Host.** Hemiptera: Tessaratomidae: Eggs of *Tessaratoma papillosa* (Drury) [1].

#### 3.4.3. *Anastatus* (*Anastatus*) *colemani* Crawford, 1912

*Anastatus colemani* Crawford, 1912: 6–7. Described: female.

*Anastatus* (*Anastatus*) *colemani*; Narendran, 2009: 78–79.

*Anastatus colemani*; Hu et al., 2011: 483, fig. 2. Misidentification of *A. japonicus*.

*Anastatus* (*Anastatus*) *colemani*, Peng et al., 2020: 361–363, fig. 3.

Material examined. None.

**Description.** See Peng et al. [4].

**Distribution.** ORIENTAL: China (Taiwan), India, Malaya, and Nepal [4].

**Hosts.** *A. colemani* was obtained from the eggs of *Degonetus serratus* (Hemiptera: Pentatomidae), and *Attacus atlas* (Lepidoptera: Saturniidae). A further record lists the lac insect, *Kerria lacca* (Hemiptera: Kerriidae) [4].

#### 3.4.4. *Anastatus* (*Anastatus*) *daiyunensis* Wang & Peng, 2024 (Figure 18)

*Anastatus daiyunensis*, Wang et al., 2024: 8–10, fig. 5.

**Type material examined. Holotype.** ♀(FAFU), Houzhai, Daiyun Mountain Natural Reserve, Dehua County, Fujian Province, China, 770 m, 15.VII.2015, Malaise trap, FAFU-267. **Paratypes.** 2♀(FAFU), same data as holotype, FAFU-268, 269.

**Non-type material examined.** 3♀(FAFU), Chebaling, Shaoguan, Guangdong Province, China, 21–23.VIII.2003, FAFU-309, 310, 313; 1♀(FAFU), Yangshan, Qingyuan, Guangdong Province, China, 7–27.VI.2021, FAFU-949; 1♀(FAFU), Wuguishan Nature Reserve, Zhongshan, Guangdong Province, China, 22.VII.2021–22.I.2022, FAFU-958; 1♀(FAFU), Chengjia Nature Reserve, Yangshan, Qingyuan, Guangdong Province, China, 18.VII–8.VIII.2021, FAFU-991; 1♀(FAFU), Chengdun, Wuyishan, Nanping, Fujian Province, China, 530 m, 28.VII.2022, Malaise trap, FAFU-1227; 1♀(FAFU), Jinuo Mountain, Jinghong, Xishuangbanna, Yunnan Province, China, 24.VIII.2022, FAFU-1271; 1♀(FAFU), Minjiangyuan Nature Reserve, Jianning, Sanming, Fujian Province, China, 554 m, 30.VII.2025, FAFU-1715.

**Description.** See Wang et al. [1].

**Distribution.** ORIENTAL: China (Fujian, *Guangdong, *Yunnan).

**Host.** Unknown.

**Figure 18:**
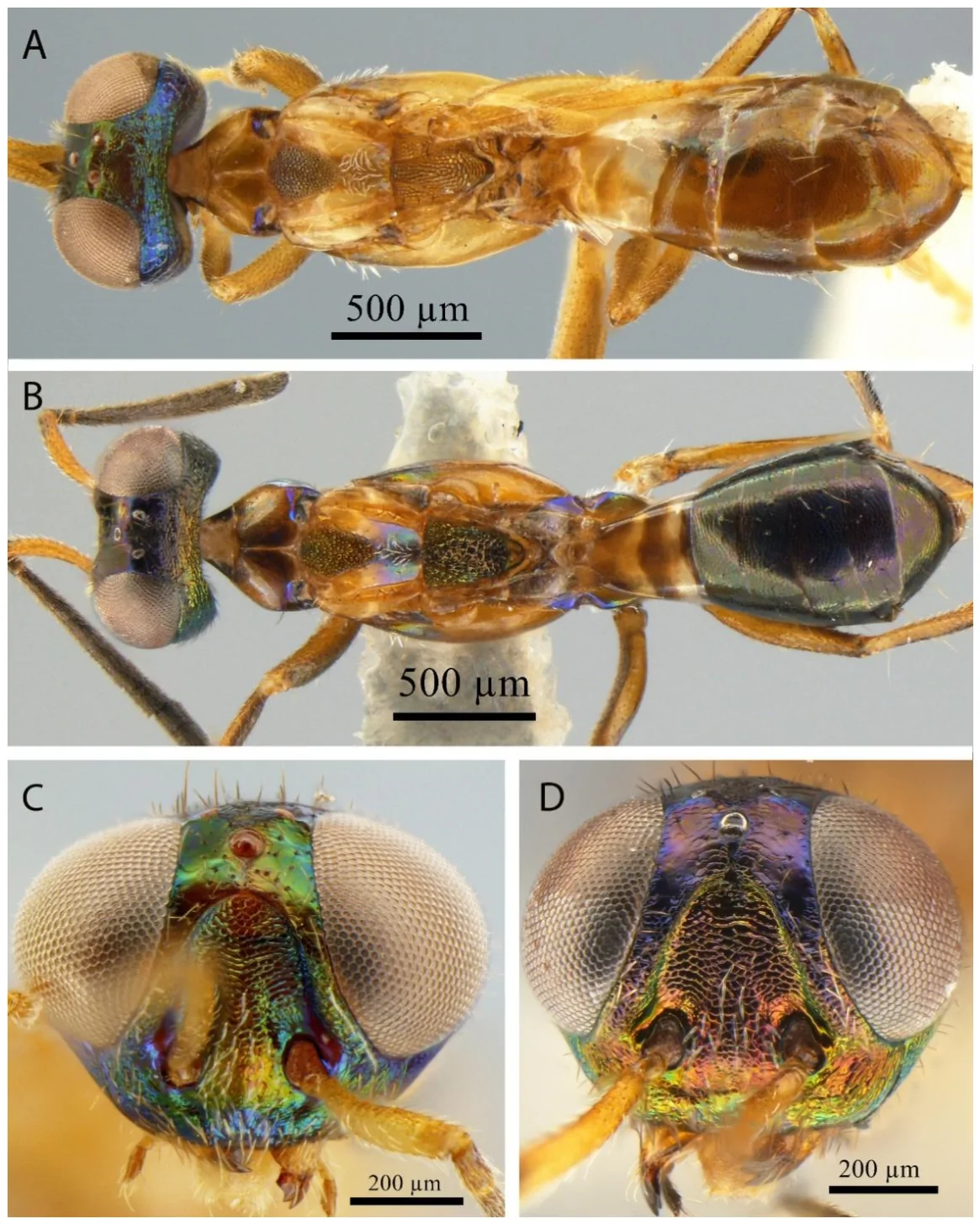
*Anastatus* (*Anastatus*) *daiyunensis* ♀: (**A**) (FAFU-310), body lateral; (**B**) (FAFU-1227), body lateral; (**C**) (FAFU-991), head front; (**D**) (FAFU-1227), head front.

**Remarks.** *A. daiyunensis* is morphologically close to *A. pariliquadrus*, but the upper portion of the parascrobal region and the region around the anterior ocellus are rugose or reticulate in *A. pariliquadrus*, whereas they are weakly coriaceous to smooth in *A. daiyunensis* (Figure 18C,D).

#### 3.4.5. *Anastatus* (*Anastatus*) *dexingensis* Sheng & Wang, 1997 (Figure 19)

*Anastatus dexingensis* Sheng and Wang, in Sheng et al. 1997: 59 (Chinese description), 63 (English synopsis), figs 6–9. Described: female.

*Anastatus kashmirensis*; Hu et al., 2011: 483–484, fig. 1. Misidentification.

*Anastatus dexingensis*; Peng et al., 2017: 4–6, figs 1–7.

*Anastatus* (*Anastatus*) *dexingensis*, Peng et al., 2020: 363–366, figs. 4,5. Described: both sexes.

**Material examined.** 1♀(FAFU), Wuyishan, Nanping, Fujian Province, China, Malaise trap, 350 m, 2.XII.2022, FAFU-1247; 1♀(FAFU), Guadun, Wuyishan, Nanping, Fujian Province, China, 28.VII.2022, FAFU-1300; 1♀(FAFU), Minjiangyuan Reserve Jianning, Fujian Province, China, 600 m, 2.XII.2025, FAFU-1511; 8♀(FAFU), Chebaling National Nature Reserve, Shaoguan, Guangdong Province, China, 20–31.VIII.2021, FAFU-1248; 11.IX.2022, FAFU-1259; 1–11.X.2022, FAFU-1272; 20–30.X.2022, FAFU-1281; 10.XI.2022, FAFU-1251; 31.VII.2022, FAFU-1596; 11–20.X.2022, FAFU-1599; 11.IX.2020, FAFU-1304; 1♀(FAFU), No specific locality (Guizhou Province), China, 10–20.VIII.2020, FAFU-1282; 3♀(FAFU), Guizhou University laboratory culture, Guizhou Province, China, VII.2025, Haoran Liao leg., FAFU-1309, 1311, 1734.

**Description.** See Peng et al. [4].

**Distribution.** ORIENTAL: China (Fujian, *Guangdong, *Guizhou, Hainan, Jiangxi, Taiwan), India (Odissa) [4].

**Hosts.** Hemiptera: Tessaratomidae: *Tessaratoma papillosa*; Lepidoptera: Lasiocampidae: *Dendrolimus kikuchii* Matsumura, 1927 and Saturniidae: *Samia cynthia* [4].

**Figure 19:**
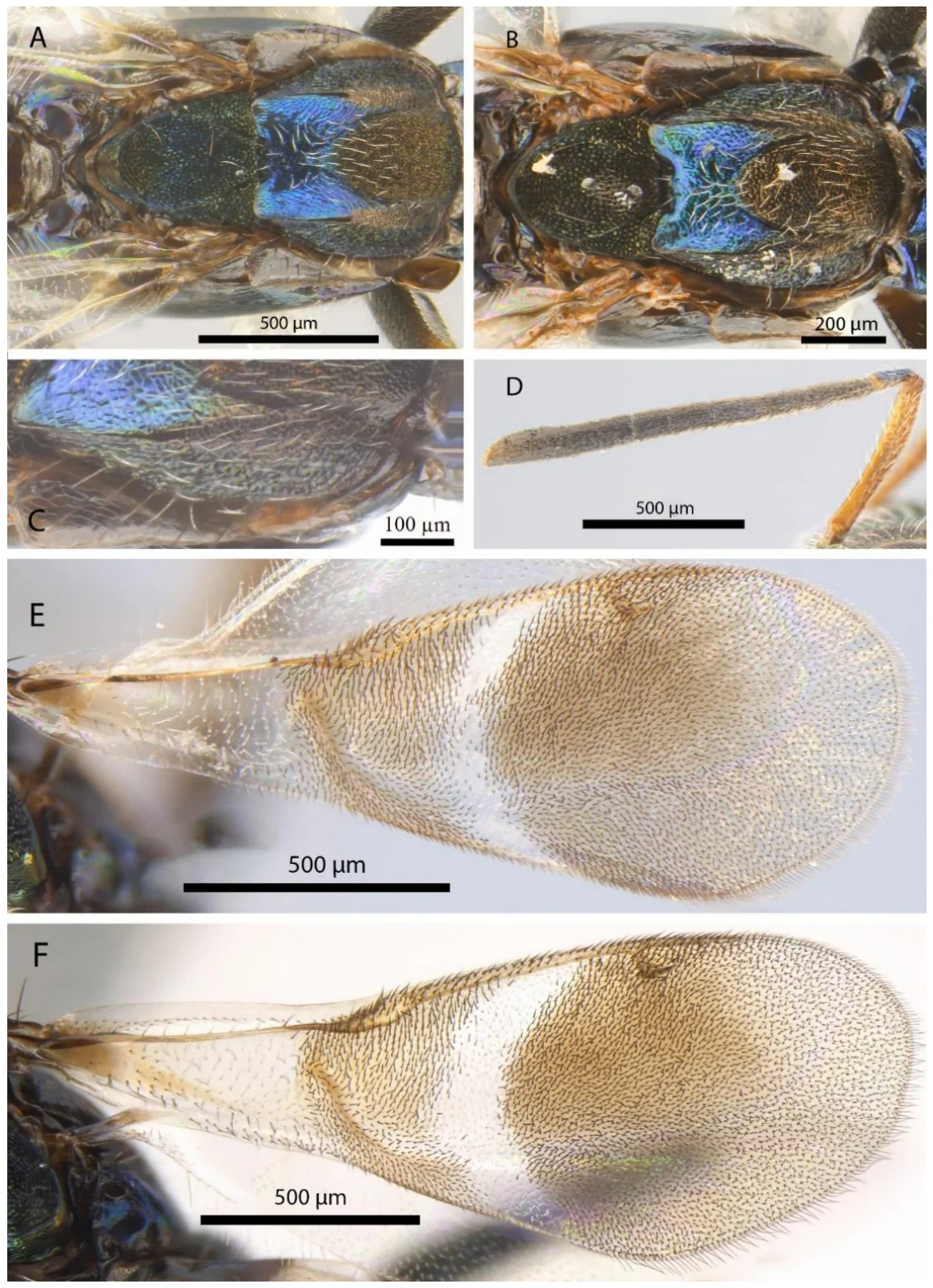
*Anastatus* (*Anastatus*) *dexingensis* ♀: (**A**,**B**), mesosoma; (**A**) FAFU-1247; (**B**) FAFU-1251; (**C**) mesoscutal lateral lobe, FAFU-1599; (**D**) antenna, FAFU-1309; (**E**,**F**), fore wing; (**E**) FAFU-1599; (**F**) FAFU-1309.

**Remarks.** *A. dexingensis* is morphologically similar to *A. formosanus* and *A. garygibsoni* based on female morphology. For distinctions from *A. garygibaoni*, see the comparison section under that species. Females of *A. dexingensis* and *A. formosanus* are very close to each other, and the key designed by Peng et al. [4] is now unable to separate these two species. For example, Peng et al. [4] used mesoscutal lateral lobes sculpture which we found to be exactly identical between the two species (both almost reticulate-imbricate with minor variations that are not species specific) (Figure 19A–C and for figures of *A. formosanus*, see below in the *A. formosanus* section). The presence of few dark setae medially on the fore wing hyaline crossband, another character used by Peng et al. [4], is also inconsistent and variable when considered together with the crossband width ratio (Figure 19E,F). We also compared antennal segments using morphometric analyses but revealed no significant difference (Figure 19D). Therefore, in this study we used male morphology and *COI* to distinguish between these two species.

#### 3.4.6. *Anastatus* (*Anastatus*) *echidna* Motschulsky, 1863 (Figure 20)

*Cacotropia echidna* Motschulsky, 1863: 57, fig. 10. Described: female.

*Cacotropia echidna*; Islam and Hayat, 1986: 65.

**Figure 20:**
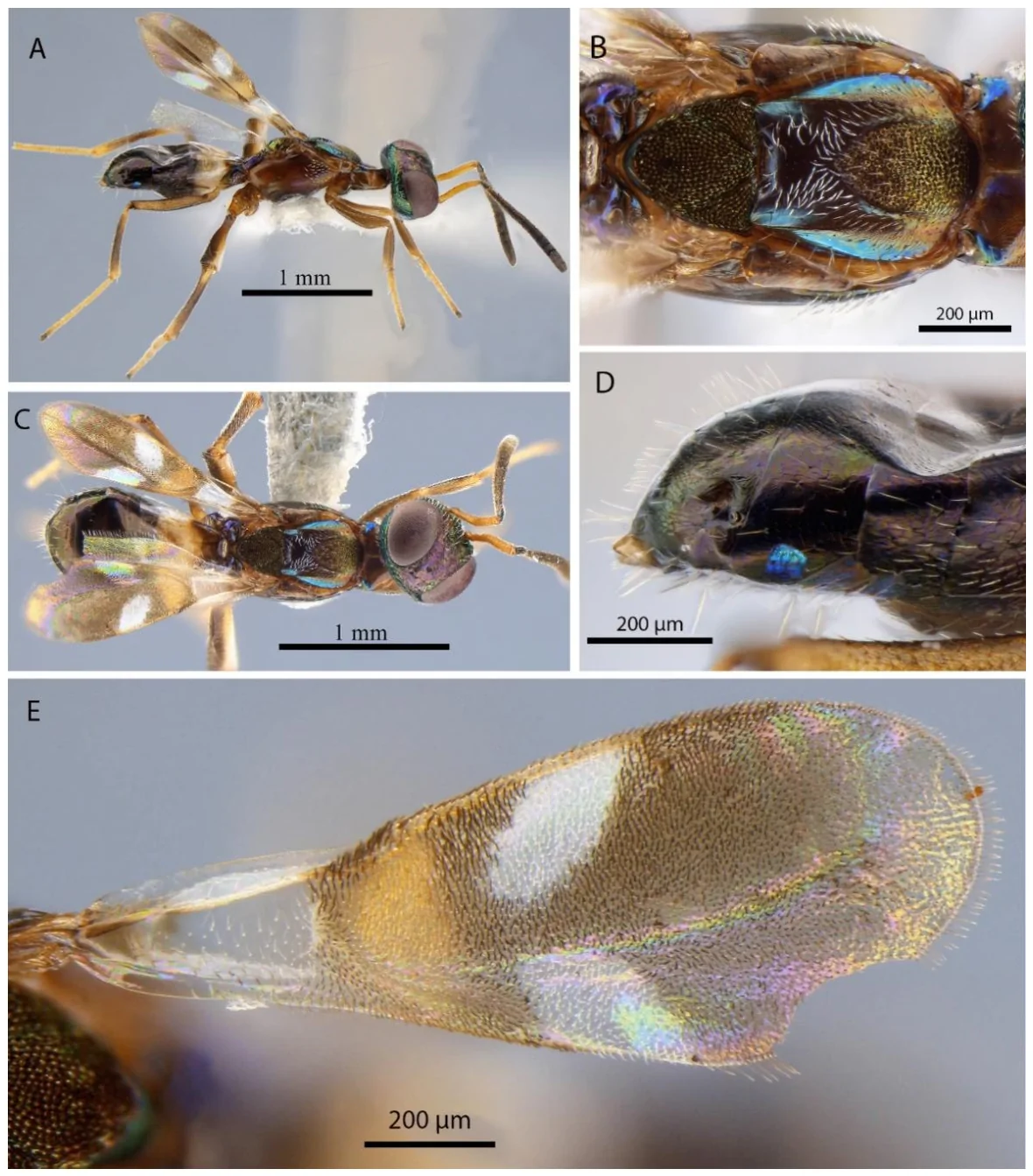
*Anastatus* (*Anastatus*) *echidna* ♀: (**A**–**E**) (FAFU-1368) (**A**) body lateral; (**B**) mesosoma; (**C**) body dorsal; (**D**) ovipositor; (**E**) fore wing.

*Anastatus echidna*; Bouček, 1988: 550.

*Anastatus* (*Anastatus*) *echidna*; Narendran, 2009: 81, 94; Gibson, 2020: 504–511.

*Anastatus acherontiae*; Yang et al., 2015b: 161–162, 255, fig. 82. Misidentification.

*Anastatus* (*Anastatus*) *echidna*, Peng et al., 2020: 366–369, fig. 6.

**Material examined.** 3♀(FAFU), Funiu Mountains, Luoyang, Henan Province, China, 21.IX.2023, FAFU-1367, 1368, 1369; 1♀(FAFU), peach orchard, Henan Province, China, 30.IX.2023, FAFU-1372.

**Description.** See Peng et al. [4].

**Distribution.** ORIENTAL: China (Fujian, Jiangxi, Hunan, Yunnan), India, Pakistan, Thailand; PALAEARCTIC: China (Hebei, *Henan) [4].

**Hosts.** Hemiptera: Pentatomidae: *Nezara* sp.; Lepidoptera: Erebidae: Arctiinae, Lasiocampidae: *Dendrolimus tabulaeformis* Tsai and Liu, 1962, *D. spectabilis*, Sphingidae [4].

**Remarks.** *A. echidna* is close to the newly described *A. neltharion*; further details are given in the remarks under *A. neltharion*. Based on *COI* analysis, specimens of *A. echidna* are mixed with the specimens of *A. meilingensis* in a single clade of the ML tree. See the discussion for further details on this part.

#### 3.4.7. *Anastatus* (*Anastatus*) *formosanus* Crawford, 1913 (Figure 21)

*Anastatus formosanus* Crawford, 1913: 249. Described: both sexes.

*Anastatus* (*Anastatus*) *formosanus*, Peng et al., 2020: 370–373, figs. 8, 9. Described: both sexes.

**Material examined.** 3♀(FAFU), Danzhou, Longmen Road orchard, Hainan Province, China, 140 m, 23.III.2017, FAFU-175; same locality (litchi tree), FAFU-1160, 1161; 3♀(FAFU), Hainan University, Hainan Province, China, 153 m, 24.III.2017, FAFU-1720, 1721, 1722; 1♀(FAFU), Wuzhishan, Hainan Province, China, 28.III.2021, FAFU-826; 1♀(FAFU), Woody Island, Sansha, Hainan Province, China, 18.IV.2019, FAFU-1250; 1♀(FAFU), Jianfengling, Hainan Province, China, 89.22 m, 30.X–30.XI.2019, Chunyang Xu leg., FAFU-1382; 2♀(FAFU), Zhega Village, Yuanjiang, Yuxi, Yunnan Province, China, 608 m, 11.IV.2017, FAFU-231, FAFU-235; 1♀(FAFU), Kaiping, Jiangmen, Guangdong Province, China, 2 m, 15.IV.2017, FAFU-232; 1♀(FAFU), Sanlingshan Forest Park, Zhanjiang, Guangdong Province, China, 17.VI.2024, Zhenhua Liu leg., FAFU-1414; 1♀(FAFU), Shaoguan, Chebaling National Nature Reserve, Guangdong Province, China, 10.XI.2022, FAFU-1454; 2♀(FAFU), ginger garden, South China Botanical Garden, Guangzhou, Guangdong Province, China, 9.VII–29.VIII.2025, Zhixuan Liu leg., FAFU-1725, 1726; 1♀(FAFU), *Camellia oleifera* forest, Chunwan, Yangchun, Guangdong Province, China, 379 m, 24.VI–22.VIII.2025, Sijie Chen leg., FAFU-1729; 2♀(FAFU), Fuzhou Fujian Province, China, 23.XI.2025, Ping Hou & Boyuan Chen leg., FAFU-1456; same site, VI.2025, FAFU-1589; 1♀(FAFU), Longxiang Island, Minhou, Fujian Province, China, 8.IV.2022, FAFU-1455; 1♀(FAFU), Lingshan, Qinzhou, Guangxi Province, China, 15.IV.2017, FAFU-1714; 1♀(FAFU), Taichung, Taiwan Province, China, 30.VII.2017, FAFU-244.

**Description.** See Peng et al. [4].

**Distribution.** ORIENTAL: China (*Fujian, Guangdong, *Guangxi, Hainan, Yunnan, Taiwan).

**Hosts.** Hemiptera: Pentatomidae: *Biprorulus bibax* and *Erthesina fullo*, Tessaratomidae: *Tessaratoma papillosa*; Lepidoptera, Lasiocampidae: *Dendrolimus kikuchii* and Saturniid: *Antheraea pernyi* [4].

**Remarks.** See the remarks section under *A. dexingensis*.

**Figure 21:**
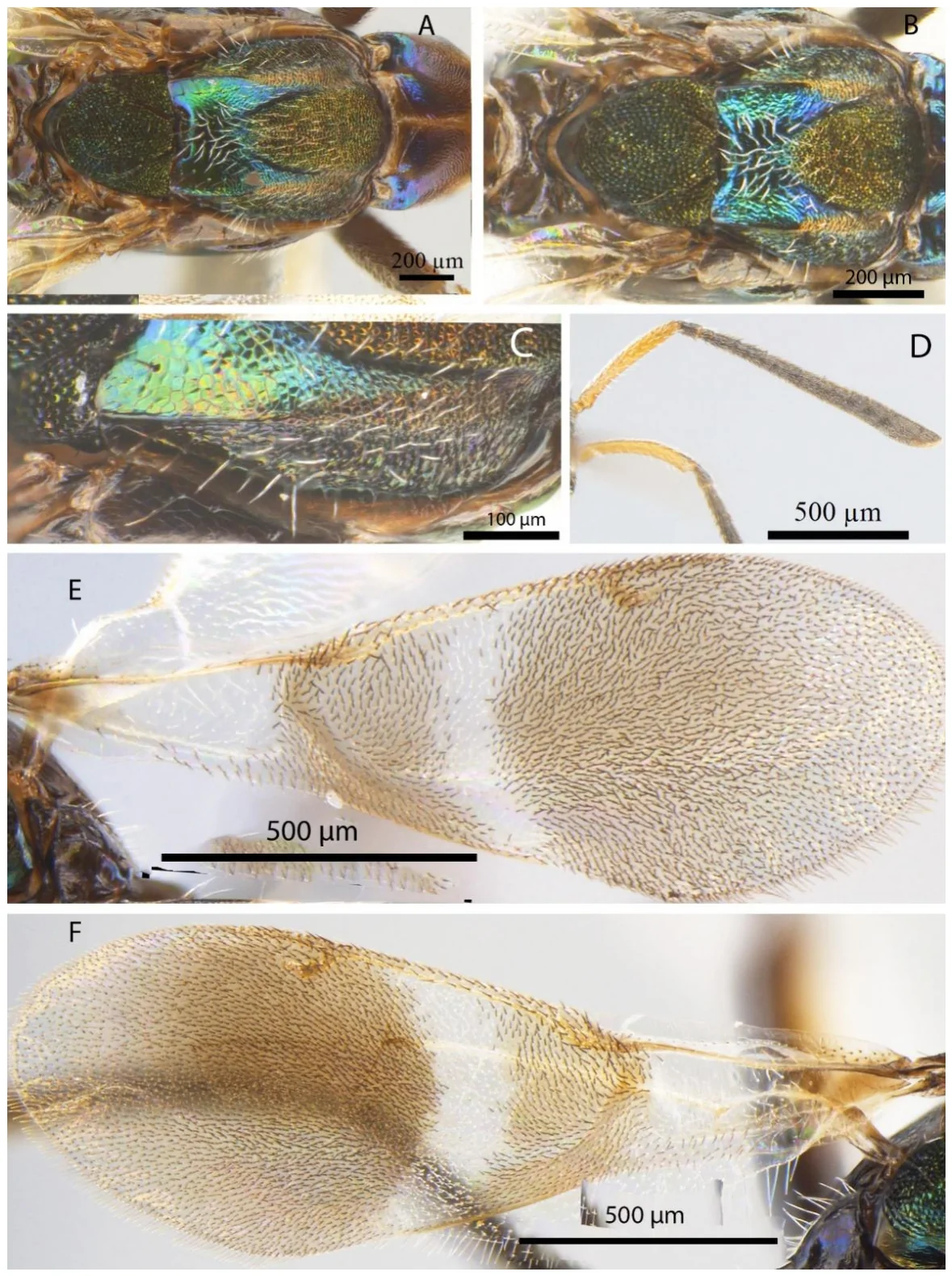
*Anastatus* (*Anastatus*) *formosanus* ♀: (**A**,**B**), mesosoma; (**A**) FAFU-1455; (**B**) FAFU-1456; (**C**) mesoscutal lateral lobe, FAFU-1454; (**D**) antennae, FAFU-1454; (**E**,**F**), fore wing; (**E**) FAFU-1382; (**F**) FAFU-1454.

##### Comparison of Males of *A. dexingensis* and *A. formosanus* (Figure 22 and Figure 23)

**Material examined.** *A. dexingensis* 3♂(FAFU): reared under laboratory conditions at Guizhou University, Guizhou Province, China, VII.2025, Liao Haoran leg., FAFU-1732, 1733, 1735.

**Description.** See Peng et al. [4]. (Fig. 5) (*A. dexingensis* ♂).

**Material examined.** *A. formosanus* 1♂(FAFU): reared from the eggs of *Tessaratoma papillosa*, Guangdong Province, China, IV.2025, HuiYang leg., voucher E0015510.

**Description.** See Peng et al. [4]. (Fig 9) (*A. formosanus* ♂).

**Figure 22:**
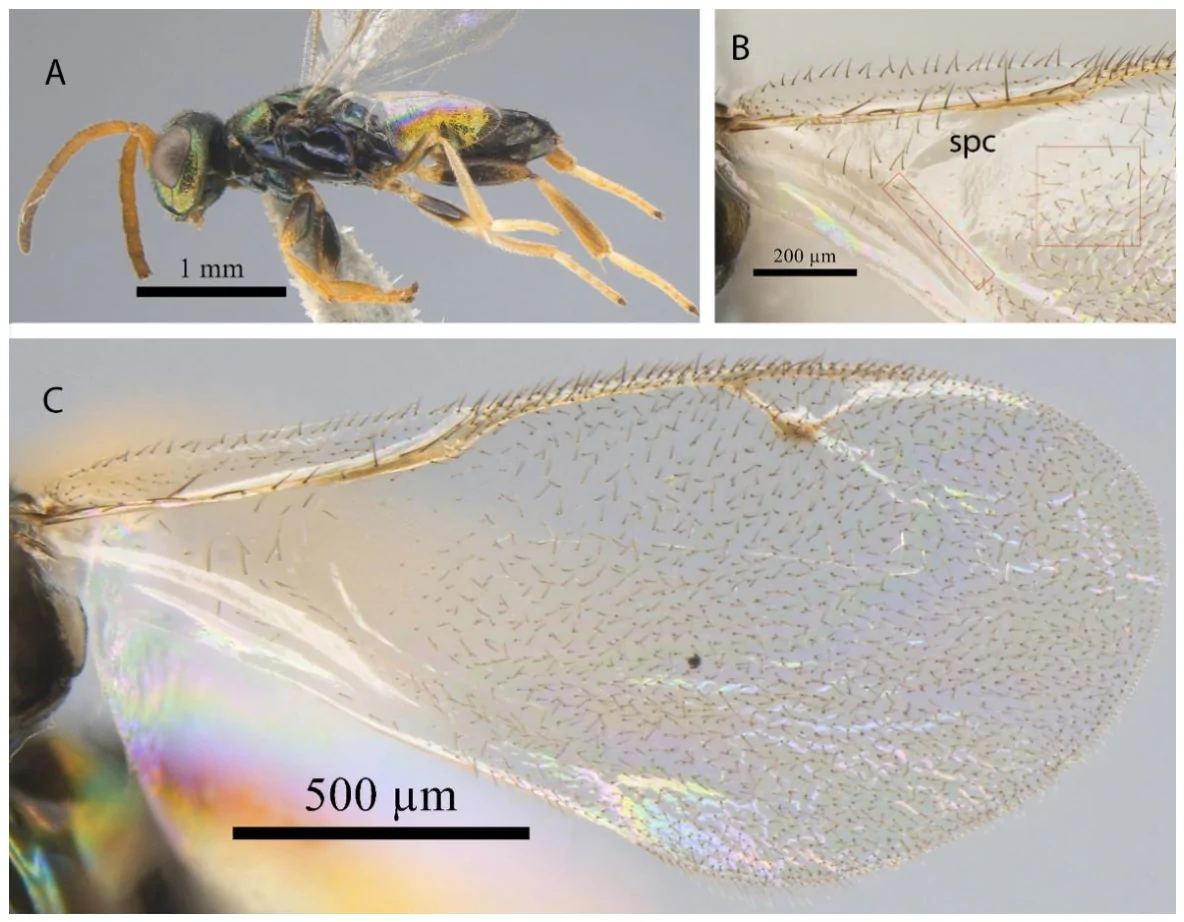
*Anastatus* (*Anastatus*) *dexingensis* ♂: (**A**) lateral body (FAFU-1732); (**B**) fore wing speculum (FAFU-1735); (**C**) fore wing (FAFU-1735).

**Figure 23:**
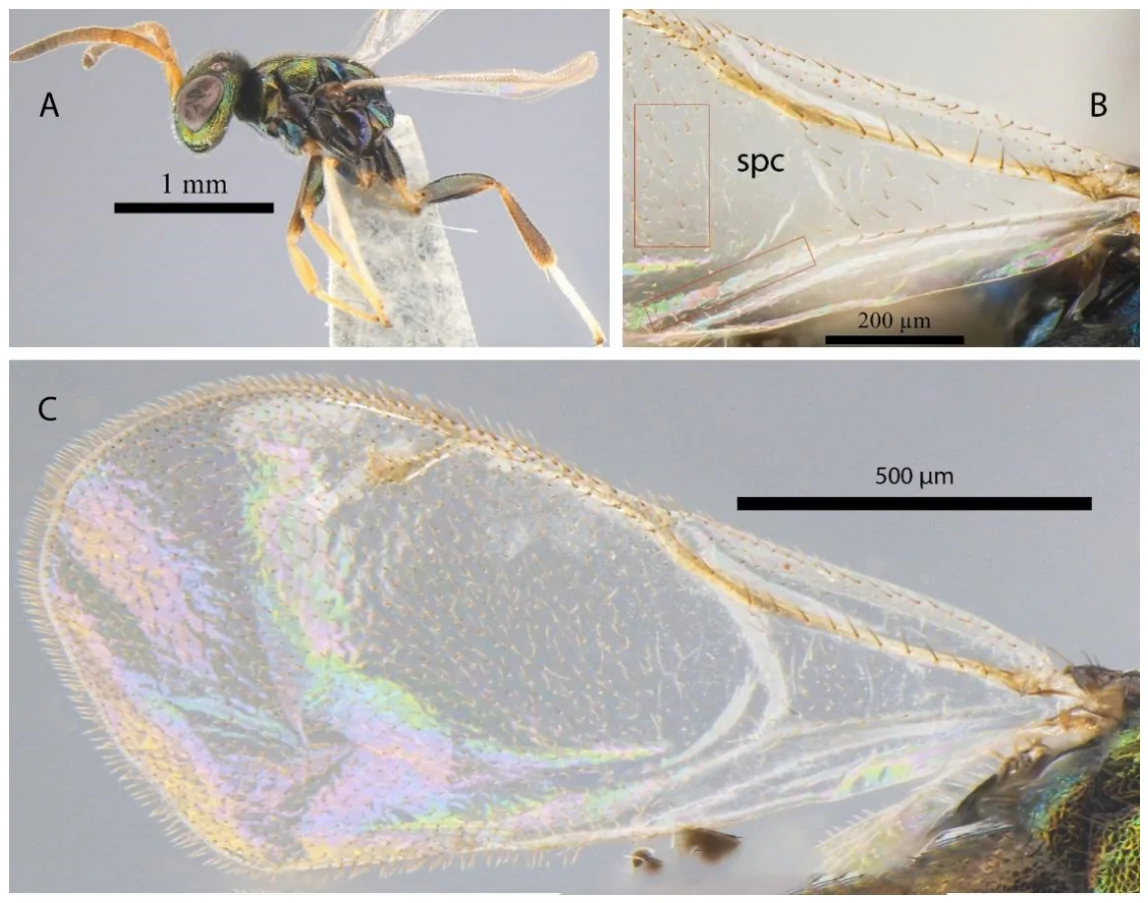
*Anastatus* (*Anastatus*) *formosanus* ♂: (voucher E0015510); (**A**) lateral body; (**B**) fore wing speculum; (**C**) fore wing.

**Remarks.** Males of *A. formosanus* and *A. dexingensis* are very similar. Peng et al. [4] distinguished them using two characters: (i) the costal cell of the fore wing is entirely setose in *A. dexingensis* (Figure 22C) but only on the apical half in *A. formosanus* (Figure 23C), and (ii) the speculum is closed posteriorly by a distinct line of setae in *A. dexingensis* (Figure 22C), whereas in *A. formosanus* this line is indistinct (Figure 23C). Although we encountered some difficulty in clarifying the first character, examination of figures revealed that the setal pattern on the costal cell is bolder and clearer in *A. dexingensis* (Figure 22), as noted by Peng et al. [4], whereas in *A. formosanus* the setae are less dense and conspicuous. Likewise, the posterior line of setae closing the speculum is distinct in *A. dexingensis* (Figure 22), but indistinct in *A. formosanus* (Figure 23).

#### 3.4.8. *Anastatus* (*Anastatus*) *fulloi* Sheng & Wang, 1997 

*Anastatus fulloi* Sheng and Wang, in Sheng et al. 1997: 61–62, figs 14, 15. Described: both sexes.

*Anastatus fulloi*; Peng et al., 2017: 10–13, figs 19–27.

*Anastatus* (*Anastatus*) *fulloi*; Chen et al., 2019: 115–117, fig. 1.

*Anastatus* (*Anastatus*) *fulloi*, Peng et al., 2020: 373–377, figs. 10,11. Described: both sexes.

**Material examined.** 2♀(FAFU), Campus of FAFU, Fuzhou, Fujian Province, China, 20.IX.2014, ex. eggs of *Erthesina fullo*, Peng Lingfei leg., FAFU-35, 36; 1♀(FAFU), same data, FAFU-151; 2♀(FAFU), Yangchun, Guangdong Province, China, 28.IV.2014, ex. eggs of *Tessaratoma papillosa*, Xiao Yuanbin leg., FAFU-60, 61; 1♀(FAFU), Hainan University, Danzhou, Hainan Province, China, 24.IV.2017, on litchi tree, FAFU-223; 1♀(FAFU), Xishan National Key Scenic Area, Guiping, Guigang, Guangxi Province, China, 14.IV.2017, on longan tree, FAFU-233; 1♀(FAFU), Fuzhou, Fujian Province, China, VI.2017, FAFU-248; 1♀(FAFU), Xinyi, Maoming, Guangdong Province, China, 13.IV.2017, on litchi tree, FAFU-296; 1♀(FAFU), Heshaba, Guizhou Province, China, 19.IX–7.X.2021, FAFU-1046; 1♀(FAFU), Fuzhou, Fujian Province, China, V.2024, Li Zixuan leg., FAFU-1146; 1♀(FAFU), Longxiang Island, Minhou, Fuzhou, Fujian Province, China, 9.IV.2021, FAFU-1159; 1♀(FAFU), Zhangjiangkou Mangrove Reserve, Yunxiao, Zhangzhou, Fujian Province, China, 6.VIII.2022, FAFU-1253; 1♀(FAFU), Xingangshan, Jiangxi Province, China, 19–29.IX.2018, FAFU-1275; 1♀(FAFU), Masson pine forest, Dinghushan Nature Reserve, Zhaoqing, Guangdong Province, China, 30.VIII–29.IX.2024, Mao Qinggong leg., FAFU-1277; 2♀(FAFU), Fangshan, Nanjing, Jiangsu Province, China, 3.XI.2025, Wang Qiyan & Liao Haoran leg., FAFU-1364, 1365; 1♀(FAFU), Shenshishan, Yunnan Province, China, 10.VIII.2022, FAFU-1383; 1♀(FAFU), Dinghushan Nature Reserve, Guangdong Province, China, 30.VIII–29.IX.2024, Mao Qinggong leg., FAFU-1415; 1♀(FAFU), Dinghushan Nature Reserve, Guangdong Province, China, 29.IX–30.X.2024, Mao Qinggong leg., FAFU-1416; 1♀(FAFU), Xingangshan, Jiangxi Province, China, 30.IX.2022, FAFU-1448; 2♀(FAFU), Fuzhou, Fujian Province, China, 8.IV.2019, ex eggs of *Dendrolimus punctatus*, FAFU-1582, 1583; 1♂(FAFU), Lingshan, Qinzhou, Guangxi Province, China, 15.IV.2017, FAFU-1713; 3♀(FAFU), Xishan National Scenic Area (longan trees), Guiping, Guigang, Guangxi Province, China, 14.IV.2017, FAFU-1717, 1718, 1719; 1♀(FAFU), Changzhou, Liyang, Huanhu North Road, Jiangsu Province, China, 97 m, 1–12.IX.2025, Liao Haoran leg., FAFU-1716; 1♀(FAFU), Dinghushan National Nature Reserve, mixed conifer–broadleaf forest, Guangdong Province, China, 135 m, 30.X–30.XI.2024, Mao Qinggong leg., FAFU-1728; 1♀(FAFU), Dinghushan National Nature Reserve, mixed conifer–broadleaf forest, Guangdong Province, China, 135 m, 2.I–1.II.2025, Mao Qinggong leg., FAFU-1731; 1♀(FAFU), Yangchun, Chunwan Town, *Camellia oleifera* forest, Guangdong Province, China, 347 m, 24.VI–22.VIII.2025, Chen Wenting leg., FAFU-1730.

**Description.** See Peng et al. [4].

**Distribution.** ORIENTAL: China (Fujian, Guangdong, Guangxi, Jiangxi, Taiwan, *Guizhou, *Hainan, *Yunnan). PALAEARCTIC: China (Gansu, Liaoning, *Hubei, *Jiangsu, *Shaanxi).

**Hosts.** Hemiptera and Lepidoptera: Within Hemiptera: Pentatomidae: *Erthesina fullo* and the Tessaratomidae: *Tessaratoma papillosa*; Lepidoptera: Lasiocampidae: *Dendrolimus punctatus*, Saturniidae: *Antheraea pernyi* and *Caligula japonica* Moore, 1862 [4,34].

**Remarks.** *A. fulloi* is close to *A. battutai*, whereas we distinguished these two species using antennal morphometrics, as explained in the Remarks section of *A. battutai* (Figure 4, Figure 5 and Figure 6).

#### 3.4.9. *Anastatus* (*Anastatus*) *gansuensis* Chen & Zang, 2019 (Figure 24)

*Anastatus* (*Anastatus*) *gansuensis* Chen and Zang, in Chen et al. 2019: 117–126, figs 2, 3A–E, 4.

*Anastatus* (*Anastatus*) *gansuensis*, Peng et al., 2020: 377–379, Figs 12, 13.

*Anastatus* (*Anastatus*) *gansuensis*, Li et al., 2025: 352–353, figs. 1C, 1D, 2.

**Material examined.** 2♀(FAFU), Jiulongchuan, Haicheng, Anshan, Liaoning Province, China, 20.VI.2015, Li Tao leg., FAFU-92, 94; 2♀(FAFU), Simingshan, Yuyao, Ningbo, Zhejiang Province, China, 5.VI.2017, FAFU-419, 420; 1♀(FAFU), Lin’an, Hangzhou, Zhejiang Province, China, 25.V.2016, FAFU-431; 5♀(FAFU), Huaxi Village, Anwen Town, Pan’an, Jinhua, Zhejiang Province, China, 26.IV.2014, FAFU-399, 404, 405, 406, 408; 3♀(FAFU), Longtangshan, Qingliangfeng, Lin’an, Hangzhou, Zhejiang Province, China, V.2012–VII.2013, FAFU-444, 447, 449; 2♀(FAFU), Haoping Management Station, Taibaishan, Mei County, Baoji, Shaanxi Province, China, 17.VIII.2016, 19.IX.2014, FAFU-758, 16; 1♀(FAFU), Luonan, Shangluo, Shaanxi Province, China, 21.XII.2018, FAFU-771; 15♀(FAFU), Ziwuling, Fuxian, Yan’an, Shaanxi Province, China, 11.VIII–4.IX.2018, FAFU-662, 685, 688, 690, 693, 711, 719, 725, 692, 729, 714, 718, 720, 724, 742, 743; 4♀(FAFU), Bamianyao, Ziwuling, Fuxian, Yan’an, Shaanxi Province, China, 8.VIII–3.IX.2018, FAFU-734, 735, 737, 739; 1♀(FAFU), Xunyangkan, Ningshan, Ankang, Shaanxi Province, China, 1.VII–11.VIII.2016, FAFU-756; 18♀(FAFU), Xingangshan, Shangrao, Jiangxi Province, China, 20.VI.2018–30.IX.2022, FAFU-1120, 1133, 1229, 1238, 1239, 1240, 1243, 1246, 1286, 1446, 1447, 1449–1451, 1458–1460; 2♀(FAFU), Jinggangshan, Jiangxi Province, China, 16.V.2022, FAFU-1461, 1462; 1♀(FAFU), Xingangshan, Jiangxi Province, China, 15.V.2022, FAFU-1513; 1♀(FAFU), Jiulianshan Nature Reserve, Longnan, Ganzhou, Jiangxi Province, China, 24.X–15.XI.2020, FAFU-981; 1♀(FAFU), Nanyuan, Wangbuling, Meiling, Nanchang, Jiangxi Province, China, 6.VII.2024, Du Jingzhe leg., FAFU-1134; 1♀(FAFU), Chunwan oil-tea forest, Yangchun, Guangdong Province, China, 9–24.XII.2024, FAFU-1218; 3♀(FAFU), Chebaling Nature Reserve, Shixing, Guangdong Province, China, 30.IX.2021–10.II.2022, FAFU-1244, 1258, 1595; 2♀(FAFU), Nanling National Nature Reserve, Shaoguan, Guangdong Province, China, Malaise trap, 22.IX–13.X.2020, FAFU-1242, 1249; 3♀(FAFU), Nanling National Nature Reserve, Shaoguan, Guangdong Province, China, VI–X.2020, FAFU-1280, 1580, 1581; 1♀(FAFU), Nanling National Nature Reserve, Shaoguan, Guangdong Province, China, 26.VI–28.IX.2021, FAFU-1241; 1♀(FAFU), Nanling National Nature Reserve, Shaoguan, Guangdong Province, China, Malaise trap, 28.IX–28.X.2021, FAFU-1598; 2♀(FAFU), Yangshan, Qingyuan, Guangdong Province, China, 7–27.VI.2021, FAFU-947, 948; 1♀(FAFU), Dinghushan Nature Reserve, Zhaoqing, Guangdong Province, China, 17.XII.2021–17.I.2022, FAFU-960; 1♀(FAFU), Haizhu Wetland, Guangzhou, Guangdong Province, China, 22.II–22.III.2022, FAFU-963; 1♀(FAFU), Lutian Village, Conghua, Guangzhou, Guangdong Province, China, Malaise trap, 17.XII.2021–17.I.2022, FAFU-993; 2♀(FAFU), Chebaling Nature Reserve, Shixing, Guangdong Province, China, X–XI. 2020, FAFU-983, 984; 2♀(FAFU), Tiejiaoshan, Lanling, Linyi, Shandong Province, China, 13–23.V.2020, FAFU-1285, 1406; 3♀(FAFU), Gaozhai Reservoir, Duyun, Qiannan, Guizhou Province, China, 28.VIII.2024, ex. Lymantriidae eggs, Xie Xin leg., FAFU-1237, 1295; 1♀(FAFU), Guizhou, Guizhou Province, China, 15–27.XI.2020, FAFU-1425; 3♀(FAFU), Shennongjia Forestry District, Hubei Province, China, VII.2023, Liu Meike & Che Jinting leg., FAFU-1500, 1507, 1509; 1♀(FAFU), Jiuniutang, Maoershan, Guangxi Province, China, 14.VII.2020, Li Tao leg., FAFU-1570; 4♀(FAFU), Shennongjia Forestry District, Guangxi Province, China, 11.IX.2022–15.X.2023, Liu Meike & Che Jinting leg., FAFU-1606, 1619, 1621, 1628; 1♀(FAFU), Badagongshan, Hunan Province, China, 31.V–14.VI.2020, FAFU-1573; 1♀(FAFU), Jianning County, Jinnao Mountain, Sanming City, Fujian Province, China, 27.VI.2014, 1520 m, Peng Lingfei leg., FAFU-58; 1♀(FAFU), Shaowu City, Longhu, Nanping, Fujian Province, China, IV.2015, Peng Lingfei leg., FAFU-100; 1♀(FAFU), Daiyunshan Houzhai Management Station 2, Dehua County, Quanzhou City, Fujian Province, China, 5.VII.2015, 850 m, FAFU-271; 3♀(FAFU), Longqishan Nature Reserve, Sanming City, Fujian Province, China, 5.VII.2018, 660 m, FAFU-746, 774, 775; 1♀(FAFU), Goudunping, Tianbaoyan Nature Reserve, Yong’an City, Sanming City, Fujian Province, China, 20.IX.2020, 1000 m, FAFU-821; 1♀(FAFU), Goudunping, Tianbaoyan Nature Reserve, Yong’an City, Sanming City, Fujian Province, China, 29.IX.2020, FAFU-846; 1♀(FAFU), Dashuikeng, Tianbaoyan Nature Reserve, Yong’an City, Sanming City, Fujian Province, China, 12.VII.2021, FAFU-887; 1♀(FAFU), Qingshui Station, Tianbaoyan Nature Reserve, Yong’an City, Sanming City, Fujian Province, China, Malaise trap, 21.IX.2020, 700 m, FAFU-893; 1♀(FAFU), Dashuikeng, Tianbaoyan Nature Reserve, Yong’an City, Sanming City, Fujian Province, China, 17.VI.2021, 1070 m, FAFU-899; 1♀(FAFU), Anhou, Tianbaoyan Nature Reserve, Sanming City, Fujian Province, China, 2024, FAFU-1292; 1♀(FAFU), Wangpingdong, Minjiangyuan Nature Reserve, Sanming City, Fujian Province, China, 2.IX.2024, FAFU-1407; 1♀(FAFU), Pinggangshang, Minjiangyuan Nature Reserve, Sanming City, Fujian Province, China, 30.VI–30.VII.2024, FAFU-1590; 1♀(FAFU), Wangpingdong, Minjiangyuan Nature Reserve, Sanming City, Fujian Province, China, 9–31.X.2024, FAFU-1597.

**Description.** See Peng et al. [4].

**Distribution.** China (ORIENTAL: Fujian, *Hunan, *Guangdong, *Guangxi, *Guizhou, *Jiangxi, *Zhejiang; PALAEARCTIC: Gansu, *Hebei, *Hubei, *Liaoning, *Shandong, *Shaanxi).

**Hosts.** Lepidoptera: Saturniidae: *Antheraea pernyi* and *Caligula japonica* [34]. New record from this study: Lymantriidae.

**Figure 24:**
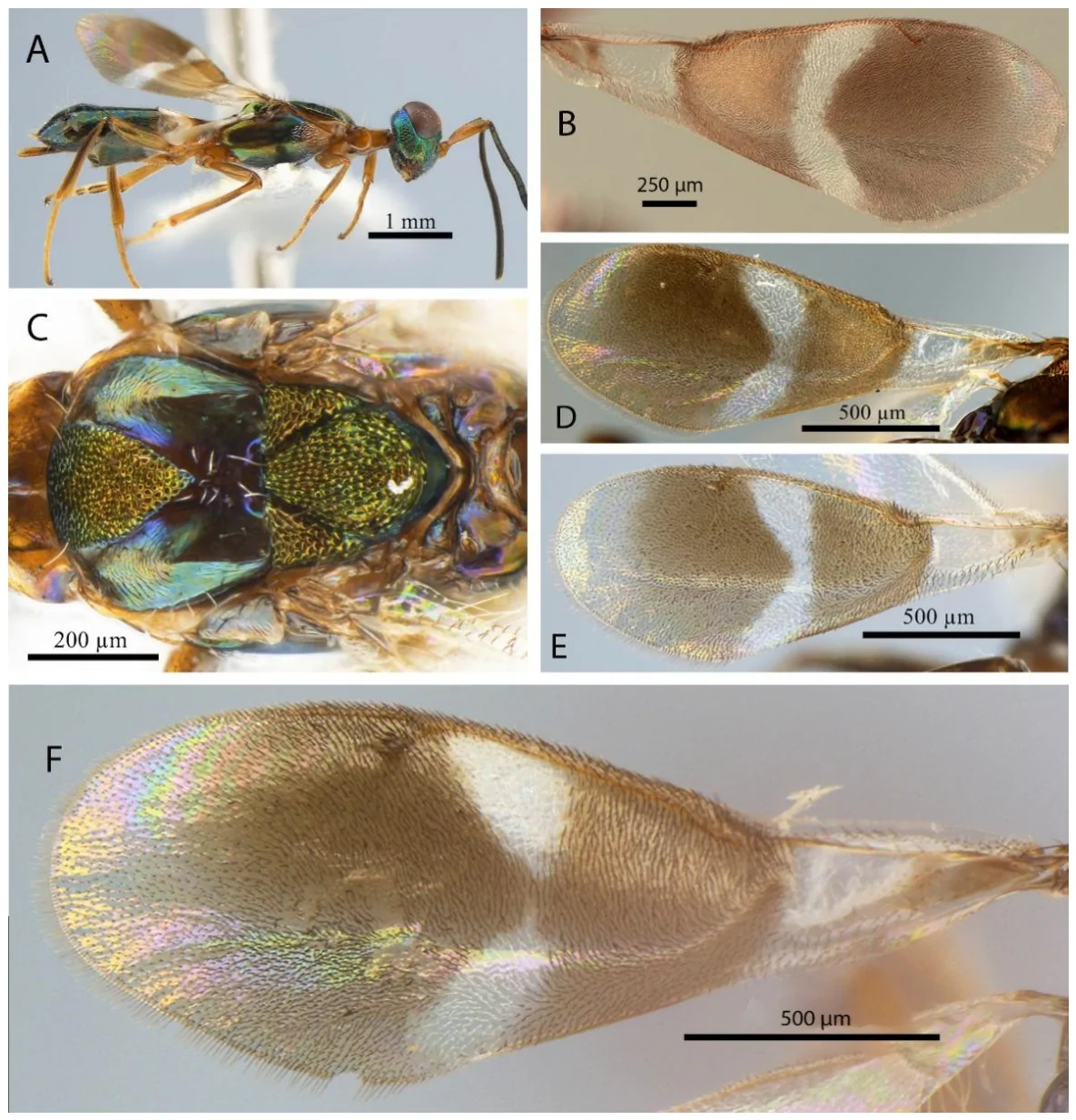
*Anastatus* (*Anastatus*) *gansuensis* ♀: (**A**) (FAFU-1598), body lateral; (**B**) fore wing; (**C**) (FAFU-1237), mesosoma; (**D**) (FAFU-692), fore wing; (**E**) (FAFU-1621), fore wing; (**F**) (FAFU-1500), fore wing.

**Remarks.** *A. gansuensis* is close to *A. kryptos*. The posterior concave lobe of the mesoscutum in *A. kryptos* (Figure 9C) is densely setose with white setae throughout, whereas in *A. gansuensis* it is not densely setose and white setae are restricted medially (Figure 24C). The fore wing hyaline crossband is broader in *A. kryptos* (Figure 9H), whereas in *A. gansuensis* it is sometimes constricted or appears as two spots (Figure 24B,D–F). See the remarks section of *A. kryptos* for the detailed morphometric analysis of the fore wing hyaline crossband width ratio (Figure 10).

*A. gansuensis* is also very close to *A. catalonicus*, as explained below:

The original description of *A. catalonicus* was based on a single female collected in Gerona, Spain [35]. The holotype was deposited in the Museo Nacional de Ciencias Naturales (MNCN), Madrid, but subsequent searches have failed to locate it, and the specimen is now considered lost [36,37].

*A. catalonicus* was originally known from Spain (mainland, the Balearic and the Canary Islands), and later from France, Germany, Bulgaria, and Romania [36,37]. The species has since been recorded from Iran (East Azerbaijan) by Lotfalizadeh & Parsa [33], and from Poland (Western Beskid) by Wiśniowski [32], establishing a broad west-to-east Palaearctic distribution [32,33].

In contrast, *A. gansuensis* was originally described from Gansu Province in China [34]. Li et al. [3] have recorded the species from Fujian Province in southeastern China, more than 1000 km southeast of the type locality, spanning both Palaearctic and Oriental regions. The authors explicitly confirmed that this wide distribution, combined with the broad range of its host *Caligula japonica* across eastern Asia, suggests the species may be more widespread than currently known.

*A. catalonicus* is documented as a primary parasitoid of *Lymantria dispar* (Lepidoptera: Lymantriidae) and *Iris oratoria* (Mantodea: Mantidae), and as an associate of the cynipid gall-wasp *Biorhiza pallida* [36,37].

*A. gansuensis* was reared as a solitary endoparasitoid of *Caligula japonica* Moore (Lepidoptera: Saturniidae) and cultured successfully on *Antheraea pernyi* (Guérin-Méneville) (Saturniidae) in the laboratory [34]. Additional examined material confirms parasitism of Lymantriidae eggs as a new host record from China in this study. Therefore, both taxa are generalist egg parasitoids attacking the eggs of large Lepidoptera and mantids.

The original description of *A. gansuensis* emphasized a fore wing with a single hyaline crossband behind the marginal vein, “occasionally with a few isolated dark setae within the band medially” [4,34]. However, Li et al. [3] examined four females from Fujian and found that “the dark setae within the band are denser and more distinct,” creating two hyaline spots (a pattern that was previously considered characteristic only of *A. echidna* (Motschulsky) among the Chinese species). The authors explicitly state: “This is important to note because the presence of dark setae creates two hyaline spots.” This observation is critically significant because the fore wing of *A. catalonicus* also has a “white transverse band strongly constricted medially, occasionally with dark setae medially” [36]. The “two hyaline spots” observed in Fujian specimens of *A. gansuensis* directly match those of the *A. catalonicus* pattern.

The ML tree showed that all sequences of *A. gansuensis* from China, including BOLD sequences from different countries, clustered in one clade, confirming them as a monophyletic clade with intraspecific similarity of 95.4%. Six representative sequences were selected: three from Bulgaria (GMBUA1120-14, GMBSV699-18, GMBSR1276-18), two from Portugal (LPSYA1994-23, LPSYA1969-23), and one from Germany (AMTPA267-15). This confirms that the geographic overlapping based on morphology was not a coincidence.

Despite these confirmations, no definitive morphological evidence was found for *A. catalonicus* from Spain, and BOLD sequences were not correctly identified as *A. catalonicus*. We suspect that *A. gansuensis* may also occur in Europe, a hypothesis that requires further clarifications. Therefore, based on this conflict, we conclude that these two species are very close based on different aspects (geographically, biologically, and morphologically), but additional evidence is needed before they can be synonymized.

#### 3.4.10. *Anastatus* (*Anastatus*) *garygibsoni* Zhou & Peng, 2024 (Figure 25)

*Anastatus garygibsoni*, Wang et al., 2024: 11–12, fig. 6.

**Type material examined. Holotype.** ♀(FAFU), Longmen Road Orchard, Danzhou, Hainan Province, China, 23.III.2017, 140 m, FAFU-173. **Paratypes.** 2♀(FAFU), same locality, 22.III.2017, 140 m, FAFU-225, 226.

**Non-type material examined.** 2♀(FAFU), Wuzhishan, Hainan Province, China, 28.III.2021, FAFU-830, 831; 1♀(FAFU), Chebaling Nature Reserve, Shixing, Shaoguan, Guangdong Province, China, 1–11.X.2022, FAFU-1261; 1♀(FAFU), Special Plant Garden, Xishuangbanna Botanical Garden, Yunnan Province, China, 562 m, 2024, Nong Qiaoyi leg., FAFU-1379; 1♀(FAFU), Tropical Rainforest, Xishuangbanna Botanical Garden, Yunnan Province, China, 600 m, 2024, Nong Qiaoyi leg., FAFU-1380; 2♀(FAFU), Baihuayuan Garden, Xishuangbanna Botanical Garden, Yunnan Province, China, 543 m, 15–30.VIII.2024, Nong Qiaoyi leg., FAFU-1381, 1410; 1♀(FAFU), Celebrity Tree Garden, Xishuangbanna Botanical Garden, Yunnan Province, China, 15–30.IX.2024, Nong Jieyi leg., FAFU-1423; 1♀(FAFU), Zhao’qing, Guangdong Province, China, 12.IX.2025, Wenting Chen leg., FAFU-1742.

**Description.** See Wang et al. [1].

**Distribution.** ORIENTAL: China (Hainan, *Guangdong, *Yunnan).

**Host.** Hemiptera: Tessaratomidae: Eggs of *Tessaratoma papillosa* (Drury) [1].

**Figure 25:**
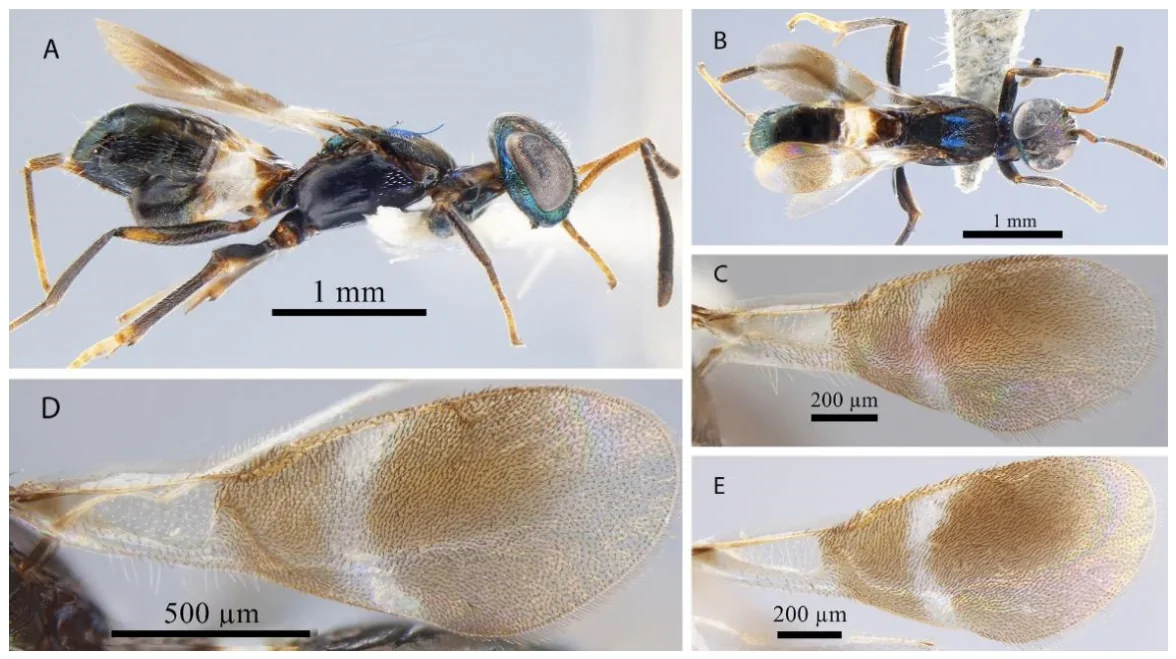
*Anastatus* (*Anastatus*) *garygibsoni* ♀: (**A**,**B**) (FAFU-1261) (**A**) body lateral; (**B**) body dorsal; (**C**) (FAFU-1380), fore wing; (**D**) (FAFU-1261), fore wing; (**E**) (FAFU-1410), fore wing.

**Remarks.** *A. garygibsoni* is close to *A. dexingensis* and *A. formosanus* based on the profemoral tooth. However, the relative measurements of the fore wing ratio (the width of infuscate region behind the parastigma relative to the hyaline crossband) distinguished *A. garygibsoni* from the other two species, as it has the highest ratio, ranging from 5.7× to 9.1× ($\bar{x}$ = 7.41). This distinction is also supported by morphology (Figure 25).

**Figure 26:**
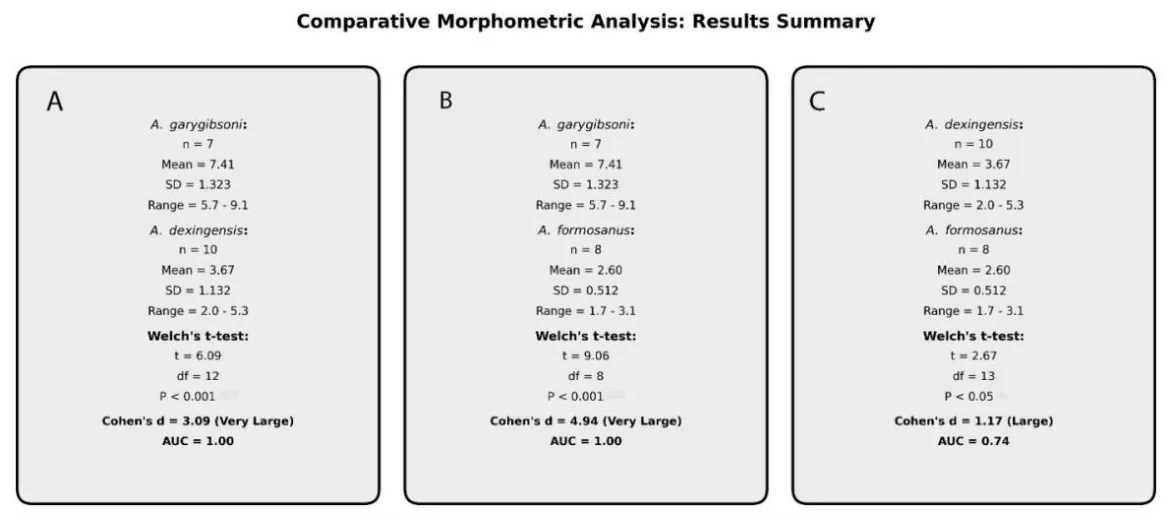
Results summary of pairwise Welch’s *t*-test comparisons for the ratio (the width of the infuscate region posterior to the parastigma relative to the width of the hyaline crossband). (**A**) *A. garygibsoni* vs. *A. dexingensis*; (**B**) *A. garygibsoni* vs. *A. formosanus*; (**C**) *A. dexingensis* vs. *A. formosanus*. These results separate *A. garygibsoni* from *A. dexingensis* and *A. formosanus*, while *A. formosanus* and *A. dexingensis* still overlap.

In contrast, in *A. dexingensis* and *A. formosanus*, the maximum ratio is below 5.3× ($\bar{x}$ = 3.60 and $\bar{x}$ = 2.60, respectively). A total of 25 specimens were examined, confirming that the ratio of the infuscate region posterior to the parastigma relative to the width of the hyaline crossband is the most informative feature for separating *A. garygibsoni* from *A. dexingensis* and *A. formosanus*. *A. garygibsoni* and *A. dexingensis* are significantly different (*p* < 0.001), with *A. garygibsoni* having a much higher ratio ($\bar{x}$ = 7.41, range 5.7 to 9.1×); *A. garygibsoni* and *A. formosanus* are also significantly different (*p* < 0.001). *A. dexingensis* and *A. formosanus* are significantly different (*p* < 0.05), but show partial overlap in measurements, and the AUC value (0.74) indicates imperfect discrimination between these two species (Figure 26).

In contrast, *A. formosanus* displayed the highest degree of character stability and the lowest mean ratio ($\bar{x}$ = 2.60). The specimens showed a narrow range of 1.67× to 3.1×, indicative of a broad, consistently clear hyaline crossband with no dark setae within the hyaline region. Moreover, *A. dexingensis* exhibited the broadest range of intraspecific variation ($\bar{x}$ = 3.67; range 2.0× to 5.3×). This plasticity is directly attributable to the inconsistent density of medial setae. While some specimens (such as FAFU-1309, and FAFU-1511) displayed a clear crossband resembling *A. formosanus* phenotype, others (e.g., FAFU-1599, ratio 5.3×) showed some dark setal presence in the hyaline crossband medially, approaching the lower limits of the ratio.

**Figure 27:**
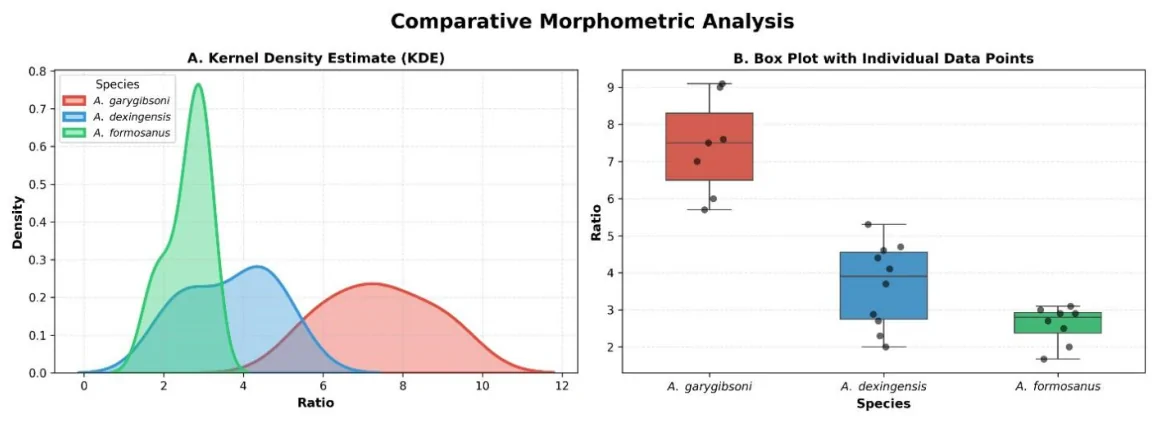
Comparative morphometric analysis of the ratio (the width of the infuscate region posterior to the parastigma relative to the width of the hyaline crossband) in *A. garygibsoni*, *A. dexingensis*, and *A. formosanus*. (**A**) Kernel density estimate (KDE) plot visualizing the separation of species in morphological space. Although the KDE smoothing bandwidth creates an apparent region of overlap between *A. garygibsoni* and *A. dexingensis*, the actual measured values do not overlap, as show in the results summary (Figure 26); (**B**) Boxplot showing the median, interquartile range (IQR), and full range of ratios, highlighting the diagnostic gap between species.

Furthermore, Kernel density estimates (KDE) and boxplots place *A. garygibsoni* in a distinct morphological cluster (Figure 27A,B). 

This analysis clarified that the numerous dark setae in the hyaline crossband of *A. garygibsoni* cause the crossband to appear interrupted, or to sometimes appear as two spots which are absent in the other two species.

#### 3.4.11. *Anastatus* (*Anastatus*) *gastropachae* Ashmead, 1904 (Figure 28 and Figure 29)

*Anastatus gastropachae* Ashmead, 1904: 153–154. Described: female.

*Anastatus bifasciatus* (Geoffroy); Ishii, 1938: 98–99 (incorrect synonymy).

*Anastatus gastropachae*; Kalina, 1981: 16, fig. 41; Sheng and Yu, 1998: 6, 8; Yang et al., 2015b: 161, 255, fig. 83.

*Anastatus huangi* Sheng and Yu, 1998: 5–7, 8, fig. 2(1–5). Described: both sexes.

*Anastatus huangi*; Peng et al., 2017: 14–16, figs 28–33.

*Anastatus* (*Anastatus*) *gastropachae*, Peng et al., 2020: 379–381, fig. 14.

*Anastatus flaviratus* Peng et al., 2020: syn. nov.

*Anastatus* (*Anastatus*) *gastropachae*; Li et al., 2025: 353–354, figs. 1A, 1B.

**Type material examined.** 3♀(FAFU), Chebaling, Shixing County, Shaoguan, Guangdong Province, China, 25.V.2002, Liu Changming leg., FAFU-169–171.

**Non-type material examined.** 1♀(FAFU), Lianghekou, Emeishan, Leshan, Sichuan Province, China, 605 m, 10.VIII.2014, Liu Qifei leg., FAFU-43; 1♀(FAFU), Guilongshan, Changting, Longyan, Fujian Province, China, 17.VI.2012, Peng Lingfei leg., FAFU-57; 1♀(FAFU), Tingjiangyuan Nature Reserve, Changting, Longyan, Fujian Province, China, 27.VI.2012, Peng Lingfei leg., FAFU-72; 2♀(FAFU), Minqing, Fuzhou, Fujian Province, China, 10.IV.2018, ex. eggs of *Dendrolimus punctatus*, FAFU-301, 302; 1♀(FAFU), Minqing, Fuzhou, Fujian Province, China, 18.VIII.2005, Liu Changming leg., FAFU-307; 2♀(FAFU), Yongtai, Fuzhou, Fujian Province, China, 11–24.IV.2018, ex. eggs of *Dendrolimus punctatus*, FAFU-375, 376; 1♀(FAFU), Jiufeng Village, Shoushan, Fuzhou, Fujian Province, China, 28.V.2024, Li Zixuan leg., FAFU-1082; 3♀(FAFU), Luoyuan, Fuzhou, Fujian Province, China, ex. *Dendrolimus* sp. eggs, FAFU-1084, 1135, 1136; 1♀(FAFU), Longhu, Nanping, Fujian Province, China, 26.VIII.2018, FAFU-1287; 1♀(FAFU), Wuyishan, Nanping, Fujian Province, China, 1–8.VI.2021, FAFU-865; 1♀(FAFU), Wuyishan Da’anyuan, Nanping, Fujian Province, China, 2021, FAFU-952; 1♀(FAFU), Chengdun, Wuyishan, Nanping, Fujian Province, China, 27.VI.2022, FAFU-996; 2♀(FAFU), Huangxizhou, Wuyishan, Nanping, Fujian Province, China, 17.IX.2022, FAFU-1346, 1347; 1♀(FAFU), Tianbaoyan Nature Reserve, Anhou, Yong’an, Sanming, Fujian Province, China, 14.VI.2021, FAFU-927; 1♀(FAFU), Tianbaoyan Nature Reserve, Anhou, Yong’an, Sanming, Fujian Province, China, 6.VII.2021, FAFU-1288; 1♀(FAFU), Chebaling, Shixing, Shaoguan, Guangdong Province, China, 21–23.VIII.2003, FAFU-308; 2♀(FAFU), Nanling Nature Reserve, Ruyuan, Shaoguan, Guangdong Province, China, 23.VII.2003, FAFU-326, 330; 1♀(FAFU), Yangshan, Qingyuan, Chengjia Nature Reserve, Guangdong Province, China, 7–27.VI.2021, FAFU-980; 1♀(FAFU), Chebaling Nature Reserve, Shixing, Shaoguan, Guangdong Province, China, 7–21.VI.2021, FAFU-1004; 2♀(FAFU), Chebaling Nature Reserve, Shixing, Shaoguan, Guangdong Province, China, 25.X–10.XI.2020, FAFU-1011, 1012; 1♀(FAFU), Nanling Nature Reserve, Ruyuan, Shaoguan, Guangdong Province, China, 28.X–25.XI.2020, FAFU-1185; 1♀(FAFU), Nanling National Nature Reserve, Ruyuan Yao Autonomous County, Shaoguan, Guangdong Province, China, Malaise trap, 22.IX–13.X.2020, FAFU-1216; 1♀(FAFU), Nanling National Nature Reserve, Ruyuan Yao Autonomous County, Shaoguan, Guangdong Province, China, Malaise trap, 22.IX–13.X.2020, FAFU-1217; 1♀(FAFU), Nanling National Nature Reserve, Shaoguan, Guangdong Province, China, Malaise trap, 9.IV–28.V.2021, FAFU-1225; 2♀(FAFU), same locality, Guangdong Province, China, Malaise trap, 28.X–25.XI.2020, FAFU-1233, 1235; 1♀(FAFU), Nanling National Forest Park, Shaoguan, Guangdong Province, China, FAFU-1144; 1♀(FAFU), Nanling National Nature Reserve, Shaoguan, Guangdong Province, China, 29.X–29.XI.2021, FAFU-1268; 1♀(FAFU), Nanling National Nature Reserve, Shaoguan, Guangdong Province, China, Malaise trap, 22.IX–13.X.2020, FAFU-1269; 1♀(FAFU), Nanling National Nature Reserve, Shaoguan, Guangdong Province, China, Malaise trap, 9.IV–28.V.2021, FAFU-1283; 1♀(FAFU), Nanling National Forest Park mixed forest, Shaoguan, Guangdong Province, China, 23.VI–29.VII.2022, Li Wenfeng leg., FAFU-1284; 1♀(FAFU), Nanling National Nature Reserve, Shaoguan, Guangdong Province, China, 9.VI.2020, FAFU-1349; 1♀(FAFU), Nanling National Nature Reserve, Shaoguan, Guangdong Province, China, 25.VIII–15.IX.2020, FAFU-1350; 2♀(FAFU), Nanling National Forest Park mixed forest, Shaoguan, Guangdong Province, China, 9.V–10.VI.2023, Li Wenfeng leg., FAFU-1351, 1352; 5♀(FAFU), Nanling National Nature Reserve, Shaoguan, Guangdong Province, China, 9.IV–28.V.2021, FAFU-1355–1359; 1♀(FAFU), Nanling National Nature Reserve, Shaoguan, Guangdong Province, China, 26.VI–28.IX.2021, FAFU-1360; 2♀(FAFU), Nanling National Nature Reserve, Shaoguan, Guangdong Province, China, 25.VIII–15.IX.2020, FAFU-1362, FAFU-1363; 1♀(FAFU), Fengyangshan, Longquan, Lishui, Zhejiang Province, China, 7–10.VIII.2003, FAFU-334; 1♀(FAFU), Tianmushan, Lin’an, Hangzhou, Zhejiang Province, China, 4.V.2014, FAFU-396; 1♀(FAFU), Tianmushan, Lin’an, Hangzhou, Zhejiang Province, China, 19.V.2015, FAFU-460; 1♀(FAFU), Simingshan, Yuyao, Ningbo, Zhejiang Province, China, 5.VI.2017, FAFU-423; 2♀(FAFU), Jinyunshan, Chongqing Municipality, China, 10.IX.2021, FAFU-953, 982; 2♀(FAFU), Jinyunshan TV Tower, Chongqing Municipality, China, 25.XI.2021, FAFU-986, 987; 1♀(FAFU), Jiulianshan Nature Reserve, Longnan, Ganzhou, Jiangxi Province, China, 23.IV–7.V.2022, FAFU-988; 1♀(FAFU), Xingangshan, Jiangxi Province, China, 20.VI.2018, FAFU-1353; 1♀(FAFU), Xingangshan, Jiangxi Province, China, 30.VIII.2022, FAFU-1053; 1♀(FAFU), Fujian Province, China, 5.IX–6.X.2023, FAFU-1145; 2♀(FAFU), Meiling, Nanchang, Jiangxi Province, China, 1.VII.2025, and 15.XI.2025, Du Jingzhe leg., FAFU-1643, 1644.

**Description.** See Peng et al. [4].

**Distribution.** ORIENTAL: China (Fujian, Hunan, Jiangxi, Zhejiang, Taiwan, *Guangdong, *Sichuan, *Chongqing), Japan, South Korea.

**Hosts.** Hemiptera: Pentatomidae: *Halyomorpha halys*; Lepidoptera: Lasiocampidae: *Dendrolimus houi*, *D. kikuchii*, *D. punctatus*, and *D. spectabilis* [4].

**Figure 28:**
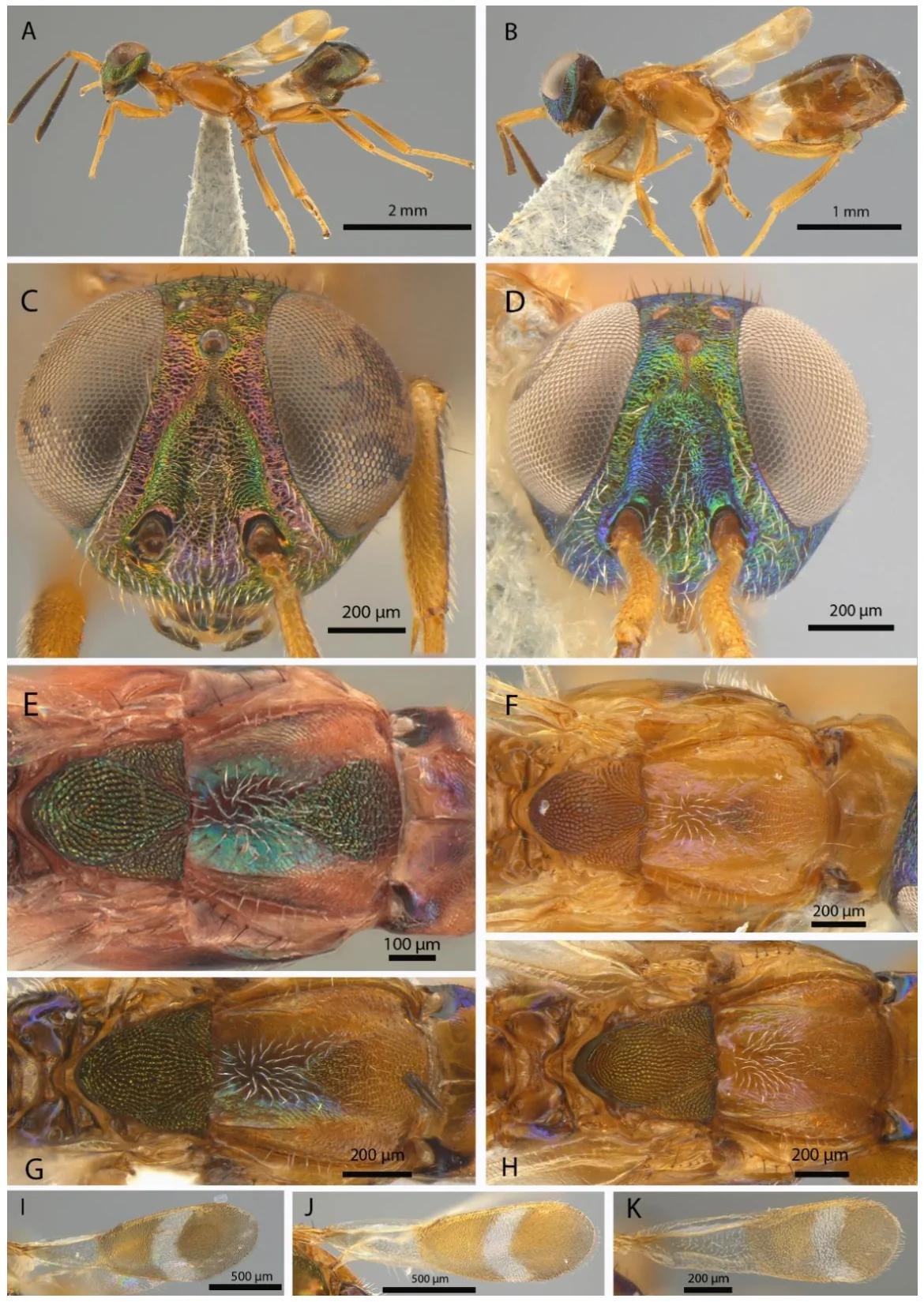
*Anastatus* (*Anastatus*) *gastropachae* ♀: (**A**) body lateral (FAFU-1359); (**B**) body lateral (FAFU-988); (**C**) head front; (**D**) head front (FAFU-988); (**E**) mesosoma; (**F**) mesosoma (FAFU-988); (**G**) mesosoma (FAFU-1351); (**H**) mesosoma (FAFU-1359); (**I**) fore wing (FAFU-1351); (**J**) fore wing (FAFU-1350); (**K**) fore wing (FAFU-988).

**Figure 29:**
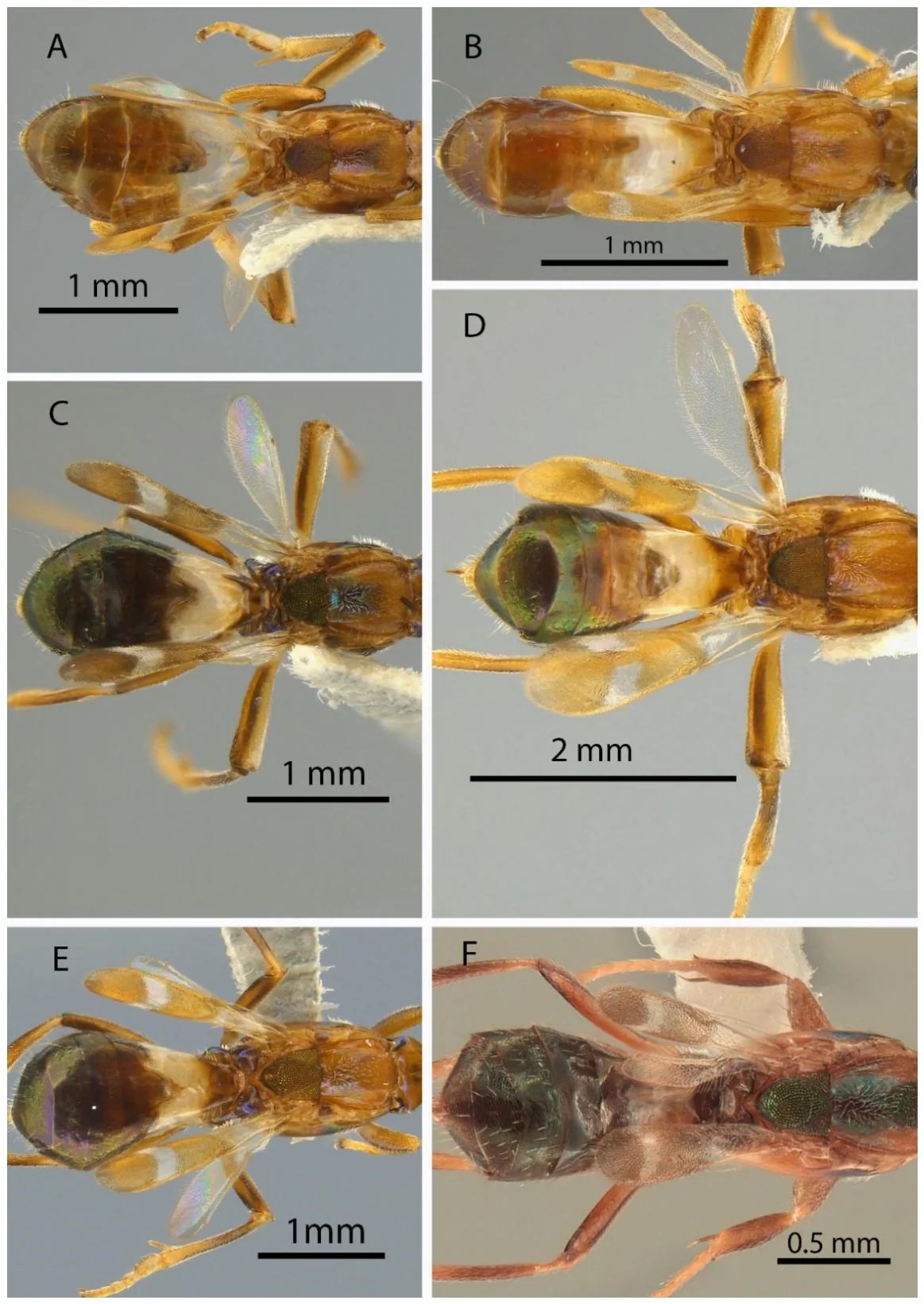
*Anastatus* (*Anastatus*) *gastropachae* ♀: (**A**) body dorsal (FAFU-980); (**B**) body dorsal (FAFU-988); (**C**) body dorsal (FAFU-1351); (**D**) body dorsal (FAFU-1359); (**E**) body dorsal (FAFU-1350); (**F**) body dorsal.

**Remarks.** After careful observation of approximately 67 females from different provinces of China, we found extensive variation in the morphology of *A. gastropachae*. This species exhibits three fore wing forms, ranging from brachypterous to medium-large (not fully developed macropterous) with intermediate forms (Figure 28A,B,I–K and Figure 29). Body colour ranges from pale yellow to dark. The head is mostly bright bluish green with the vertex, gena and occiput metallic bluish-green to bright green (Figure 28D), but sometimes greenish to violet purple in frontal view (Figure 28C). The colouration of mesoscutum varies among females from non-metallic to medium metallic to fully metallic lustre. The mesoscutum, scutellum, and axillae range from lacking metallic lustre to exhibiting strong green to bluish metallic lustre (Figure 28E–H), as noticed by Li et al. [3]. The gaster is mostly pale brown to dark with metallic lustre under some angles of light, and the base is partly yellowish-white (Figure 29). After careful morphological examination including the type material (FAFU-169, 170), we selected some previously identified females of *A. flaviratus* and, based on the availability of DNA, performed PCR and *COI* sequencing on three specimens (FAFU-980, FAFU-988, FAFU-996). These sequences nested within the *A. gastropachae* clade in the ML tree, also confirmed *A. flaviratus* as a new synonymy of *A. gastropachae*.

#### 3.4.12. *Anastatus* (*Anastatus*) *japonicus* Ashmead, 1904 (Figure 30)

*Anastatus japonicus* Ashmead, 1904: 153. Described: both sexes.

*Anastatus japonicus*; Kalina, 1981: 17–19, plate 1, fig. 6; Yang et al., 2015b: 163–164, 255, fig. 84.

*Anastatus* (*Anastatus*) *japonicus*; Narendran, 2009: 82, figs 26, 27.

*Anastatus* (*Anastatus*) *japonicus*; Chen et al., 2019: 126–128, fig. 5.

*Anastatus bifasciatus* disparis Ruschka, 1921: 265. Synonymy by Bouček, 1977: 124–124.

*Anastatus disparis*; Burgess, 1929: 574 (new status).

*Anastatus flavipes* Sheng and Wang, in Sheng et al., 1997: 6–7 (Chinese description). 8 (English abstract), figs 10–13.

*Anastatus dendrolimus*; Xiao et al., 2001: 204. Misidentification.

*Anastatus colemani*; Hu et al., 2011: 483, fig. 2. Misidentification.

*Anastatus tenuipes*; Hu et al., 2011: 483–484. Misidentification.

*Anastatus flavipes*; Peng et al., 2017: 7–10, figs 8–18.

*Anastatus* (*Anastatus*) *japonicus,* Peng et al., 2020: 381–387, figs 15–17. Described: both sexes.

*Anastatus* (*Anastatus*) *japonicus*, Li et al., 2025: 354, figs. 3A, 3B.

**Material examined.** 1♀(FAFU), Tianmushan, Lin’an, Hangzhou, Zhejiang Province, China, 30.VI.2014, He Sunqiang leg., FAFU-26; 2♀(FAFU), same location, 21.IV.2014, ex, eggs of *Notobitus meleagris*, He Sunqiang leg., FAFU-28, 29; 1♀(FAFU), same location, 30.VI.2014, FAFU-137; 1♀(FAFU), Jinggangshan, Jiangxi Province, China, 29.VII.2014, 1360 m, Liu Qifei leg., FAFU-38; 3♀(FAFU), Baiwangshan, Haidian, Beijing, China, VII.2015, ex. eggs of *Cappaea tibialis*, FAFU-153, 154, 155; 2♀(FAFU), Lengquan Village, Haidian, Beijing, China, VII.2014, ex. eggs of *Dolycoris baccarum* and *Halyomorpha halys*, FAFU-156, 157; 2♀(FAFU), Shengshuyuan, Haidian, Beijing, China, 7.VII.2013, ex. eggs of *Halyomorpha halys*, FAFU-158, 159; 1♀(FAFU), Langfang, Hebei Province, China, 26.VII.2013, FAFU-160; 1♀(FAFU), Xishan, Haidian, Beijing, China, VIII.2014, FAFU-161; 1♀(FAFU), Baiwangshan, Haidian, Beijing, China, VIII.2014, FAFU-162; 1♀(FAFU), Lengquan Village, Haidian, Beijing, China, 2013, ex. eggs of *Plautia fimbriata*, FAFU-163; 2♀(FAFU), Shengshuyuan, Haidian, Beijing, China, 2013, ex. eggs of *Plautia fimbriata*, FAFU-164, 165; 1♀(FAFU), National Chung Hsing University, Taichung, TaiwanProvince, China, 30.VII.2017, FAFU-246; 3♀(FAFU), Changchun, Jilin Province, China, VI–VII.2017, ex. eggs of *Antheraea pernyi*, Chen Yongming leg., FAFU-259, 261, 262; 2♀(FAFU), Kangxian, Longnan, Gansu Province, China, 23.I.2018, ex. eggs of *Dictyoploca japonica*, Chen Yongming leg., FAFU-461, 462; 1♀(FAFU), Ninghua, Sanming, Fujian Province, China, 15.VII.2017, Peng Lingfei leg., FAFU-514; 1♀(FAFU), Longqishan Nature Reserve, Sanming, Fujian Province, China, 18.V.2018, 740 m, FAFU-516; 3♀(FAFU), Fu County, Yan’an, Shaanxi Province, China, 8.VIII–3.IX.2018, FAFU-664, 736, 738; 1♀(FAFU), Chebaling, Guangdong Province, China, 10–20.II.2022, FAFU-1054; 2♀(FAFU), National Chung Hsing University, Taichung, Taiwan Province, China, 30.VII.2017, FAFU-1154, 1155; 1♀(FAFU), Guizhou, Guizhou Province, China, 10–21.XI.2020, FAFU-1274; 2♀(FAFU), Funiushan, Luoyang, Henan Province, China, 21.IX.2023, FAFU-1366, 1370; 1♀(FAFU), Tianjin, China, I.2023, FAFU-1371; 1♀(FAFU), Benxi, Liaoning Province, China, 26.VII.2023, FAFU-1463; 1♀(FAFU), Dalian, Liaoning Province, China, 30.VII.2023, FAFU-1602; 1♀(FAFU), Pinggu District, Dongchangyu, Beijing, China, 6.VI.2018, Guoyue Yu leg., FAFU-1746.

**Description.** See Peng et al. [4].

**Distribution.** PALAEARCTIC: China (Beijing, Gansu, Hebei, Heilongjiang, *Henan, Jilin, Liaoning, Shaanxi, Shandong, Shanxi, *Tianjin); ORIENTAL: China (Anhui, Fujian, Guangdong, Guangxi, *Guizhou, Hainan, Hong Kong, *Hunan, Jiangsu, Jiangxi, Taiwan, Zhejiang).

**Hosts.** Hemiptera (Alydidae, Pentatomidae: *Plautia fimbriata*, *Cappaea tibialis*, *Dolycoris baccarum*, *Halyomorpha halys*, *Menida violacea*, and *Plautia crossota*); Lepidoptera (Lasiocampidae, Lymantriidae, Notodontidae, Papilionidae, Saturniidae); hyperparasitoid (Braconidae and Encyrtidae) [4]. New host records from this study: *Notobitus meleagris* (Alydidae: Hemiptera) and *Antheraea pernyi* (Saturniidae: Lepidoptera).

**Remarks.** *A. japonicus* is morphologically similar to *A. nozdormu*, *A. beaglei*, and *A. yalini*. See the remarks sections of these species for further details.

**Figure 30:**
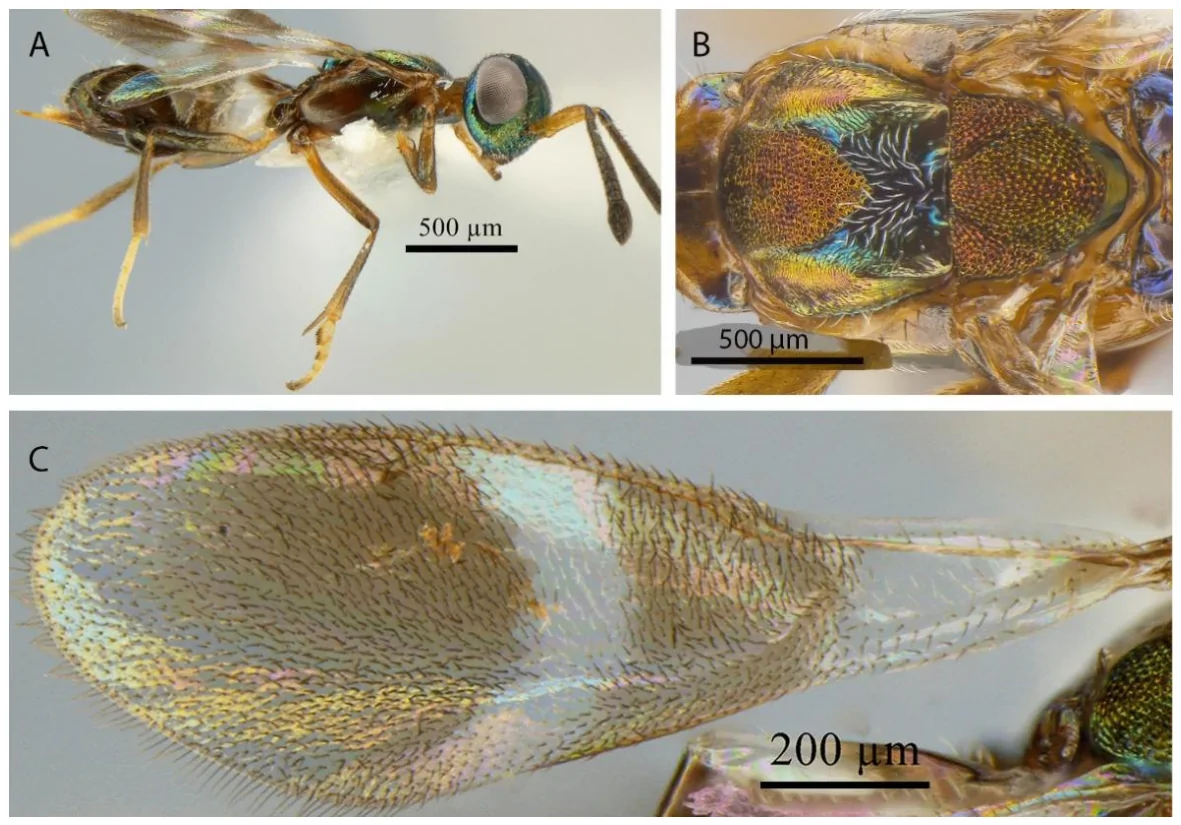
*Anastatus* (*Anastatus*) *japonicus* ♀: (**A**) (FAFU-1602), body lateral; (**B**) (FAFU-1370), mesosoma; (**C**) (FAFU-1602), fore wing.

#### 3.4.13. *Anastatus* (*Anastatus*) *makrysourus* Zhou & Peng, 2024 (Figure 31)

*Anastatus makrysourus*, Wang et al., 2024: 12–14, fig. 7.

**Material examined.** 1♀(FAFU), Longqishan Nature Reserve, Sanming, Fujian Province, China, 5.VII.2018, FAFU-777; 1♀(FAFU), Zhangjiangkou Mangrove Base Boardwalk, Yunxiao, Zhangzhou, Fujian Province, China, 22.IX.2023, FAFU-1228; 1♀(FAFU), Futian Mangrove Ecological Park, Futian District, Shenzhen, Guangdong Province, China, 1.IX.2020, Li Kunyuan leg., FAFU-1303; 1♀(FAFU), Xinglong Medicinal Botanical Garden (coconut forest), Wanning, Hainan Province, China, 15–31.VIII.2020, FAFU-1408; 1♀(FAFU), Celebrity Tree Garden, Xishuangbanna Botanical Garden, Xishuangbanna, Yunnan Province, China, 15–30.IX.2024, Nong Jieyi leg., FAFU-1498; 1♀(FAFU), *Camellia oleifera* forest, Chunwan Town, Yangchun, Guangdong Province, China, 379 m, 3.III–18.IV.2025, Han Zhang leg., FAFU-1736.

**Description.** See Wang et al. [1].

**Distribution.** ORIENTAL: China (Hainan, *Fujian, *Guangdong, *Yunnan).

**Host.** Unknown.

**Figure 31:**
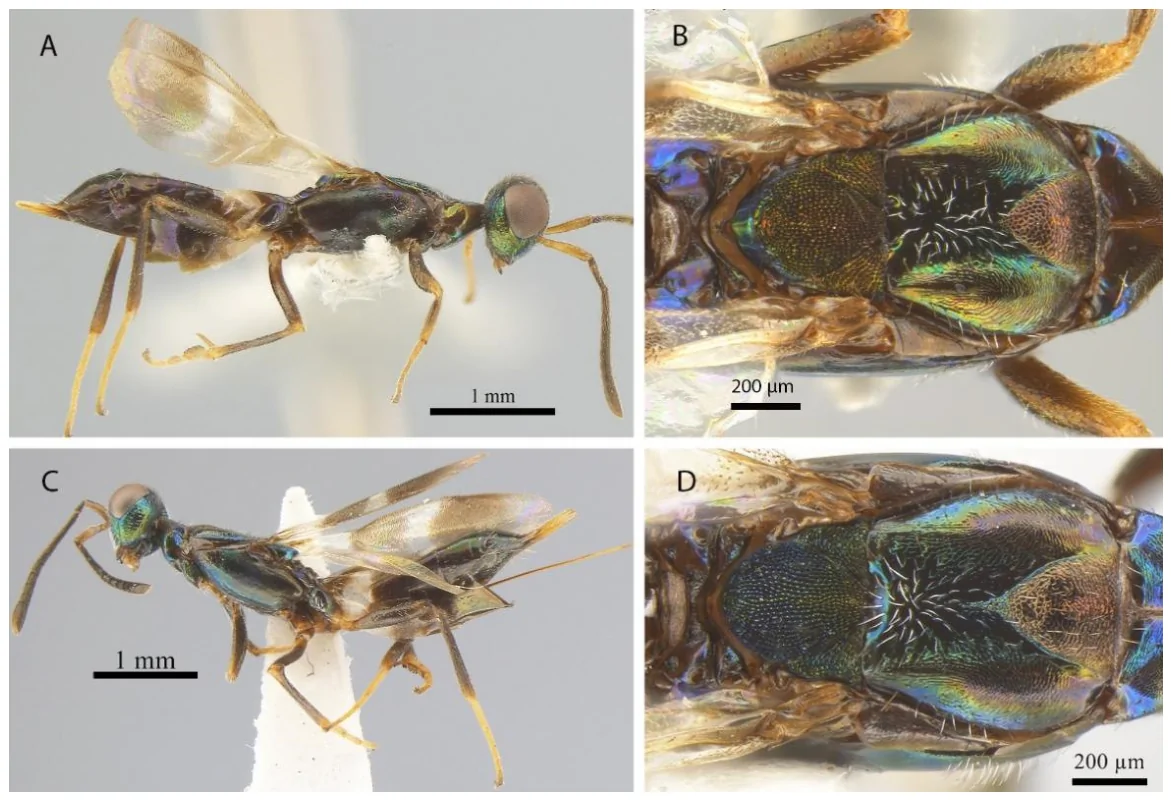
*Anastatus* (*Anastatus*) *makrysourus* ♀: (**A**,**B**) (FAFU-1303) (**A**) body lateral; (**B**) mesosoma (**C**,**D**) (FAFU-777) (**C**) body lateral; (**D**) mesosoma.

**Remarks.** *A. makrysourus* is unique among Chinese *A*. (*Anastatus*) species in having longer ovipositor sheaths. However, it exhibits intraspecific variations in ovipositor size, ranging from longer (Figure 31C) to medium (Figure 31A), as well as in mesoscutum colouration (Figure 31B,D), although these variations are inconsistent across specimens. Moreover, we have only four females from China and two from Thailand (THAMG333-23, THAMG12611-23) with *COI* data. The ASAP results indicated that *A. makrysourus* may comprise two putative MOTUs/lineages with an intraspecific genetic divergence of 7.3%. At this point, there are insufficient specimens for comprehensive morphological assessment. Hence, based on the monophyletic clade of this species in the ML tree, and inconsistent morphological characters, we still consider it as a single species.

#### 3.4.14. *Anastatus* (*Anastatus*) *meilingensis* Sheng & Yu, 1998 (Figure 32)

*Anastatus* (*Anastatus*) *meilingensis* Sheng and Yu, 1998: 5–6 (Chinese description), 8 (English abstract), fig. 1. Described: both sexes.

*Anastatus meilingensis*; Peng et al., 2017: 16–18, figs 34–40.

*Anastatus* (*Anastatus*) *meilingensis*; Chen et al., 2019: 128–130, fig. 6.

*Anastatus* (*Anastatus*) *meilingensis*, Peng et al., 2020: 387–389, figs. 18,19.

**Material examined.** 1♀(FAFU), Yashushan, Ninghua, Sanming, Fujian Province, China, 5.I–13.I.2017, FAFU-266; 1♀(FAFU), Minqing, Fuzhou, Fujian Province, China, 18.VIII.2005, Changming Liu leg., FAFU-305; 1♀(FAFU), Rubber Plantation Research Institute, Jinghong, Xishuangbanna, Yunnan Province, China, 30.VII.2003, FAFU-317; 3♀(FAFU), Chenliangshe Village, Tiantai, Kangxian, Longnan Gansu Province, China, 23.I.2018, ex. eggs of *Antheraea pernyi* (ginkgo silkworm), Yongming Chen leg., FAFU-466-468; 1♀(FAFU), Jinyun Mountain, Chongqing, China, 10.IX.2021, FAFU-992; 1♀(FAFU), Sanlingshan Forest Park, Zhanjiang, Guangdong Province, China, 17.VI.2024, Zhenhua Liu leg., FAFU-1270; 1♀(FAFU), Xiyuangong, Fuzhou, Fujian Province, China, 7.VIII.2025, ex ootheca of mantis (parasitizing), Yi You leg., FAFU-1302; 1♀(FAFU), Jianfengling, Hainan Province, China, 30.X–30.XI.2019, Chunyang Xu leg., FAFU-1579; 1♀(FAFU), Luoyuan, Fuzhou, Fujian Province, China, IV.2024, ex eggs of *Dendrolimus* sp. on *Cryptomeria fortunei*, FAFU-1147; 1♀(FAFU), Huboliao Nature Reserve, Nanjing, Zhangzhou, Fujian Province, China, 300 m, 2018, FAFU-1289; 3♀(FAFU), Guizhou University laboratory culture, Guizhou Province, China, VII.2025, Haoran Liao leg., FAFU-1737, 1738, 1739.

**Figure 32:**
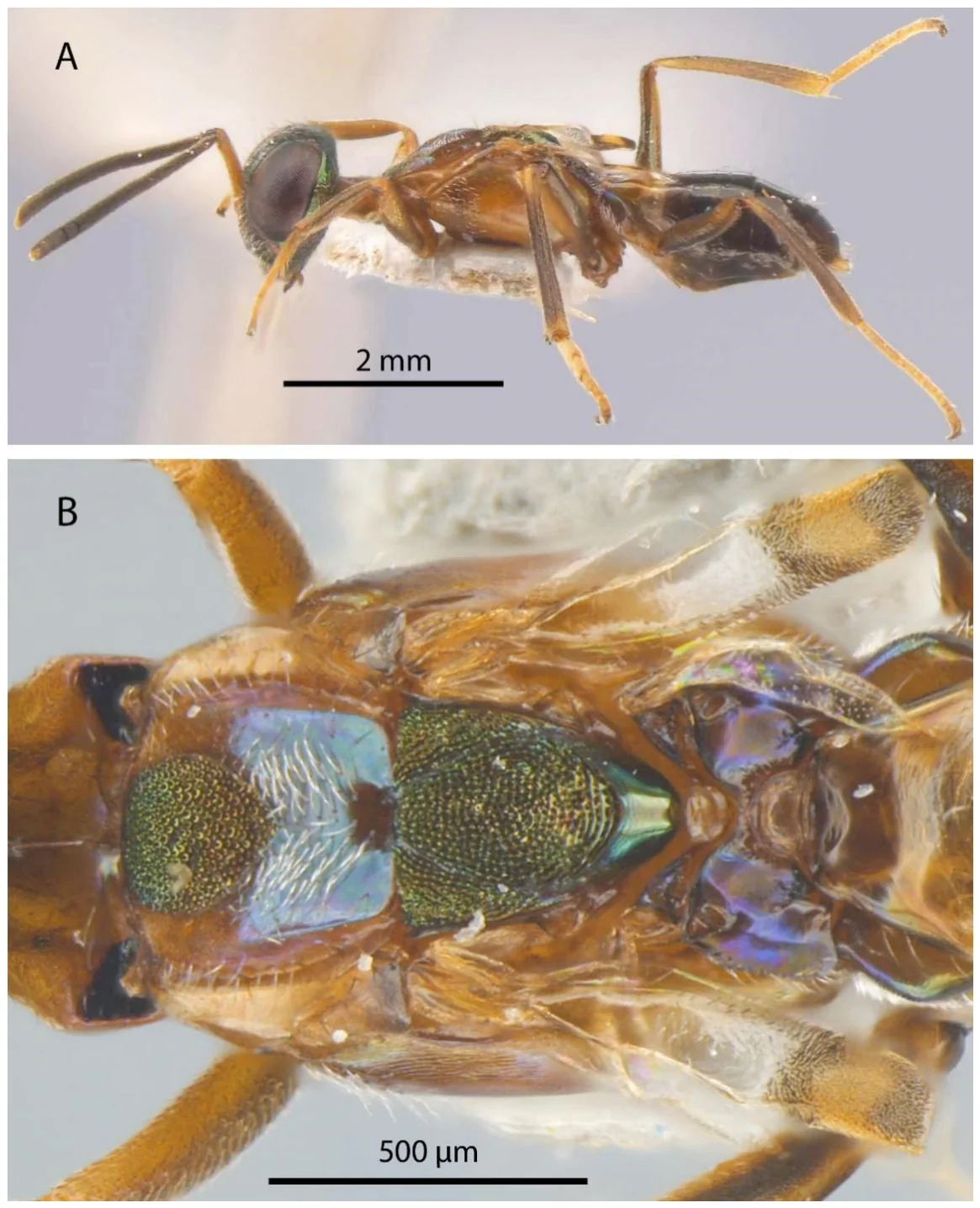
*Anastatus* (*Anastatus*) *meilingensis* ♀ (FAFU-1147): (**A**) body lateral; (**B**) mesosoma.

**Description.** See Peng et al. [4].

**Distribution.** ORIENTAL: China (*Chongqing, Fujian, *Guangdong, Hainan, Hunan, Jiangxi, *Yunnan). PALAEARCTIC: China (Gansu).

**Hosts.** Lepidoptera: Lasiocampidae: *Dendrolimus kikuchii* and *D. punctatus*, Notodontidae: *Stauropus alternus*, Saturniidae: *Antheraea pernyi* and *Caligula japonica* [4]; New host record from this study: ootheca of mantis (Mantodea).

**Remarks.** Molecular analysis revealed that specimens of *A. meilingensis* were mixed with those of *A. echidna* in the ML tree. However, these two species are morphologically distinct: *A. meilingensis* is brachypterous (Figure 32A,B) and lacks a hyaline crossband or spots, whereas *A. echidna* is macropterous and has two hyaline spots (Figure 20A,C,E). See the Discussion section for further explanation.

#### 3.4.15. *Anastatus* (*Anastatus*) *orientalis* Yang & Choi, 2015 (Figure 33)

*Anastatus orientalis*; Choi et al., 2014: 135. Nomen nudum.

*Anastatus orientalis* Yang and Choi, in Yang et al. 2015a: 292–300, figs 1–24. Described: both sexes.

*Anastatus* (*Anastatus*) *orientalis*, Peng et al., 2020: 390–392, figs 20, 21.

**Figure 33:**
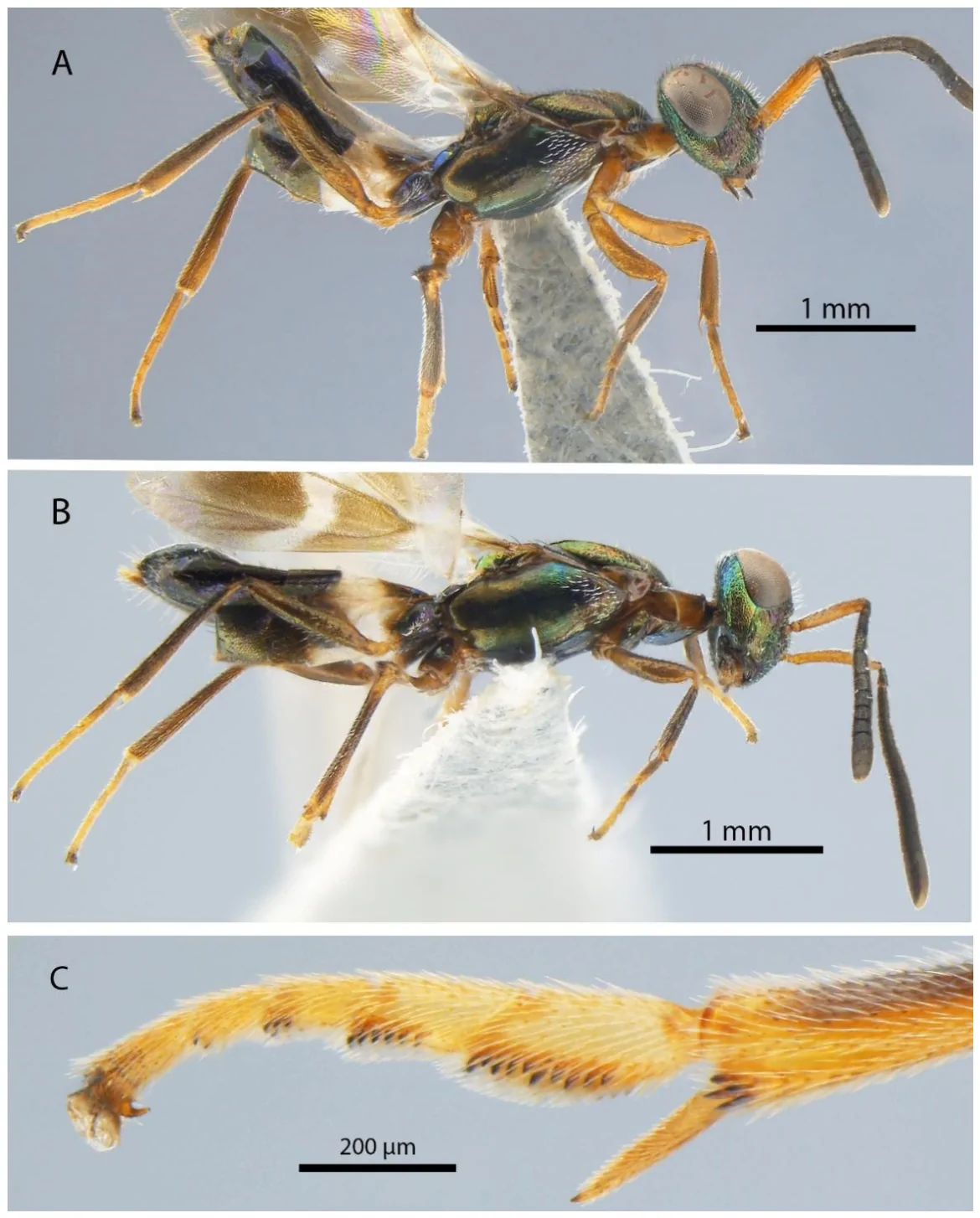
*Anastatus* (*Anastatus*) *orientalis* ♀: (**A**) (FAFU-1376), body lateral; (**B**) (FAFU-1499), body lateral; (**C**) (FAFU-1571), mesotarsus.

**Material examined.** 1♀(FAFU), Wuyishan, Nanping, Fujian Province, China, FAFU-591; 1♀(FAFU), Wuyishan, Nanping, Fujian Province, China, 15.VI–12.VII.2021, FAFU-895; 1♀(FAFU), Wangpingdong, Minjiangyuan Nature Reserve, Sanming, Fujian Province, China, 18.V.2024, FAFU-1306; 1♀(FAFU), Shankou, Minjiangyuan Nature Reserve, Jianning, Fujian Province, China, 27.VIII.2025, FAFU-1499; 1♀(FAFU), Pinggangshang, Minjiangyuan Nature Reserve, Sanming, Fujian Province, China, 30.VI–30.VII.2024, FAFU-1591; 2♀(FAFU), Fuzhou, Fujian Province, China, 19.IX.2023, ex. eggs of Pentatomidae, FAFU-1593, 1594; 1♀(FAFU), Bamianyao, Ziwuling, Fuxian, Yan’an, Shaanxi Province, China, 3.IX.2018, FAFU-663; 1♀(FAFU), Xingangshan, Shangrao, Jiangxi Province, China, 16.V.2022, FAFU-1276; 4♀(FAFU), Red date Orchard, Tianjin Agricultural University, Tianjin, China, 21.IV.2024, Li Weiqiong leg., FAFU-1373, 1374, 1375, 1376; 1♀(FAFU), Wanshougong, Shandong Province, China, 10.VI.2018, Li Tao leg., FAFU-1571.

**Description.** See Peng et al. [4].

**Distribution.** PALAEARCTIC: China (Beijing, Hebei, Shaanxi, Shandong, Tianjin); ORIENTAL: China (*Fujian, *Jiangxi, *Zhejiang). South Korea [4].

**Hosts.** Hemiptera: Fulgoridae: *Lycorma delicatula*, Pentatomidae [4].

**Remarks.** *A. orientalis* is close to *A. fulloi*, but differs as follows: the mesotarsus of *A. orientalis* is uniformly yellowish to white except for dark pegs (Figure 33C), and the procoxae are sometimes dark though usually paler than the lateral panel of the pronotum ventrally (Figure 33A,B). In *A. fulloi*, the mesotarsus is almost completely infuscate, similar to the dark pegs, but at least two basal tarsomeres are conspicuously infuscate over at least the dorsal or posterior ends, and the procoxae on the ventral side are always entirely much darker than the lateral panel of the pronotum. The spur is pale yellow in *A. orientalis* (Figure 33C), whereas in *A. fulloi* it is usually dark.

#### 3.4.16. *Anastatus* (*Anastatus*) *pariliquadrus* Peng & Tang, 2020 (Figure 34)

*Anastatus* (*Anastatus*) *pariliquadrus*, Peng et al., 2020: 392–394, fig. 22.

**Material examined.** 1♀(FAFU), Chebaling Nature Reserve, Shixing, Shaoguan, Guangdong Province, China, 11–27.VIII.2021, FAFU-961; 1♀(FAFU), Jiulianshan Nature Reserve, Longnan, Ganzhou, Jiangxi Province, China, 14.IX.2020, FAFU-990.

**Description.** See Peng et al. [4].

**Distribution.** ORIENTAL: China (Guangdong, *Jiangxi).

**Hosts.** Unknown.

**Figure 34:**
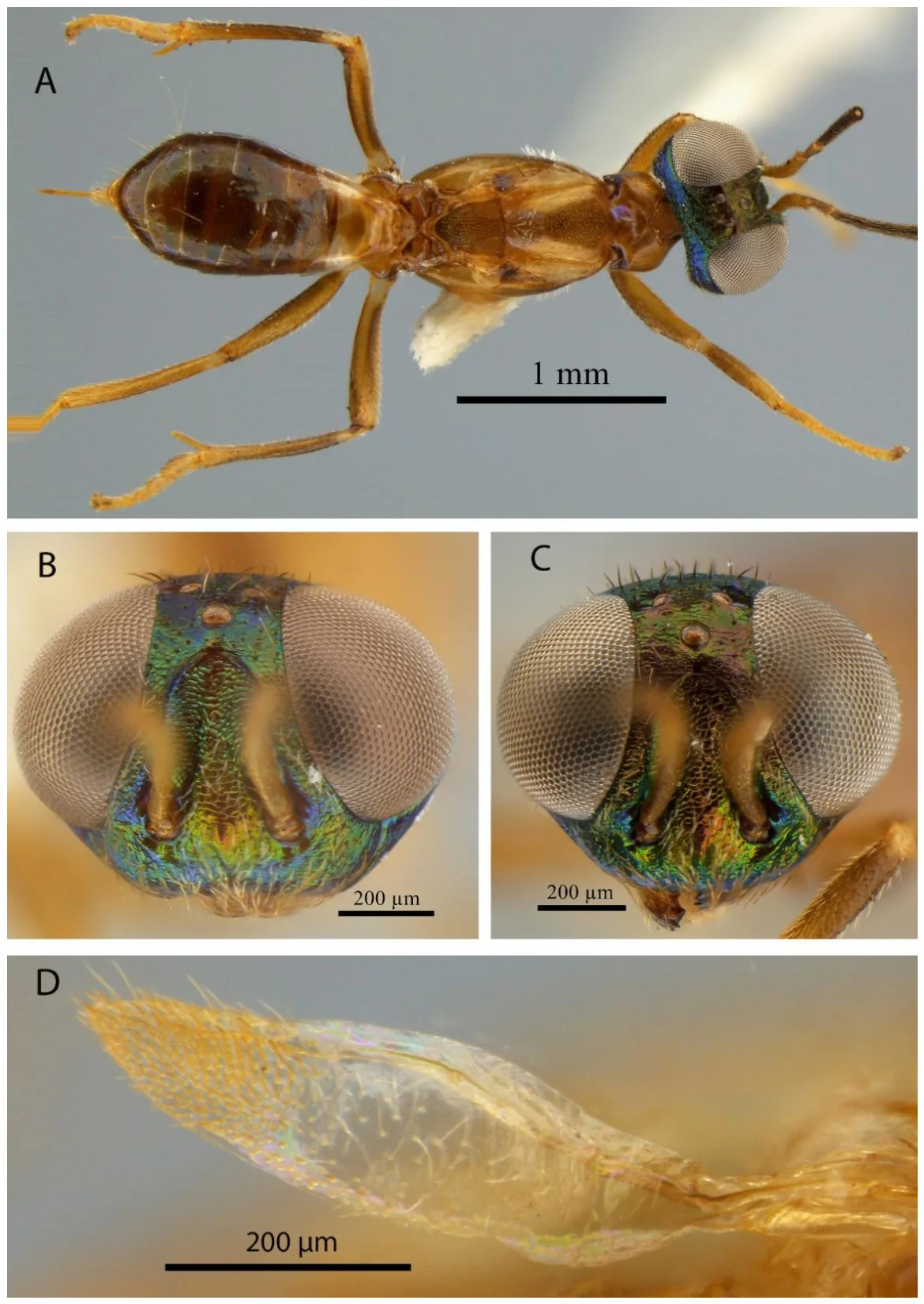
*Anastatus* (*Anastatus*) *pariliquadrus* ♀: (**A**) (FAFU-961), body lateral; (**B**) (FAFU-990), head front; (**C**) (FAFU-961), head front; (**D**) (FAFU-990), fore wing.

**Remarks.** *A. pariliquadrus* is morphologically close to *A. daiyunensis*, but the upper portion of the parascrobal region and the region around the anterior ocellus are rugose or reticulate in *A. pariliquadrus* (Figure 34B,C), whereas they are weakly coriaceous to smooth in *A. daiyunensis*.

#### 3.4.17. *Anastatus* (*Anastatus*) *polikiarkoudus* Wang & Peng, 2024

*Anastatus polikiarkoudus*, Wang et al., 2024:15–16, fig. 8.

**Material examined.** None.

**Description.** See Wang et al. [1].

**Distribution.** ORIENTAL: China (Hainan).

**Hosts.** Unknown.

**Remarks.** This species is special in that its body is covered with dense white setae, except for the scrobal depression, two patches behind eyes, the scutellum, and the posterior portion of the acropleuron. The type specimens of this species are deposited in the Natural History Museum, London, UK, so we were unable to examine them.

#### 3.4.18. *Anastatus* (*Anastatus*) *shichengensis* Sheng & Wang, 1997 (Figure 35)

*Anastatus shichengensis* Sheng and Wang, in Sheng et al. 1997: 58–59, figs 1–5. Described: both sexes.

*Anastatus shichengensis*; Peng et al., 2017: 19–21, figs 41–48.

*Anastatus* (*Anastatus*) *shichengensis*, Peng et al., 2020: 394–397, Figs 23, 24. Described: both sexes.

*Anastatus* (*Anastatus*) *shichengensis*, Li et al., 2025: 354, figs. 3C, D.

**Material examined.** 1♀(FAFU), Longmen Road orchard, Danzhou, Hainan Province, China, 140 m, 23.III.2017, FAFU-174; 3♀(FAFU), Longmen Road orchard, Danzhou, Hainan Province, China, FAFU-381, 382, 383; 2♀(FAFU), Hainan University, Danzhou, Hainan Province, China, 28.III.2021, FAFU-827, 836; 1♀(FAFU), Baoting, Hainan Province, China, 60 m, 25.III.2017, on litchi tree, FAFU-237; 1♀(FAFU), Qianjin Station, Wanning, Hainan Province, China, 19.X–XI.2020, FAFU-1019; 1♀(FAFU), Maoming, Xinyi, Guangdong Province, China, 90 m, 13.IV.2017, on litchi tree, FAFU-236; 1♀(FAFU), Shixing, Chebaling National Nature Reserve, Shaoguan, Guangdong Province, China, 10–20.XI.2022, FAFU-1214; 1♀(FAFU), Shixing, Chebaling National Nature Reserve, Shaoguan, Guangdong Province, China, 30.V–10.VII.2021, FAFU-1296; 1♀(FAFU), Shixing, Chebaling National Nature Reserve, Shaoguan, Guangdong Province, China, 30.IX–10.X.2021, FAFU-1413; 1♀(FAFU), Shixing, Chebaling National Nature Reserve, Shaoguan, Guangdong Province, China, 11–20.X.2022, FAFU-1600; 1♀(FAFU), Dinghushan Nature Reserve, Zhaoqing, Guangdong Province, China, 17.I–17.II.2022, FAFU-1422; 1♀(FAFU), Dinghushan Nature Reserve, mixed coniferous-broadleaf forest-1, Zhaoqing, Guangdong Province, China, 31.III–29.IV.2025, Qinggong Mao leg., FAFU-1724; 2♀(FAFU), woody plant area, South China Botanical Garden, Guangzhou, Guangdong Province, China, Malaise trap, 12.XI–9.XII.2025, Ruichong Wang leg., FAFU-1740; 1♀(FAFU), woody plant area, South China Botanical Garden, Guangzhou, Guangdong Province, China, Malaise trap, (site 7, Pugang), 29.VIII–12.XI.2025, FAFU-1741; 1♀(FAFU), Lingshan People’s Square, Qinzhou, Guangxi Province, China, 608 m, 15.IV.2017, FAFU-242; 1♀(FAFU), Fozizhen, Lingshan, Guangxi Province, China, 68 m, 15.IV.2017, FAFU-292; 1♀(FAFU), Yingtaoling station, Minjiangyuan Reserve, Jianning, Sanming, Fujian Province, China, 950 m, 10.VI.2015, FAFU-274; 2♀(FAFU), Houzhai, Daiyunshan, Dehua, Quanzhou, Fujian Province, China, 15.IX.2015, FAFU-474; same locality, 25.IX.2015, FAFU-487; 1♀(FAFU), Goudunping, Tianbaoyan Reserve, Yong’an, Sanming, Fujian Province, China, 1000 m, 13.VIII.2020, FAFU-819; 3♀(FAFU), Longxiang Island, Fuzhou, Fujian Province, China, 9.IV.2021, FAFU-841, 842, 1158; 1♀(FAFU), Wuyishan, Nanping, Fujian Province, China, Malaise trap, 2021, FAFU-931; 1♀(FAFU), Wuyishan, Fujian Province, China, Malaise trap, 1120 m, 3.XII.2022, FAFU-1264; 1♀(FAFU), Xianfengling, Wuyishan, Fujian Province, China, 1188.6 m, 19.VIII.2025, Qiyan Wang leg., FAFU-1418; 1♀(FAFU), Wuyishan National Park, Fujian Province, China, 860 m, 28.XI.2022, FAFU-1585; 1♀(FAFU), Guizhou, Guizhou Province, China, 15–27.XI.2020, FAFU-1262; 1♀(FAFU), Guizhou University laboratory culture, Guizhou Province, China, 11.VIII.2025, Haoran Liao leg., FAFU-1308; 1♀(FAFU), National Nature Reserve, Shaoguan, Guangdong Province, China, 9.V.2004, FAFU-345; 1♀(FAFU), Nanling National Forest Park, Shaoguan, Guangdong Province, China, 1545 m, 28.IX–22.X.2022, Wenfeng Li leg., FAFU-1273.

**Description.** See Peng et al. [4].

**Distribution.** ORIENTAL: China (Fujian, Guangdong, Guangxi, *Guizhou, Hainan, Jiangxi, Taiwan).

**Host.** Hemiptera: Tessaratomidae: *Tessaratoma papillosa*.

**Figure 35:**
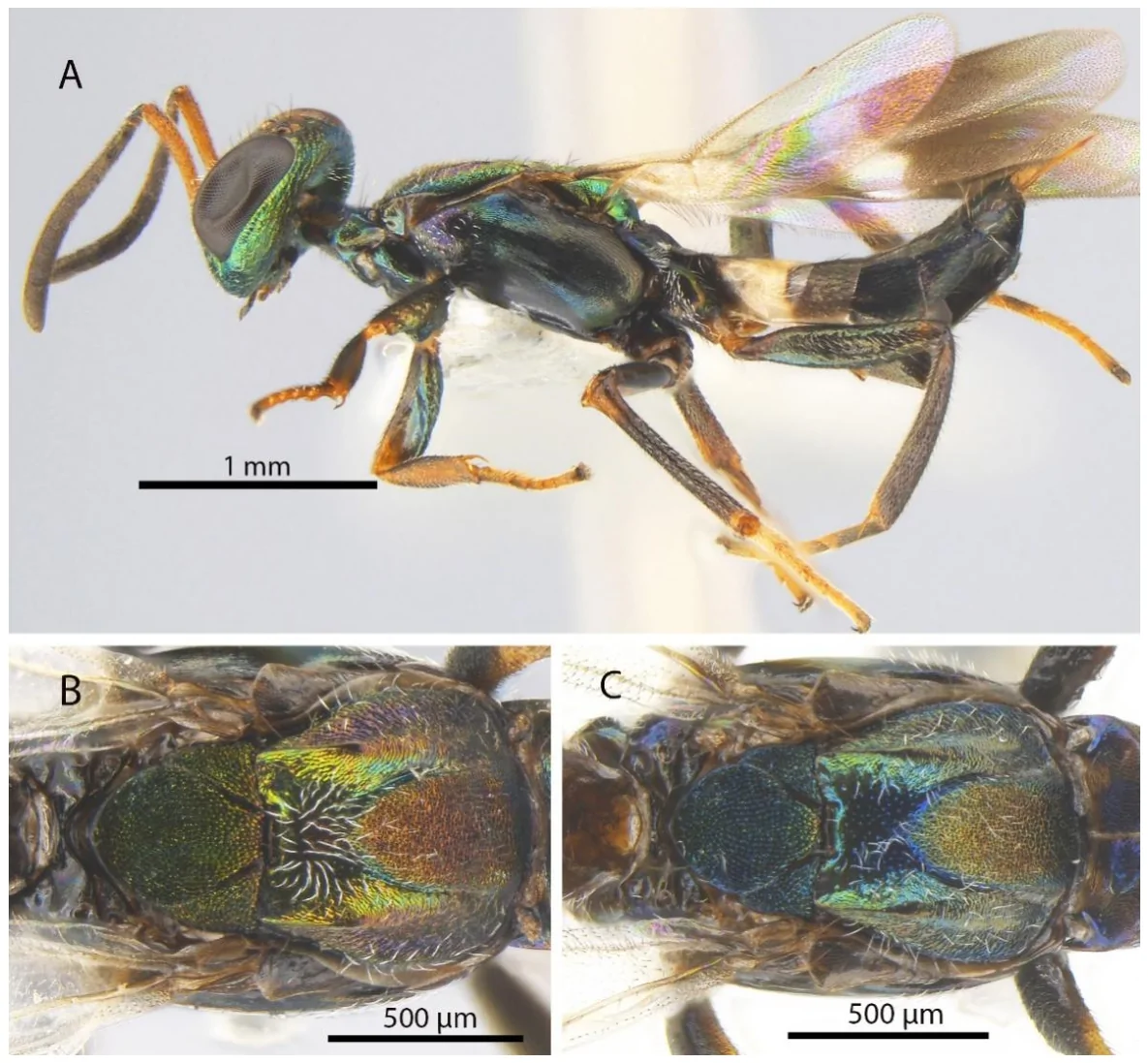
*Anastatus* (*Anastatus*) *shichengensis* ♀: (**A**) (FAFU-1308), body lateral; (**B**) (FAFU-1585), mesosoma; (**C**) (FAFU-1264), mesosoma.

**Remarks.** *A. shichengensis* is characterized by a profemur with the ventral margin obtusely angulate within about the apical third (Figure 35A). *COI* analysis revealed that *A. shichengensis* has three different clades, which together form a monophyletic group. ASAP analysis recovered three putative MOTUs, but specimens from all three clades exhibit the same morphology except for mesoscutum colouration (Figure 35B,C). We only observed different mesoscutal colour variations, but these were not uniquely associated with particular clades. Therefore, based on similar morphology and monophyletic evidence, we regard them as a single species, although the intraspecific genetic divergence was 7.8% (including two specimens from Thailand, THAMJ1185-23 and THAMH5106-23, with the remaining specimens all from China).

#### 3.4.19. *Anastatus* (*Anastatus*) *taibaiensis* Wang & Peng, 2024

*Anastatus taibaiensis*, Wang et al., 2024: 16–18, fig. 9.

**Type material examined.** 2♀(FAFU), Haopingsi, Taibai Mountain, Baoji City, Shaanxi Province, China, Malaise trap, 22.VIII.2014, Yangzhao Fu leg., FAFU-20, 21.

**Description.** See Wang et al. [1].

**Distribution.** ORIENTAL: China (Shaanxi).

**Hosts.** Unknown.

#### 3.4.20. *Anastatus* (*Anastatus*) *zdenekbouceki* Zhou & Peng, 2024

*Anastatus zdenekbouceki*, Wang et al., 2024: 18–20, fig. 10.

**Material examined.** None.

**Description.** See Wang et al. [1].

**Distribution.** ORIENTAL: China (Hainan).

**Hosts.** Unknown.

### 3.5. Key to the Species of Chinese Anastatus Motschulsky, 1859

**1.** fl2 distinctly longer than clava; propodeum with comparatively long plical region, the foramen not extended to anterior margin of propodeum **·······································································································*A*. (*Cladanastatus*) Bouček···2**

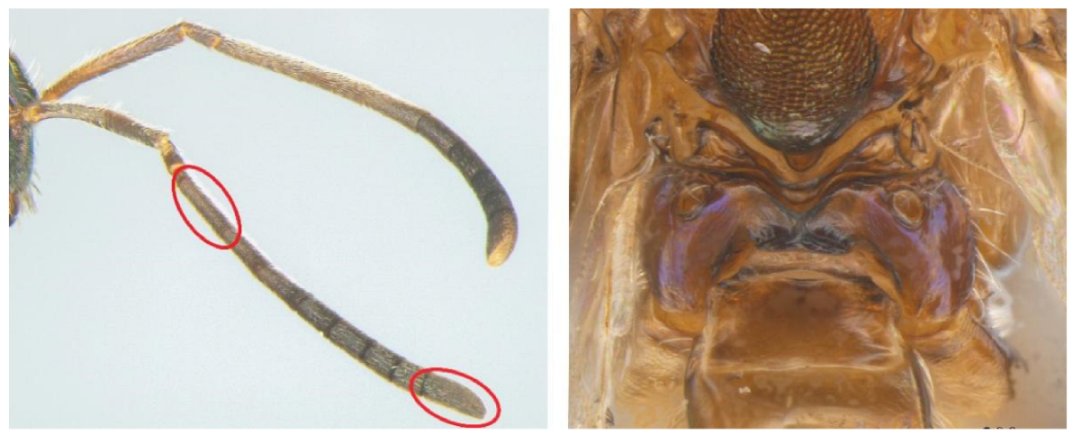

- fl2 shorter or rarely about as long as clava; propodeum with foramen incurved to V-shaped anteromedial emargination of anterior margin **·····································································································*A.* (*Anastatus*) Motschulsky···3**

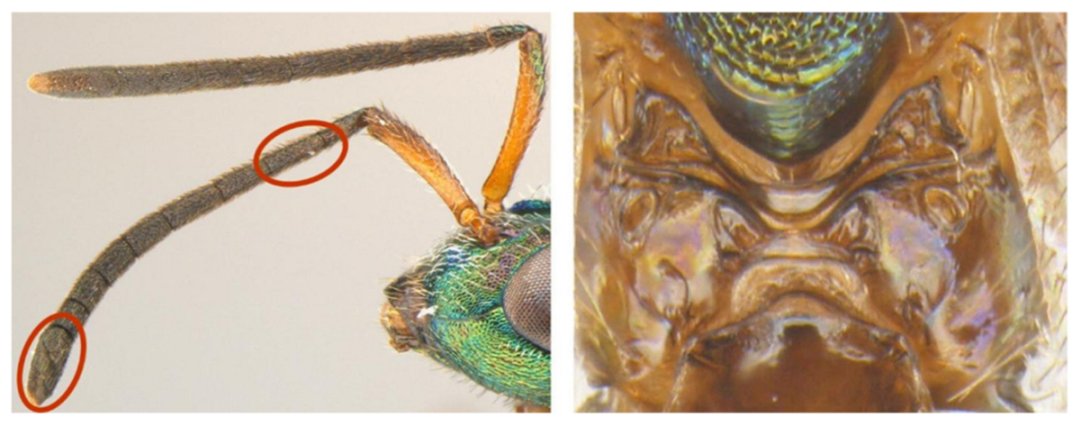

**2(1).** Ovipositor sheaths about 0.38× the length of gaster while about 0.42× the length of the hind tibiae in dorsal view **························*A.* (*Cladanastatus*) *madagascariensis* Bouček**

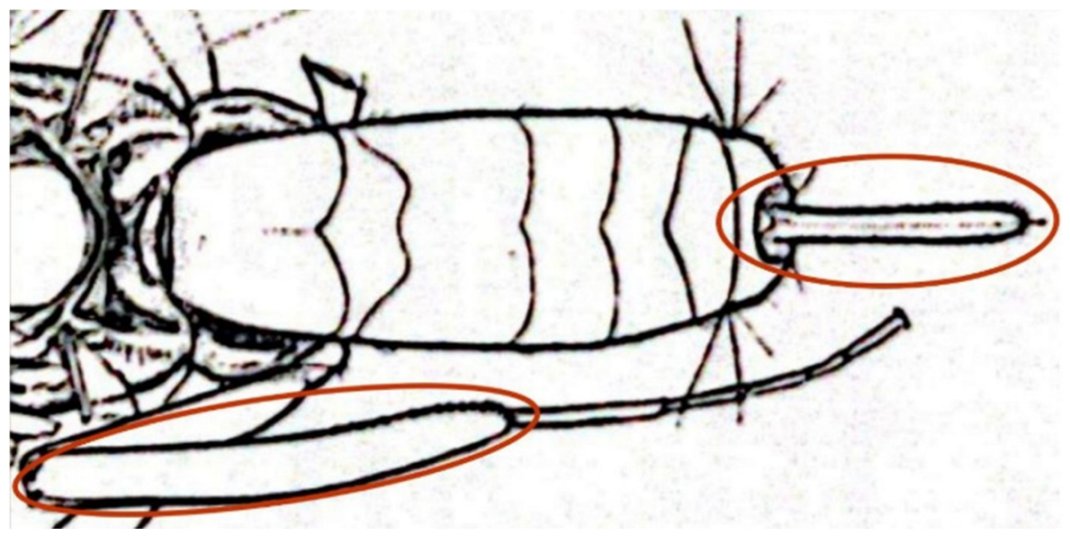

- Ovipositor sheaths on an average about 0.13× the length of gaster and 0.29× the length of hindtibiae in dorsal view **·············································································································*A.* (*Cladanastatus*) *alexstrasza* Khan, Li & Peng, sp. nov.**

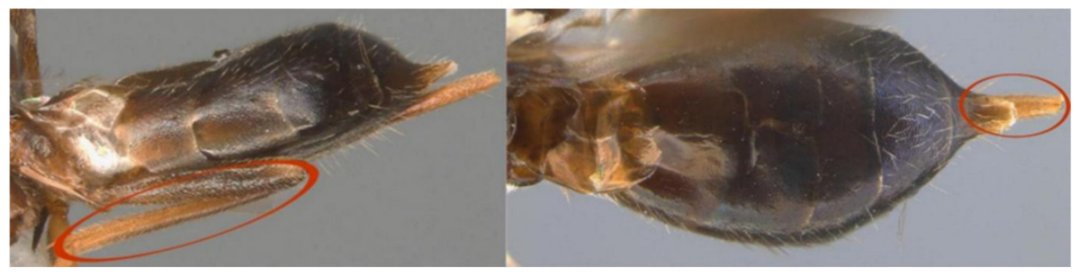

**3(1).** Fore wing brachypterous to medium sized, not extending to the apex of gaster **··························································································································································4**

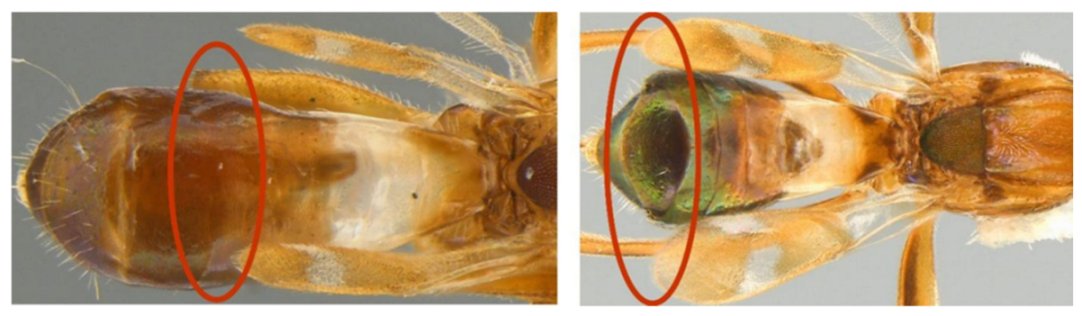

- Fore wing macropterous, extending at least to or exceeding the apex of gaster **··················9**

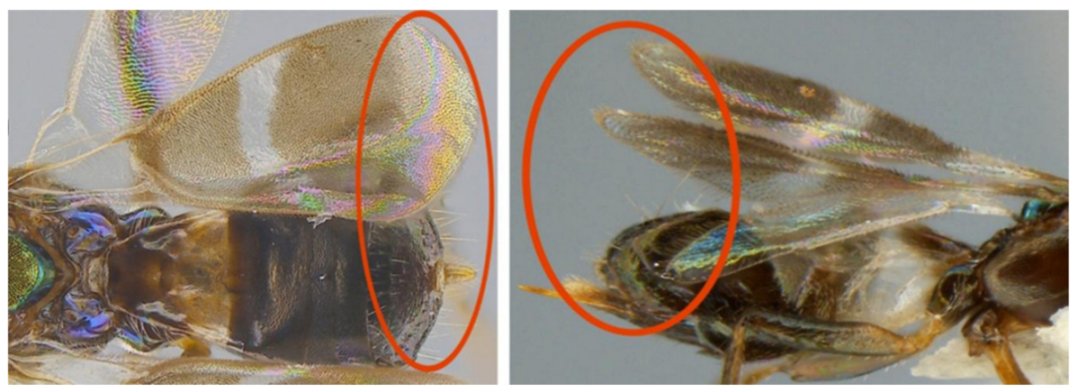

**4(3).** Fore wing with hyaline crossband behind mv and variable in length ranging from brachypterous to medium large; mesoscutum, scutellum, and axilla with strong green to blue metallic lustre to without metallic lustre **························································································*A*. (*Anastatus*) *gastropachae* Ashmead**

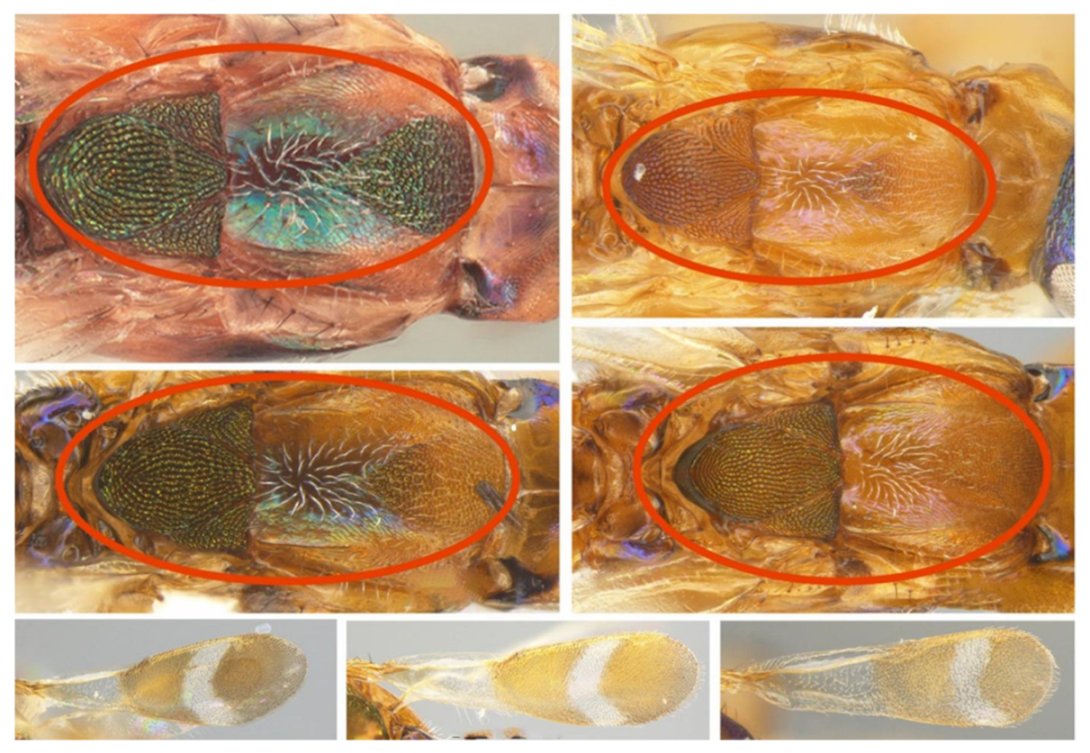

- Fore wing brachypterous, without hyaline crossband and exceeding up to Gt2 **················5**

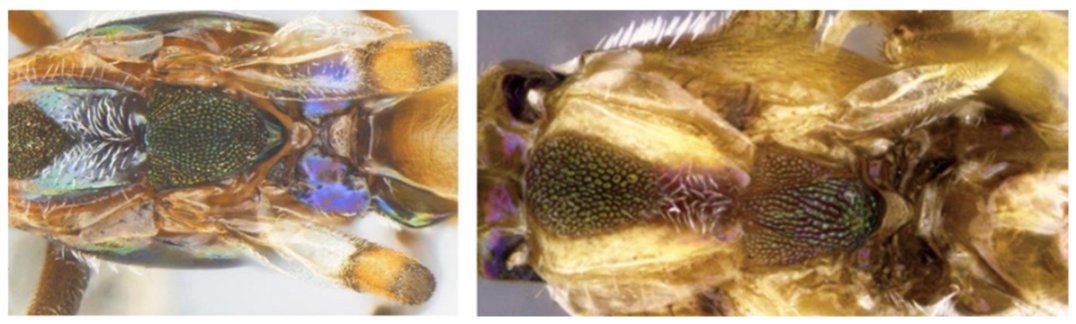

**5(4).** Body covered with dense white setae, except scrobal depression, two patches behind eye, scutellum, and posterior portion of acropleuron **·············································································*A*. (*Anastatus*) *polikiarkoudus* Wang & Peng**

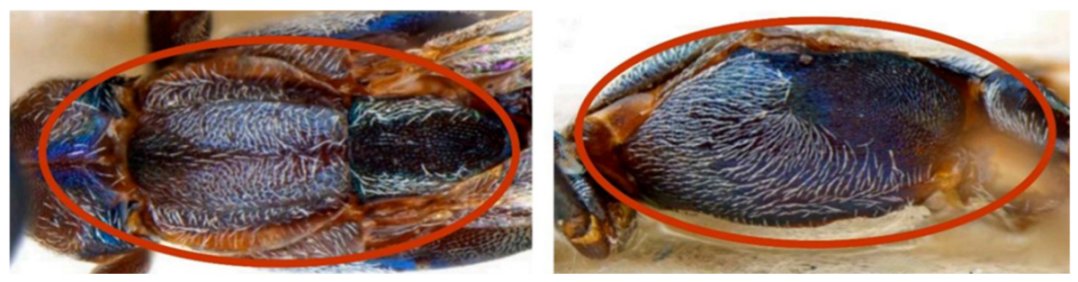

- Body setation different from the species above **········································································6**

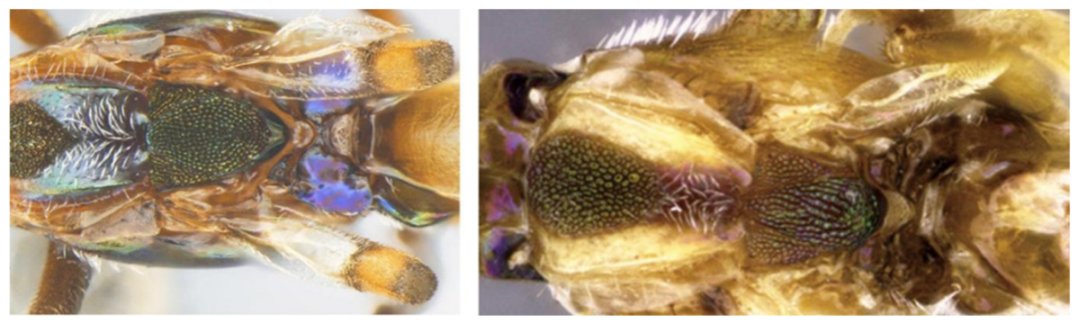

**6(5).** Upper portion of parascrobal region and region around anterior ocellus weakly coriaceous to smooth **··················································*A*. (*Anastatus*) *daiyunensis* Wang & Peng**

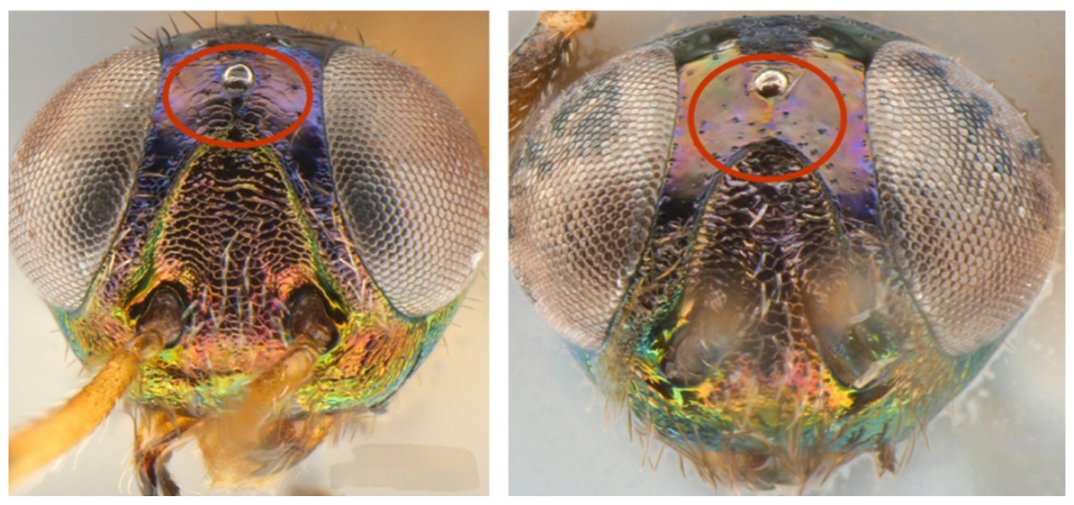

- Upper portion of parascrobal region and region around anterior ocellus not smooth, though rugose or reticulate **···········································································································7**

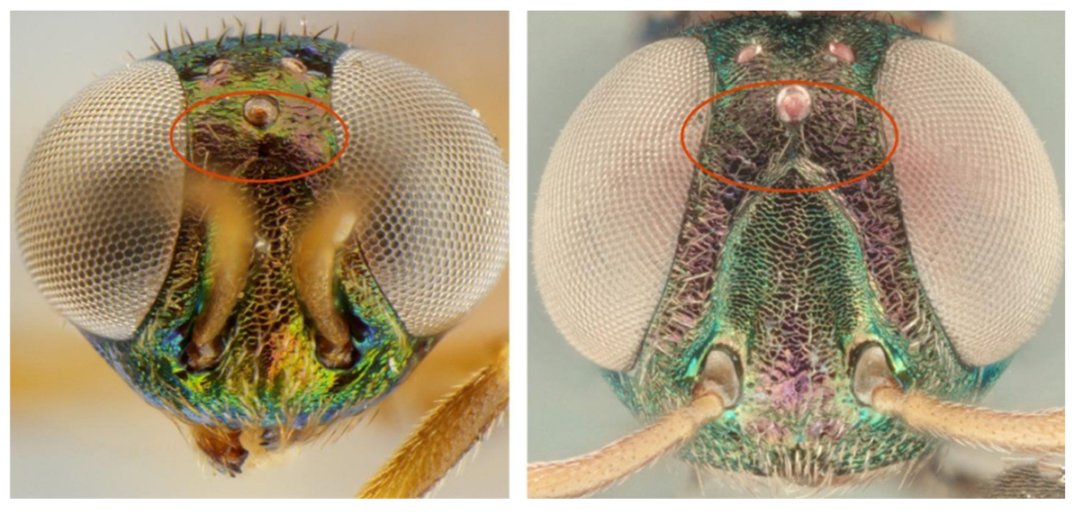

**7(6).** Fore wing discal region covered with dense, light-coloured setae **··················································································*A*. (*Anastatus*) *pariliquadrus* Peng & Tang**

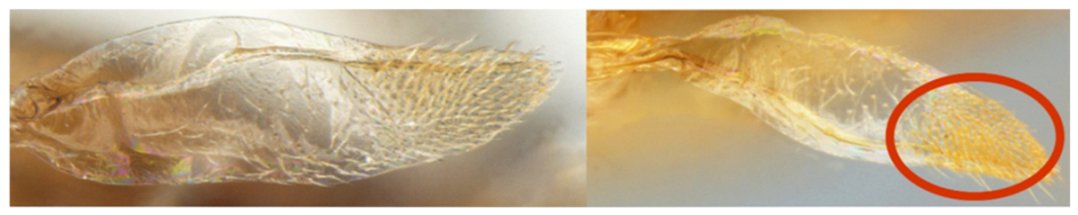

- Fore wing discal region covered with dense, dark brown setae except for region of orangish setae medially **················································································································8**

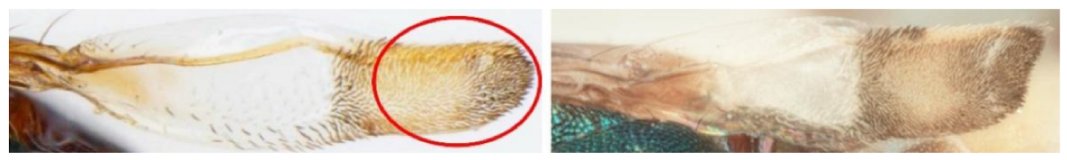

**8(7).** Fore wing with rounded apex **·············································································································*A*. (*Anastatus*) *zdenekbouceki* Zhou & Peng**

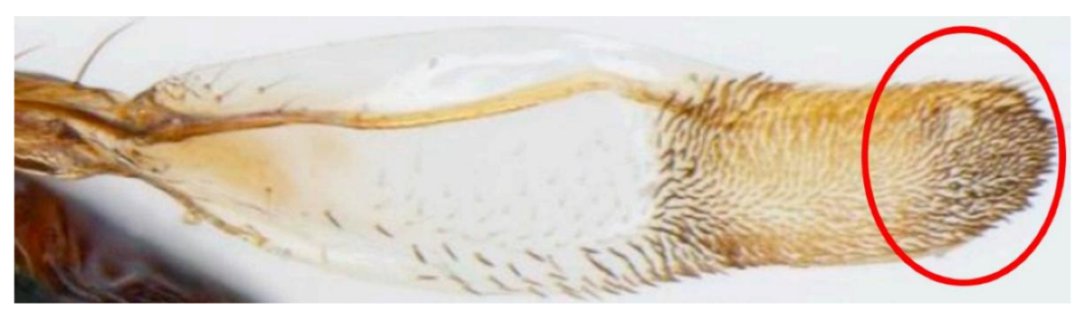

- Fore wing with apex somewhat truncate **··························································································*A*. (*Anastatus*) *meilingensis* Sheng & Yu**

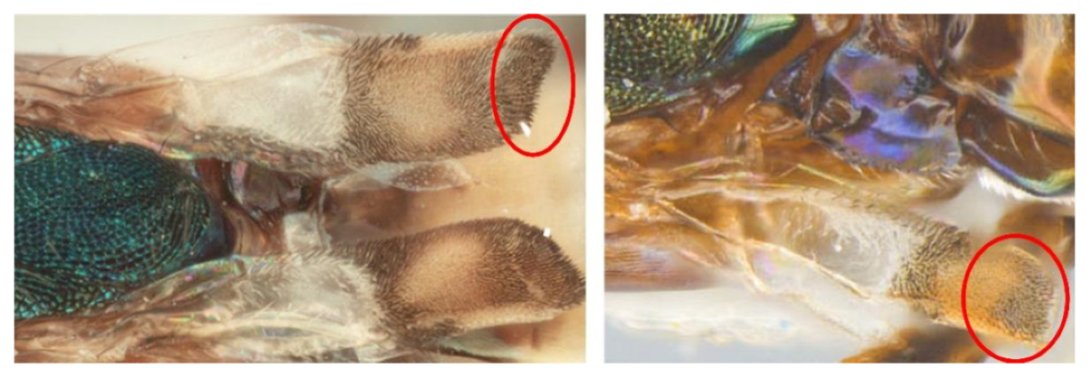

**9(3).** Fore wing without hyaline crossband behind marginal vein **········································································*A*. (*Anastatus*) *ashrafi* Khan, Li & Peng, sp. nov.**

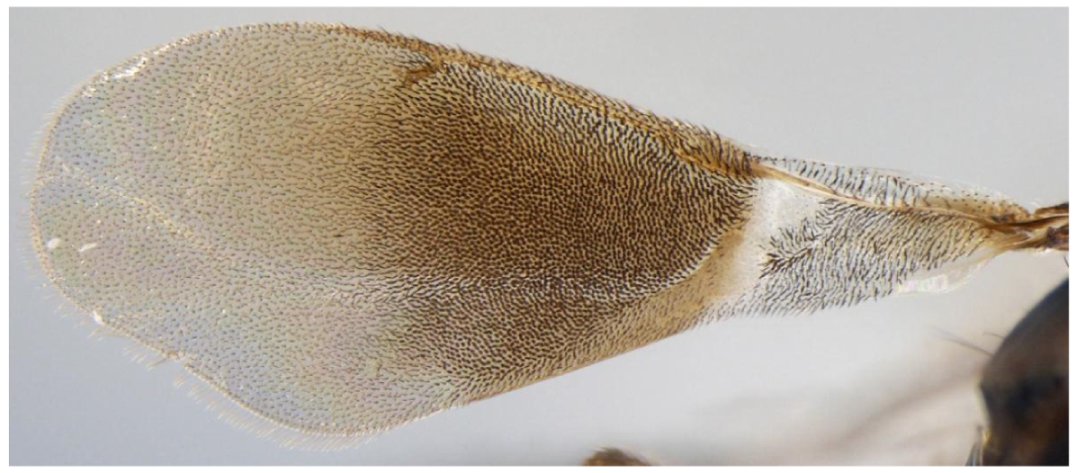

- Fore wing with hyaline crossband, while sometimes interrupted by dark setae medially or two spots behind marginal vein **·····················································································10**

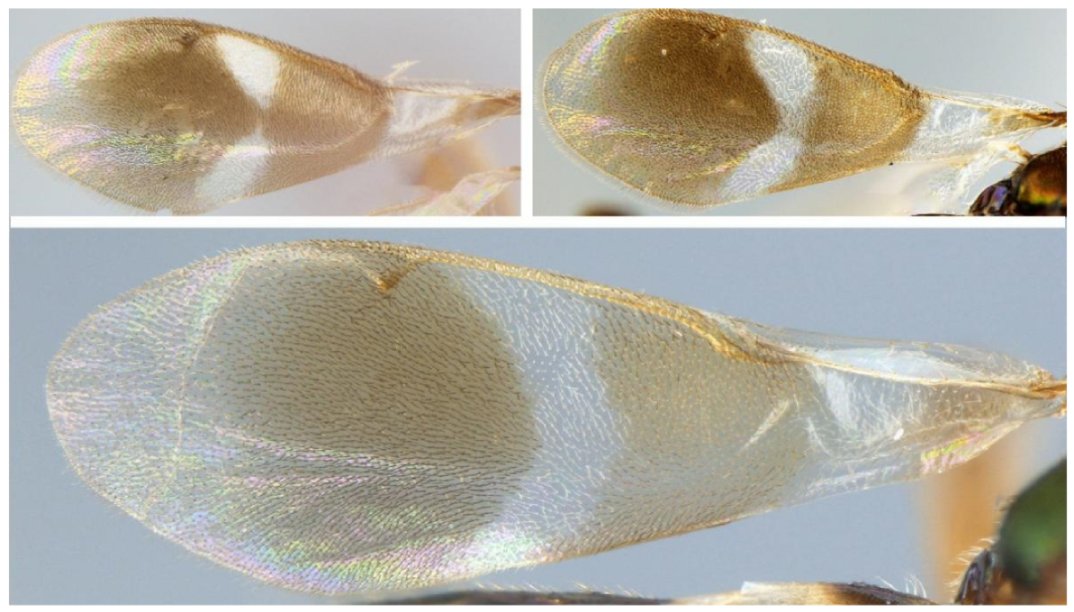

**10(9).** Fore wing with two hyaline spots **················································································11**

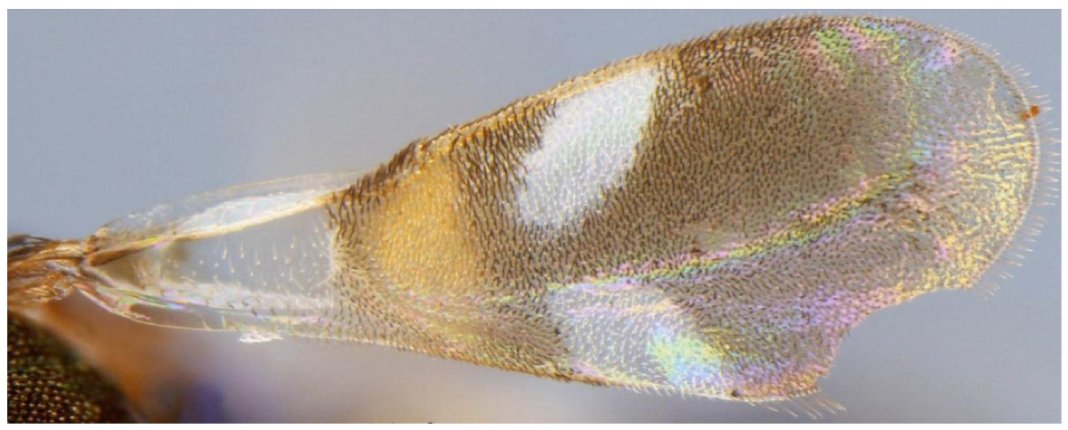

- Fore wing with complete hyaline crossband or sometimes interrupted medially with dark setae (looks constricted) **·····································································································13**

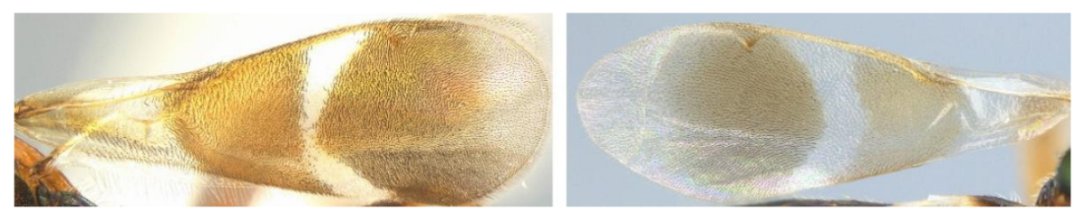

**11(10).** Posterior depressed region of mesoscutal medial lobe with very few setae; dark setae between hyaline spots more dispersed, fore wing basal cell bare; ovipositor sheaths comparatively longer **····································································································*A*. (*Anastatus*) *neltharion* Khan, Li & Peng, sp. nov.**

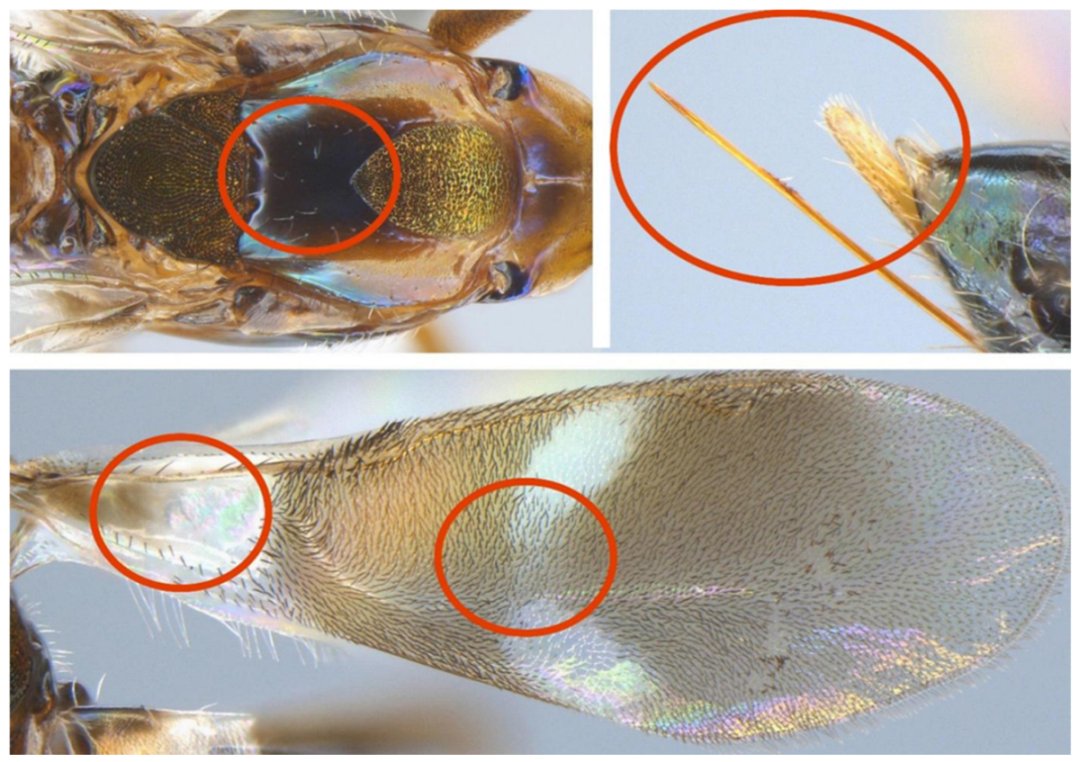

- Posterior depressed region of mesoscutal medial lobe with dense to medially restricted setae; dark setae between hyaline spots not dispersed, fore wing basal cell not bare; ovipositor sheaths comparatively shorter **······················································································12**

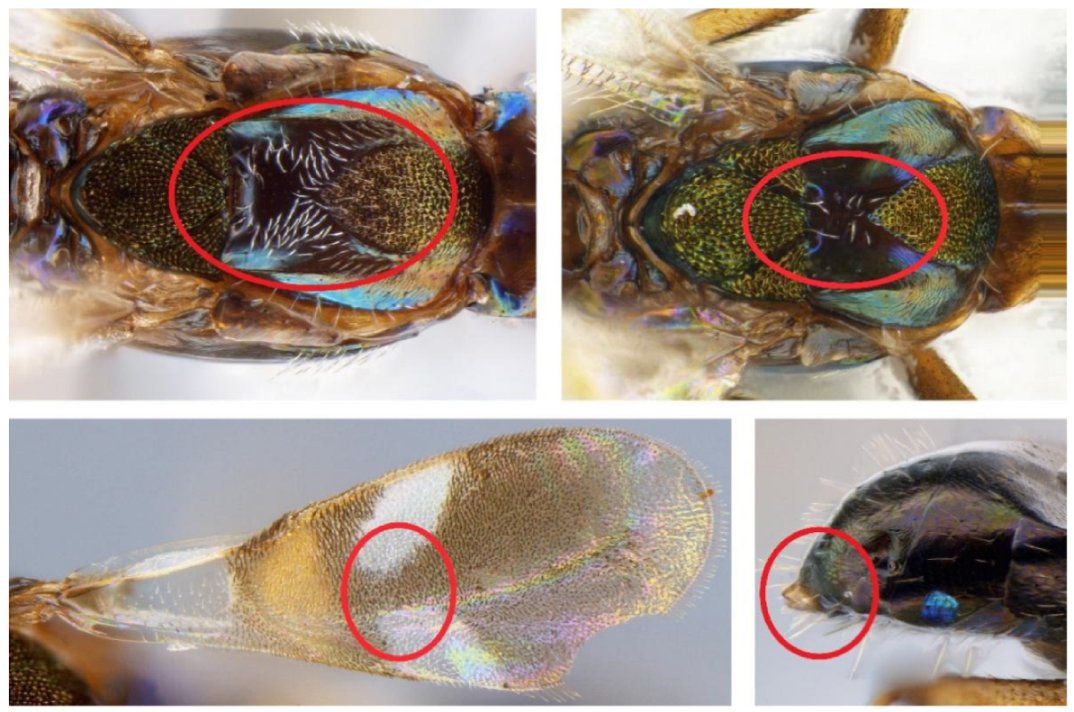

**12(11).** Posterior depressed region of mesoscutal medial lobe with dense white setae; dark setae between hyaline spots not scattered **·····················································································································*A*. (*Anastatus*) *echidna* Motschulsky**

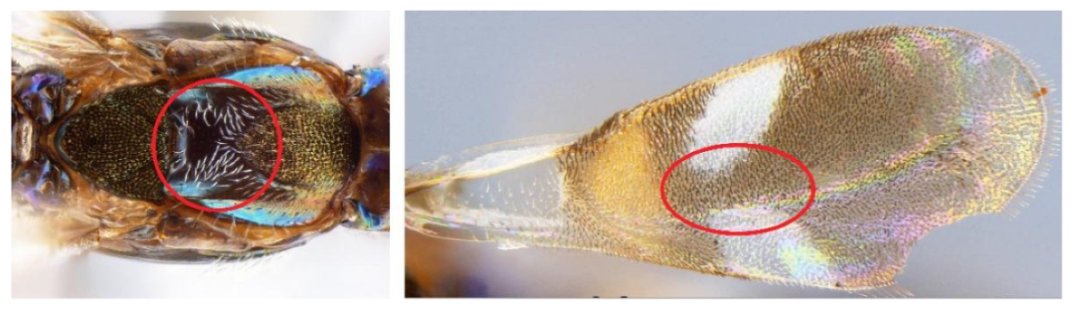

- Posterior depressed region of mesoscutal medial lobe medially setose for a width about equal to width of the bare region on either side; fore wing hyaline crossband constricted and sometime appears as two spots **··············································································································*A*. (*Anastatus*) *gansuensis* Chen & Zang**

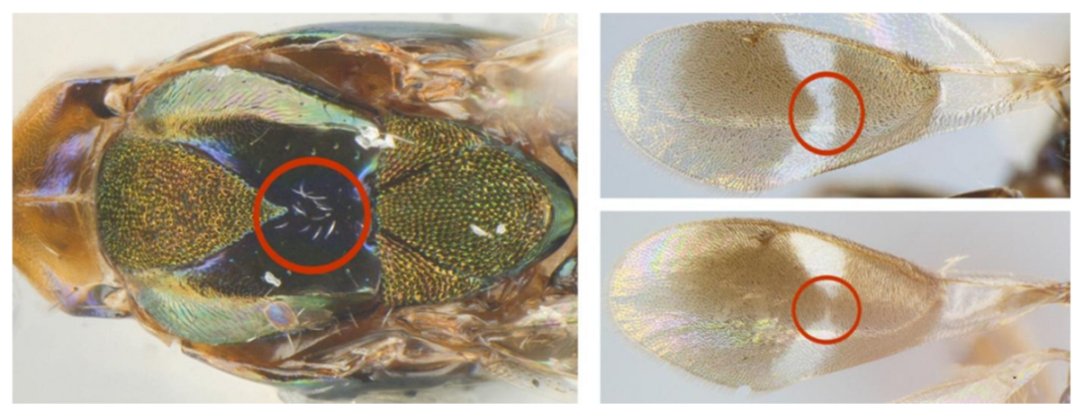

**13(10).** Profemur ventrally with blunt to sharp tooth within about apical third **·························································································································································14**

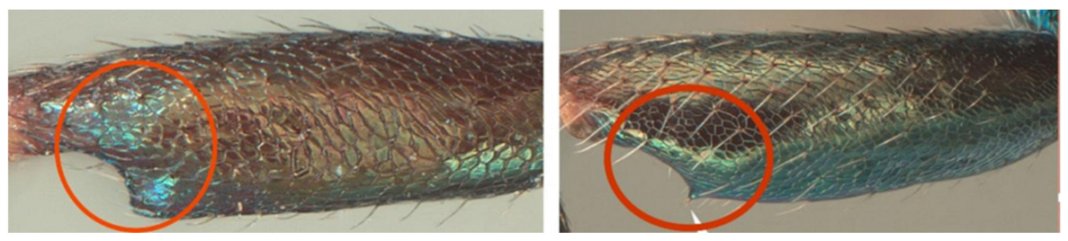

- Profemur ventrally evenly curved, without distinct angulation or tooth apically **·························································································································································18**

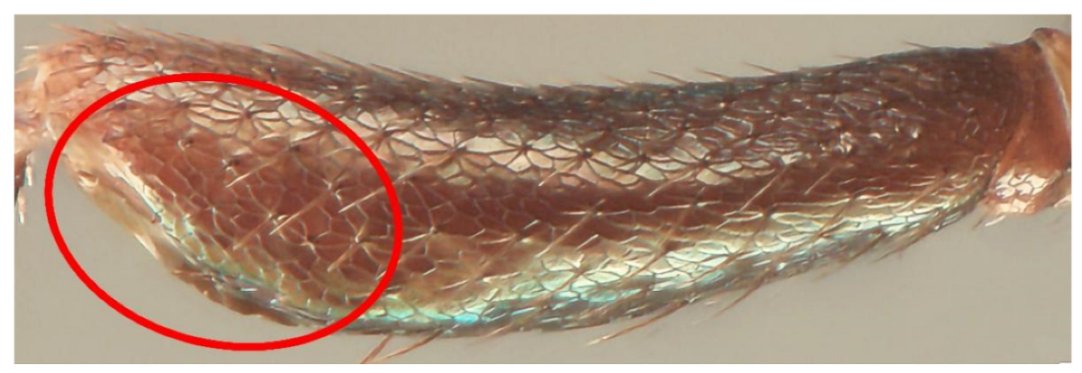

**14(13).** Anterior convex region of mesoscutal medial lobe mesh-like coriaceous to pustulate over anterior half and mesh-like reticulate over posterior half; posterior depressed region of mesoscutal medial lobe only sparsely setose **································································································*A*. (*Anastatus*) *colemani* Crawford**

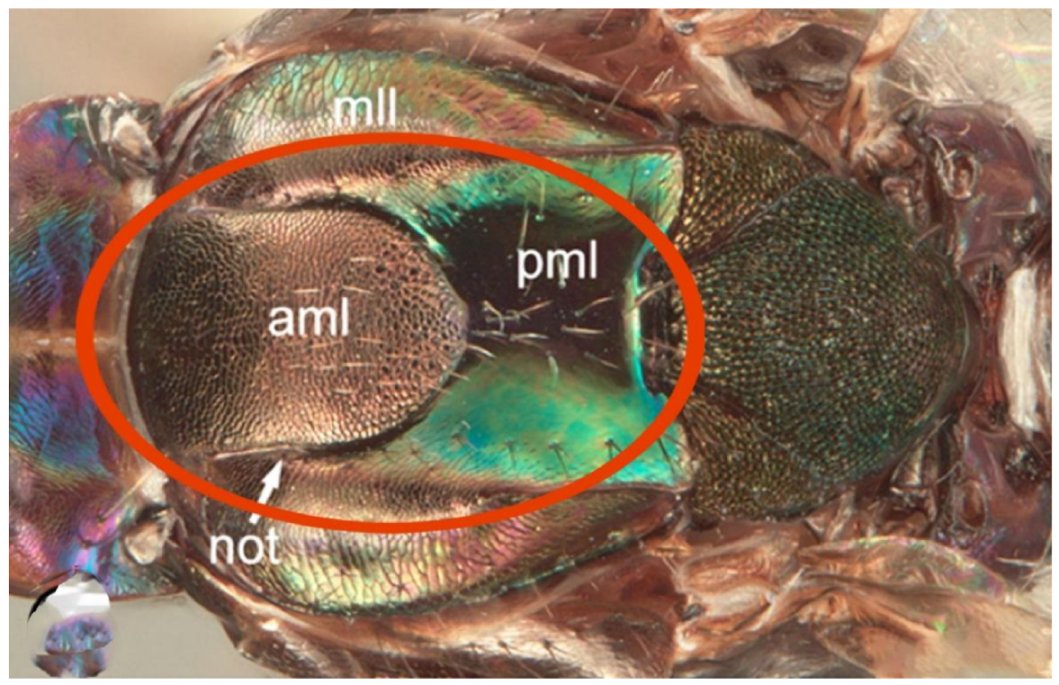

- Anterior convex region of mesoscutal medial lobe entirely reticulate; posterior depressed region of mesoscutal medial lobe densely setose **·····································································15**

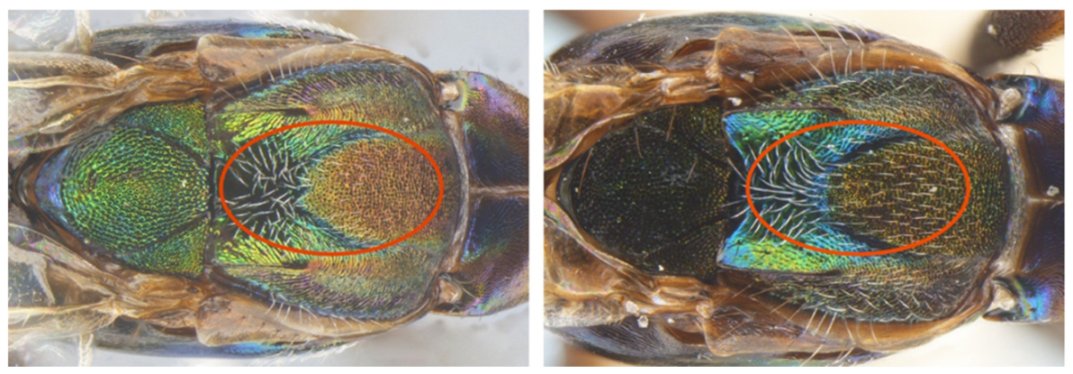

**15(14).** Profemur with ventral margin abruptly angulate, forming a blunt tooth **··············································································*A*. (*Anastatus*) *shichengensis* Sheng & Wang**

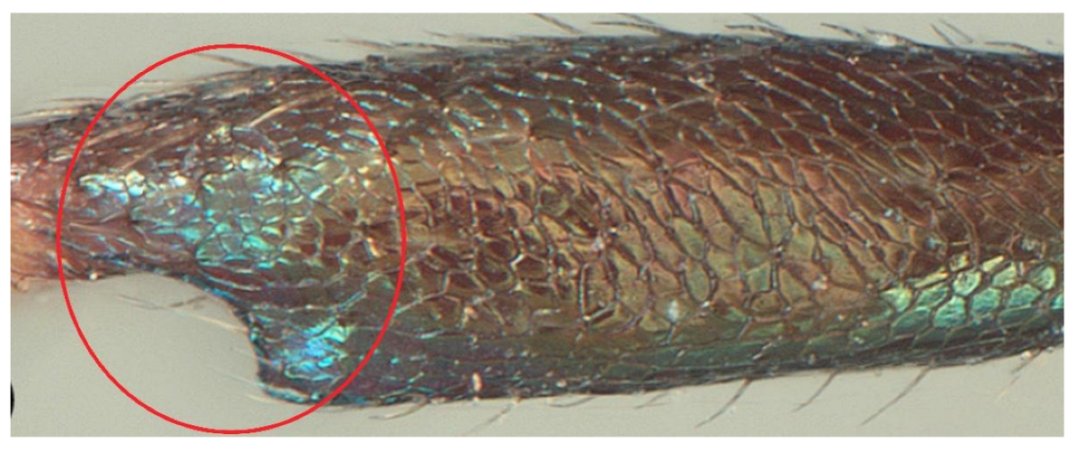

- Profemur with ventral tooth distinct and spine-like [*formosanus* group] **···························16**

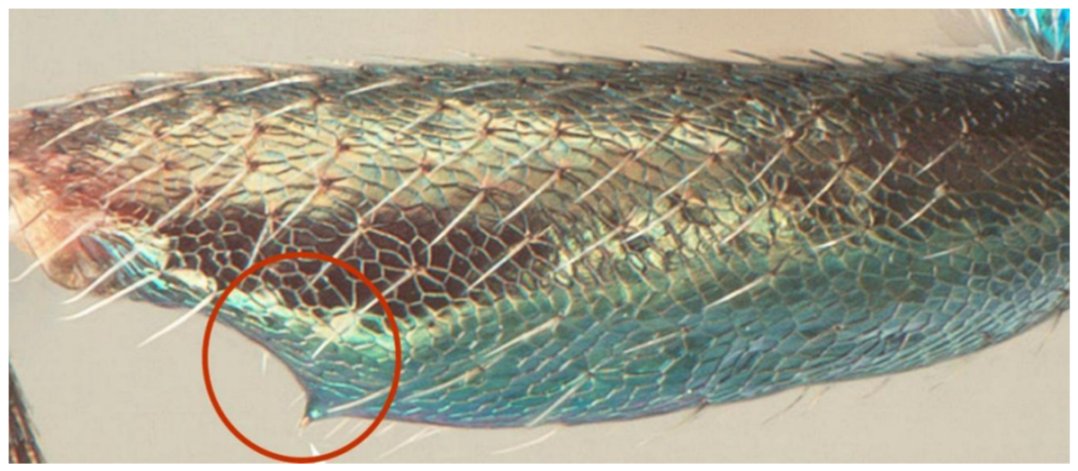

**16(15).** Fore wing with narrow hyaline crossband, infuscate region behind parastigma almost 5.7× to 9.1× wider than crossband, and many dark setae within hyaline region; mesotibial apical spur usually dark brown **··················································································································*A*. (*Anastatus*) *garygibsoni* Zhou & Peng**

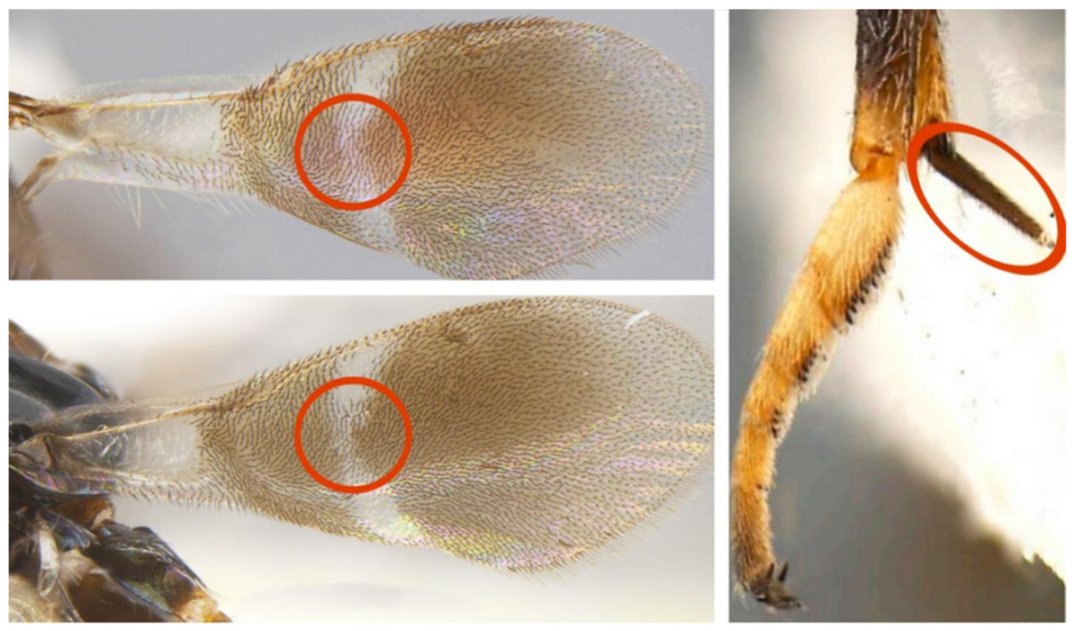

- Fore wing with wide hyaline crossband, infuscate region behind parastigma about 1.67× to 5.3× wider than crossband; mesotibial apical spur sometimes dark brown or as pale as metatarsus **····································································································································17**

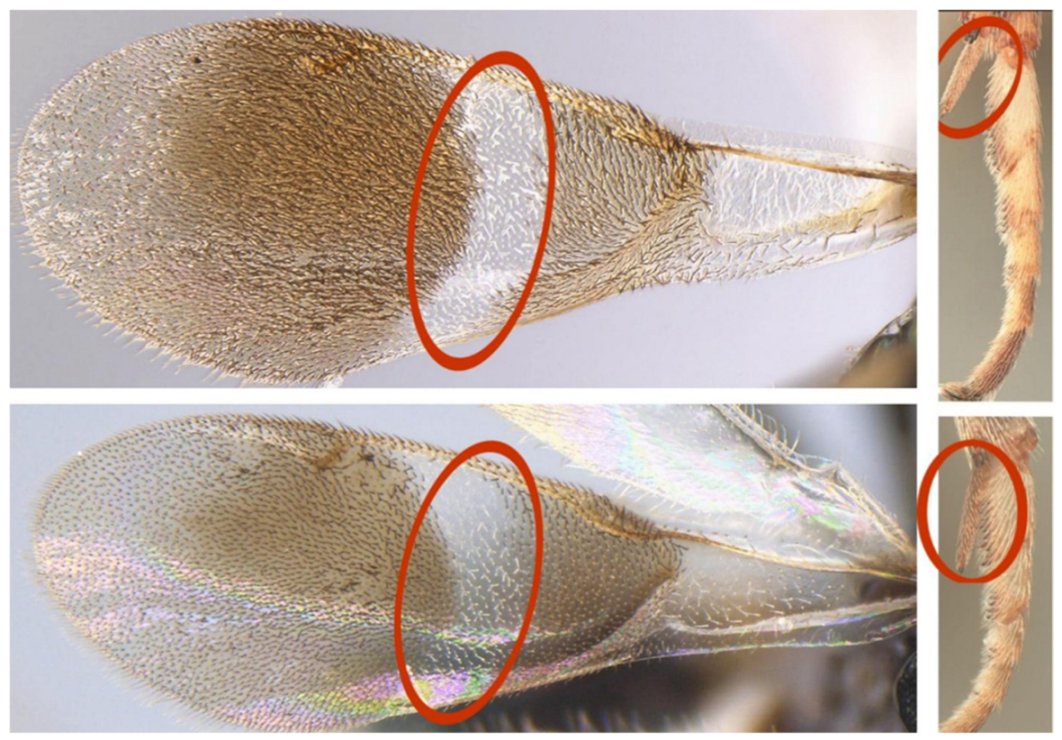

**17(16).** Females exactly similar based on morphology, though separated based on *COI* and male morphology; setal pattern of male’s costal cell not bolder and clearer; setal pattern of speculum posteriorly closed line indistinct **··································································································*Anastatus formosanus* Crawford**

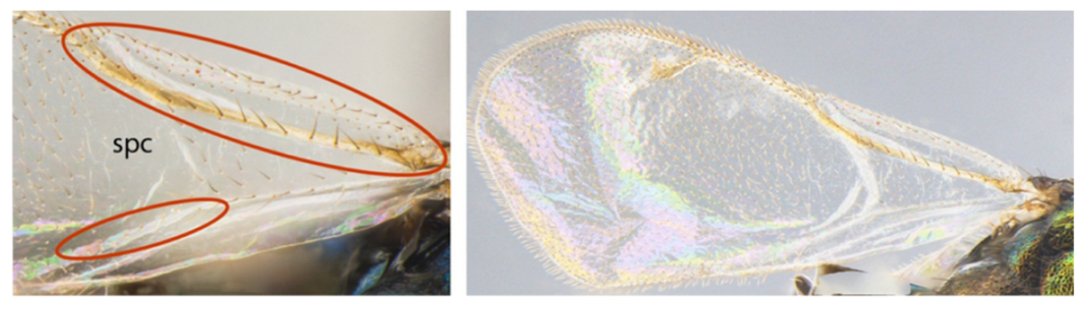

- Females exactly similar based on morphology, though separated based on *COI* and male morphology; setal pattern of male’s costal cell bolder and clearer; setal pattern of speculum posteriorly closed line distinct **························································································································*A*. (*Anastatus*) *dexingensis* Sheng & Wang**

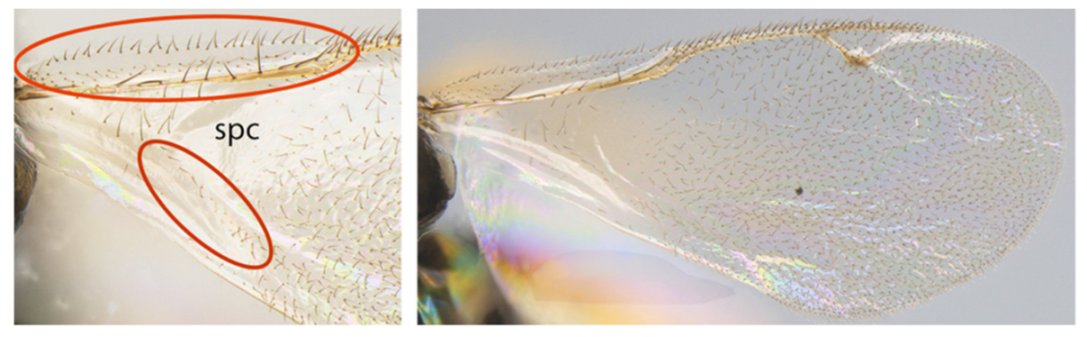

**18(13).** Anterior convex region of mesoscutal medial lobe at least extensively mesh-like coriaceous, at most distinctly reticulate only posteriorly **·······················································19**

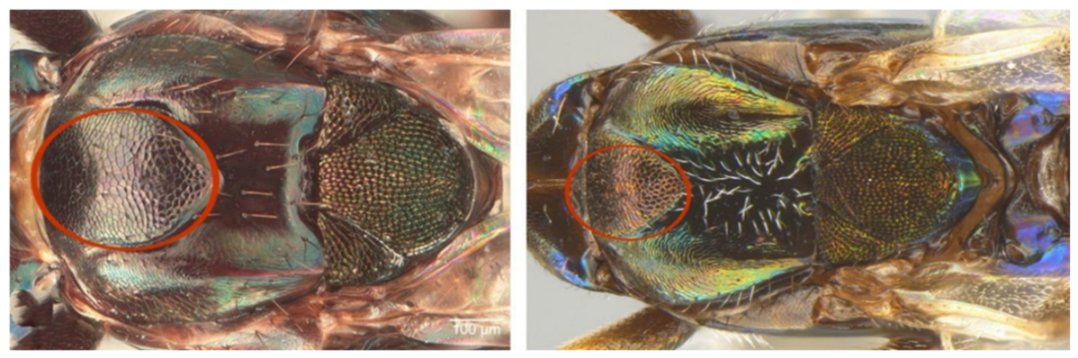

- Anterior convex region of mesoscutal medial lobe mostly to entirely mesh-like reticulate or transversely alutaceous-imbricate to strigose-imbricate **···················································20**

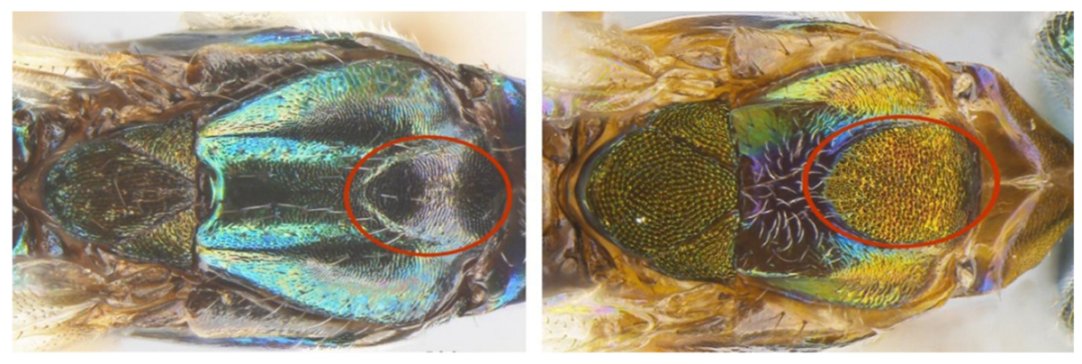

**19(18).** Ovipositor shorter than apical tarsomere **·····································································································*Anastatus bifasciatus* Geoffroy**

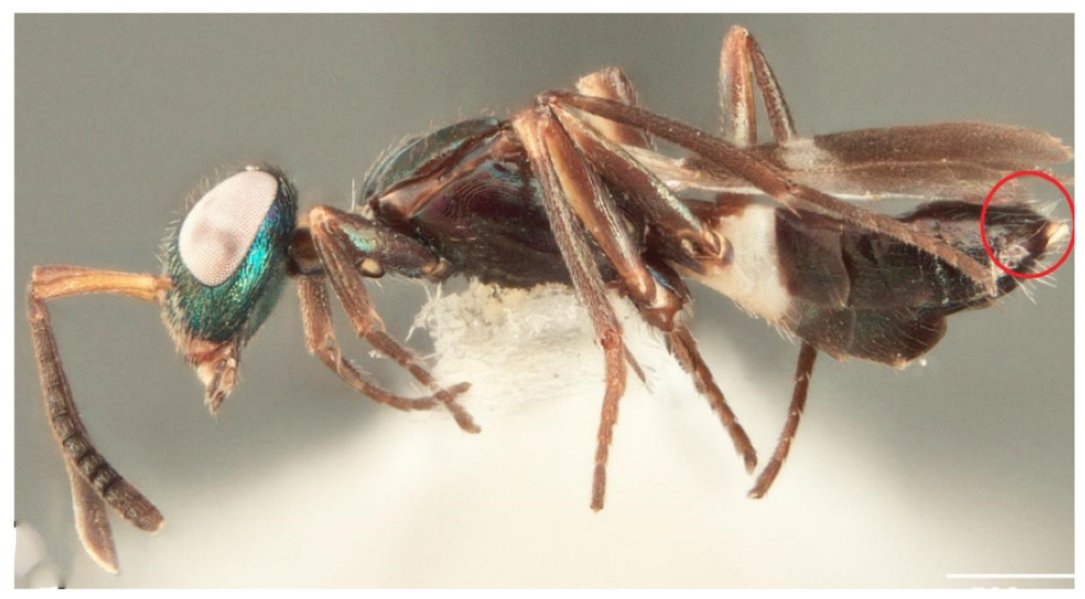

- Ovipositor much longer than apical tarsomere **··················································································*A*. (*Anastatus*) *makrysourus* Zhou & Peng**

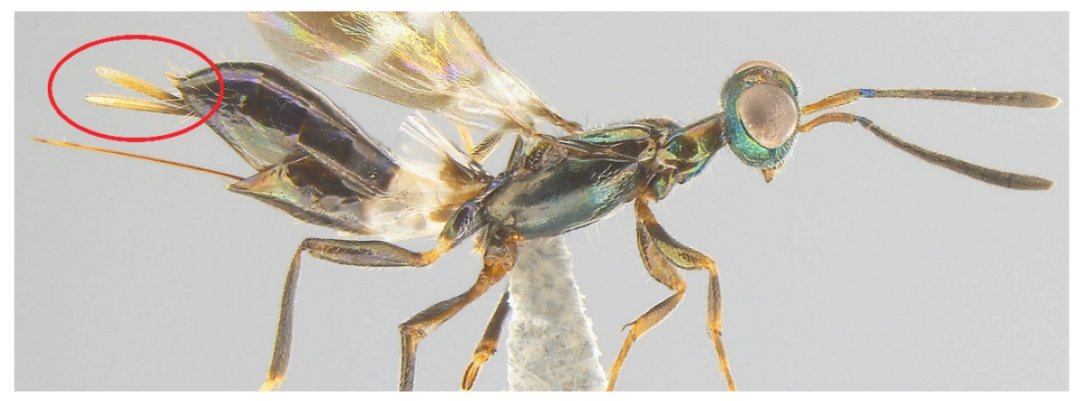

**20(18).** Anterior convex region of mesoscutal medial lobe mostly transversely alutaceous-imbricate to strigose-imbricate, and posterior depressed region entirely mesh-like reticulate; fore wing hyaline crossband obscure and narrowed laterally **·······················································································*A*. (*Anastatus*) *mantoidae* Motschulsky**

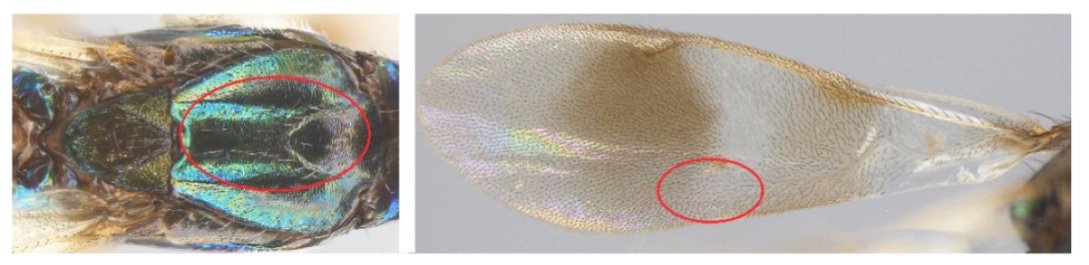

- Anterior convex region of mesoscutal medial lobe mostly to entirely reticulate, posterior depressed region reticulate to smooth; fore wing hyaline crossband clear **·························································································································································21**

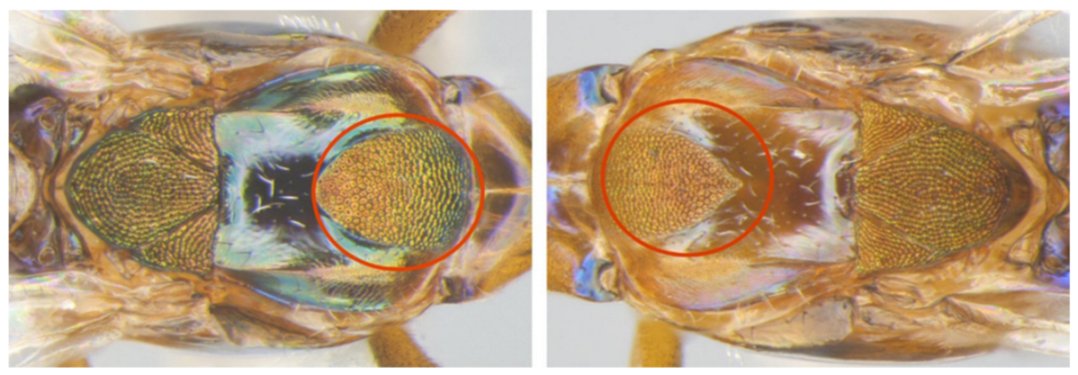

**21(20).** Acropleuron with posterior two-thirds to one-third paler (orange, brown, or yellow) than dark mesoscutum **·······································································································22**

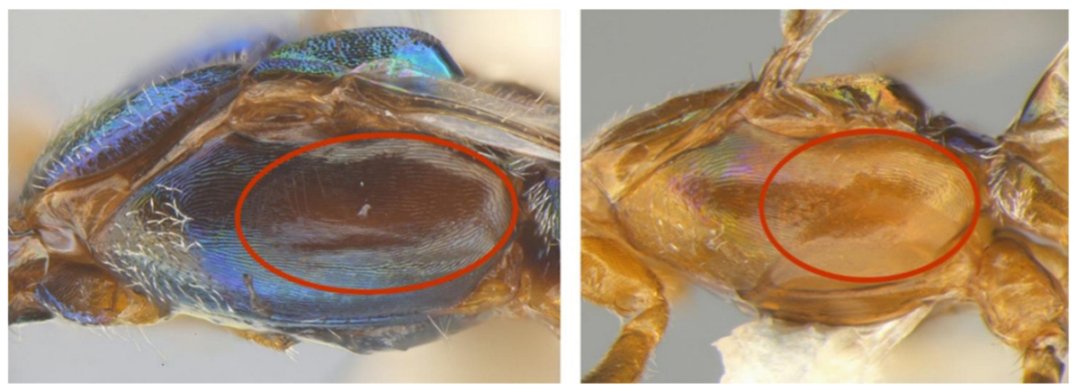

- Acropleuron with posterior portion uniformly as dark as mesoscutum [*fulloi* group] **·························································································································································29**

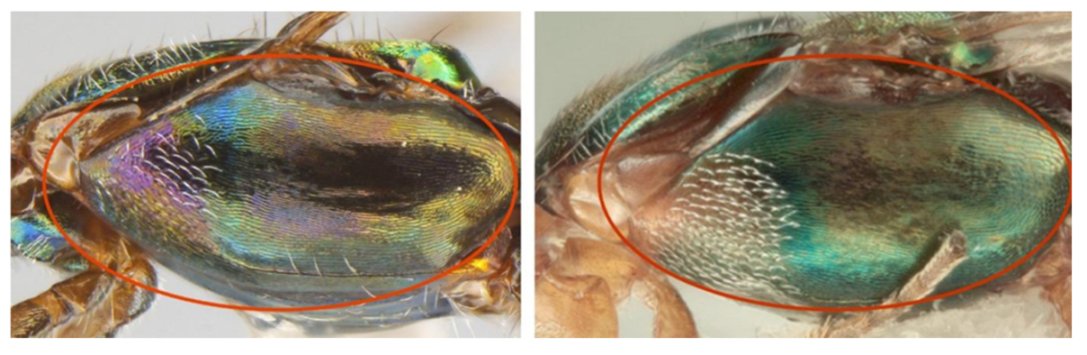

**22(21).** Acropleuron with only posterior third paler (brown) than dark mesoscutum **························································································*A*. (*Anastatus*) *caeruleus* Wang & Peng**

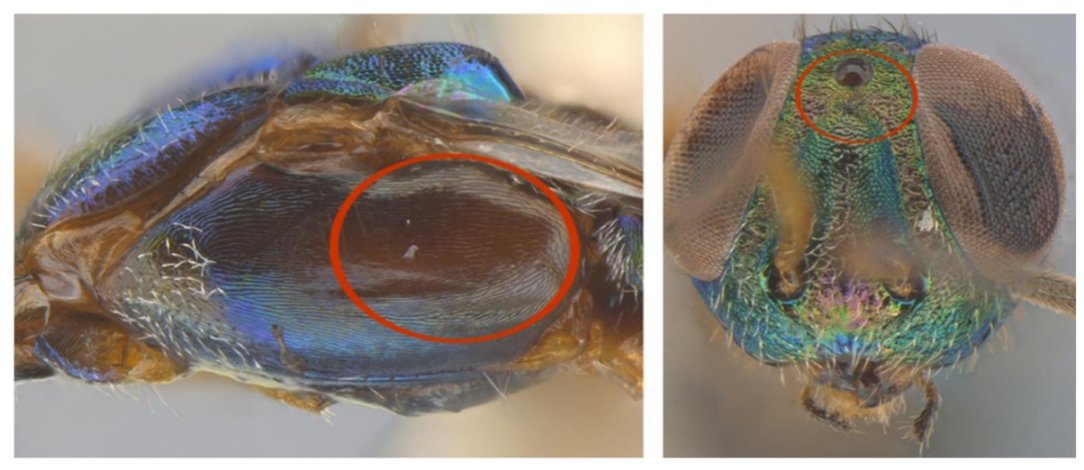

- Acropleuron distinctly paler than dark mesoscutum over at least about posterior half [*japonicus* group] **··························································································································23**

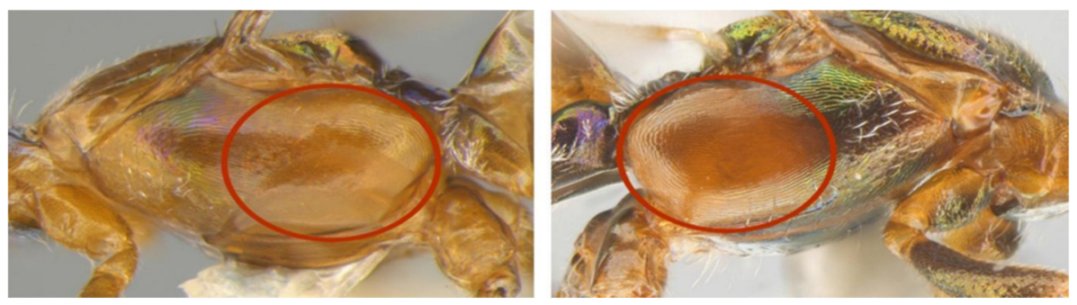

**23(22).** Posterior depressed region of mesoscutal medial lobe rough **···································24**

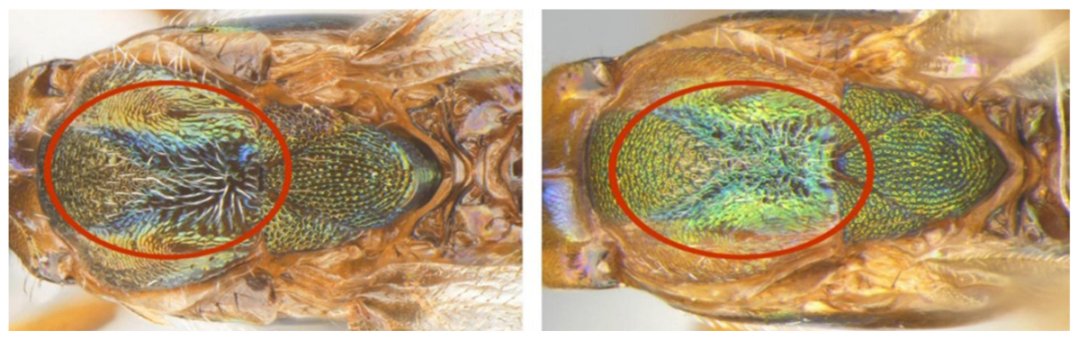

- Posterior depressed region of mesoscutal medial lobe smooth to rough **···························26**

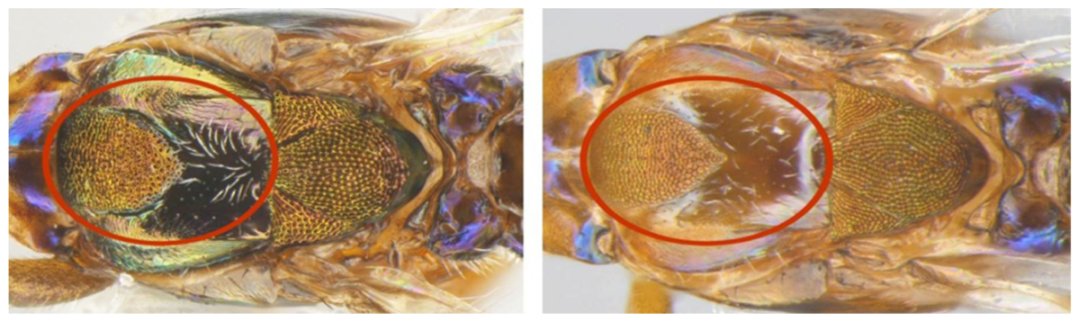

**24(23).** Gaster with dark brown to orangish transverse bands; fore wing hyaline crossband constricted medially with few dark setae **···························································································*A*. (*Anastatus*) *tigrinus* Khan, Li & Peng, sp. nov.**

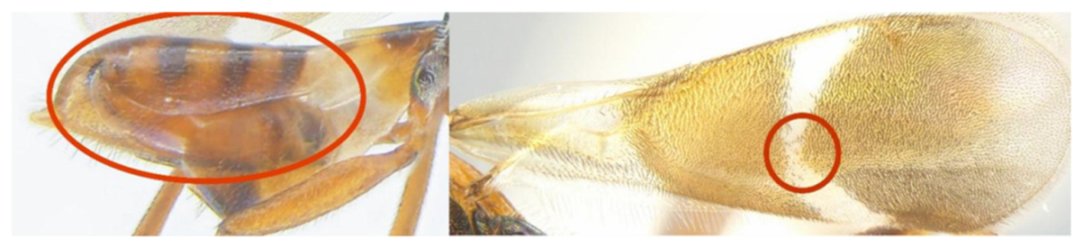

- Gaster without dark brown to orangish transverse bands; fore wing hyaline crossband without dark setae medially **·······································································································25**

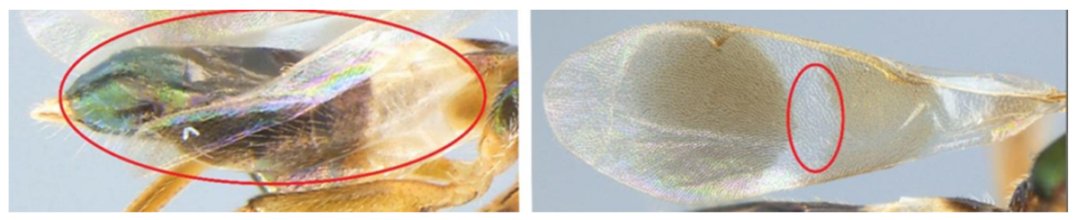

**25(24).** Mesoscutum mostly greenish, posterior depressed region of mesoscutal medial lobe with few white setae, acropleuron with mesopectus slightly dark greenish to violet spot anteriorly at upper end, though remaining part of acropleuron and profemur pale **·································································*A*. (*Anastatus*)** ***napoleonis*** **Khan, Li & Peng, sp. nov.**

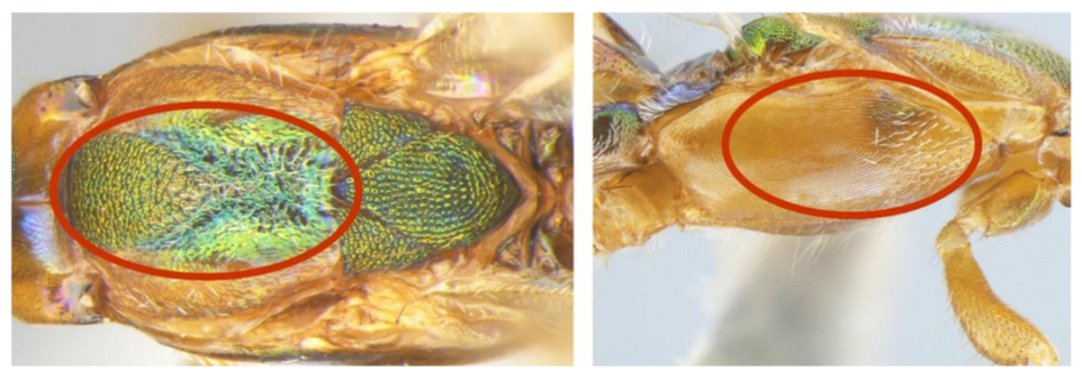

- Mesoscutum mostly dark brown with orangish to bluish metallic lustre, posterior depressed region of mesoscutal medial lobe setose with white setae, acropleuron with mesopectus and profemur mostly dark brown with green lustre **···································································*A*. (*Anastatus*) *huijuanae* Khan, Li & Peng, sp. nov.**

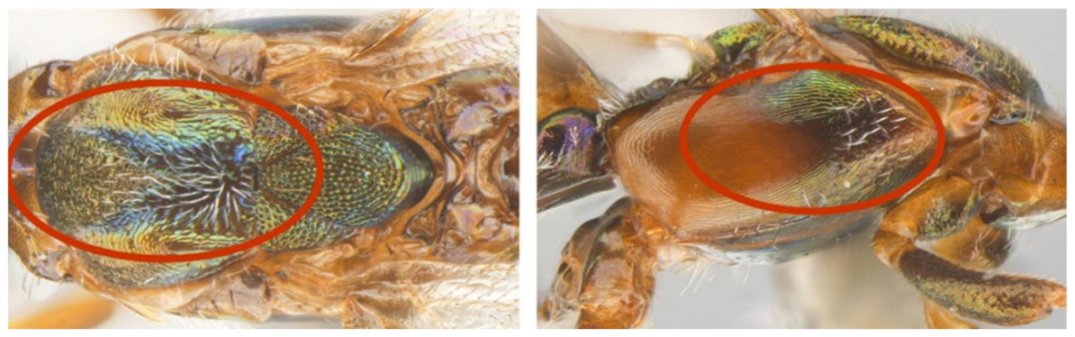

**26(23).** Basal cell of fore wing entirely bare; posterior depressed region of mesoscutal medial lobe smooth with few white setae **······················································································27**

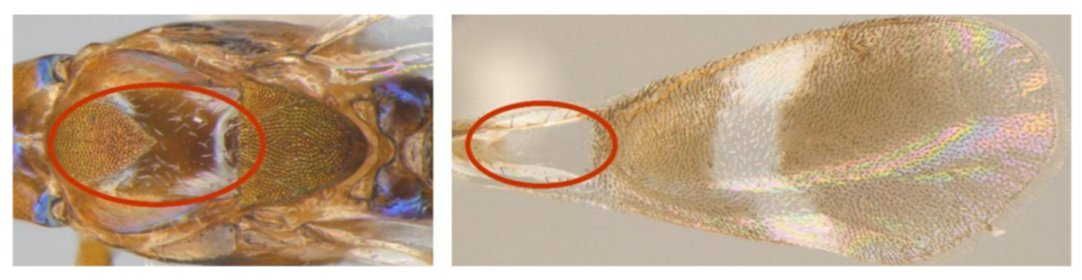

- Basal cell of fore wing not bare; posterior depressed region of mesoscutum not entirely smooth and densely setose with white setae **············································································28**

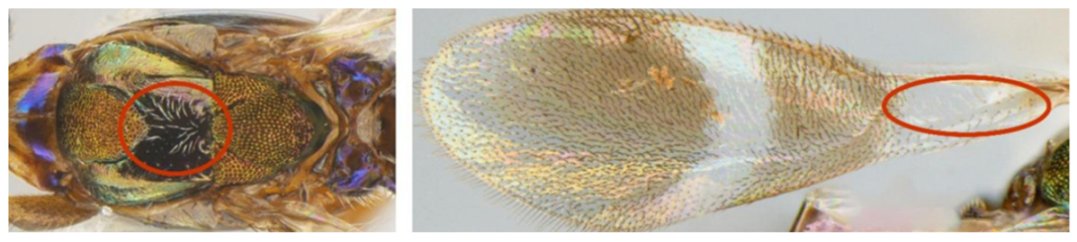

**27(26).** Posterior depressed region of mesoscutal medial lobe pale brown and setae scattered equally throughout the posterior lobe; callus of propodeum broader **··································································*A*. (*Anastatus*) *nozdormu* Khan, Li & Peng, sp. nov.**

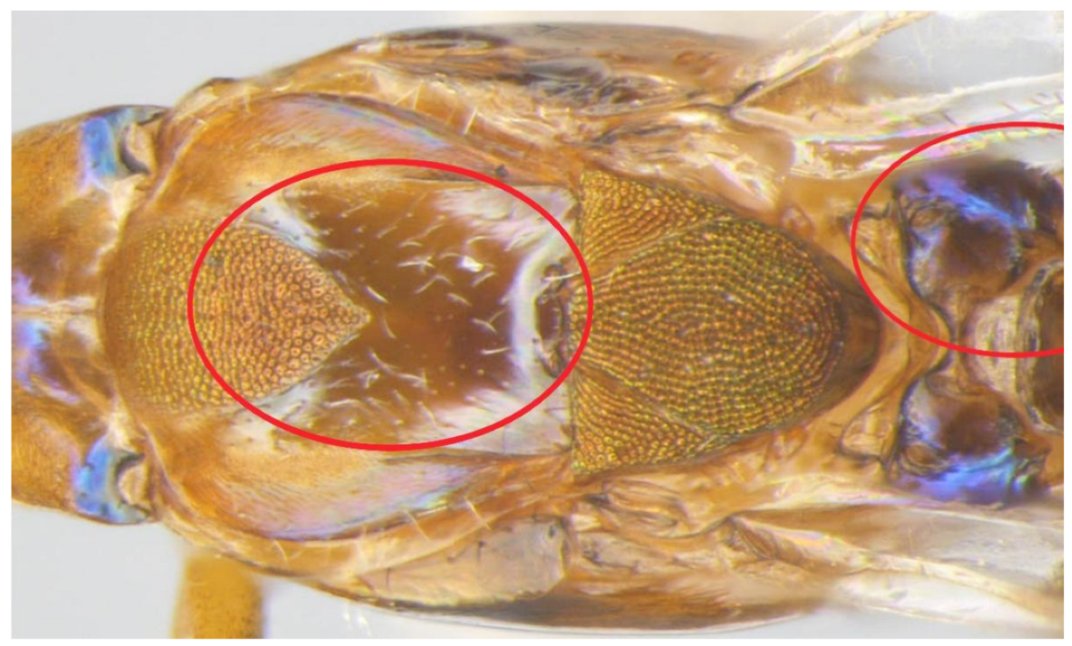

- Posterior depressed region of mesoscutal medial lobe with few white setae medially; callus of propodeum not as broad as mentioned above **··········································································*A*. (*Anastatus*) *yalini* Khan, Li & Peng, sp. nov.**

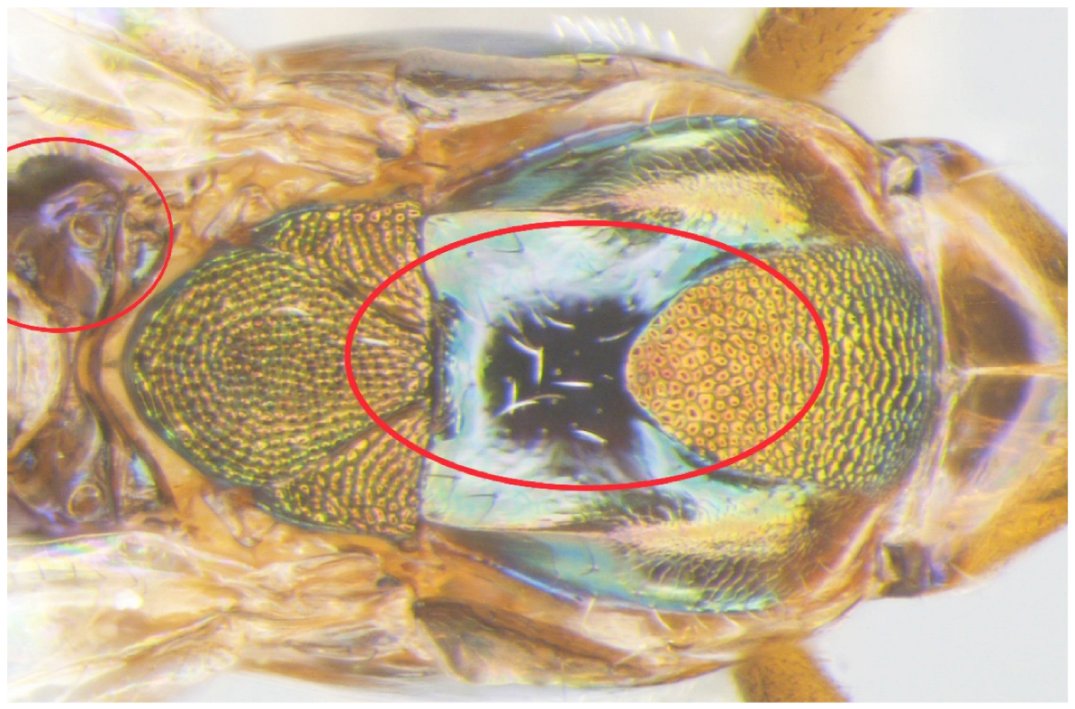

**28(26).** Head with upper parascrobal region mostly smooth to slightly coriaceous; fore wing hyaline crossband broader and straight **································································································*A*. (*Anastatus*) *beaglei* Khan, Li & Peng, sp. nov.**

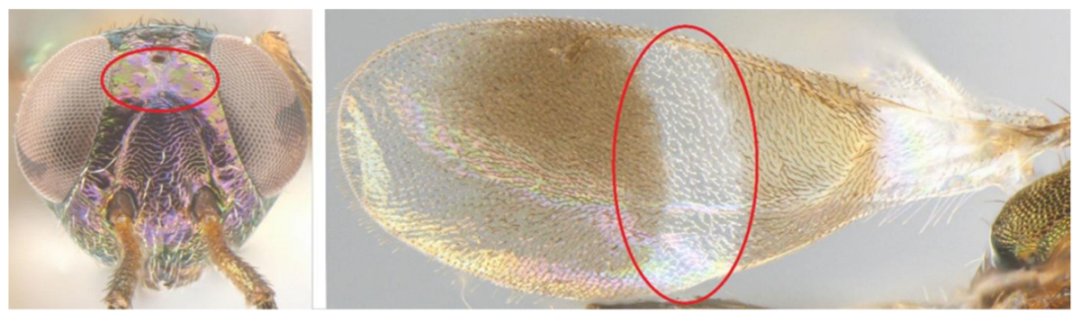

- Head with upper parascrobal region not smooth; fore wing hyaline crossband not as straight as mentioned above and slightly curved **·······························································································*A*. (*Anastatus*) *japonicus* Ashmead**

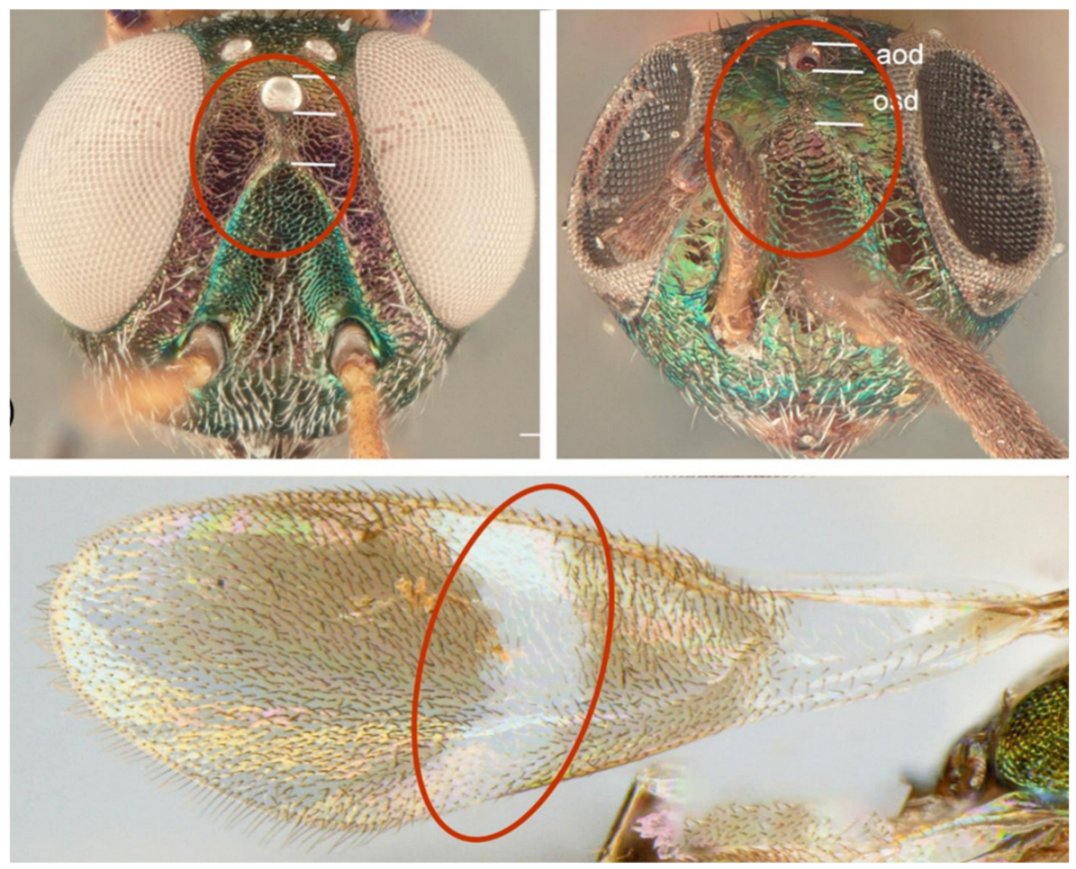

**29(21).** Face overall bright purple in frontal view except scrobal depression bluish green **·····················································································*A*. (*Anastatus*) *taibaiensis* Wang & Peng**

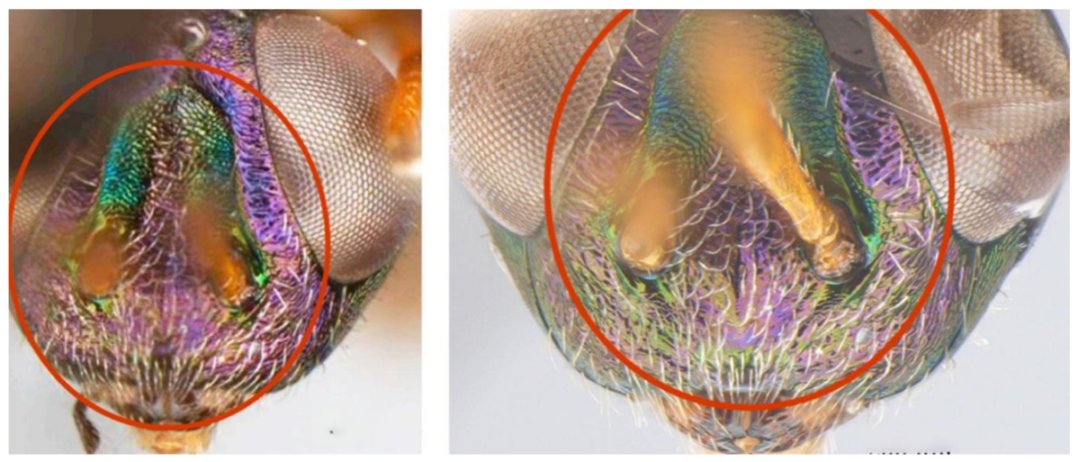

- Face mostly green with more-or-less purple or blue metallic lustre **···································30**

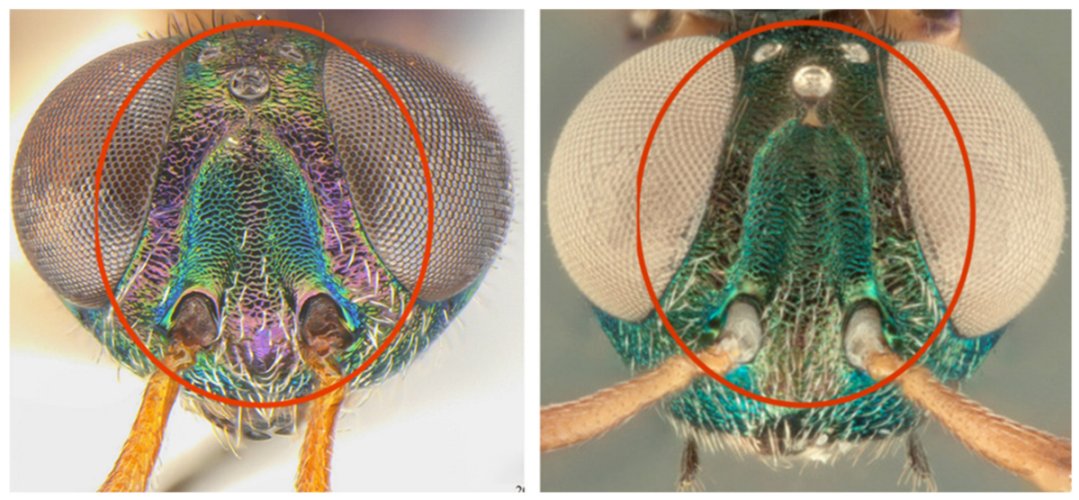

**30(29).** Flagellum with all funiculars longer than wide **···························································31**

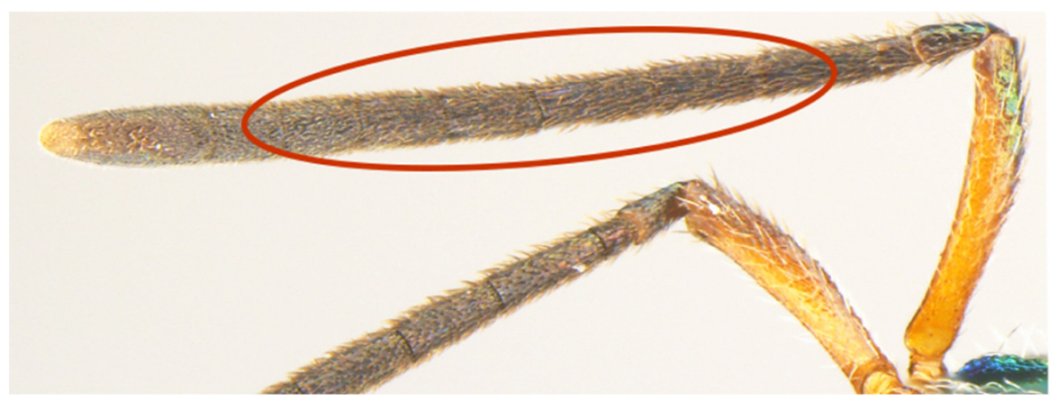

- Flagellum with at least apical three funiculars quadrate to slightly transverse **·················32**

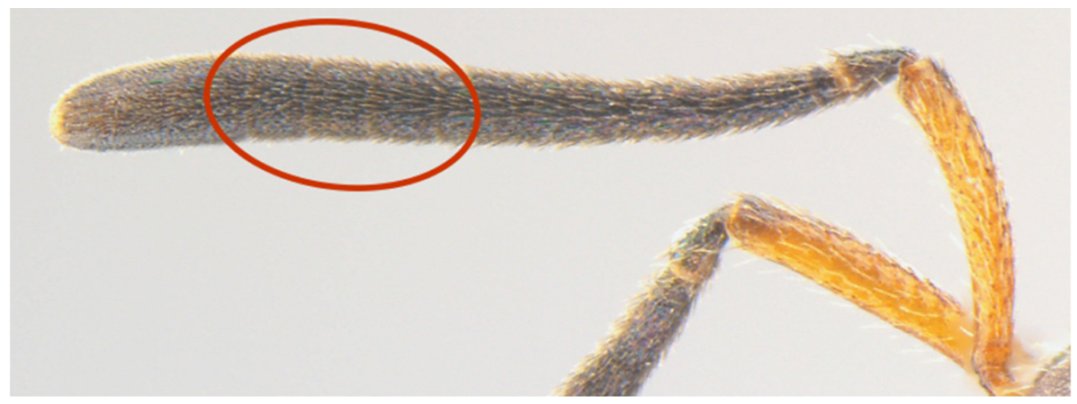

**31(30).** Posterior depressed region of mesoscutal medial lobe setose medially for a width about equal to width of bare region on either side; fore wing hyaline crossband constricted and sometimes like two spots, infuscate region width/hyaline crossband width ranges 3.0–4.2× **··············································································*A*. (*Anastatus*) *gansuensis* Chen & Zang**

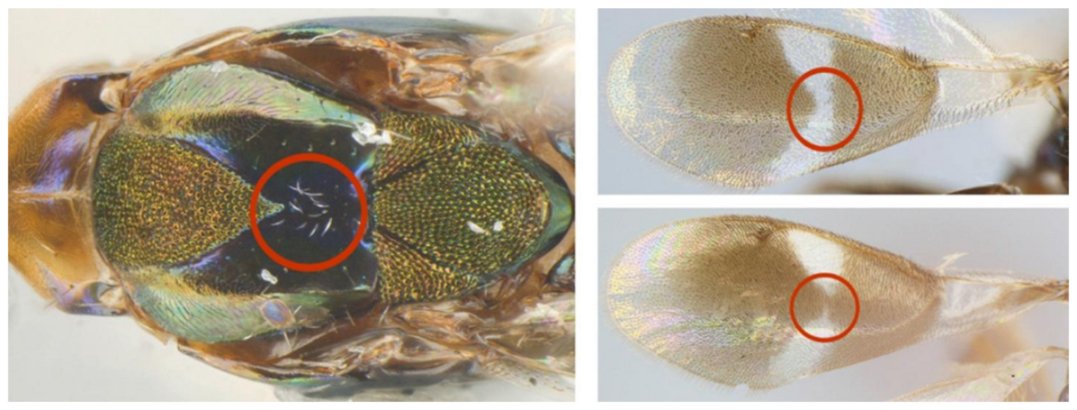

- Posterior depressed region of mesoscutal medial lobe setose for almost entire width; fore wing hyaline crossband broader, infuscate region width/hyaline crossband width range 2.0–2.8× **························································*A*. (*Anastatus*) *kryptos* Khan, Li & Peng, sp. nov.**

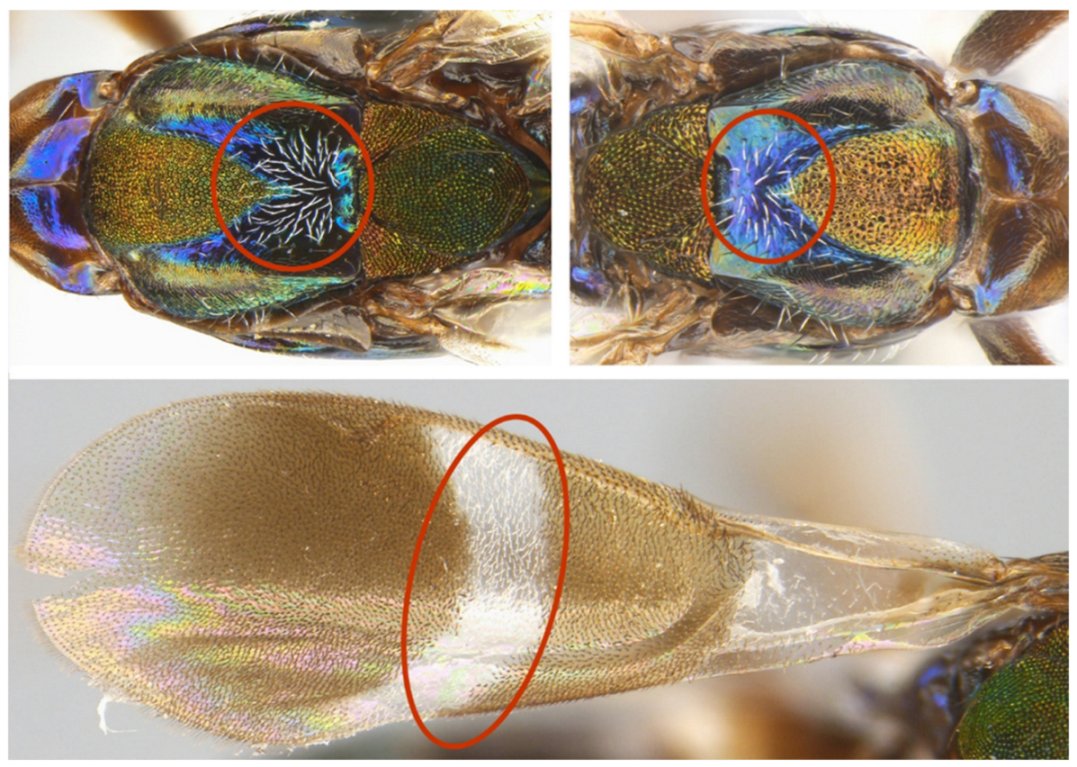

**32(30).** Mesotarsus uniformly yellowish-to-white in contrast to dark pegs; procoxa with at least ventral surface similarly pale to lateral panel of pronotum **·························································································*A*. (*Anastatus*) *orientalis* Yang & Choi**

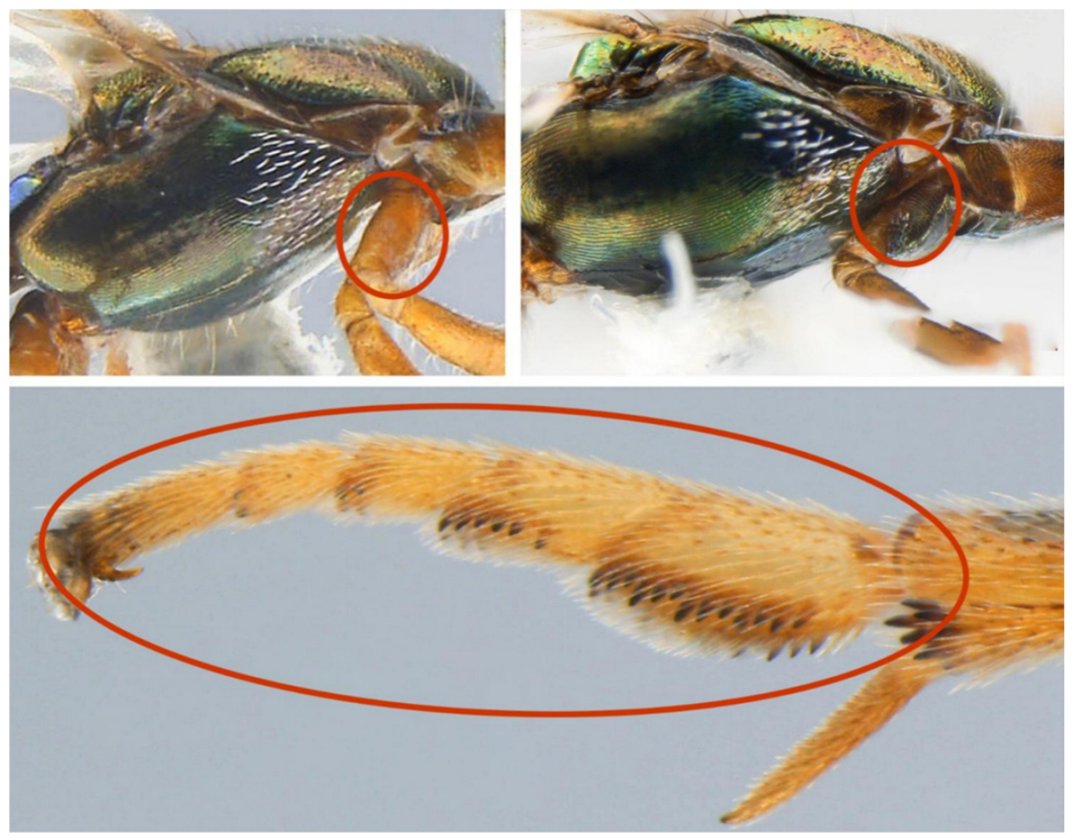

- Mesotarsus sometimes entirely infuscate, similar to dark pegs, but at least two basal tarsomeres variably conspicuously infuscate over at least dorsal and posterior surfaces; procoxa entirely dark, much darker than lateral surface of pronotum **·······································33**

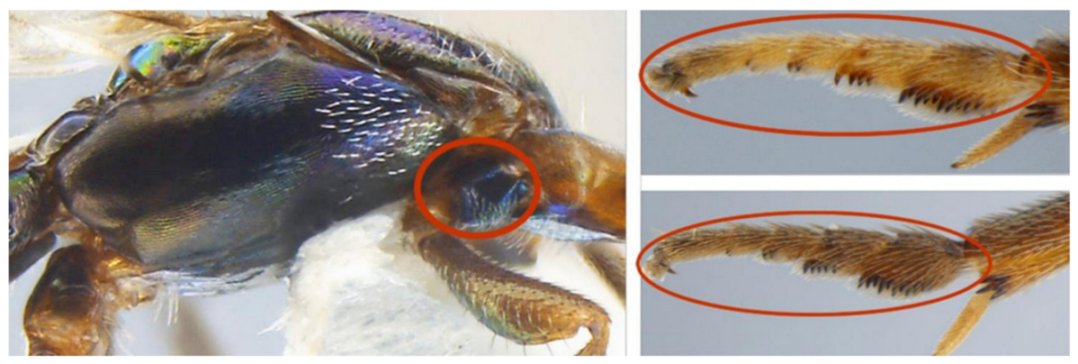

**33(32)**. fl2 width distinctly wider than fl1 width (width ratio ranges 1.12–1.28); overall body thin and long, gaster always longer than mesosoma **······································································*A*. (*Anastatus*) *battutai* Khan, Li & Peng, sp. nov.**

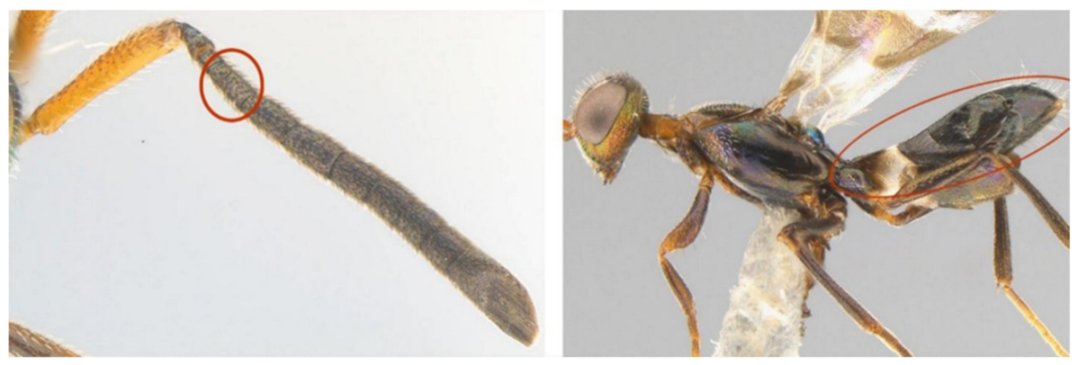

- fl2 width slightly wider or subequal to fl1 width (width ratio ranges 1.00–1.14); overall body not as thin as above, gaster shorter than mesosoma, though sometimes equals to or slightly longer but not always longer **·····························································································*A*. (*Anastatus*) *fulloi* Sheng & Wang**

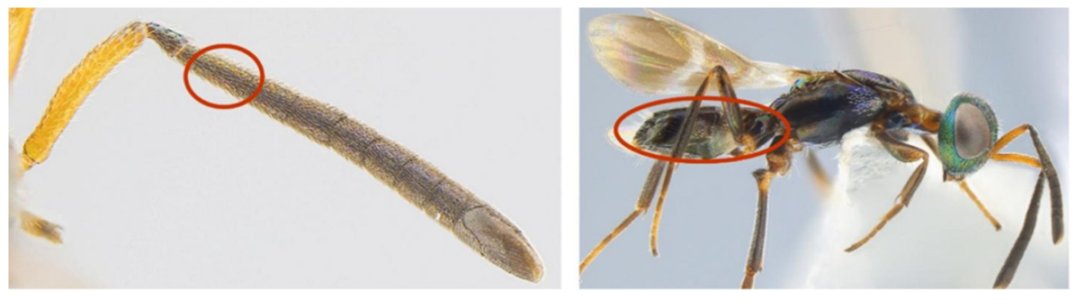


## 4. Discussion

The present study provides a comprehensive treatment of the genus *Anastatus* from the Palaearctic and Oriental regions of China, comprising 32 species. Among these, 11 species are described as new, with ten assigned to the subgenus *A*. (*Anastatus*) and one to *A*. (*Cladanastatus*). In addition, *A*. (*Anastatus*) *mantoidae* is recorded from China for the first time, and a new synonymy is proposed, placing *A. flaviratus* as a junior synonym of *A. gastropachae*. Furthermore, 15 species are newly recorded from different provinces of China.

The phylogenetic analysis contributed to species delimitation in several ways beyond merely confirming morphological species boundaries. First, reciprocal monophyly with strong bootstrap support provided an independent evolutionary criterion supporting the distinctness of ten newly described species and corroborating the validity of previously described morphospecies. Second, the tree topology and ASAP partitions revealed an intraspecific genetic structure within geographically widespread species (e.g., *A. makrysourus*, *A. shichengensis*), allowing explicit testing of whether such divergence corresponded to cryptic morphological variation; the absence of a correlated morphological or geographic structure led us to interpret these splits as intraspecific variation rather than cryptic species. Third, molecular data were instrumental in resolving taxonomic uncertainty, specifically by demonstrating that specimens previously identified as *A. flaviratus* were nested within the *A. gastropachae* clade, thereby supporting their formal synonymy. Fourth, comparison with BOLD sequences from Europe, Southeast Asia, and Africa enabled evaluation of whether Chinese populations represented endemic lineages or parts of more widely distributed species, thus extending species delimitation beyond Chinese borders. Finally, the phylogeny identified cases where *COI* divergence was insufficient to resolve species boundaries (e.g., *A. echidna* and *A. meilingensis*), highlighting where morphology must take precedence when molecular and morphological data are in conflict.

While the phylogenetic tree provided an evolutionary framework for species validation, some species pairs remained morphologically close; therefore, we have provided a detailed morphometric analysis for reliable diagnosis. Comparing the morphology of morphologically similar species allowed the recognition of three major groups—the *formosanus*, *fulloi*, and *japonicus* groups—which are mainly based on profemur tooth, dark procoxae, and acropleuron colouration. However, some species are even more closely related, including the pairs *A. gansuensis* and *A. kryptos*, and *A. fulloi* and *A. battutai*, as well as the triplet *A. dexingensis*, *A. formosanus*, and *A. garygibsoni*. For these taxa, we first sought unique characters based on morphology, and later also applied morphometric analyses to distinguish them. Diagnostic traits included the ratio of infuscate region width to hyaline crossband width for distinguishing *A. gansuensis* and *A. kryptos*, and the relative width of fl2_W (which showed the strongest discriminatory power) for separating *A. fulloi* and *A. battutai* (Figure 4, Figure 5 and Figure 6). Similarly, *A. garygibsoni* was distinguished from *A. dexingensis* and *A. formosanus* based on the ratio of the infuscate region width behind parastigma relative to the hyaline crossband (Figure 26 and Figure 27). While *A. dexingensis* and *A. formosanus* were very similar based on females, males provided clear diagnostic characters: in *A. dexingensis*, the costal cell of the fore wing is entirely setose, but less so in *A. formosanus*, or only the apical half is setose; additionally, the speculum of the fore wing is closed posteriorly by a distinct line of setae in *A. dexingensis*, whereas in *A. formosanus* it is indistinct. These morphological differences, together with *COI* sequence data, were used to distinguish them (Figure 22 and Figure 23).

Sequences of the *COI* gene were analysed for 34 species (including 26 from China and eight from other countries), but five singletons precluded intraspecific comparison. For the remaining 29 species with multiple sequences, independent intraspecific genetic divergence was calculated for 27 species and ranged from 0% to 7.8% (mean = 3%; median = 2.3%; SD = 2.35). Nearly half of all species (13/27) showed low genetic divergence (2% or even less), e.g., *A. bifasciatus* (0%), *A. neltharion* (0.2%), and *A. tigrinus* (0.3%), indicating that low mitochondrial divergence is the prevailing pattern. Conversely, five species exhibited markedly high genetic divergence: *A. makrysourus* (7.3%), *A. shichengensis* (7.8%), *A. kryptos* (6.5%), *A. huijuanae* **sp. nov.** (5.8%), and *A. garygibsoni* (5.5%). These correspond to the taxa that ASAP partitioned into multiple MOTUs, namely *A. makrysourus* (two MOTUs), *A. shichengensis* (three), and *A. huijuanae* **sp. nov.** (two). Thus, we treat these as intraspecific variation. A detailed distance matrix for all 325 sequences generated in Geneious Prime version 11.0.9+11 (64 bit) is provided in the Supplementary Data, and further details on pairwise comparisons can be found therein.

A contrasting pattern was observed for *A. echidna* and *A. meilingensis*: despite multiple sequences per species, their haplotypes were intermixed within a single clade (<4.7% total divergence) and ASAP delimited them as one MOTU, so *COI* entirely failed to resolve them. This likely reflects incomplete lineage sorting, slow mitochondrial substitution rates under metabolic constraints in small chalcids, or endosymbiont-mediated introgression [38]. Nevertheless, both species remain unequivocally diagnosable by stable morphological characters—*A. echidna* has a macropterous fore wing with two distinct hyaline spots (Figure 20E), whereas *A. meilingensis* has a brachypterous fore wing lacking any hyaline crossband or spot (Figure 32A). We therefore maintain both as valid, interpreting *COI* divergence as lagging behind morphological speciation.

Beyond these local species-boundary conflicts, the phylogeny also revealed broader geographic patterns when compared with publicly available sequences. Comparison of Chinese *Anastatus* with BOLD and NCBI sequences from Europe, Southeast Asia, and Africa (see Section 3.1) indicated that some Chinese species may be more widely distributed than previously recognized, although further authentication is required to confirm transboundary conspecificity. For instance, *A. gansuensis* and *A. catalonicus* are very closely related, with overlapping distribution, shared host associations, and similar fore wing hyaline crossband pattern. Molecular data (*COI*) also support their divergence, although this remains tentative, since *A. gansuensis* itself may have a broader distribution than currently recognized, requiring further clarification. However, the holotype of *A. catalonicus* is unavailable, diagnostic images are lacking, and BOLD identifications remain unverified as we were unable to obtain sequences from Spain (origin of *A. catalonicus*); these factors preclude formal synonymy, which is further explained in the remarks section of *A. gansuensis*. Their conspecificity therefore remains unresolved and will require integrative study with authenticated specimens from Spain.

In conclusion, species delimitation in *Anastatus* requires an integrative approach combining detailed morphology with multiple genetic markers. Despite this framework, *A. formosanus* and *A. dexingensis* still need to be resolved based on female morphology because females are predominantly used in mass-rearing and field release. For example, *A. dexingensis* was identified as an active parasitoid of the litchi stink bug in a recent publication on the composition of parasitoids from Guangdong, China. This species was nested within our *A. formosanus* clade [39]. Therefore, reliable female-based diagnostic characters are very important for practical identification in applied biological control programs. Future studies should incorporate nuclear genes such as *ITS2* and *EF-1α*, along with expanded geographic sampling, to better resolve species boundaries and assess cryptic diversity within the genus.

## Figures and Tables

**Table 1:** Multivariate analysis of antennal measurements, (*A. battutai* > *A. fulloi*) in flagellomere measurements, while (*A. fulloi* > *A. battutai*) in scape and pedicel measurements for AUC values. AUC (area under ROC (receiver operating characteristic) curve); df (degrees of freedom); *p* (probability)-value; t (t-statistic).

Variables	*A. battutai* (n = 7)	*A. fulloi* (n = 10)	t	df	*p*-Value	Cohen’s d	AUC
fl2_W	11.9 ± 0.6 (11.2–12.8)	10.8 ± 0.7 (10.0–11.4)	4.76	10	<0.001	2.50	0.97
fl4_W	16.3 ± 0.9 (15.1–17.9)	14.9 ± 1.5 (12.6–17.0)	2.34	15	0.033	1.08	0.80
scape_L	78.1 ± 5.9 (70.8–85.4)	85.3 ± 7.3 (70.0–99.2)	−2.20	15	0.044	1.04	0.79
ped_len	19.1 ± 1.7 (16.5–21.7)	21.6 ± 2.4 (17.7–24.9)	−2.40	15	0.030	1.11	0.80

## Data Availability

All DNA sequences are available in GenBank (COI accession numbers mentioned in Table S1).

## Supplementary Materials

The following supporting information can be downloaded at: https://www.mdpi.com/article/10.3390/insects17090918/s1, Figure S1: Distance matrix S1; Nucleotide alignment S1: ASAP species delimitation S1; Table S1: Sequences with accession numbers.

## References

[1] Wang Z., Zhou Y., Zou Y., Liu Q., Peng L. (2024). Seven New Species of *Anastatus* Motschulsky (Hymenoptera: Chalcidoidea: Eupelmidae) from China Identified Based on Morphological and Molecular Data. Insects.

[2] Ghosh D., Sheela S., Kumar P.G., Rameshkumar A. (2025). Description of Two New Brachypterous Species of *Anastatus* (Chalcidoidea: Eupelmidae) from India. Zootaxa.

[3] Li Z., Xu S., Wang Z., Chen P., Wu J., Peng L. (2025). Species of Eupelmidae (Hymenoptera: Chalcidoidea) from the Fujian Tianbaoyan National Nature Reserve, China. Zootaxa.

[4] Peng L., Gibson G.A.P., Tang L., Xiang J. (2020). Review of the Species of *Anastatus* (Hymenoptera: Eupelmidae) Known from China, with Description of Two New Species with Brachypterous Females. Zootaxa.

[5] Gibson G.A.P. (2020). Redescription of *Anastatus mantoidae* Motschulsky, the Type Species of *Anastatus* Motschulsky, 1859, and *Anastatus echidna* (Motschulsky), the Type Species of *Cacotropia* Motschulsky, 1863, with Respect to Taxonomy of *Anastatus* (Hymenoptera: Eupelmidae: Eupelminae). Zootaxa.

[6] Risbec J. (1952). Contribution à l’étude des Chalcidoïdes de Madagascar. Mém. Inst. Sci. Madag. (E).

[7] Bouček Z. (1979). Description of a New Eupelmid Parasite (Hymenoptera: Chalcidoidea) of Cockroaches in India. Bull. Entomol. Res..

[8] Gibson G.A.P. (1995). Parasitic Wasps of the Subfamily Eupelminae: Classification and Revision of World Genera (Hymenoptera: Chalcidoidea, Eupelmidae).

[9] Fusu L., Ebrahimi E., Siebold C., Villemant C. (2015). Revision of the Eupelmidae Walker, 1833 Described by Jean Risbec. Part 1: The Slide-Mounted Specimens Housed at the Muséum National d’Histoire Naturelle in Paris. Zoosystema.

[10] Stahl J., Babendreier D., Haye T. (2018). Using the Egg Parasitoid *Anastatus bifasciatus* against the Invasive Brown Marmorated Stink Bug in Europe: Can Non-Target Effects Be Ruled Out?. J. Pest Sci..

[11] Stahl J., Gariepy T., Beukeboom L., Haye T. (2019). A Molecular Tool to Identify *Anastatus* Parasitoids of the Brown Marmorated Stink Bug. Entomol. Exp. Appl..

[12] Broadley H.J., Sipolski S.J., Pitt D.B., Hoelmer K.A., Wang X., Cao L., Tewksbury L.A., Hagerty T.J., Bartlett C.R., Russell A.D. (2023). Assessing the Host Range of *Anastatus orientalis*, an Egg Parasitoid of Spotted Lanternfly (*Lycorma delicatula*) Using Eastern U.S. Non-Target Species. Front. Insect Sci..

[13] Gómez Marco F., Yanega D., Ruiz M., Hoddle M.S. (2023). Proactive Classical Biological Control of *Lycorma delicatula* (Hemiptera: Fulgoridae) in California (US): Host Range Testing of *Anastatus orientalis* (Hymenoptera: Eupelmidae). Front. Insect Sci..

[14] Chen Y.M., Qu X.R., Li T.H., Iqbal A., Wang X., Ren Z.Y., Desneux N., Zang L.S. (2021). Performances of Six Eupelmid Egg Parasitoids from China on Japanese Giant Silkworm *Caligula japonica* with Different Host Age Regimes. J. Pest Sci..

[15] Chen Y.M., Gong R.N., Wang X., Desneux N., Zang L.S. (2025). Assessing Potential for Biological Control of Japanese Giant Silkworm *Caligula japonica* Using *Anastatus gansuensis*, a Thelytokous Parasitoid Firstly Reported in Eupelmidae. Pest Manag. Sci..

[16] Kärrnäs E., Hansson C., Wahlberg N. (2025). Large-Scale DNA Barcoding Reveals Cryptic Diversity in Eulophid Wasps (Hymenoptera, Chalcidoidea, Eulophidae). Arthropod Syst. Phylogeny.

[17] Fusu L. (2017). An Integrative Taxonomic Study of European *Eupelmus* (*Macroneura*) (Hymenoptera: Chalcidoidea: Eupelmidae), with a Molecular and Cytogenetic Analysis of *Eupelmus* (*Macroneura*) *vesicularis*: Several Species Hiding under One Name for 240 Years. Zool. J. Linn. Soc..

[18] Gibson G.A.P. (2021). Revision of the Old World Genus *Mesocomys* Cameron (Hymenoptera: Eupelmidae). Zootaxa.

[19] Burks R.A., Gibson G.A.P., Heraty J.M., Heraty J.M., Woolley J.B. (2025). External morphology of adult Chalcidoidea. Chalcidoidea of the World.

[20] Folmer O., Black M., Hoeh W., Lutz R., Vrijenhoek R. (1994). DNA primers for amplification of mitochondrial cytochrome coxidase subunit I from diverse metazoan invertebrates. Mol. Mar. Biol. Biotechnol..

[21] Minh B.Q., Nguyen M.A.T., von Haeseler A. (2013). Ultrafast Approximation for Phylogenetic Bootstrap. Mol. Biol. Evol..

[22] Nguyen L.-T., Schmidt H.A., von Haeseler A., Minh B.Q. (2015). IQ-TREE: A Fast and Effective Stochastic Algorithm for Estimating Maximum-Likelihood Phylogenies. Mol. Biol. Evol..

[23] Kalyaanamoorthy S., Minh B.Q., Wong T.K., von Haeseler A., Jermiin L.S. (2017). ModelFinder: Fast Model Selection for Accurate Phylogenetic Estimates. Nat. Methods.

[24] Zhang D., Gao F., Jakovlić I., Zou H., Zhang J., Li W.X., Wang G.T. (2020). PhyloSuite: An Integrated and Scalable Desktop Platform for Streamlined Molecular Sequence Data Management and Evolutionary Phylogenetics Studies. Mol. Ecol. Resour..

[25] Seabold S., Perktold J. Statsmodels: Econometric and statistical modeling with Python. Proceedings of the 9th Python in Science Conference (SciPy 2010).

[26] Pedregosa F., Varoquaux G., Gramfort A., Michel V., Thirion B., Grisel O., Blondel M., Prettenhofer P., Weiss R., Dubourg V. (2011). Scikit-Learn: Machine Learning in Python. J. Mach. Learn. Res..

[27] Waskom M.L. (2021). Seaborn: Statistical Data Visualization. J. Open Source Softw..

[28] Virtanen P., Gommers R., Oliphant T.E., Haberland M., Reddy T., Cournapeau D., Burovski E., Peterson P., Weckesser W., Bright J. (2020). SciPy 1.0: Fundamental Algorithms for Scientific Computing in Python. Nat. Methods.

[29] Cohen J. (1988). Statistical Power Analysis for the Behavioral Sciences.

[30] Harris C.R., Millman K.J., van der Walt S.J., Gommers R., Virtanen P., Cournapeau D., Wieser E., Taylor J., Berg S., Smith N.J. (2020). Array Programming with NumPy. Nature.

[31] Hunter J.D. (2007). Matplotlib: A 2D graphics environment. Comput. Sci. Eng..

[32] Wiśniowski B., Jirak-Leszczyńska A. (2021). Six Species of Eupelmid Wasps (Hymenoptera: Chalcidoidea, Eupelmidae) New to Poland and New Records of Another Two Species. Acta Entomol. Sil..

[33] Lotfalizadeh H., Parsa M. (2018). On the Occurrence of Anastatus catalonicus Bolívar y Pieltain (Hym.: Chalcidoidea, Eupelmidae) in the East-Palaearctic. Proceedings of the 23rd Iranian Plant Protection Congress, Gorgan, Iran, 27–30 August 2018.

[34] Chen Y.-M., Gibson G.A., Peng L.-F., Iqbal A., Zang L.-S. (2019). *Anastatus* Motschulsky (Hymenoptera, Eupelmidae): Egg Parasitoids of *Caligula japonica* Moore (Lepidoptera, Saturniidae) in China. ZooKeys.

[35] Bolívar y Pieltain C. (1934). Estudio monográfico de las especies españolas del género Anastatus Motsch. (Hym. Chalc.). Eos.

[36] Askew R., Nieves-Aldrey J. (2004). Further Observations on Eupelminae (Hymenoptera, Chalcidoidea, Eupelmidae) in the Iberian Peninsula and Canary Islands, Including Descriptions of New Species. Graellsia.

[37] Askew R.R. (2005). A New European Species of Anastatus Motschulsky (Hym., Eupelmidae). Entomol. Mon. Mag..

[38] Klopfstein S., Kropf C., Baur H. (2016). *Wolbachia* Endosymbionts Distort DNA Barcoding in the Parasitoid Wasp Genus *Diplazon* (Hymenoptera: Ichneumonidae). Zool. J. Linn. Soc..

[39] Li Z., Song M.-H., Li W.-Q., Zu G.-H., Lü X., Chen H.-Y. (2026). Parasitoid Composition of the Litchi Stink Bug *Tessaratoma papillosa* (Hemiptera: Tessaratomidae) from Guangdong Province of China. J. Appl. Entomol..

