# Supplementary material for: Integrative Taxonomic Study of Chinese Anastatus (Hymenoptera: Eupelmidae) Based on Morphology and COI Barcoding, with Description of Eleven New Species
Source: Insects. 2026 Sep 1;17(9):918. doi: 10.3390/insects17090918 (PMC13607660; doi:10.3390/insects17090918)

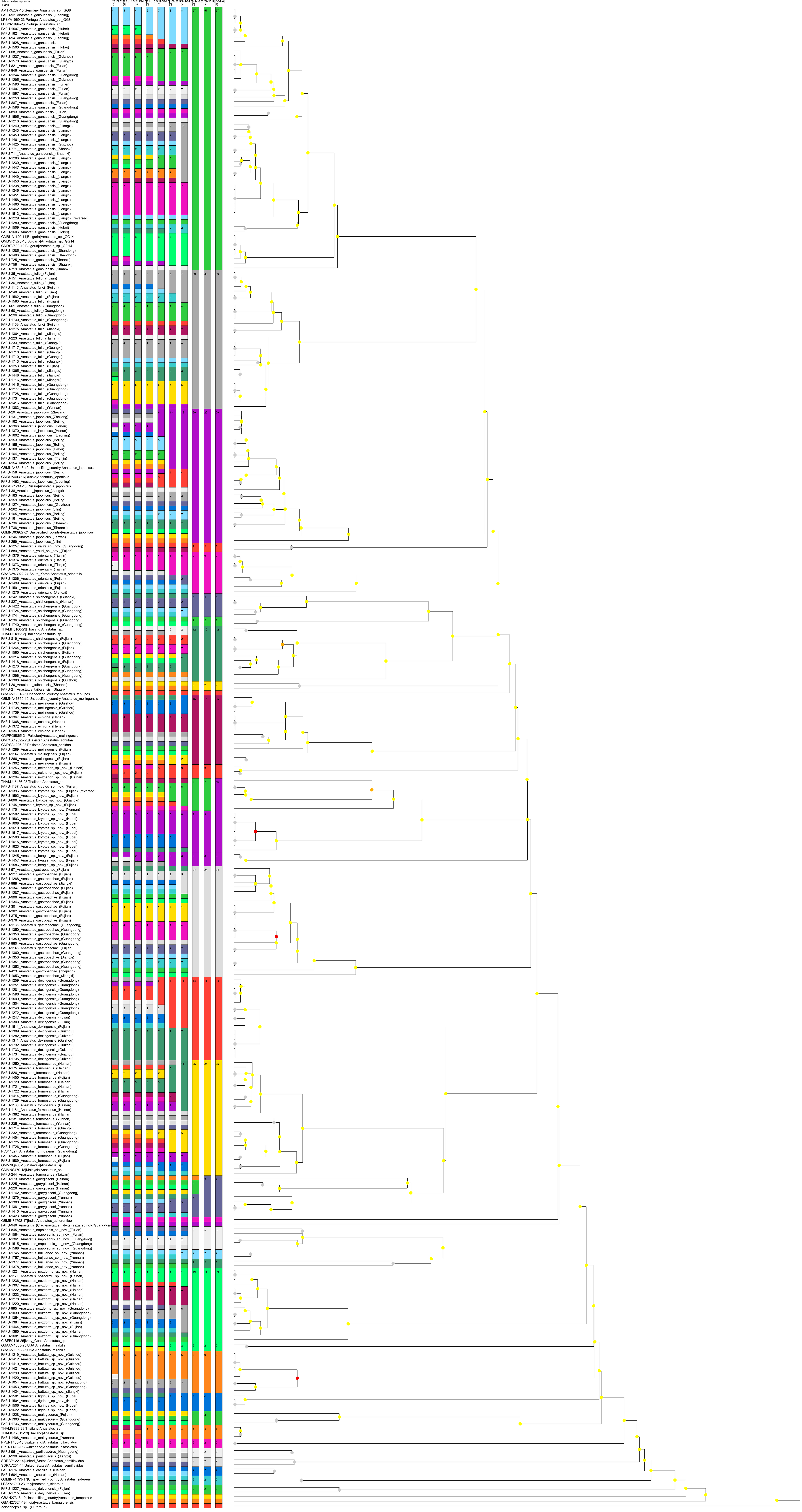

Supplement: Supplementary file 1 [file insects-17-00918-s001.zip › Nucleotide alignment.tab.groups.pdf]
